# Evolution of Tonal Organization in Music Optimizes Neural Mechanisms in Symbolic Encoding of Perceptual Reality. Part-2: Ancient to Seventeenth Century

**DOI:** 10.3389/fpsyg.2016.00211

**Published:** 2016-03-30

**Authors:** Aleksey Nikolsky

**Affiliations:** Braavo! EnterprisesLos Angeles, CA, USA

**Keywords:** ancient Babylonian and Greek music, diatonic/chromatic music, modulation and alteration, musical texture and pictorial perspective, musical key and pictorial perspective, environmental topography and tonal organization, pitch zone, aesthetic emotion

## Abstract

This paper reveals the way in which musical pitch works as a peculiar form of cognition that reflects upon the organization of the surrounding world as perceived by majority of music users within a socio-cultural formation. Part-1 of this paper described the origin of tonal organization from verbal speech, its progress from indefinite to definite pitch, and the emergence of two main harmonic orders: heptatonic and pentatonic, each characterized by its own method of handling tension at both domains, of tonal and social organization. Part-2, here, completes the line of historic development from Antiquity to seventeenth century. Vast archeological data is used to identify the perception of music structures that tells apart the temple/palace music of urban civilizations and the folk music of village cultures. The “mega-pitch-set” (MPS) organization is found to constitute the principal contribution of a math-based music theory to a new diatonic order. All ramifications for psychology of music are discussed in detail. “Non-octave hypermode” is identified as a peculiar homogenous type of MPS, typical for plainchant. The origin of chromaticism is thoroughly examined as an earmark of “art-music” that opposes earlier forms of folk music. The role of aesthetic emotions in formation of chromatic alteration is defined. The development of chromatic system is traced throughout history, highlighting its modern implementation in “hemiolic modes.” The connection between tonal organization in music and spatial organization in pictorial art is established in the Baroque culture, and then tracked back to prehistoric times. Both are shown to present a form of abstraction of environmental topographic schemes, and music is proposed as the primary medium for its cultivation through the concept of pitch. The comparison of stages of tonal organization and typologies of musical texture is used to define the overall course of tonal evolution. Tonal organization of pitch reflects the culture of thinking, adopted as a standard to optimize individual perception of reality within a social group in a way optimal for one's success, thereby setting the conventions of intellectual and emotional intelligence.

## Introduction

Part-1 of this paper presented the framework for study of tonal organization^1^ in any kind of music. Based on the available data from archeology, anthropology, ethnomusicology and psychoacoustics, the known forms of tonal organization were lined out in a timeline, where the cognitive constraints of perception of different musical typologies were used as criteria for deciding which form of organization came first. The pattern of acquisition of music skills during infancy was used to hypothesize the succession of stages in separation of music from speech and descent of definite pitch organization from indefinite one. The existing types of indefinite-in-pitch music were analyzed to identify *khasmatonal* and *ekmelic* modes as specialized methods of processing indefinite pitch. Mechanisms of their evolution into *oligotonal* definite-pitch mode were defined. The principle of triadic induction was shown to determine the growth of oligotonal into mesotonal, and mesotonal into multitonal schemes. The resulting hemitonic *heptatony* and anhemitonic *pentatony* presented two alternative methods of organizing vertical and horizontal harmony—each offering a dedicated style of handling tonal tension—reflecting a more general style of worldview, based on the parallels between tonal tension and social tension. Commitment to heptatony or pentatony as the principal means of tonal organization within a culture, then, appears to generally correspond to the preferred lifestyle in a social group. This correspondence could be the product of abstraction of individual lifestyle preferences into the tonal schemata of a *musical mode*, and further mediation of the multitude of such modes within a social group - until the statistically prevailing mode would establish the model of tonal organization.

Part-2 continues drawing the lineage until the rise of Western tonality, identifying yet another venue of musical representation of perceptual reality—vertical and horizontal tonal structures encoding the perceived relation of multiple objects in one's surrounding. The spatial organization of depicted images appears to share the same principles as the tonal organization of music in the same culture, probably originating in its environmental topography. Spatial-to-tonal correspondence is the strongest in Western tonality, but is noticeable at earlier stages defined in Part-2: *diatonic* and *chromatic mega-pitch-set* (MPS) systems, and *non-octave hypermode*.

## Genesis of modal family and the role of tetrachord

What separates prehistoric and historic forms of music is the emergence of math-based music theory and notation. Notation encourages production of complex compositions in observation of theoretic rules, and restrains discrepancies in reproduction of the same tune. Oral transmission of folk music, in contrary, employs variation as the leading music-making principle. Any information technology designed to enhance the retention of symbolic information should be regarded as stimulation for the emergence of abstract thought (Couch, 1989).

Musical implementation of abstraction was the inference of *modal family* from a *single mode*. The model of it is documented in cuneiform notation of the Hurrian Hymns and related texts from Ugarit ca. 1400 BC. They reveal that Assyrian/Babylonian music was heptatonic, based on 7 modes^2^ named after a particular series of 5ths that were used to generate each of the modes (Kilmer and Tinney, 1996).

1. Audio: An arrangement of the Hurrian Hymn No. 6, Anne Kilmer's transcription (1974). Kilmer's dyadic interpretation (Kilmer, 1974) was criticized for a number of inconsistencies with the data from the recovered music theory texts (West, 1994). http://bit.ly/1jttPpG
2.Audio: the alternative transcription by West (1993), which also was criticized (Crocker, 1997). http://bit.ly/1IVNBTM
3. Audio: the alternative transcription by Dumbrill (1998). Despite huge differences between the transcriptions of this single surviving sample of Babylonian music, together with the retrieved music theory texts, it provides substantial information on general principles of tonal organization. http://bit.ly/1KqPzJO

Our “Mixolydian G” formed the base of Babylonian system^3^. Prioritization of Mixolydian mode is known in numerous Eurasian folk music systems (Belaiev, 1963). Mesopotamian music theory must have adopted it from folk tradition and “rasterized” it mathematically, adopting the tetrachord as the formative tool in modal genesis.

Eurasian instrument-makers have traditionally conceptualized ambitus through equivalence of 4th, which according to Beliayev (1990, p. 248) manifests “the first stage of maturity” in tonal organization—supporting professionalization of folk musical culture. Modal integrity of 4th was epitomized in the Pythagorean cult of Tetraktys (“quaternary”), originating in primitive cultures of the Bronze Age (Burkert, 1972, pp. 188–191), likely in Babylon (Barbera, 1984)^4^.

**Figure 1:**
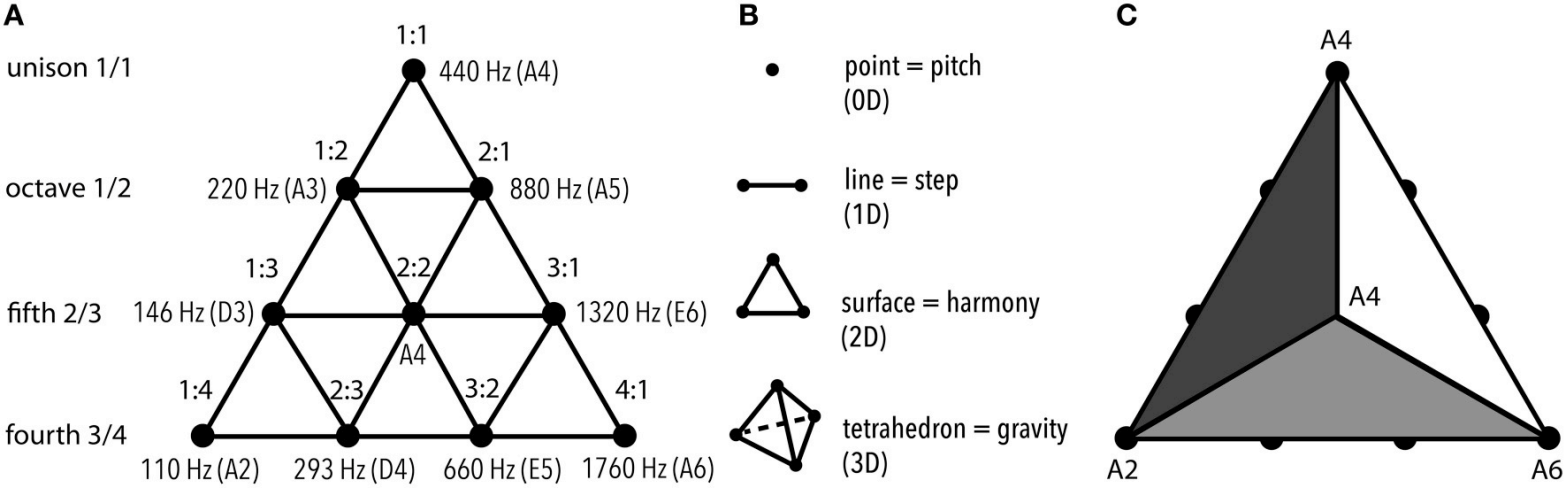
**Tetraktys (the “fourness”): the geometric representation of harmonicity of “4th.” (A)** Musical aspect of tetraktys. On the left, each row is assigned a value that expresses the ratio of the string required for production of a perfect musical interval. Numbers 1, 2, 3, and 4 follow one another sequentially. Each row contains intervals of the designated type. On the right, the tetraktys diagram is combined with the lambdoma matrix (Bruhn, 2005, p. 67) that was associated with Pythagorean teaching in Ancient Greece (Hero and Foulkrod, 1999). The lambdoma ratios are applied to the frequency value of A4 (440 Hz) taken as 1:1, producing A, D, and E—the unmutable (hestote) tones that comprised the skeleton of the Ancient Greek musical system. Obviously, the harmonic aspect of tetraktys determined the tonal organization of Greek and probably earlier civilizations. **(B)** Spatial aspect and its melodic correspondence. Each row represented a parameter in spatial representation: 1 point indicated zero dimensions; 2 points defined line, 1 dimension; 3 points defined surface, 2 dimensions; 4 points—a 3D geometric figure. Musically, this would be equivalent in distinguishing between an isolated pitch, a melodic step produced by succession of 2 pitches, a particular harmony produced by the relations of 3 pitches, and a gravitational frame of reference generated by 4 pitches—evident if to look at pitch values at any of the sides of the tetraktys in **(A)**. **(C)** Centripetal gravity encoded in tetraktys. The configuration of 10 points that comprise the tetraktys can represent 3D by implying 4 triangles. Then, their “corners” correspond to the same octave-equivalent pitch, indicating that the tetraktys is “tuned” to a certain tone **(A)**.

Tetraktys was the *earliest rational conceptualization of spatial and tonal organization* in a single scheme (Figure 1) of an equilateral triangle filled up with symmetrical rows of 1 to 4 dots. Each of these numbers encoded a geometric concept: 1—point, 2—line, 3—surface, and 4—tetrahedron—everyone of which contained the one before it (Riedweg, 2005).

Together, they represented world's order, where point symbolized unity, line—limit, surface—harmony, and tetrahedron—cosmos. This convention displayed amazing vitality throughout the ages, nourishing philosophy of Christian, Arabic, and Jewish traditions (McCartin, 2010). Since Ancients considered numbers “sounding”—following the paradigm of proportional shortening of the string length to attain different pitches, the idea of inclusiveness of a number was understood musically as well (Barbera, 1977): 4 and its integers expressed perfect consonant intervals (2:1 = octave, 3:2 = fifth, and 4:3 = fourth). Musical Tetraktys then served as the *container of harmony*: intervals that could be expressed in numbers not greater than 4 were considered “*symphonia*” (accord), whereas all other intervals, including 3rd and 6th, were considered “*diaphonia*” (discord) (Kholopov, 2006, p. 64). Tetraktys determined the assortment of pitches usable in music, which was also understood in cosmogonic terms. Plato, in Timaeus, described the derivation of music tuning as creation of the World-Soul—and the model for his calculations probably came from earlier Mesopotamian sources (Crickmore, 2009a).

Byzantine, Arabic, Persian, Indian, Syriac, Armenian, Georgian, and Western European music theories—as well as many Eurasian folk traditions—share the tetrachord base. Sachs (1962, p. 163) notes near-omnipresence of 4th in world's music: absent only in Polynesia and Micronesia. Cultural proliferation and longevity of 4th indicate cognitive reasons for its prominence.

Ancient Greek theory may offer a hint for possible explanation. Greeks conceived intervals in terms of stepwise singing male voices: thence, 4th was a sum of 3 steps and an aggregate of the constituent intervals: 2nd and 3rd, each of which carried its own psychological features. Greeks must have been aware of the displacing tendency of a 2nd^5^. Therefore, perception of melodic 2nd involved competition between two tones—a kind of duality—as opposed to monadic unison. Going over the step produced a leap of a 3rd that contrasted a 2nd by leaving a trace and thereby introducing a new dimension of vertical harmony. Whenever enclosed in a melodic 3rd, two 2nds, did not create friction, making the 3rd indeed the expression of “harmony”. Increasing the leap by another step produced a 4th which contained a 2nd and a 3rd—two intervals of different valence, one implying duality (disharmonious) and the other, accordance (harmonious). Such 4th encapsulated all basic tonal relations: *sameness, otherness*, and their synthesis—*concordance*.

This intervallic numerology could date back to Aurignacian culture, where the first cosmogonic concepts were forged in terms of solar/lunar, day/night, male/female dialectics, usually expressed in 2:1, 3:1, and 2:3 proportions (Frolov, 2003). Babylonian philosophy coordinated these proportions and expressed them mathematically as well as musically.

The integrating capacity of a 4th manifested itself in tetrachordal organization. This is still evident in maqam where the II and III tones inside a tetrachord can shift in their tuning values as needed, whereas the marginal tones remain permanently locked (Zannos, 1990). The inclusive power of such tetrachordal 4th is quite obvious to musicians, especially on a string instrument. And string instruments played a formative role in crystallization of the earliest epic Indo-European tradition. Four pitches that corresponded to melodic stresses of the ancient Sanskrit and Greek bridged Rig Veda to Homer - illuminating presence of 4 PCs by the look of a 4-string lyre, used to accompany epic singing (West, 1981). The idea of a tetrachord would then simply be the abstraction of a tunable lyre's string with marginal tones locked by the 3:4 proportion.

Optimization of interval-tracking could explain the preference for tetrachordal organization: most individuals cannot track more than four simultaneously moving entities (Drew and Vogel, 2008). This becomes an issue in heptatonic music-making, where 4th often stands as a “collection” of 4 tones, each of which requires attention and memory. Storing auditory images for each pitch—as incremental representation by a lookahead feature of the brain's error-detection circuitry—occurs while singing a familiar melody in one's mind (Janata, 2012). Facility of quick arithmetic estimations (1+1+1+1 etc.), necessary for vocal coordination, could make 4th into an optimal melodic size “chunk.”

A 4th also provides the best compromise between melodic and harmonic consonance (see Part-1): vertical 4th fuses well, while horizontal 4th might not segregate in very fast tempo (Huron, 2001)^6^. Its closest competitor in size is 3rd, but 4th has a serious taxonomic advantage of being a *perfect* interval: for trained musicians, narrowing or widening a 4th by about 12 cents reduces its recognizability, making the listeners hear it as another interval, whereas for major 3rd the tolerance zone is 50% wider, about 18 cents, and for minor 3rd—25 cents (Burns and Ward, 1978). Furthermore, the tuning zone for 4th, enclosed between 3rd and 5th (that are harmonically contrasting to 4th) is substantially narrower than for the range occupied by 3rds (enclosed between the dissonant 4th and 2nd). According to Moran and Pratt (1926), 4th enjoys the lowest deviation rate amongst all intervals, with 13.5 cents average. This makes 4th a better tuning reference than 3rd, which in history of acoustics has been notorious for exorbitance of tuning standards and preferences (Barbour, 2004). Listeners' resolution of interval size is the highest for 4th (43 cents)—exceeding the 5th (50 cents)—and presenting an asymmetric bias towards the 3rd: major 3rd, extended 37% toward 4th, is still heard as a 3rd, whereas 4th allows for only 18% extension toward 3rd (Shackford, 1962).

Preference for 4th might have a developmental origin—there is evidence that mother-to-infant vocalizations during the first 2 years of life tend to tonally tune to the harmonic row of the same fundamental (85% of communication), where 4th is the *only* interval used outside the row, as an “infrafix” below the fundamental (Van Puyvelde et al., 2010). Such frame of reference would favor unison and 4th as the smallest size perfect consonances, comfortable for vocalization—and tetrachord exactly sets the unison and 4th as the structural axis for melody-making.

**Figure 2:**
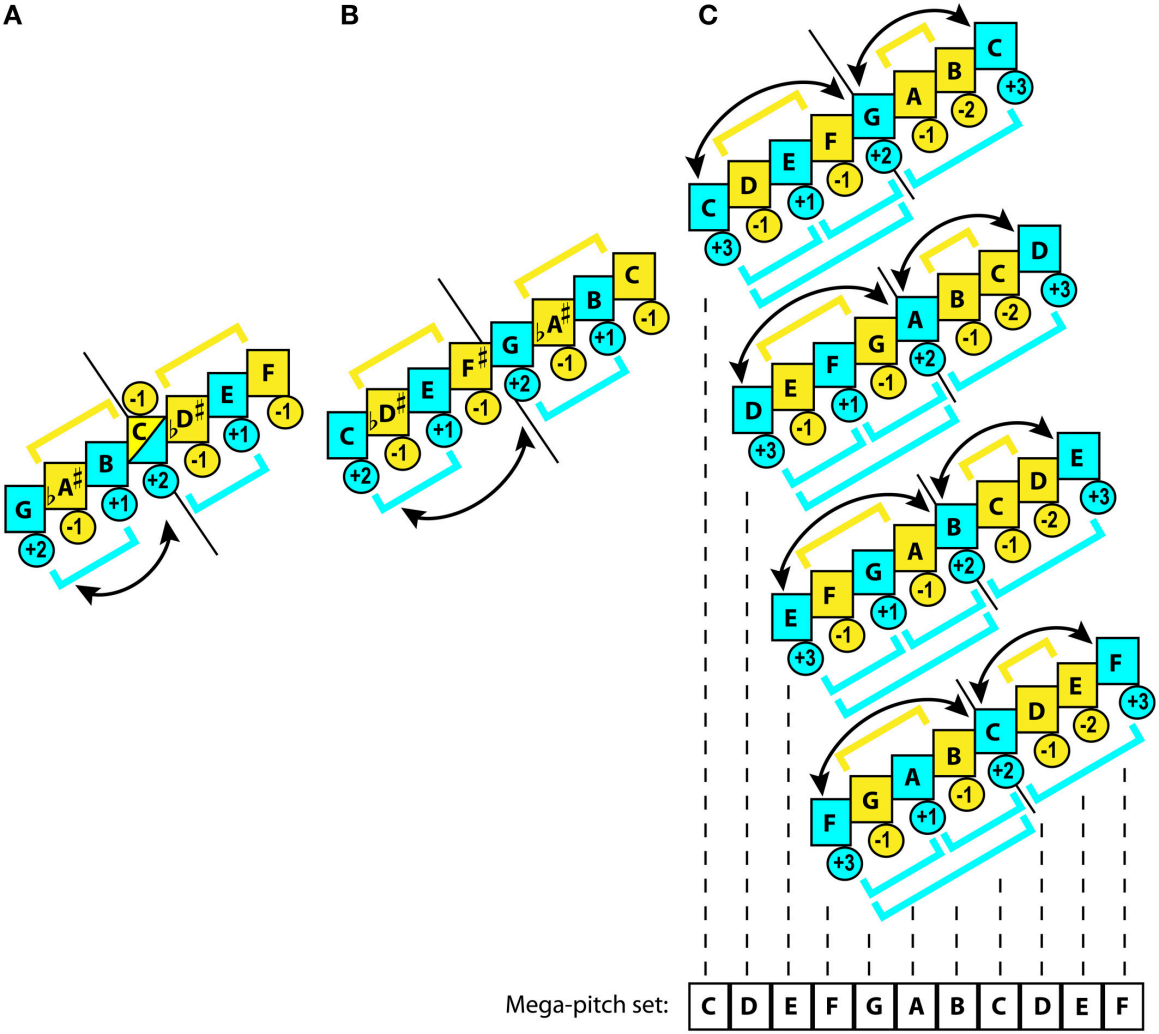
**Genesis of Mixolydian polymodal family**. Blue color marks stability, while yellow—instability. Brackets illustrate hierarchic grouping of degrees. Arrows indicate the tendency of tones to alternate as gravitational centers. Figures in circles rate relative gravitational values within the subset: negative indicate instability (−2 more unstable than −1), while positive—stability (+3 more stable than +1). Short black straight line shows the central axis for a mode formed by modal subsets within a mode. Curved arrowed lines indicate the shifts of gravity between the mutable anchors. Placement of this line between two tones indicates a disjunct connection. Placement under the middle of a tone indicates a conjunct connection between the subsets. The dashed black lines display the mega-set membership of the pitches. **(A)** Mixolydian conjunct heptatonic non-octave mode. The mode has 2 equivalent subsets with gravitational ambiguity in the center. The IV degree alternates from stable to unstable state, depending on the tetrachord in which it is melodically engaged. The hierarchy is limited to the binary distinction between stable degrees, where the lowest tone of the tetrachord receives greater weight. Modal mutability occurs between I and IV degrees. **(B)** Rotated Mixolydian disjunct heptatonic non-octave mode. This mode has two equivalent subsets with unambiguous gravitational map, where the lowest tone in the tetrachord remains the anchor. Although, its hierarchy is as elementary as in **(A)**, this mode is closer to the uni-tonicity. Modal mutability here occurs between I and V degrees in the manner of “tonic”-“dominant.” Also equivalence of 4th produces nearly perfect match in tuning between I and VIII degrees. **(C)** Ionian octave equivalent diatonic mode and its three most common modal transpositions: Dorian (D), Phrygian (E), and Lydian (F). This mode features two inequivalent subsets with contrasting placement of unstable degrees. In the lower pentachord they alternate with stable degrees, whereas in the upper tetrachord they are entangled between the stable margins. Both subsets are conjunct through the V degree, which thereby receives greater modal prominence than the III degree. Also, the upper tetrachord is harmonically understood as the “inversion” of the basic pentachord in reference to the stable degrees. The mode is fully octave equivalent. The very same modal scheme is reproduced in every transposition (C, D, E, F). “Sameness” of the pitch values (i.e., D = D) across the sister-modes despite their difference in functionality for each of the sister-modes generates the MPS.

For Eurasian instrumental music, expansion of ambitus usually occurs by addition of an extra tetrachord above the initial one. Mixolydian tetrachord presents a choice par excellence because of its ease of tuning on the string and on the pipe (tone+tone+semitone)^7^, and uniformity of its conjunct reproduction: G-A-B-C+C-D-E-F (Figure 2A).

The second favorite is the Phrygian tetrachord (semitone+tone+tone)^8^, which is simply an intervallic inversion of Mixolydian. Disjunct addition of this tetrachord forms a Phrygian mode: E-F-G-A+B-C-D-E, which was probably a later development^9^.

Each of these tetrachords constitutes a characteristic 4-tone intonation. Perceptually, the progression of two whole-tones, followed by a semitone, designates a melodic vector: Mixolydian tetrachord suggests ascent, whereas Phrygian—descent. Both are found amongst the world's most widespread modes (Gill and Purves, 2009). Singing them creates illusion of “resolution” toward the upper or lower 4th. A semitone is known to project directional ascending/descending melodic motion (Roederer, 2008, p. 184). Delviniotis et al. (2008) discovered that performers habitually increase the first interval length, and proportionally decrease the last in ascending scales, while inversing this treatment in descending scales—which would emphasize the vector of melodic inertia.

Melodic tetrachord highlights the gravitational relations and suggests spatial concomitants.

- Pentatonic conjunction of trichords “rounds the corners” by avoiding sharp-sounding minor 2nds and projecting concordance;
- Heptatonic conjunction of Phrygian or Mixolydian tetrachords amplifies ascending or descending directionality of the resultant scale, connoting insistence and purposefulness.

Chronologically, induction of Mixolydian mode must have preceded octave equivalence. The Mixolydian conjunct mode is *non-octave* in its design (Beliayev, 1990, p. 281), and is characterized by alternation in gravity between the base tones of both tetrachords. The IV degree here tends to change from stable to unstable state, depending on the tetrachord in which it is melodically engaged. Like pentatony, this mode lacks gravitational hierarchy, but features greater tension, since its unstable tones tend to shift closer to a stable degree in expressive tuning, usually employed by performers (Morrison and Jánina, 2002).

According to Beliayev (1990, p. 288), conjunct Mixolydian produces a mode equivalent to Ionian via tetrachord rotation. This pseudo-Ionian remains *non-octave*, since its disjunct tetrachords make the upper C unstable whenever mutable^10^ G temporarily becomes “tonic.”

In practice of earlier oligotonal and mesotonal music, singers commonly used transposition-by-*interval*^11^.

4. Audio: Udasan Yryata, healing incantation. Occasionally, transposition was applied to a particular portion of a song as deliberate expressive means. Transposition of the oligotonal PS: from F-Ab-Bb to G-Bb-C (between the 2nd and 3rd strophes). http://chirb.it/NcLnD1

Heptatonic modes promoted transposition-by-*degree*^12^, introducing the pitch-class set (PCS) concept. The underlying idea of diatonicity originates from cultivation of string plucking instruments (Belaiev, 1963)—which were cardinal for Mesopotamian civilization (Lawergren, 1997). The visibility of strings, each easily equated to a pitch class (PC), and correspondence between the string length and interval size makes a mode obvious to players. Facility of producing few tones simultaneously prototypes the observation of vertical intervals that emerge between vocals and instrumental accompaniment.

5. Audio: Maddoh, Pamir. The accompaniment on rubab (6-string lute) provides an example of vertical 2nds occasionally produced by plucking the adjacent strings. http://bit.ly/1kOmO49

This is exactly what Nippur music-instruction tablets specify: notation of vocal part with lyrics set against the pitches of the lyre (Colburn, 2009)—for the first time graphically exhibiting the dimension of musical texture.

## Formulation of the mega-pitch-set

The next development occurred when the triad induction (see Part-I) caused to re-conceptualize the lower tetrachord plus a tone above it as *pentachord* (258), forging a concept of melodic intonation of 5th as a modal unit^13^, and introducing a new hierarchic layer I-III-V into a mode. Pseudo-Ionian *non-octave* mode then transfigured into *Ionian octave* mode, with the mutability I-V instead of I-IV. The new axis of I-V pioneered the “authentic” functionality, in light of which the older I-IV axis could be viewed as “plagal.” The novelty of the authentic relationship was that it typically supported a melodic development that would build a climax point and emphasize the prevalence of “tonic” at the end. Krohn et al. (2007) confirmed that the largest N1 component in the ERP corresponded to hearing the V degree of the major key^14^.

With pentachordal scheme in place, musicians begin reproducing a succession of the same tones from the II rather than the I degree—turning II into the new I degree—and filling up the upper end with an extra tone. Such *transposition-by-degree* creates a “sister” 7-tone mode, with identical pentachord hierarchy that shares the PS (C-D-E-F-G-A-B-C and D-E-F-G-A-B-C-D), uniting both modes into a single system. There is experimental evidence that listeners categorize such modes by ear despite their identical PCs (Rohrmeier and Widdess, 2012).

It is not an accident that the three closest Mixolydian transpositions (Dorian, Phrygian, and Lydian)^15^ top the interval set (IS) harmonicity list of the world most popular heptatonic modes (Gill and Purves, 2009). Also sister-modes “harmonize” the music repertoire by making all songs share the same intervals classes (ICs). This “pan-harmonization” separates the partially octave-equivalent multitonal mode of “village” music from the completely octave-equivalent mode of the “palace” modal system. Their difference is manifested in the presence of *mega-pitch-set (MPS)*: a set of tones, legitimized as the building material for any musical composition by music theory.

The larger is the set, the greater is the harmonization, and therefore the greater is the *stretch of gravity*, causing overall reduction in tension. The earliest Sumerian harps had 11-15 strings, which by the eighteenth century increased to 29 strings (Lawergren, 1997). The ambitus of music performed on such harps greatly exceeded that of the typical folk heptatonic music, easing tension—appropriately for meditation in temple, and eulogy in palace. The “easing up effect” distinguishes MPS from earlier folk heptatonic forms.

**Figure 3:**
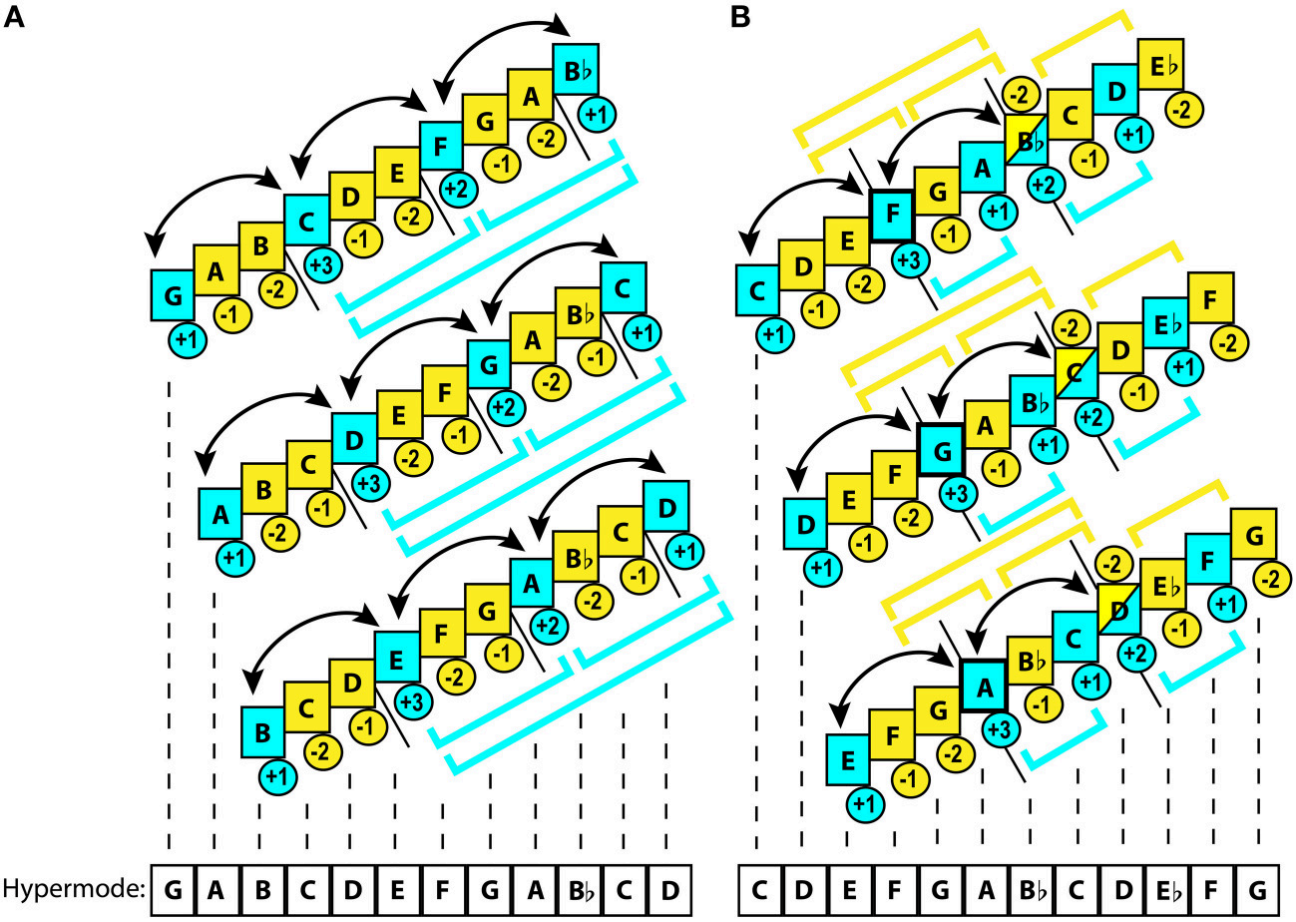
**Different types of non-octave hypermode.^27^** Yellow color represents unstable, while blue—stable degrees. Brackets illustrate hierarchic grouping of the degrees. Arrows mark the tendency of the tones to alternate as gravitational centers. Figures in circles rate relative gravitational values within the subset. Short black straight lines mark the modal subsets within a hypermode: when a line is placed under the middle of a tone, this tone serves as an anchor for two subsets, generating the conjunct connection between them. **(A)** Three obykhodnyi hypermodes of the hexáechos system: major (G), minor (A), and diminished (B). This hypermode features nearly perfect *subset equivalence*. Hierarchical organization is present only for stable degrees, where the lowest pair (G-C) often generates a plagal inclination. The highest stable degree (Bb) is hardly ever used as finalis, which usually falls on the central anchors (C or F). Unstable degrees do not form any groups, as in pentatony. However, tension here is weaker than in pentatony because of deeper 3-level hierarchy of stable degrees. Position of a semitone marks the “leading tone.” Anchor points relate to each other by 4th, readily forming the 4th–chord “triads” that are treated like consonance. **(B)** Three Georgian tetrachordal hypermodes: major (C), minor (D), and diminished (E). Unstable tones in the center of the hypermode provide modal integrity by forming unstable dyads (E-G, G-Bb), entrapping the central tonic dyad (F-A). Sometimes this tonic dyad alternates in gravity with the upper tetrachord's anchors (Bb-D). Whenever this happens, Bb shifts from unstable to stable function. However, the hypermode remains centripetal due to the harmonic dissonance of the diminished octave E/Eb, which generates a melodic inertia toward the inward resolution. The lowest tetrachord executes a complimentary plagal function. The utmost upper tetrachord usually keeps its highest tone unstable, leaning on its lowest tone. Altogether, the tetrachords feature *subset inequivalence*: gravitational contrast between each other (unlike the trichord hypermode). Tonal mapping of the major hypermode (centripetal F) serves as a prototype for the minor (G) and diminished (A) versions.

**Figure 4:**
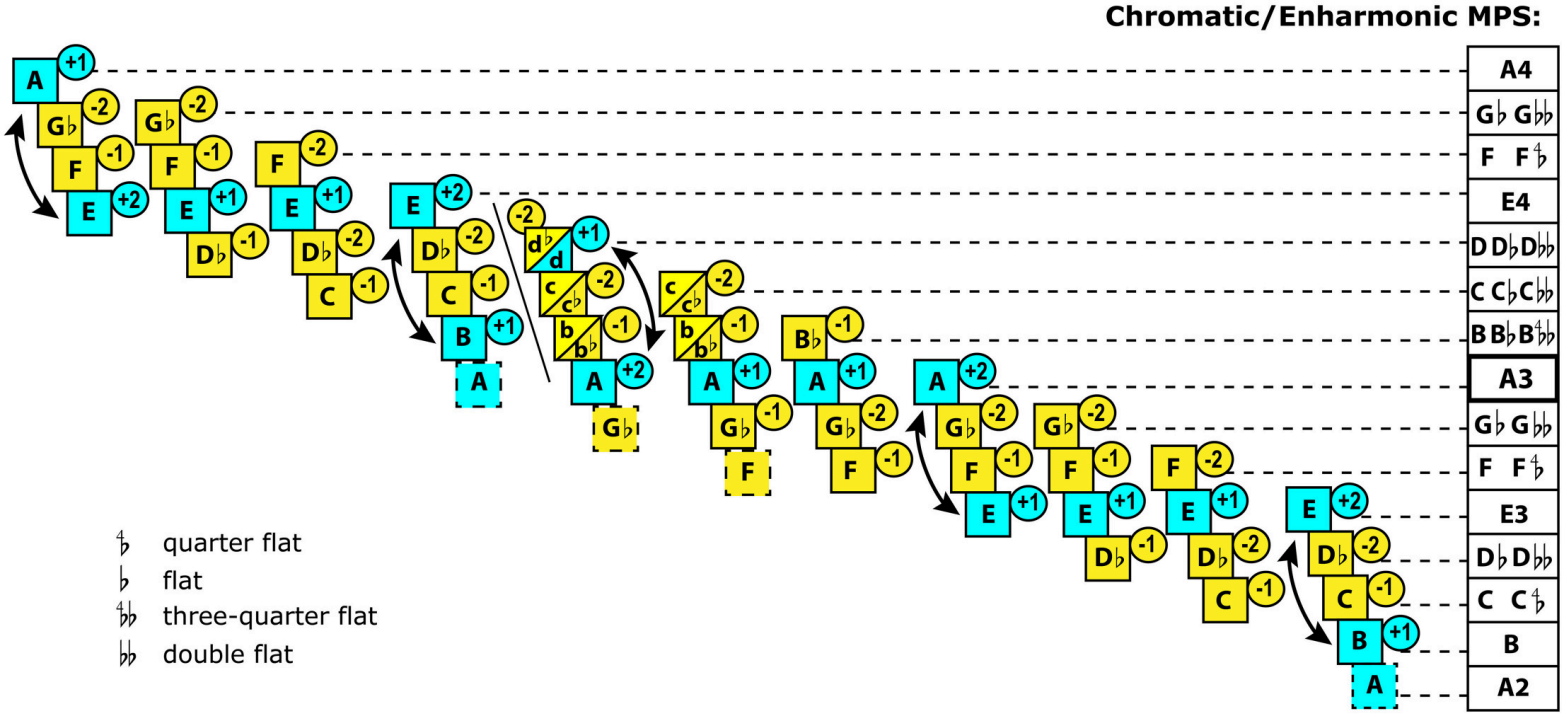
**Chromaitc system according to Cleonides (Aristoxenian school), c.1st century BC**. This system contains 11 subsets based on rotation of fixed diatonic tetrachords. Blue color marks the permanent degrees that were associated with stability. Yellow color marks the mutable degrees that required resolution. The tonal tension rating, marked in the circles, is my estimation based on Beliayev's method^40^. Mese (A3) is assigned greater stability in tetrachords with two stable tones whose “tonic”/“dominant” interaction is marked by an arrow. E3 and E4 are assigned greater stability than the marginal stable tones (B & A4). Of the two unstable tones, the upper one always involves alteration, whereas the lower one retains its “diatonic” state in the chromatic genus—suggesting subordination of the upper tone to the lower one. The black diagonal line marks *diezeuxis*—the break between the tetrachord *meson* (E3-A3) and tetrachord *diezeugmenon* (B3-E4). This break leaves no common tones between these tetrachords in the chromatic/enharmonic genera (B-C-Db-E vs. A-Bb-Cb-D). In order to enable a smooth melodic transition across *diezeuxis*, Cleonides reserves three pentachords at strategic points (A-B-C-Db-E, Gb-A-B-C-E, and F-Gb-A-B-C), which significantly complicates the Systema Teleion (which engages two upper tetrachords, B-E and E-A) as well as the Systema Metabolon (which, instead, terminates the MPS at D with the tetrachord *synemmenon* A-Bb-C-D). The lowest pentachord adds a stand-alone A2 in order to accomplish the A/E modal framework. The chart reflects the *chromatic* genus. The column on the right summarizes the pitch values for the chromatic as well as *enharmonic* genera of the unstable tones. The enharmonic genus can be told by the quarter-flat, three-quarter-flat, and double-flat alteration signs. The entire MPS reveals a *centripetal* tendency, where the greatest variability in pitch occurs next to the central Mesa (A3). The entire system is characterized by extreme *gravitational diversity*: altogether there are 6 types of distribution of gravity within a modal subset.

The MPS mode loses some of the sovereignty of a stand-alone mode: it is no more a container of characteristic intonations popular within a particular kind of music. The MPS mode has to share its degrees with other modes, evident when one mode immediately follows another mode (as in verse/chorus or song-dance)^16^. Perceptual “sameness” of degrees encourages the performer to strip off the MPS mode of those intonations whose expressive tuning violates the tuning of a sister-mode. Eventually, all modes within a family turn out being “averaged.” This can be seen in comparing Figure 3A from Part-1 to Figure 4C here: the hierarchy of stable degrees is the same, but the hierarchy of unstable degrees flattens in the MPS. There are only two gradations here: VII vs. II-IV-VI. In the folk heptatonic mode there were 4 gradations: least unstable IV, more unstable II, yet more subordinate VI, and leading VII this hierarchy ends up reduced by one level by the demand to preserve the pitch values for all the member degrees across all sister modes^17^.

The earliest reliable sample of Ancient composition is Epitaph of Seikilos. Sustained in diatonic mode, it was likely composed in observation of the music theory of the day (Mathiesen, 1999, p. 150), exemplary of MPS melody.

6. Audio: Epitaph of Seikilos, 1st century AD. Ancient Greek Phrygian diatonic tonos (coincides with modern Dorian E). Unstable degrees are somewhat averaged and moderated in their attraction to stable degrees, as compared to the stand-alone folk heptatonic mode in the example below. http://bit.ly/1Ch6ECE

Its most obvious trait is non-formulaic structure. Diversity of Epitaph's intonations outweighs the only pattern present in the entire composition (line-endings 3-4). Abundance of directional shifts and over-degree-skips obscures anchoring.

7. Audio: Thracian Air. Modern Dorian E (Hypodorian) mode. Well-marked tonicity makes resolution of the unstable degrees clear. The phrase sampled in this example is continuously repeated throughout the recording of the entire song. http://chirb.it/MG6Iw2

Nearly all survived Ancient Greek music features improvising style, even choirs. In this, they contrast the overall formulaic aptitude prevalent in European folklore (Zemtsovsky, 1987), suggesting opposition of folk and palace/temple music in Antiquity (see Appendix 1 in Supplementary Material).

If a *beauty-in-averageness effect* (Winkielman et al., 2006) can make a folk tune, averaged by modifications of multiple musicians, appear attractive and “natural,” an authored tune can make an “artificial” and idiosyncratic impression. Likeability here is traded for originality. Certainly, the authored tune can also be orally disseminated. But practice of performance under supervision of a musical administrator in Ancient Mesopotamia (Michalowski, 2006) was not likely to provide enough freedom in variation for averaging effect to occur. Administrated music tends to turn into hard “rule.” And later Hellenic civilization made exact public reproduction of someone else's composition socially unprestigious.

Individual practice of following melodic rules sets in place hierarchic processing of pitch, where the “invented” contours are filled up with the standardized intervallic detail-establishing the modern tonal standard of pitch pattern processing (Stewart et al., 2008). Just like performers, listeners here need to know the tonal schemata before they face a particular music work. Melody processing in such music is driven by instant *automatic* response to the tonal progression conceived or auditioned—relating it to long-term memory (Brattico et al., 2006) for pitch-set class (PSC) and interval-set class (ISC). Pre-attentive response indicates that modal rules are optimized and hard-wired in the MPS, as opposed to earlier modal systems:

- In pre-MPS heptatony the standardized contours were filled with idiosyncratic intervallic detail;
- In MPS heptatony the idiosyncratic contours are filled with standardized intervallic detail.

Emergence of the concept of “key” in music theory reflects this advance. The term “key” is often used synonymously with “tonality,” which is inaccurate. Ancient Greek music used keys that did not constitute tonality. The modern notion of key implies presence of a fixed PCS subordinated to a single tone. In practice, *key* was brought to life by the necessity to retune string instruments before playing in a different mode (Kholopov, 2006, p. 73). Tuning always proceeds from a certain tone to which other tones are adjusted. Hence, one pitch is singled out from a PS and the entire PS is inferred from it. This is not exactly about stability, but rather *priority materialized in audiation*. Such “key” is *not* found in folk cultures (Kvitka, 1973, p. 25).

Tuning practice encourages a single key to incorporate multiple modes—to minimize retuning. This is where the complex interplay between “key” and “mode” begins (Solomon, 2000, p. 75). Convenience of immediate switching from one popular mode to another overweighs the importance of key's integrity, legalizing certain alterations^18^. These alterations become “modal”—characterizing a certain mode. Their very presence testifies to the presence of key.

Over time, key earned its own ground, different from mode: Greeks distinguished between “modulation according to the scale,” and “modulation according to the key” (Hagel, 2009, p. 5). Their keys could be transposed like our keys, and were associated with “key signatures” (West, 1992, p. 179)—but they neither incorporated the notion of tonic triad, major/minor inclinations, nor implied vertical harmonic functionality (185).

Rigidity of key rules secured the processing speed, enabling the handling of larger stocks of data. Shulgi's introduction of “rigid music” set the foundation for the evolution of complexity in Western music, allowing music structures to convey more information about the perceptual reality as perceived by the creator of music.

An MPS mode becomes a member in the assortment of modes, whose knowledge is obligatory for a professional musician. He is supposed to choose the right mode appropriate to the occasion. Specialization of modes is promoted by ensemble performance and genre application^19^, and relies on professionalization^20^. Beliayev (1990, p. 296) underlines that professionalization of tradition necessarily involves development of multimodality. By the eighteenth century BC, there was already an internationally recognized system of accreditation of musicians—in courts and temples of Near East—with clearly defined ranks, and frequent relocation and integration of musicians through conquest and gift-exchange (Franklin, 2007). Already a 2800 B.C. relief shows two lyres playing together (Krispijn, 2010). In order for harpists to stay “in tune” with one another's strings, they had to share the same understanding of a mode/key. Middle Assyrian tablet VAT 10101 (West, 1994, p. 170) presents a census of Akkadian love-songs, classified by modes (tunings)^21^.

It took about 2000 years for the heptatonic MPS tradition to settle before the Greeks established the status quo for the entire region. Already for Ancient Romans there was no alternative to Greek music: there are almost no traces left of original Etruscan music (Powley, 1996)—it was overwhelmed by Greek influence (Landels, 2002, p. 182). Reliance on the ultimate music-making scale became organic part of this influence (Winnington-Ingram, 2015, p. 50). This was a direct outcome of conceptualizing the ISC, and teaching the ear to center on different tones of the same PS.

The circle of 5ths was that instrument which equalized the MPS. Ernest Clements (1935) reserved the term “quintal” to refer to what I call MPS scales—as opposed to folk heptatony. The diatonic Mediterranean MPS is cross-culturally implemented in the *system of 8 modes*, produced by modal transposition from each of the degrees of the principal heptatonic mode, including its intervallic reproduction an octave higher, with the tonic placed in another tetrachord. Werner (1948) investigated such octoechos systems, tracking them to the beginning of the 1st millennia BC Mesopotamia. He concluded that division in 8 modes was a melodic concomitant of the mathematically realized harmonic octave affinity assigning a dedicated mode to every degree within an octave, which originated not in musical but in cosmological and calendaric numerology.

Pentatonic mode passed through a similar transformation in constructing the *pentatonic system* (Cook, 1995)^22^. The origin of the idea of pentatonic MPS must date back to the ninth century BC, when the tones of PCS obtained their standard pitch names (Kuttner, 1965). Around the fifth century music theory had in place the principles for reproduction of the “legitimate” pentatonic modes across the available tonal space. The similarity of Chinese hexagon circle of 5ths with Chaldean music theory is striking—most likely determined by their astronomic correspondences (Daniélou, 1995, p. 37). The Yang-Yin dialectics defined the anchor points on odd degrees (Yang) vs. “unstable” even degrees (Yin)^23^. Sixty pitches were standardized by the use of precisely manufactured bells—used as reference tones for tuning instruments (Falkenhausen, 1992). Music theory devised a nominal 12-tone system by inferring the whole-tone scale and then dividing the whole tones in halves^24^. Its main purpose was to absolutize the pitch values for use in all possible transpositions of the legitimate pentatonic modes (Bagley, 2005)—in effect, a *mega-pentatonic* PS.

The idea of *concert pitch standard* is a logical consequence of cultivation of MPS: the idea of maintaining the sameness of tones across the sister modes suggests adoption of some standard of reference. Especially in ensemble performance and ecclesiastic application, a particular pitch could be assigned to a specific supernatural power justifying its standardization. It is unlikely that the Chinese MPS was the only absolute one. Greeks tuned their lyres to aulos, and designed their notation around fixed names of pitches (West, 1992, p. 273). Greek citharas incorporated tuned resonators which would ensure fixed pitches (Hagel, 2009, p. 69). Amazing is that modern pitch standard (A4) closely corresponds to the reference tones that defined Ancient Greek MPS (A2-A4). It would be extremely interesting to find out if the phenomenon of perfect ear existed in antiquity, or if it is a byproduct of modern tonality (Steblin, 1987).

Pan-harmonization of music system can be seen as means of resolution of cognitive conflict. It is not by chance that civilizations of Mesopotamia and Egypt, China, and India, all embraced cosmogonic music theory about the same time they developed script systems. Rise of literacy^25^ and analytical method of thinking^26^ were promoting awareness of complexity, contradictions, and imperfection of the state of things in the cultural environment. Analytic approach to text paved the road for rationalization of notions inherited from the traditional folk culture (Civil, 1994). Mesopotamian education system trained to grasp and put in use the meaning of texts (Michalowski, 2012).

Conflict of interests was a common motive in Sumerian and Akkadian literature, with plenty of vivid illustrations of invective and reproaching rhetoric (Foster, 1996, p. 220). In Akkadian literature, first person's speech often emphasized the state of cognitive dissonance. A very popular epistolary genre often presented complaints of unfair treatment (Vulliet, 2011). Even more conflicting were the genres of diatribe, where two persons competed in the verbal attack of each other, disputing before some deity (Hallo, 2010, p. 120). Verbal skills played a deciding role in forging and polishing counter-distinctive manner of thought, thereby amplifying awareness of cognitive dissonance. At its pinnacle was the rise of judicial rhetoric, which exposed conflicts of interest between different individuals, and rewarded better argumentation (Hallo, 2010, p. 126). Trials were held in public, and declaration of each of the parties was pivotal in influencing the court's weighing of the conflicting statements (Wilcke, 2007, p. 44).

Babylonian culture saw a marked increase in individualism (Foster, 2011), confrontation, and disorder, earning the nickname “Dark Age” that hit the entire Mediterranean region around the twelfth century BC (Drews, 1995). The assumption of the state ideology that serving king's interests serves everyone's interests turned out to fail to motivate the subjects to defend the state against external intrusions or internal plots. Cognitive dissonance should be put on the list of contributing factors in the inability of the Bronze Age palatial cultures to sustain resilience toward environmental and international stresses. The “barbaric” tribes, with more homogenous social structure and “cognitively consonant” music would have had an advantage over Mesopotamian civilized societies, subdivided and weakened by contradictory interests of their social groups and their musics.

Codification of the MPS system should be viewed within this context of growing cognitive dissonance. Rational harmonization of the entire compass of all available music tones was not a deliberate political move in reaction to social pressures, but an elemental biological response. Inspired by correlative cosmologies, mathematically-based theories of music harmony catered to neurobiological need of the brain to reduce informational stress by employing a new strategy of organizing data and establishing ways for synthesis of new quality out of it (Farmer et al., 2000).

## Non-octave hypermode

Ancient Greek Systema Metabolon set the theoretical foundation for yet another distinct method of tonal organization—found in Medieval Western Europe, Byzantium, Russia, Armenia, Georgia, Azerbaijan, and Bulgaria. The title *hypermode* (Pashinian, 1973) captures its principle of stitching multiple tetrachords or trichords into a single system, spanning well over an octave. The tonal integrity is achieved by taking small elementary subsets, deficient to determine the makeup of the entire melody, and uniformly conjoining them according to the “chain principle” (Sachs, 1960): addition of a twin-subset whenever melody runs over the margin of a subset. The expanded set is treated compositionally as a single entity—especially pronounced in a polyphonic setting.

At the heart of hypermode is the fixed registral contrast between marginal tetrachords/trichords. The PCs of each subset are permanently mounted in the overall ambitus, disallowing alterations. This music makes a fairly diatonic impression between adjacent subsets, while evoking “friction” between the remote ones, expressed in “false relation” of the octave-inequivalent tones. Equivalence of 4th (or 5th) binds the mode.

8. Audio: Ne oryol li s lebedem kupalisia, lyrical Cossack song, Southern Russia. B-C#-D-E-F#-G-A-B-C, false relation C#-C induces subtle increase in tension in the high register—in contrast to relaxation at low register. http://bit.ly/1JzPqDA

The melody sustained in hypermode exhibits a peculiar “elastic” effect: as long as the phrases stay in the same registral position, they appear “casual,” but ascending induces tension, whereas descending—relaxation. The entire melody contracts/expands like an elastic band through cycles of tension/relaxation. The greater the amount of subsets, the greater the “elasticity.”

9. Audio: Mussorgsky—The Great Gate of Kiev, the 2nd theme. The 12-tone hypermode: G#-A#-B-C#-D#-E-F#-G#-A-B-C#-D, with 2 false relations D#-D and A#-A within 4 trichord subsets. http://chirb.it/2dw7f3

In a few equintervallic diatonic subsets, elasticity is minimal: i.e., Byzantine hexáechos^28^ (Figure 3A) is very close to diatonic MPS.

10. Audio: The Little Entrance “Come, Let Us Worship” [Priidite, poklonimsia], 2-part Znamennyi chant, based on Byzantine hypermodal system. Minimal tonal tension from ascending motion through 3 equintervallic trichords. http://bit.ly/1IO8mlX

Larger size subsets, such as tetrachordal and pentachordal, common in Georgian traditional music, increase functionality of PCs, inducing substantially greater instability—which is handled by more elaborate hierarchic organization.

11. Audio: Kakhuri nana. Lullaby. Georgian tetrachordal hypermode (Figure 3B) with the characteristic diminished octave G#/G (Gogotishvili, 2010). The unstable functionality prevails over the stable one. http://chirb.it/yqEtCD

Non-octave hypermodes presented a window for expression for the strictly controlled amounts of tension (see Appendix 2 in Supplementary Material for details) that was compartmentalized in different registers. The resulting opposition to “natural” (for speech and animal vocal communication) association of high register with submissiveness while of low register with aggression (Ohala, 2006), marks the contribution of hypermode to the establishment of specialized musical tonal semantics—in contrast to verbal tonal semantics.

Yet another historic landmark was divergence of hypermode from chromatic system by providing a diatonic-based alternative to the chromatic expandability by alteration/modulation (see below). A noticeable affiliation of hypermodal organization with the Christean plainchant, which subsequently shaped the folk music of many Eastern Orthodox nations and ethnicities, expressed rejection of the cultural heritage of the Greco-Roman philosophy of music and an attempt to restore the older Sumero-Babylonian cosmology on new theological ground (see Appendix 3).

## Alteration and modulation

Unlike the hypermode, the diatonic MPS did not restrict degrees to sustain their pitch values throughout the music work. The need to temporarily increase tension was handled by alteration and modulation. The term “alteration” refers to raising or lowering of a degree in a PCS, involving modification of the IS. When this happens, listeners familiar with this PCS become surprised by its deviation from the norm. The impulse to restore familiar IS is what is responsible for momentary increase in tension associated with the alteration, when the listener experiences intense expectation for it to comply to the norm (Margulis, 2005).

Alteration is a form of *cognitive dissonance*. Formulation of Systema Metabolon (“the modulating system”) concurred with the formation of the discipline of dialectics in Ancient Greece (Losev, 2000, pp. 601–35), and with the growth of public interest in it (i.e., rhetorics, sophisms; Laertius, 1958, p. 137). As people realized the limitations of words in reference to real objects, the dialectic method of defining opposites began to make an imprint at first on the manner of conducting scholarly research and legal matters, then on the discipline of rhetoric in general, and finally on tonal organization. The primary function of music to harmonize was understood through opposition of tension and relaxation, “united by disunion” (Plato, 2012, p. 13).

Neither unfixed ekmelic degrees, nor expressively tuned multitonal degrees of pre-MPS musics involved cognitive dissonance. Rather, they constituted exaggeration of intonation in pitch—what Cazden (1971) termed “modal inflection.” The principal difference is that “*chromatic*” alteration implies production of *two* colors and cognitive conflict, whereas “inflection” implies saturating a *single* color and no cognitive conflict.

12. Audio: Shelkovoya travushka, Nekrasov Cossacks. The IV degree here exists in three flavors (normal, sharpened, and flattened)—marking the opening of each strophe with a tonal “blot (see the frequency analysis in the Demonstration-4 in Part-I).” http://chirb.it/sJ8dCG

Modal inflections are modally normative: justified by the permanence of melodic rule.

13. Audio: Alilo, Georgian ritual Christmas song. The melodic rule: in the middle voice, every time B goes to C#, it sharpens, but every time it descends to A# in the opening of every strophe—it stays natural. http://chirb.it/8r2mhm

Alteration does not possess such permanence and logic. By its nature, it is *accidental*. Alteration splits the normative degree into few versions within the same composition, calling for further “resolution”: two versions cannot both be “right,” one ought to be “wrong,” and therefore “corrected^29^.”

Alteration is relatively rare in oral traditions^30^ reserved to technically advanced professional music with fully fledged music theory^31^.

14. Audio: Maddoh, Pamir. Improvisation on a ghazal by Hafiz. The stanza starts with the altered degree C#, creating a dissonance in relation to the accompaniment—and then resolves into B, restoring the initial non-altered mode: E-F#-G#-A-A#-B-C-D#. http://chirb.it/mrvGbd

Alteration should not be mistaken for progression of *natural* degrees in folk “microtonal” modes, where seemingly “chromatic” degrees are normative (Petrovi, 1994). Such modes can contain their own *micro-alterations*.

15. Audio: Falak-I Badakshani, Pamir. Microtonal alterations of four “natural chromatic” degrees within the ambitus of F#4-A4, providing extra tension for a genre of funeral lamentation (Levin, 2007). http://goo.gl/SnUhO1

See Presentation-1: Alterations/Micro-alterations.

“Modulation”— transition from one musical mode to another without a break—differs from alteration by violating *gravity* rather than *PCS*. Modulation has been theorized exclusively within the framework of Western music. Similar devices are known in other advanced music systems (Indian, Arabic, Chinese)—although without receiving much attention in their music theory. Modulation in folk music presents a novel and controversial object of study.

The most common form of gravitational shift in folk music is intra-modal mutability.

16. Audio: Li Weri, a Senufo funeral, Côte d'Ivoire. Intra-modal mutability in pentatonic mode from C to Eb and finally to F. http://bit.ly/1hnTnF6

Zemtsovsky (1998) calls this “pentatonic enharmonism”: ability of PCs to get included in different trichords, where the same PC would act as an anchor in one trichord, whereas remain unstable in another trichord. Similar “enharmonism” is possible in hemitonic modes usually involving membership of the same PC in two different tetrachords.

17. Audio: Nozanin-Shod-I Uforash, call-and-response sozonda (wedding), Bukhara. Intra-modulation a step up, from Eb to F, in a heptatonic mode. http://chirb.it/zD8eLk

Mutability of multitonal mode (see Part-1) restricts intra-modulation to only 2-3 anchor-tones, making gravitational shifts predictable and regular.

18. Audio: Ocarina solo, Bulgaria. Each sentence (provided sample) starts in A, in major inclination, but ends in F#, in minor inclination. Such A/F# alternation shapes the form of the entire composition, only by the end of it committing to a prolonged F#. http://bit.ly/1Ga3LYa

The MPS *generalizes* diatonic “enharmonism”: if folk mutability shifted gravity for a *single tone*, MPS modulation shifts the *entire set*—rebuilding it from *any* of the degrees.

Helladic music probably featured simple diatonic modulations (Franklin, 2002). Its original pitch set constituted an Olympic trichord E-F-A (West, 1992, 164). As time progressed, the set size grew—ultimately reaching an octave species, allowing for inter-tetrachordal enharmonism. Despite their size, all MPSs are treated in the same way: music users remember the normative sets, and upon detecting modulation, hypothesize a new set from what they already know (Raman and Dowling, 2012).

The *entire PSs* are alternated—even if, technically speaking, the PS degrees retain the same pitch values (as C-Ionian/A-Aeolian). In reality, their pitches are not exactly retained, since each PCS imposes its own expressive tuning: certain degrees are slightly sharpened or flattened, depending on their function in the PCS (Sundberg et al., 1995). The same tone B will be intoned sharper in Ionian C, and flatter in Aeolian A (Tchesnokov, 1961, p. 58). Although, this adjustment is not as drastic as a single tone mutation in a folk multitonal mode, it nevertheless does occur^32^. In the polymodal system, the music user remembers modes by their IS, including their characteristic expressive tuning (Brattico et al., 2006). Absence of expressive tuning is perceived as faulty performance (Sundberg, 1982). Every time music modulates from mode to mode, the melodic ISC switches, causing reassignment of expressive tuning values—all at once, as in switching from one tuning table to another. This is what the phenomenon of “harmonic modulation” practically entails.

Listeners take expressive tuning as a prompt in detecting the most stable (immutable tuning) and unstable (most mutable) degrees. They estimate modulation in terms of gradations in tension determined by the *intervallic value of the modulation*— the interval between the old and new tonics. Thus, modulations to subdominant (C-F) are perceived “tenser” than modulations to dominant (C-G) (Korsakova-Kreyn and Dowling, 2012). It seems that the listener's affective response to modulation is determined by the way in which the entire PS and IS of the “arrival” mode appears to the listener in relation to the “departure” mode. Thus, modulation from minor dominant to minor tonic appears different than modulation from minor subdominant to minor tonic. Transition from one PS/IS to another is processed probably as a single percept akin to the standard progression of chords^33^. The emotional reaction to modulation proves to be one of the most exciting stimuli in music listening experience (Korsakova-Kreyn and Dowling, 2014). We shall see later how this emotionality is important for the emergence of chromatic system.

Modulation usually involves alteration—their combination pioneered in Ancient Greece.

19. Audio: Mesomedes—Hymn to the Muse, second century AD, brief modulation from Lydian to chromatic Hypolydian mode by the end of the hymn (Hagel, 2009, p. 287). http://bit.ly/1Rpp5l2

Hellenic listeners identified melodies by intervallic differences (Lippman, 1964, p. 160): which involved IS, IC, ICS, and ISC^34^. *Interval-tracking* habit was responsible for non-formulaic composition as opposed to *contour-tracking* habit of earlier folk-musicians.

Both, Babylonian and Assyrian songs fit a single song into a single mode (Franklin, 2013, p. 218). In Classical Greek music, a song often contained a nexus of tetrachords, each bearing its own modal organization (West, 1992, p. 226).

Professionalized folk cultures can come close to what might appear as a chromatic modulation either by emulating MPS music or forming *composite* mode-a compound of 2 or more stand-alone modes (Belaiev, 1963).

20. Audio: Duma about Marussia of Bohuslav, Ukraine. Modulation from E to B that appears to be influenced by the Western classical modulation from tonic to dominant. http://bit.ly/1Eg6y2B
21. Audio: Toshto Marii Kushtymo Sem, Marian dance. Here, Pentatony that characterizes the music of Volga Finns is enriched by the *composite* mode C-D-Eb-E-F-G-Ab-A-C, which was most probably generated by adding together the C-D-E-G-A and incomplete C-Eb-F-G-Bb (without Bb) pentatonic modes. http://chirb.it/MdNz3B

When folk musicians learn a diatonic PCS, they begin to transpose it by degree. Eventually, they come to connect two tunes, each associated with its own mode, into a medley. Then, one mode becomes transposed so that it would start on the same I degree as another. As the performer gets used to this juxtaposition, he can combine intonations from both modes within the same song. Even pentatonic modes acquire quasi-chromaticism in this way. Thus, two pentatonic modes built from the same tone (i.e., C-D-E-G-A and C-Eb-F-G-Bb) produce quasi-altered III degree (C-D-Eb-E-F-G-A-Bb). The complete combination of all pentatonic modes results in a 9-tone *composite* mode C-D-Eb-E-F-G-Ab-A-Bb^35^.

However, “chromatic” tones in composite modes are never used in scalar fashion (Belaiev, 1963). Even when a folk musical instrument includes the entire chromatic scale, as in Chinese shen or pipa (Riemann, 1899, p. 5), it hardly ever plays chromatic successions. Tunes remain pentatonic or diatonic, while the “chromatic” tones are reserved solely for passing from one mode to another (von Hornbostel, 1975, p. 41).

## Chromatic polymodal system

The more frequent is the alteration, the more likely it is for it to cause habituation, lose its affinity with cognitive dissonance and acquire more “consonant” status. This is what must have happened in the Hellenic culture. According to Ancient Greek sources, altered tone's function was to “shade” the diatonic degrees: notable was the reference to “sweetness” of chromatic alterations (Hagel, 2009, p. 154)^36^. Pleasantness of alteration was responsible for the quick popularization of lute in Greece from the fourth century BC: unlike lyre, lute allowed to comfortably produce chromaticism (Higgins and Winnington-Ingram, 1965). Fashion for alterations could have “normalization effect” on chromaticism, so that its cultivation would have “domesticated” the cognitively dissonant aspect of it (Katsanevaki, 2011).

22. Audio: First Delphic Hymn to Apollo, second century BC. Essentially, this composition presents spare use of chromatic alterations shading of the Phrygian tetrachord (West, 1992, p. 288). http://bit.ly/1Plytkj
23. Audio: Katolophyromai fragment from Orestes by Euripides, from papyrus, 3rd century BC. Most of the melodic content of this lamentation in chromatic Lydian mode is made of altered degrees. http://bit.ly/1g3VzB5

Chromatic alteration became affiliated with aesthetic emotion after the practice of connecting certain modes with certain affects was established through the temple culture of Sumerian and Egyptian cults, some time around the 2nd millennium BC (Farmer, 1965) (see Appendix 3 in Supplementary Material).

Earlier agricultural civilizations heavily depended on the calendar, which boosted the development of astronomy and math, but carried no mystic and esoteric implications to entitle numerology to a governing status delegated to the elite (Frolov, 1992, p. 152). Babylonian music theory was first to link the arithmetic definitions of musical tones to cosmology. Cosmology empowered music with the status of natural law, equating music's influence with the sun or the moon. Just as excess or shortage of sunlight can cause problems, so presence or absence of certain modal qualities in music was believed to be beneficial or hazardous for a person. This doctrine is known as “ethos” and existed in numerous Ancient civilizations (Kaufmann, 1976; Rowell, 1981; Deva, 1995; Katz, 1996; Thrasher, 2008).

In the 6th century BC, Sakadas of Argos started combining different ethea in a single composition by employing intra-modulations between different verses of his song. Then, Aristoxenus' Perfect System rationalized the means for the composer to generate his *individual* map of tonal tension suitable for a particular composition.

24. Audio: Second Delphic Hymn, second century BC. The music is built on the Lydian tetrachord, alternating between Hypolydian and chromatic Lydian modes—which seems to be reserved as means of a peculiar compositional arrangement, unlike the modal stereotypicity of folk music. http://bit.ly/1LCtjkc

Rising standard of authorship incorporated *modal creativity*. Greek civilization championed cultivation of *melopoeia*, art of composing music, put forth by Plato (Kholopov, 2006, p. 74). From the fifth century BC until the Dark Ages, authorship guided expression in the arts. Distinguished authors' names were perpetuated, encouraging other artists to either follow their steps or to compete with them. Growing popularity of chromatic style in the fifth century Athens reflected the antithesis of diatonic conventionality vs. chromatic originality. For the next half-millennium, enharmonic and chromatic genera made the diatonic genus look too predictable and unimpressive (Franklin, 2002).

Chromatic modulations were restricted to melodic junctions between the adjacent tetrachords: alteration could only follow the consonant “bounding” tones at the tetrachord's end (Hagel, 2009, p. 10). Thereby, diatonic system provided the skeleton for all modulations and alterations—very much like in a modern key. However, not all musicians followed the rules (Franklin, 2002).

Crexus, Timotheus, and Philoxenus were condemned for increasing the number of strings on the lyre, and excessive elaboration—blamed for using “polyharmonia” to appeal to the mob's ideas of plurality and liberation (LeVen, 2014, p. 81). This accusation should be understood in the context of dithyramb contests and theatrical plays becoming exceedingly popular to the extent of introduction of entrance fees for the first time in Greek history (Csapo, 2000). Theater musicians made lavish profit and enjoyed enormous popularity—this, together with the growing market (18 theatrical festivals per year, fourth century BC) unleashed fierce economic competition (Csapo, 2011). New Music was definitely based on the direct approval/disapproval of live audiences. The immediate reason for the split of public opinion, and voices for its condemnation was its break of conventional ties between mode and genre, and its inter-strophic modulation—which could be rather abrupt, even a semitone apart (Hagel, 2009, p. 44).

25. Audio: Lamentation from Iphigenia Aulidensis by Euripides, third century BC. Modulation from Hyperaeolian to Hyperphrygian mode by common tone. http://bit.ly/1DtOwq2

Chromatic music represented new philosophy of consumerism of aesthetic emotions—in opposition to Platonic philosophy that reserved diatonic music for propaganda of “right” emotions (Stamou, 2002). Chromatic music grew out of older enharmonic music that was cultivated in *Dyonisiac* dithyramb, and became related to theater and symposium (drinking parties), both of which involved aesthetic appreciation. Chromaticism as “sweetening” of intervals by tonal shading served to evoke states ranging from “pleasant” to “lugubrious” (Franklin, 2005)—essentially, aesthetic emotions^37^.

Athenian chromaticism replaced cosmogonic consonance admiration with admiration for realistic impersonation of humanistic character traits, interwoven into dramatic development. Aristoxenus' chromatic system instrumented this change by rejecting older Pythagorean numerology as “dogmatic,” and basing a new music theory on psychoacoustic principles put to service of the composer (Barker, 1978).

Another important issue was the topological reference frame: Babylonian/Pythagorean diatonic theory was all *arithmetic*, defined by *prescriptive* numerical proportions, whereas Aristoxenian chromatic theory was *geometric*—*descriptive* of actual distances on monochord's strings. Remarkable is the commonality of Aristoxenian and Euclidian approaches to the infinitely smallest magnitude, setting a conceptual and a terminological correspondence between musical and physical spaces (Barbera, 1977). Chromatic tetrachords reflected the contemporary advance in the irrational numbers, presenting breakthrough from Pythagorean ratios (Scriba, 2015, p. 44). Babylonian mathematics had strong arithmetic-algebraic character: tables and lists of reciprocals and roots provided the “right” answer for a particular use, where “the geometrical form of the problem was usually only a way of presenting an algebraic question” (Struik, 1987, p. 28). In contrast, Greek geometry sought methods for inferring the relations between objects based on empirical proof.

Moreover, Euclid introduced a strong *personalized* aspect in such calculations, where angles and distances were estimated from the viewpoint of a particular spectator (and not “in general”), resulting in discrepancies between “optic” and “perspectival” evaluations (Andersen, 2008, p. 725). Unlike Babylonian geometry, Euclidian geometry was influenced by scenography (728), acquiring strong spatial connotations (considering geometric lines as representations of what can be actually seen around)—in contrast to Babylonian “aprioristic” line of thinking (providing ready numbers for a particular application).

Chromatic music was a tonal system *engineered* to present *emotional theater*: to convey detailed emotional information prompted by the text and/or dramatic action. Chromatic MPS broke away from a diatonic MPS by becoming a storage of modulation/alteration possibilities for the composer. To minimize the inconvenience of retuning the lyre, which remained a reference instrument for theory, musicians had to find as many common tones between different modes as possible. And seven principal modes, when built from the open string E, mark the E-A-B core of immutable tones, thereby forming the axis for categorization and hierarchical organization (Gombosi, 1951). Of E-A-B, central A3 seemed to execute the function of the ultimate tonic (West, 1992, p. 219).

Just as ekmelic and mesotonal modes, chromatic modes were crystallized by the *permanence* of tuning: the least frequently retuned tones acquired the status of stability, while the most alterable tones ended up at the bottom of the tonal hierarchy. The *synékheia* (continuity) law postulated that all chromatic modifications to be derived from diatonic MPS for better melodic coherence (Franklin, 2005)—Aristoxenus was clear on using the entire MPS as a reference for chromatic alterations (Hagel, 2009, p. 44).

The MPS structure in Figure 4 represents the chromatic/enharmonic key of A (Strunk and Treitler, 1998, p. 37), expanded over all the available sonic space—what was called Systema Ametabolon (West, 1992, p. 223). Aristoxenus described 13 chromatic “keys” which altogether regulated organization of chromatic/enharmonic genera, built from each of the 12 semitones between Hypodorian F2 and Hyperphrygian F3 (Hagel, 2009, p. 48). The description of the chromatic system might sound extremely complex, but in practice, the overall number of PSCs in the MPS was not exorbitant^38^. There was little distinction between the chromatic and enharmonic genera^39^. Greek notation did not distinguish between them at all (West, 1992, p. 255), and the performance practice left the exact choice to the discretion of the performer. In reality, musicians had to deal with no more than 14 different types of tetrachords: 2 types of each of the 7 principal keys.

The entire Systema Ametabolon clearly stresses the A/E gravity, with tonic/dominant functionality. The epicenter of chromatic mutability falls at the upper middle of the MPS (Figure 4). This is the register where melodies show the greatest modal complexity. The peculiarity of Greek system is that all alterations are *descending*^41^. The descending functionality of Ancient Greek music probably originated from the Archaic trichord E-F-A (West, 1981), with its characteristic “directing” semitone placed at the bottom. This trichord became a melodic frame, where extra tones could be placed in between E and A, forming two oldest heptatonic genera, diatonic and enharmonic, circa seventh century BC, credited to Olympus (Barker, 2007, p. 99). Chromatic genus evolved later, as a simplification of enharmonic genus, and gained in popularity—up until AD: surviving musical fragments from the Roman period are almost wholly diatonic, and both, Gaudentius and Macrobius reported that chromatic and enharmonic genera were obsolete by fifth century AD (West, 1992, p. 165).

Chromatic music was ousted in the West, but not in the East of Roman Empire. Greek chromatic MPS impacted all the territories between Greece and India—conquered by Alexander during the heydays of chromatic music. “Gapped” structure with chromatic/enharmonic *pyknon* (a pinch of three close pitches) penetrated local folk cultures and created a special intervallic class—what Kholopov (1988, p. 38) named “*hemiolic*” (“hemiolia”—the 1½:1 ratio). Hemiolic mode differs from diatonic by its chromaticism: recoloration (chroma) of ICs due to their inequality—most prominent in microtonal varieties of hemiolic modes, i.e., maqam Hijaz-Kar-Kurdi C-Db-E¾b-F-G-A¾b-B¾b-C (Racy, 2004, p. 108, see Appendix 4 in Supplementary Material).

26. Audio: B'utho, Syrian Orthodox chant, Tminoyo mode. Chromatic alterations with microtonal inflections: A-Bb-Cb-D(Db)-Eb-F-Gb(G)-Ab(A)-Bb-Cb-Db (Lundberg, 1997). http://chirb.it/cvyFOy

See Presentation-2: Post-Hellenic chromaticism.

Hemiolic modes are decidedly *non-diatonic*: their tones *cannot* be positioned in a circle of perfect 5ths. Gapped tones represent discrete—and not altered—degrees of the mode: the entire music work might be based on the stationary gapped tones, without any modification. Such mode differs from pentatony by *contrast* of gap and semitone, where expansion of one causes shrinking of another, inducing tonal tension. Instability of both gapped tones is responsible for their flexibility in expressive tuning, which enables them to come closer to a target stable tone, thereby exaggerating tension and relaxation (Marcus, 1993). The emotional expression of hemiolic gaps also opposes that of pentatonic trichords: related to heightened pleasure and even ecstasy (ibid.), unlike the gapped tones in pentatony, which constitute a commonplace rather than a sign of elation there.

Manuel (1989) reserves a special term for hemiolic/ music amalgamated by Arabic, Turkish, Greek, Jewish, Gypsy, Romanian, and Andalusian traditions - “*the Mediterranean tonality*”—first documented in the early nineteenth century manuscripts of linear notation. Initially strong, microtonal enharmonic component of such music eventually became “retuned” into the Western classical music diatonic tonal space, as the performance practice adopted accompaniment with chords. The traditional monodic implementation, on the other hand, has preserved the microtonal adjustments, especially those of the hemiolic gap, supporting the tonal organization that is distinctly different from Western tonality (Marcus, 1993).

The Mediterranean implementation of chromatic recoloring shares affective aspirations with Western *musica ficta* (Westrup, 1954), but follows a different modal order.

- Western chromaticism was accidental in nature, and followed the *trichordal* scheme, where the chromatic degree would be jammed between two diatonic degrees.
- Mediterranean chromaticism was regular, modally driven by melodic inflections, following the *tetrachordal* scheme, where two chromatic degrees would be encapsulated between two diatonic degrees.

This difference determined polarly opposite paths of their development. Western chromaticism, from the twelfth century on, supported emergence of polyphony as a standard of composition—by becoming a tool of regulating the *vertical* harmony by means of triadic “over-a-degree” vertical relations. Mediterranean chromaticism fueled melodic complexity, serving as the primary expressive means for the composer in organizing *horizontal* harmony by means of intra-tetrachordal alterations and modulations. This important distinction has led to completely different spatial connotations for Western tonal key and Mediterranean tonality. Western spatio-tonal design went towards incremental geometric projection of ever growing complexity, while Eastern Mediterranean focused on ornamental patterning the nexus of small size modules (al Faruqi, 1985). This contrast seems to reflect more fundamental opposition of philosophies, where Western Christian and Eastern Islamic cultures appear to form the core for the divergence between Western and Mediterranean tonalities (see Appendix-4).

## Tonality and perspective as models of representational organization

From a perceptual angle, the concept of chromatic tonal key incorporates uniformity of distribution of stability/instability within the PCS, which involves 5 hierarchical ranks (Lerdahl, 2009):

1. tonic (function of gravitational center);
2. dominant, subordinated to tonic, yet providing an anchor alternative to it (opposing function);
3. mediant subordinated to tonic, while comprising the tonic triad together with dominant (complimentary function);
4. 4 coordinated non-tonic diatonic degrees subordinated to tonic, dominant and mediant (auxiliary function);
5. chromatic degrees, each subordinated to a neighboring diatonic tone (function of a leading tone).

Such organization evolved from the diatonic MPS through standardization of intervallic relations by means of counterpoint techniques which every composer was expected to know. Ability to hear equivalent concords (triads) between multiple parts, and recognize them as a single typological percept was set in place during the Renaissance (Nutting, 1974). Merging of vertical intervallic relations into a triad sonority, and its categorization by the bass together supported emergence of major/minor tonality in place of the old 8-mode MPS (Parncutt, 2011).

Subsequently, psychological representations of tonality in terms of chords, for Western listeners, became as real as hearing the tones themselves (Vuvan and Schmuckler, 2011). Tones, intervals, and chords are processed through imagery representation, and the representation of chords is derived from the representation of tones (Hubbard and Stoeckig, 1988). Therefore, chords should be regarded as common chunks of pitches, remembered by music users to facilitate tonal navigation across the music work. Standardization of chords is what separates Western polyphony from *folk “natural” polyphonies* (see Appendix 5 in Supplementary Material) with their unrestrained abundance of possible vertical combinations of tones. Thus, Megrelian polyphony uses 18 types of chords on the VI, 14—on the V, and 13—on the IV degrees (Arom, 2010).

27. Audio: Henry VIII—Pastime with good company (c.1510). The 3-part vertical harmony is based on a few triads: 1 chord on the VI degree, 3—on the V, and 1—on the IV. http://bit.ly/1MLiDSx
28. Audio: Odola, Megrelian work song. The 3-part harmony contains plentiful variety of chords—in stark contrast to the example above. http://chirb.it/bgEdJa

Yet another crucial distinction is that *Non-Western “natural” homophonies* lack chordal functionality, treating the vertical harmonic aspect as secondary to melodic and timbral aspects. Such “chords” should be regarded as “timbre-harmonic clusters” that form no continuous linear development—nothing close to the Western notion of *standard harmonic progressions* (Kubik, 1999, p. 108). Tonal tension here plays no role in vertical organization, and is reserved to horizontal harmony alone: it is not chords that resolve into one another, but tones of the principal melody. Devoid of any hierarchic relationship, “chords” only thicken the texture or provide the reference frame to illuminate the mode in a manner of a pedal cluster of tones. Such are the “chords” of Japanese Sho music (for 17-reed pipes), whose frigidity throughout a composition offsets fluidity of the principal melody (Malm, 2000).

29. Audio: Ompeh, Efufu area of Ghana. The example of a call-and-response leader/chorus form popular in West Africa where the chorus contains multi-part dubbing of the melody—what appears as a chain of reproductions of the same “chord.” http://bit.ly/1TxJy4E

In fact, penetration of Western chordal mentality in non-Western music systems has had detrimental impact on their original tonal organization. Native performers start thinking musically in terms of Western triads and functions—which then remaps their modal intonations and produces new hybrid modes.

30. Audio: Men Kyrym, Crimean Tatar song, Uzbekistan. This solo song features unmistakable tonic, subdominant, and dominant functions in the melody—noticeably different from pentatonic organization that is traditional to neighboring Tatar ethnicities in Eastern Europe and Central Asia. http://chirb.it/z4besm

Crystallization of permanent chromatic “tendency tones” (Huron, 2006, p. 160) was another factor in shaping tonality: diatonic MPS afforded chromaticism only as melodic “accidents” of few types (Adams, 2010), whereas the tonal key embedded chromatic alterations as organic constituent of the PCS—transposable altogether with the tonic (Brown, 1992). Tonal chromaticism became “generative” (Forte, 1980)—in fact, *needed* to establish tonal integrity, so that its absence becomes a sign: an expression of sublimity, purity, or naiveté by strictly diatonic music (Vashkevich, 2006, p. 7).

Chromatic layer coexists with a diatonic layer as “chromatic pitch fields” (Burnett and Nitzberg, 2007) mapped in certain areas of PCS and remembered by music-users as a hierarchy of inter-connected “pitch alphabets” to be referenced during parsing of music (Deutsch, 2012).

Intelligibility of pitch alphabets embedded in a tonal key must be the immediate cause for the steady pattern of global Westernization, observable since the introduction of tonal keys in the eighteenth century. This process is often denigrated as “colonialistic,” but the truth of the matter is that functional tonality provided the cognitive framework that facilitated creation and comprehension of music for non-Western musicians, so that their Westernization is nothing but demonstration of universality of the cognitive benefits of hierarchic functionality (Yanov-Yanovskaya, 1999). Other issues, such as economic and political advantages, would not have come into play unless the Western music system presented a more effective way of encoding gradations in tension/relaxation than did the traditional local systems. Categorizing melody in terms of implied chords is a form of chunking—a way of compressing information^42^. Processing music in terms of standardized progressions of implied chords is another method of chunking, enabling even greater compression. Furthermore, both compression methods allow implicit learning of tonal regularities by mere exposure (Tillmann et al., 2000): figuring out which pitch constitutes a part of which chord, and which chord—part of which key, requires no teacher. Ease of implicit learning must be the underlying reason why many non-Western musicians tend to either switch to Western tonality or hybridize their native systems with tonality (Nettl, 1986). Adoption of tonality in the Third World countries essentially is the same as adoption of banking system or electrification.

Technically, what made Western tonality cognitively special was the crystallization of purely *intervallic* hierarchic typology, where vertical ISC came to replace melodic ISC as the basis of categorization in auditory perception. If early Medieval polyphony had all its parts share the same PS (Atkinson, 2008, p. 127), late Medieval polyphony was conceived linearly, often generating harmonic differences between parts (Bent, 1984). Compositionally, polyphonic harmony was constructed as a sum of monophonic harmonies, different parts in different species of 4ths and 5ths—until tonality of the early seventeenth century brought all parts to a common denominator of a single fixed IS, defined in semitones (Atcherson, 1973). So, the tonal composer conceived the entire texture as a single tonal construct which he had decided *precompositionally*—in contrast to the modal composer who could only discover the actual harmonic results after summing up all the parts (Mangani and Sabaino, 2008).

Nomothetic centripetal hierarchy of tones, fixed in their subordination and coordination relations, is quite analogous to the astronomic model of planets orbiting the sun, discovered during the Renaissance—as well as to the organization of depicted images in linear perspective (Cook, 2011). Tonality, heliocentricity, and perspective, all implement the same idea of harmonious arrangement of numerous peripheral objects in relation to a centered object. All three also deal with motion: physical, melodic, or “dynamic”—the latter term is reserved for reference to visual representation of “directed tension” in pictorial composition (Arnheim, 1984)—closely matching the idea of tonal tension in melodic harmony^43^.

And this is not a coincidence. Music composition has a long history of co-influence with architecture and ornamental design, all defined along the dimension of “virtual gravity” (Galeyev, 2003). Their connection comes naturally: sound and light are waves, subject to the same laws of reflection, dispersion, absorption, diffraction, and interference—differing mostly in wavelength: a musical sound-wave is about the size of a human, while optical wave is microscopic (Nazajkinskij, 1972, p. 116). The laws of physical space that rule audio and optic transmission prototype laws of virtual space constructed by art-works—and, here, musical texture becomes the cross-modal equivalent of visual depth (127).

Visual objects populate the *visual space*, whereas musical tones fill up the *musical texture* comprised of simultaneous sounding parts and voices. Percepts of pitch and visual size are cross-modally intertwined (Bien et al., 2012): we become aware of the presence of tones in the virtual music space essentially by the same mechanism as we locate visual objects. Melodic layer in musical texture serves as an equivalent of the visible surface—that is closest to the observer. In the pitch domain, discretization occurs in terms of intervals; in the visual domain—by formation of contours. Visual contour equates melodic contour (Terhardt, 1995)—both, outline the object of perception. The correspondence between the two has been known in musical literature since Jean-Jacques Rousseau (Galeyev, 2007). It also finds confirmation in experimental research (Weinstein and Gridley, 2010).

We relate one polyphonic part to another by estimating the vertical intervals between them in terms of their harmonicity, rhythmic simultaneity of tones, and contrast in melodic contour—once we identify the concurrent parts, we track them by their vertical order (Palmer and Holleran, 1994). Cardinality of vertical order is confirmed by long-standing compositional practice of avoidance of part-crossing in voice-leading (Huron, 1991). Despite dividing our attention between all the registered parts (Demany and Semal, 2013), pitch is best detected in the upper part—and this is disregarding whether or not the upper part contains more semantically important material (Palmer and Holleran, 1994). Moreover, the ERP studies of perception of polyphonic music indicate that formation of parallel audio information streams is pre-attentive and involves better encoding in the higher part, and even years of experience playing a low-range instrument does not reverse this bias (Trainor et al., 2014). The high part superiority effect was found in 7-month-old infants, suggesting automatic ordering of segregated audio streams (Marie and Trainor, 2013).

A similar effect occurs in visual perception of direct motion: we receive more information about the motion of *closer* objects, whereas optical invariants of *distant* motion are not picked up by the observer: closer motion is processed faster and with greater accuracy (DeLucia, 2008).

Observers can estimate trajectories of up to eight simultaneously moving objects (DeLucia and Novak, 1997)—quite on par with melodic motion in polyphonic parts of Italian Renaissance music, where five parts were the norm for sophisticated style, and 3-part writing was considered a sign of simple folk style (Dubravskaya, 1996, p. 56). Although experimental studies demonstrated that non-musicians are only able to count up to three concurrent parts, and musicians—four concurrent parts (Stoter et al., 2013), there is evidence (Huron, 1989) that at least some musicians can identify a 5-part polyphony when following not an analytical strategy of denumeration, but resort to a holistic strategy: estimating by how many more parts the texture is thicker than two parts. Such “thickness guess” would be perceptually analogous to estimation of crowdedness in a set of visual objects.

Interestingly, the tendency of the upper part in a multi-part setting to be the most busy in contrast to the lowest part that tends to house slower rhythm (Broze and Huron, 2012) remarkably resembles the motion parallax, sensitivity and awareness of which is found in 6-month-old infants (Condry and Yonas, 2013), raising questions about the genetic roots of greater acuity of perception of proximal data.

By the same token that we ascribe greater importance to objects that are coming toward us in the depth parameter, we ascribe greater urgency to sounds that are higher in pitch: “raising voice” implies calling for attention. Everyone knows from experience of vocalizing that raising of the voice involves activation of higher part of the vocal folds (Nazajkinskij, 1972, p. 156). Perhaps, being “higher in pitch” translates into “more important,” so as “being closer.” Yet another implication could be drawn from the Doppler effect, which engages cross-modality—but only in relation to the non-static stimuli. For dynamic stimuli, ascending pitch is congruent with growing in size, whereas descending pitch—with shrinking in size—in accordance with the visual illusion of an approaching object growing in size (Eitan et al., 2014).

## Renaissance polyphony and perspective: parallels in organization

Just like Renaissance painters went into experimentation with projective geometry to develop an eye for perspective, their music colleagues employed an empirical technology. At least from 1531, erasable tablets became a common object of trade, an accessory for “serious” business of composition, indispensable for sketching a music work (Owens, 2000). At first, composers used tablets as visual aids in configuration of pitches in a single part, but by 1612 entire multi-part compositions were drafted on a larger cartella (Owens, 1998, p. 74). Visual representation of music on tablet became mentalized. Monteverdi described his compositional process as “warping” melodic lines in his head before notating the music, a norm of composition at least since 1537: in his instructional treatise, Auctor Lampadius distinguished between a mental and a written stage in composing (64–73). Composers definitely abode by the graphic representation on a cartella when they employed “musical proportions” of painters and architects onto the proportions of sections in music form (Reynolds, 1987), employing decidedly spatial approach to planning a composition.

Composers were well aware of the shift in compositional approach: Tinctoris named 1437 as the Rubicon between “discordant” and “concordant” styles (Blackburn, 2013)—distinguished by the manner of laying out parts according to the harmonic plan. Lowinsky (1989) likened the emergence of this innovative “simultaneous concept of a polyphonic whole” with the development of the theory of perspective. By 1523, composers religiously followed the established technique of a simultaneous conception of all parts in a prescribed order (Dahlhaus, 2014, p. 94)—quite similar to artists religiously applying perspective.

31. Audio: Josquin—Ave Maria (1485), 4-part motet. Graphic visualization by Stephen Malinowsky demonstrates the role of spatiality in the distribution of musical phrases, evoking the state of equilibrium—corroborated by the dominance of C major triad throughout its tonal plan. http://bit.ly/1ishbHP
  Image 1. Piero della Francesca—Brera Madonna (1472). One of the first generation works that employed precise linear perspective, characterized by strong sense of harmonicity and proportionality of composition, contributing to the impression of serenity. http://bit.ly/1LHNDNR

Rise of tonality accompanied the rise of perspective—in the same cities, sharing the same user base, artistic ideals, and similar organizational principles. Perspective made the first public impression in 1425 (Edgerton, 2009, p. 5). Renaissance “*monality*” (Wienpahl, 1971)—modality with the major/minor triadic principle of organization—presented the first style of tonal rather than modal integration of harmony. It flourished in the genre of frottola, popular since 1490s (Prizer, 1975).

32. Audio: Tromboncino—Frottola “Ostinato vo' seguire” (1509). Solo melody establishes “one-point” perspective for the lute accompaniment that contains progression of chords and melodic figurations over implied chord. http://bit.ly/1LiHgzJ

Lute and guitar accompaniment exposed explicit “triadic thinking”—where music was processed in terms of melody supported by chords rather than counterpoint rules that still dominated vocal and keyboard treatises (Christensen, 1992). Similar homophonic unity characterized uni-syllabic delivery of the multi-part arrangements.

33. Audio: Josquin—El Grillo (1505), frottola for 4 vocal parts. This multi-part texture clearly demonstrates the integrative effect of “thinking in chords, with the melody in the upper part.” http://bit.ly/20XIyLU

Eventually, chordal thinking led to the establishment of *general bass* (Schulenberg, 1984): a practice of improvising a progression of chords to a given bass line—which can be viewed as an auditory equivalent of scaling 3D objects onto a 2D plain. Here, the bass acts as a ground line, whereas a vertical slice of texture is projected onto it by every beat, so that harmony controlled by a certain chord covers a specific number of beats aligned in relation to the bass. This constant projection of the vertical parameter onto horizontal time-line is what every keyboard player was supposed to do while accompanying an ensemble or a solo instrument (Bach, 1949).

34. Audio: Monteverdi—Zefira Torno (1632), madrigal for 2 parts and basso continuo. Here, complex harmonic pulse is set by the repetitive formula in the bass. Toward the end, repetitions are disrupted before the movement resumes and marks the end with a flourishing cadenza. http://bit.ly/1KnpZI4

General bass discloses *harmonic pulse* that serves has been serving as an important compositional means, equal to time signature in its formative power, in post-Renaissance music for most part of the Common Practice Period.

35. Audio: Pachelbel—Canon in D (1680). Graphic visualization by Stephen Malinowski demonstrates the formula of 8 chords that is consistently repeated in the bass, forming the progression of 5 vertical harmonies that unite the melodic material of all three melodic parts. http://bit.ly/1BJp5Vp

Pioneers of both, perspective and chordal textures, found their inspiration in Ancient Greece. Renaissance theorists of perspective were heavily drawing on treatises by Euclides and Ptolemy (Edgerton, 1974). Music theorists were equally heavily leaning on Aristoxenus and Ptolemy (Galilei, 2003). Vincenzo Galilei, the father of the famous astronomer, forged a new method of improvising harmonies on a string instrument to accompany his own singing (what he called “arie”), following the harmonic models of popular Italian folk songs of his time (Palisca, 1960). He believed that in doing so, he was restoring the venerated principles of Greek composition by creating perceptual analogs to Greek *tonos* and *modo.*

Both restorations, of “tonality” and perspective, relied on mathematics. Just as much as visual artists invested into projective gadgets, Renaissance musicians went into calculating optimal ratios for tuning in order to maintain the purity of chords while keeping the melodic line expressive. Moreover, musicians and artists utilized the same proportions—a tradition dating back to Peter Abelard, consummated in Alberti's theory of “musical proportions” (applying ratios of musical consonances to geometric figures) which remained influential until the eighteenth century (Pintore, 2004)^44^.

From the very beginning, Renaissance perspective was bound to proportionality (Wittkower, 1953). Perspective rules strikingly resemble centripetal gravity in tonality. Moreover, perspective rules are mirrored in *vertical*, as well as *horizontal* harmonic organization (Table 1).

**Table 1:** **Correspondence of principles of canonic linear perspective and principles of eighteenth century Western tonality**.

	**Principles of Linear Perspective**	**Principles of Vertical Harmony**	**Principles of Horizontal Harmony**
Centrality principles	The single viewpoint principle: ideally, a spectator is supposed to stand where the artist stood while drawing—at the equidistant point where all the rays that are reflected from the depicted objects join together, marking the center of projection. Observation from any other point is regarded as inferior, ought to be avoided (Greene, 1983).	Single part principality: The composer, the performer, and the listener all are supposed to focus attention on one part (voice) at a time—determined by placement of the theme: which can be defined as a distinct characteristic progression of motifs, reused in a music work in the capacity of a discrete unit of expression (Mazel, 1979, p. 150).	The tonicity principle: A particular tone ought to be recognized by the composer, performer, and listener as the strongest in stability amongst all tones used within a music work—thereby setting a single point where all the vectors of resolution for all unstable tones in the key would meet. If related keys were engaged in a composition, their tonics would be regarded as secondary.
Single ratio principles	The principle of single scale of proportions: Certain unit of measurement has to be selected, and then applied consistently to the entirety of the picture to measure and arrange intervals of space between the depicted objects in order of their position towards the spectator, representing their distance from him.	The principle of logarithmic intervallic spacing: The parts (voices) at the top of the texture must be closer to each other as compared to the parts (voices) at the bottom of the texture—ideally, staying close to logarithmic ratio, similar to intervallic distances between the partials in a harmonic series (Huron, 2001). The lower the register, the wider the intervals.	The principle of equal temperament: The entire MPS has to consist of tones that are equally spaced from one another by the interval of the well-tempered semitone, defined by division of an octave into 12 parts—so that the same piece of music would maintain its intervallic integrity precisely, if transposed to a different key.
Vectorization principles	The principle of single horizon: All the lines directed toward the depth of a depicted space, away from the spectator, must converge and vanish at the *rational* horizon line, defined by imagining an infinite horizontal plane. This line marks the “vanishing point” directly opposite to the “vantage point” occupied by the spectator. Only a slight deviation from the direct angle is afforded for the vantage point: the line that joins the vantage and the vanishing points sets the direction for viewing.	The principle of harmonic pulse: All the parts/voices are supposed to comprise vertical relations, usually prominent on strong metric time (downbeat), forming harmonies that obtain the anchor function in harmonic progressions – especially in faster tempo music (Dubovsky et al., 1965, p. 420). This vertical harmonic “slicing” of texture on metrically important time follows a set of rules for reduction of the melodic content of voices/parts to the frame of implied known chords—estimated from the lowest tone upwards.	The principle of key recapitulation: All tones of the composition must comply with the IS model of major or minor keys, and follow the tonal plan suitable for a given genre and music form, where the music work has to start and end in the same key, securing tonal integrity (Kholopov, 1988, p. 237). In this scheme, the “native” tonic of a key is found to oppose the “foreign” tonic of another key (albeit related), marking the vector of tonal development, indicative of the overall intricacy of music form.

Three main principles that distinguish the basic one-point linear perspective find close match in the standards of tonal organization in the music composed according to the functional tonality theory that was formulated by Rameau (1971).

Horizontal unification originated from vertical unification in Renaissance polyphony: Renaissance triadic tonality was born out of polyphonic texture, by satisfying the counterpoint rules in 4-part cadences (Randel, 1971). The modern day consensus of historic musicologists holds that compositional process of 14–15th century polyphony was based on expansion of 2-part into multi-part counterpoint (Moll, 2014). Renaissance mode essentially determined the melodic composition through cadential plan of the soprano and tenor parts—in a way similar to classical tonality (Meier, 1988, pp. 123–236)—and was largely controlled by usage of major/minor triads (406–421). Most of the Renaissance went into attempts to forge the meantone temperament with optimal sonance of triads on the degrees critical for most common keys (Lindley, 1976): thereby, the idea of optimal division of octave was interconnected to the idea of better sounding chord, which is essentially the same idea that governed logarithmic intervallic distribution between parts (Huron, 2001).

Tri-unification of centrality, ratio and vectorization is specific to Western civilization. Other cultures that came in touch with the theory of perspective, notably, Arabic and Chinese, did not develop a “psychological mental set” (Edgerton, 1974) out of the intellectual components of algebra, geometry, astronomy, religion, and art. Unique was the aspiration of Westerners to rationalize capitalism as an extension of moral law, seeking “symbiosis between God and mammon,” and putting math in the service of this double-goal—using it to define the Divine order while simultaneously gaining practical benefits in making music and fine art. Neither China nor Arabia, although technologically ahead of Europe by the fifteenth century, generated a culture based on the theoretical realization of mathematically ordered world, designed for practical production of utilities. Arabic scholars of the eleventh century knew Euclid and linear perspective (Belting, 2008), yet despite their contact with artists, perspective made zero impression on Arabic visual composition (Raynaud, 2009), not going any further than affecting depiction of details in architectural design (Yazar, 1991).

Solely in the West the artist adhered to the model of Divine emanation: God creates Man, and Man creates art, which is Divine. Christianity was a viable force in promoting perspective and tonality. Not only perspective was sanctified by the Quattrocento theologians as a faithful representation of Divine light reflected from an object (Edgerton, 2009, p. 29), and not only the foundation of major/minor chords were justified by Zarlino as uncovering perfection of God and Nature (Gozza, 2000, p. 58), but Christian understanding of omnipresence of God in every material particle paved the road to hierarchic unification of pictorial objects, as well as musical tones.

Hierarchic unification opened doors to compression of information, which enabled great complexity, unparalleled in other cultures.

36. Audio: Tallis—Spem in alium (c.1572), a 40-part motet. Extremely thick polyphonic texture, with complex division in groups. Graphic visualization by Stephen Malinowski http://bit.ly/1Lqc7PS
  Image 2. Bruegel the Elder—The Procession to Calvary (1564). Extensive landscape is filled up with detailed rendition of over a 100 characters, subdivided in multiple concurrent events—all integrated in a single bird's view perspective. http://bit.ly/1Q9XGm1

Tonal organization, just like pictorial perspective, establishes a particular model of symbolic representation of reality, shared by majority of the members of the same socio-cultural formation. Such model sustains over a period of time, instilling the same approach to reality in old and new generations—until the time when, for some reason, the socio-economic change renders this approach inadequate. Then, the old model of symbolic representation is abandoned and replaced by a new one. The entire Western history consists of numerous such symbolic “revolutions.” Each major historic period in Western civilization seems to carry its own “special perspective” (Panofsky, 1991, p. 21)—and a corresponding model of tonal organization (see Appendix 6 in Supplementary Material).

## Melodic line, geometric line and environmental topography: their connection

Just as the shaded line can inform whether the drawing engages the depth parameter, performance of a single melodic line can indicate the vertical and horizontal harmony.

A string player or singer, brought up with Western tonality, tunes each melodic tone according to its membership in the chord implied by the vertical harmony (Friberg et al., 2006)—even if the entire music work is solo. Tonal performance presents an ongoing challenge of constant mediation between *melodic* and *harmonic* tuning.

The pre-MPS folk music does not involve such intricacies. The archaic folk singer, raised in monodic music culture, is unaware of implied chords, following only the melodic aspect of tuning. Representatives of any primordial polyphonic folk traditions are likely to combine melodic and harmonic tuning of their respective polyphonic system. Kubik (1985) observed harmonic tuning “accents” amongst the encultured groups of African ethnicities—quite similar to having an accent in speaking a language^45^.

Nikolai Garbuzov coined the concept of *zonal hearing* to address the discrepancies of such “accents.” Through a series of methodic experiments he established a frequency range within which majority of musicians perceived a particular tone as “the same pitch”—a pitch zone. The exact values differed between different performers, testifying to the presence of a recognizable individual tuning style adopted by each musician as part of his “individual sound.” Yet, overall, all individual styles overlapped within a range of 58-76 cents, disclosing what appeared to be the range of cultural convention of tuning^46^. Each melodic interval slightly varied in zonal width: from 24 cents for unison to 76 cents for minor 2nd (Garbuzov, 1980, p. 92)^47^.

This variability reflects the difference in distribution of “tendency tones” in modal intonations. Each *pitch zone* can be viewed as part of representation of a traditionally established set of frequencies, remembered as a repertoire of standard intonations. The proof of that is the categorical perception of pitch errors: listeners judge about 60% deviations from a standard frequency as “correct” in tuning—possibly due to the same error correction mechanism that is engaged in perception of verbal phonemes (Siegel and Siegel, 1977).

A *pitch zone* is the aggregate value of all the expressive tunings for a given degree of a PS across all intonations that characterize this mode, afforded by the music-users (Garbuzov, 1980, p. 144). Narrowing a pitch zone for a specific degree in a mode/key necessarily indicates the presence of an important modal intonation that utilizes this degree—and can reveal a particular method of tonal organization. Narrowing of all the pitch zones would signal of the performer's concern with the vertical harmony.

In the same way that lack of harmonic functional hearing leads the folk-fiddler to render a tonal melody with overly strong “melodic” accent, the inability to integrate all the perceptual stimuli presented in a pictorial image prevents members of unacculturated groups from decoding the spatial information in perspective-based drawings (Hudson, 1967). The culprit here is the same—centering on a single aspect of organization due to lack of integrative experience. And just as folk musicians can be taught tonal music, non-Westernized people can learn to draw in linear perspective (Mshelua and Lapidus, 1990).

Non-classical listener hears a modernistic composition as a sort of “noise” because he treats music “unmusically”: he applies the hearing criteria for environmental listening (Nazajkinskij, 1972, p. 173). He employs fissure—isolation of a presumably important sound signal from unimportant ones. Musical listening, in contrary, requires *integration* of simultaneous sounds, calling for application of an adequate scheme of tonal coding. Not knowing the scheme forces the listener to “flatten” the tonal richness into a guessed “melody”—similar to how a child “flattens” the perspective organization while drawing a 3D object. There is experimental evidence that pitch is mapped to height for isolated tones differently than for melodic intervals—by non-musicians, while musicians process pitch automatically (Lidji et al., 2007). This suggests that spatial representation of tonal organization has to be learned.

The type of limitation, experienced by a viewer while encoding/decoding a perspective-based image, can be telling of his tonal hearing (see Appendix 7 in Supplementary Material for the example of tonal/spatial correspondence in perception of Nenets traditional art).

Each scheme of tonal organization abstracts those perceptual features of the living environment that are crucial for the success of the typical representative of a given culture in pursuing his life goals. Sophistication of a scheme is only an answer to the sophistication of socio-cultural conditions for survival: 2-dimensional representation with indefinite intervallic values sufficed the earliest schemes, whereas later schemes required more dimensions and greater precision.

Tonality, as well as perspective, can be understood as a generic system of representation of 4-dimensional space in 2-dimensional framework: pitch/time for music and x/y for pictorial art, where the observation point is defined according to the position of the individual perceiver. Similar dimensional conversion characterizes literature, where perceptual reality is described from an angle of the speaker, in 2D fashion, word-per-time (Uspensky, 1995, p. 80). Noteworthy, the onset of naturalistic depiction, literature, and harmonic theory all concur in Mesopotamian urban culture.

The next landmark, invention of perspective, paralleled chromatic music and lyric poetry that became means of individual self-identification. Here, the cognitive centerpiece that supported them all was concise definition of one's position in relation to something: be it a depicted object, a composed melody in a certain mode, or a subject of a poem.

Crystallization of linear perspective c.1300 further advanced the self-orientation function: defining a specific observation point *optimal* for the observed object. The epitome was the spectator positioning himself inside the church to view a fresco—thereby the emerging Franciscan order sought to “include” the viewer into the painting, increasing intensity of the aesthetic emotions and attracting more people to join the congregation (Benton, 1989). The musical equivalent here would be the style of *musica reservata* where, in the polyphonic texture, the listener had to track a theme that expressed a particular emotion related to the lyrics—shaded tonally with the help of chromaticism (Meier and Dittmer, 1956). There, orientation occurred by triadic sonority and melodic diatonicity guiding the listener in mapping spots of tension and relaxation. Polyphony, tonality, and perspective, each in its own way, all faceted the same sense of individualism that flourished circa fifteenth century (Hyer, 2008).

Their symbiosis did not fall apart after homophony replaced polyphony as the dominant compositional method during mid-eighteenth century^48^.

37. Audio: Schumann—Scherzo, Piano Quintet Eb Major. Homophonic parts are engaged in complex textural “kaleidoscope” nexus by means of their pitch contours and rhythmic patterns—evident in this graphic visualization by Stephen Malinowski. http://bit.ly/1QoCi8S

Both, implementation of tonality in musical texture and linear perspective in 3D representation, serve the same purpose of the orientation of an individual in sonic/visual space by means of defining the foreground, background, and the distance between them. The earliest germ of foreground/background relation in harmony is constituted by the notion of “tonal distance” in monody: the remoteness of the auditioned tone from a remembered “tonic.” The idea of coordination of tonics was born in Sumer, with the establishment of the sister-mode family, in MPS modes. It took a while before, in Baroque music, it evolved into “key distance” and still later—into “key relationship,” determined by the circle of 5ths. Exploration of “tonal distance” throughout history of music very much parallels exploration of representation of spatiality in pictorial art—and parallels do not stop there: the foreground/background dialectics penetrates into sculpture, architecture, and theater (Grauer, 1996).

Tonal organization is more likely to prototype pictorial representation than vice versa. The archetype could be the Central Asian tradition of carpet weaving, where weavers use chants to aid memorization of ornamental patterns—possibly dating to an older Indo-European tradition, mentioned by Homer and Bacchylides (Tuck, 2006).

Music behaviors are more widespread than drawing behaviors—especially in egalitarian-oriented folk cultures. Every known music tradition follows some kind of “music theory” (Blacking et al., 1995, p. 224), whereas the number of cultures that lack tradition and theory of depiction is vast. In tribal society, it is hard to estimate personal use of music by every tribe-member (although in numerous cultures, “personal songs” occupy a prominent place), but evidently collective use of music can take 2–3 h daily in a subsistent society that lives under constant environmental pressure, yet dedicates to music so much of the tribe's resources (Huron, 2003, p. 64). Despite intense professionalization, levels of public engagement in music-making stayed high even in the industrial West (congregational and amateur music)—until the 1930s (Rothstein, 2006; Chybowski, 2008). Nothing comparable in scale is known in art history: neither in prehistoric^49^ (Curtis, 2006, p. 235), modern tribal^50^ (Myers, 2002, p. 63), ancient (Hauser, 1999, p.15), medieval (Florensky, 1996) nor early modern art (Pears, 1991)—drawing from nature has remained the privilege of few ordained and/or gifted artists until the emergence of welfare system and public education (Alexander and Rueschemeyer, 2005).

Relatively clear public consensus on what constitutes excellence in music vs. unclear criteria of excellence in visual arts (Milbrath et al., 2015) suggests that it is music that conserves the method of organization for spatial representation. Drawing adheres to a theory only in religious applications, such as iconography, or in professional institutions, such as academy of arts—where such theory noticeably lacks uniformity, with different schools adhering to different techniques and methods of drawing.

Depiction requires extensive knowledge of visual shapes and command of looking for strategies in determining which level of description to use for analysis of the depicted object and “good” copying performance (Pratt, 1984). Hockney (2006) informs about the multiplicity of technical gadgets utilized by artists throughout ages, starting from Euclid^51^. Producing and reproducing a tune does not require any comparable technology. To succeed in capturing the spatial organization the drawer must learn to make things look “right” by drawing them “wrong” (Arnheim, 1954, p. 76). Copying a tune by ear is much more intuitive than copying an object by eye.

Unlike ubiquitous acquisition of singing skills (Hargreaves, 1986, pp. 66–104), “learning” how to draw presents a substantial cognitive barrier to children due to required alternations from an object- to a viewer-centered representation, involving curbing of “natural” instincts of viewing (Freeman, 1987). Children's drawing improves by age, because they elaborate better strategies to mediate between “what” and “how” to draw (Park and I, 1995). Their knowledge of what to draw typically interferes with their choice of depicting strategy (Tallandini and Morassi, 2008). The effective solution becomes abstraction of a graphic-motor schemata adopted per object type (Phillips et al., 1978) based upon the criterion of reducing ambiguity in picture (Willats, 1977). The entire development of pictorial skills throughout childhood is shaped by this goal (Morra, 2008). Noteworthy, children are more prone to copy each other's pictures than to infer from life (Wilson and Wilson, 1984).

Reproducing a tune is nowhere near as technical, intellectual, problematic, and cognitively demanding as depicting an object. Russian state general education program requires recognition/reproduction of parts in multi-part settings from 10-year old children (Apraksina, 1983, p. 110)—and majority of the population has been complying to this program for few decades. Children of around that age can infer a tune from a polyphonic folk-song by themselves without any assistance (Naumenko, 2013, p. 124). Flattening of a polyphonic song into a monophonic version is a common trait in many ethnic cultures (Jordania, 2006, pp. 376–378). Conversion of musical texture seems to present no obstacle comparable to 3D-2D conversion. No wonder, musicality is considered a biological trait of Homo (Morley, 2013, p. 5), whereas ability to realistic depiction is reserved for specially gifted (Golomb, 2004, pp. 201–278).

Music occupies a prominent position in children development, only magnifying toward adolescence. In contrast, drawing attracts children at the age of 2–3 years, waning afterwards (Winner, 2007). An adolescent at large becomes estranged to drawing, once so favored at a younger age—in polar opposition to his musical interests (Vygotsky, 1984, p. 61). Modern 8–18 year olds spend on average 2.5 h daily for music (Rideout et al., 2010). The underlying reason must be affiliation of music with cognitive consonance. Vygotsky (2008, p. 397) underlines that childhood presents the most tragic stage in life, when the organism finds itself in the greatest disharmony with the environment, being under constant pressure to quickly bring itself “in-tune” with its surroundings—this cognitive dissonance calls for “music of upbringing”—harmonious nurturing. And nothing answers this call better than music itself.

## The course of tonal evolution

Existence of 14 schemes of tonal organization (see Appendix 8 in Supplementary Material) suggests that tonal order in music manifests a general mental organizational scheme, which serves as a cultural *adaptation of human perceptive apparatus* to a life style optimal in a certain environment (Lomax, 2004, p. 281). Younger generations in a socio-cultural formation keep reproducing the same schemata as long as the same life style pertains. Music appears to act as a principal enforcer of this mental enculturation. The emotional nature of music, its affinity with entrainment, and its capacity to reduce cognitive conflict, all make tonal organization a powerful tool in shaping a methodology of thinking within a given community. Gravitational laws of musical virtual reality reflect perception of physical laws of phenomenological reality, and link the navigation strategies for both of them.

The rigorous and systematic organization of music is a product of pressure imposed by natural selection to adequately collect information about the kinds of objects that exist in the environment, what they do, and how they can possibly be used (Terhardt, 1995). Through a complex process of conversion of frequency data into pitch information, the brain exercises an organizational scheme that is archetypical for majority of music users in a given community. Nature provides humans with some “template” of sensory categories in their default values (i.e., preference for vertical and horizontal consonance, binary meter and frequency range of the speaking voice), and cultural application modifies this “template” into a “document” that would serve a particular need, common for a given community. Such “document” seems to work like a script for our senses, organizing them to operate following the coherent set of principles (Walker, 2004). Auditory imagery is known to evoke visual and/or kinesthetic experience, which in turn influences auditory imagery (Hubbard, 2013). Capacity to make emotional judgments about imagined music (Halpern, 2012) makes music a par excellence tool for social engineering.

Pitch presents a perfect medium for exercising discretization: human hearing is inherently zonal—it cannot distinguish between different pitches in a clear-cut manner; there is a zone between frequencies of tones in a familiar PS, upon hearing of which the listener cannot reliably tell which pitch it is. Even extremely gifted and well-trained soloists observe 6-21 cent inter-zonal threshold (Garbuzov, 1980, p. 205). Parncutt and Cohen (1995) also specify the 10–20 cent threshold^52^.

This “margin for error” is responsible for mutability of pitch detection, requiring some error-correction mechanisms^53^. Jordan (1987) demonstrated that listeners can discriminate intervals of 25-50 cents, but categorize them in terms of diatonic IS, and musicians do so more than non-musicians. Models of tonal organization can be viewed exactly as algorithms designed to minimize indiscretion in detection and production of pitches in a given socio-cultural formation. Similar mechanisms must be at play in the perception of other important attributes of musical sound. Garbuzov experimentally identified the zonal nature of perception of dynamics (Garbuzov, 1955), timbre (Garbuzov, 1956), tempo, and rhythm (Garbuzov, 1950). Garbuzov's pupil, Rags (1980, p. 33) sums them in the following definition: “*Zone* is the measure—a quantitative characteristics of a musical conceptualization…of the inter-relationships between certain qualities of musical tones”—product of an attempt to discretize gradations in “fuzzy sets” of auditory parameters.

It appears that during the passed century the zones and zonal thresholds have shrunken in perception of Western classical music (Rags, 1980, p. 33). For non-tempered intervals, inter-zonal thresholds are about 2–2.5 times more narrow than the thresholds of the adjacent zones (Garbuzov, 1980, pp. 89–99). Moran and Pratt (1926) confirmed this ratio for the tempered scale. Comparative investigation of how Western and native Java musicians estimate intervals in Western and gamelan music demonstrate that Javanese listeners have wider zones of pitch uncertainty in auditioning Western music than Western listeners do in Javanese music (Perlman and Krumhansl, 1996). Léothaud et al. (1997) report that in Central African octave-equivalent polyphony, differences as large as 80 cents^54^ between two structurally equivalent keys are still considered a unison.

Another trend, noticeable throughout the evolution of tonal organization, is the progressive increase of the number of pitch zones within an ambitus of a mode (Rags, 1980, p. 42).

The more modern the stage, the more pitch gradations fit within the same frequency band (from 2 entities in khasmatonal to 17 entities in tonal key), and the narrower are the zones reserved for each of the pitch entities (from up to 1½ octaves for khasmatonal to 24 cents for tonal key; Table 2).

**Table 2:** **Pitch zone discrimination throughout the evolution of tonal organization in low voice vocal music**.

**Type of tonal organization**	**Number of pitch zone classes in the music system**	**Maximal number of pitch zones in the vocal compass (2 octaves)**	**Typical width of a pitch zone**	**Normative tuning precision**
Pre-mode	1 = whole compass	undetermined	2 octaves	Absent
Khasmatonal	2-3 registers	undetermined	≥ octave	Widely variable
Ekmelic	2-4 regions	4 indefinite degrees	2nd—4th	Selectively variable
Oligotonal	2-4 pitches/regions	4 definite/indefinite degrees	2nd—3rd	Fixed/variable
Mesotonal	5-6 pitches	6 definite degrees	2nd/3rd	Fixed/bi(tri)-optional
Mutitonal	7-10 (<) pitches	10 (<) definite degrees	≥ semitone	Fixed/bi(tri)-optional
Pentatonic	5 pitch classes	11 pitches (5 degrees)	1/1½ tone	Fixed
Heptatonic	7 pitch classes	15 pitches (7 degrees)	1/½ tone	Fixed
Diatonic MPS	7 pitch classes	15 pitches (7 degrees)	1/½ tone	Concise
Hypermode	3-5 pitch classes	10-12 pitches (3-5 degrees)	≥ semitone	Concise
Chromatic MPS	16 pitch classes	27 pitches (4 degrees)	63-84 cents	Microtonally concise
Tonality	17 pitch classes	35 pitches (7 degrees)	24-76 cents	Microtonally concise

“*Pitch zone” means the range of pitch values that is operated as a single entity in tonal organization. “Pitch zone class” is the reproduction of the same “pitch zone” over an octave. The compass of an average singing voice is taken as 2 octaves (Kob et al., 2011). For the khasmatonal music the compass is an octave wider because of inclusion of falsetto singing (Heylen et al., 2002). Oligotonal music engages no more than 4 degrees, some of which are fixed in pitch, while others variable. “Bi-optional” differs from “variable” by narrowing the tuning options to 2: “standard” or inflected. “Tri-optional” offers “standard,” sharpened or flattened versions. Pentatonic/heptatonic modes and MPS introduce full octave equivalence and fix all pitch values. Ancient Greek chromatic system contains 16 discrete PCs within A2-A4, 11 of which are octave-equivalent. That leaves 27 available pitch slots in the range reserved for vocal composition. The critical width of chromatic semitone can be assessed based on discussions of chromatic shading in period treatises (Hagel, 2009, p. 217). Vocal solo music in Western tonality contains 17 PCs (and 35 slots in 2-octave range) since it tends to adhere to the Pythagorean model (Ghrab, 2005), especially for non-professional singers (Devaney et al., 2011), making semitones narrower than well-tempered tuning (Vurma and Ross, 2006)*^54^.

**Figure 5:**
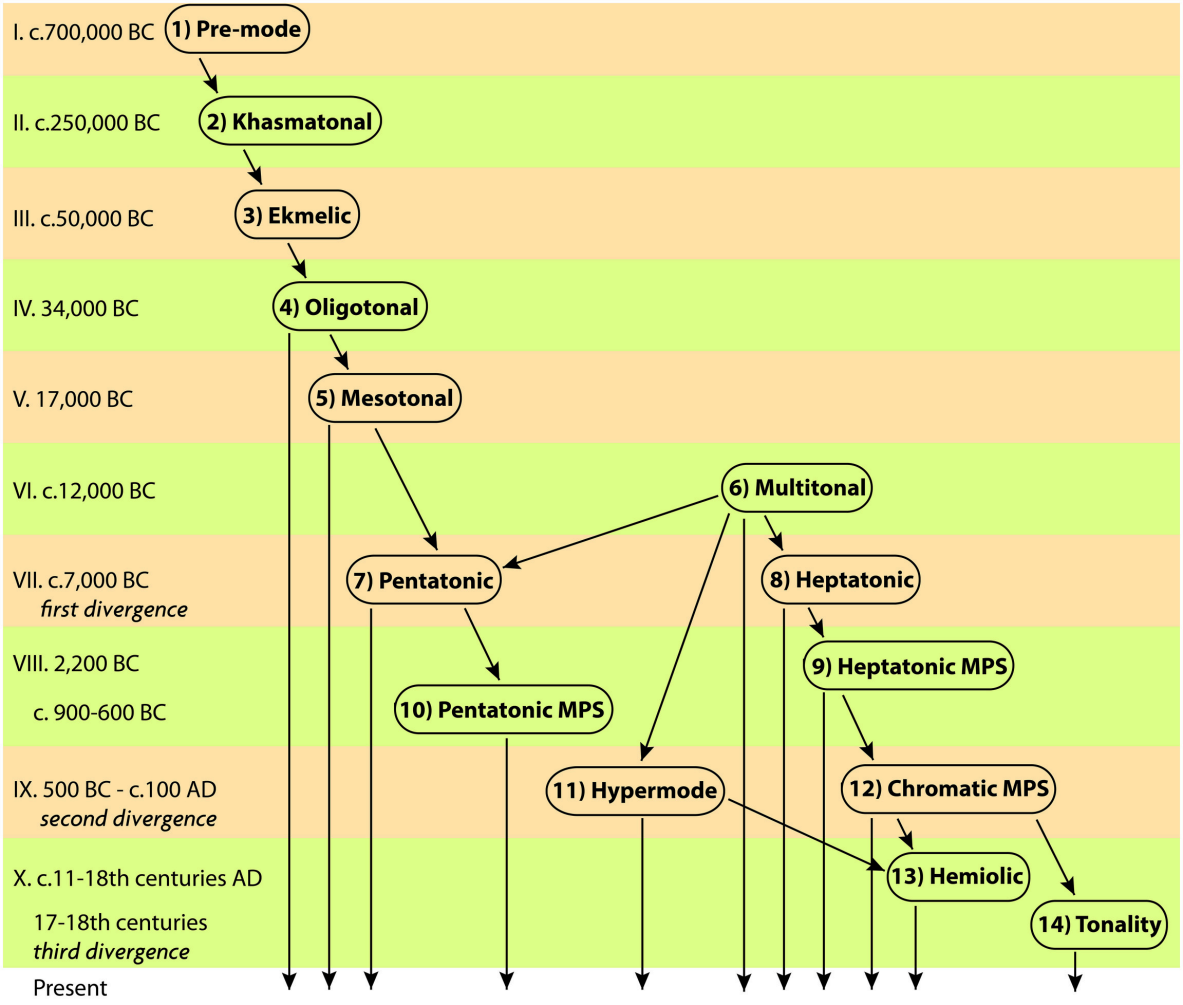
**14 schemes of tonal organization that occur through 10 stages**. Stages 1–6 are cumulative until the divergence between pentatony and heptatony. Second divergence occurred between chromatic and diatonic MPSs, bringing to life hypermode. Chromatic MPS produced yet another divergence between hemiolic modality and tonality. The importance of this is that competence in first six schemes is shared by all music users, who can switch between operating in any of them and their “native” music system: i.e., a mother raised on music of Western tonality employs khasmatonal organization in her motherese. Divergence, on the other hand, requires learning of a scheme that is diverged in relation to the music user's native system.

Ten stages of tonal evolution are mostly *cumulative*—(Figure 5)^55^. Alekseyev (1986) illustrates how archaic principles of pitch organization are still present in modern music. *Khasmatonal* principle manifests itself in folk yodeling or rock music growling. *Ekmelic* principle comes out in gliding inflections and half-spoken pitches of blues or rap.

A music-user from a later stage of tonal development possesses apparatus to decode the representational schemata of earlier cultural formations, but a listener raised exclusively on music of earlier stages cannot adequately decode spatial aspects in music of a later stage. This is most obvious in the communication of motherese: Western mothers have no problems reserving to the khasmatonal organization in their singing despite their competence in tonality (see Presentation-3 from Part-I), while for their babies khasmatonal music remains the only form of tonal organization that they seem to be able to fully follow. Their perception of tonality is severely limited (Trehub, 1987). Walker (1987) concludes his overview of the experimental studies of cross-cultural pitch perception: “the more remote from Western auditory and visual cultural artifacts the subjects, the less likely they are to select the kind of visual metaphor for sound than those trained in Western musical practices consistently select.”

## General summary

Zemtsovsky (2012) considers the very concept of musical tone to carry a signature of tonal organization. Artificiality of periodic oscillation in musical vocalization calls for justification. Hearing a single pitched tone generates expectation, and hence, induces gravitational projection toward the following pitched tone. In order to make sense, a tone *must* proceed to another tone (or be repeated), forming an intonation. And a melodic intonation becomes not only a brick in the melody, but a *quantum of gravity* in the virtual reality of music. The entire progression of 11 modal schemes proceeds toward “triadic” mentality of perspectival depth and polyphonic texture—this direction is evident not only in Western music, but in other cultures as well, where it materializes in textures of various density depending on the social order, environmental factors, and cultural exchange (Vinogradov, 1973, p. 106).

It appears that progressive increase in tonal acuity is the result of evolution in melodic complexity. Crystallization of melodic contour typology brought to life ekmelic pitch regions to replace much wider and less definite khasmatonal registers. Forging of absolute intervals of 2nd, 3rd, and 4th between the anchored tones replaced ekmelic regions with oligotonal pitches. Triad induction caused variable tuning of unstable degrees to recede to bi-optional tuning. Transposition-by-degree of pentatonic and hemitonic motives fixed unstable degrees in tuning, and narrowed the pitch zone to a semitone. Institution of chromatic alterations further narrowed the zones below a semitone. This entire process of “pitch-zooming” presents an adaptation to the cultural need for greater discrimination.

Parallel increase in PCs and establishment of complex tonal hierarchy within a mode made encoding of tonal relations effective enough to support simultaneous data transmission via multiple sound streams, up to 7-componental music texture. Each of the components simultaneously encodes information by means of idioms of pitch, rhythm, meter, and harmony, plus the contribution of expressive parameters of dynamics, tempo, articulation, timbre, and music form^56^. Such unprecedented density of information makes music a par excellence tool for abstraction of important features in a living environment, and mediation of their schemata between the members of the same social group in the best interests of each individual.

Language is an important factor that shaped melodic intonation and its tonal organization. Propensity of language communication to cultivate cognitive dissonance finds a counterbalance in propensity of musical communication to cultivate “cognitive consonance”: *a state of inspiration and empowerment, characterized by experience of integrity, clarity, and consistency of attitudes in a person, as well as togetherness within a group*. People sing together to share the same experience, but talk one after another—usually to resolve an issue. Listeners routinely engage into musical behaviors for relaxation or recreation, which is less common for speech that normally cannot run in a semi-automatic regime, unlike music. Less stressful manner of experiencing music is likely the result of different strategies preferred in comprehension of music vs. speech. The tendency to fuse spectral content dominates the perception of music, while the tendency to segregate phonemes prevails in the perception of pitch (Bregman, 1994, pp. 461–589). Then, the need to differentiate between the spectral elements greatly promotes the realization of opposition and discrepancy within the sound material, whereas the need to integrate partials into musical tones, and tones into chords, promotes the realization of similarity.

It looks like both forms of communication co-evolved from some primordial animal-like vocalization, defining each other through the different treatment of pitch—each forging its dedicated processing system (Zatorre and Baum, 2012). The ethnomusicological evidence leads one to believe that melodic intonation was formed by borrowing and exaggerating the pitch contour of the conventional verbal intonations, while contrasting the verbal timbral organization and articulation style. The decidedly “artificial” manner of khasmatonal and ekmelic intonations was shaped by counter-distinction to the “natural” manner of speech. Stabilization of pitch in oligotonal music led to reduction in timbral complexity, changing the manner of its opposition to speech. Oligotonal music started contrasting speech primarily by greater harmonicity of its tones and their timbral uniformity—a “bel canto” style of sound production, employed by a given music culture as “canonic.” Speech intonation, on the other hand, featured much more diverse spectral content of each of its phonemes, instituting the fission/fusion antithesis. At this point permanence in tuning of PS and hierarchic ordering of pitches—as well as timbral uniformity—became the “musical” traits. This border is not clear-cut: orators and poets often “melodize” their speech (Nazajkinskij, 1972, p. 261), whereas composers engage in a “recitative” melodic style, sometimes deliberately emulating verbal intonation (Pearl, 2006). However, overall, music tends to contrast speech in pitch organization—most evident in tonal languages, where the musical pitch contour of traditional songs often violates the normative intonation contours of the lyrics (List, 1961).

In a compensatory manner, cardinal stages in the development of language tend to concur with shifts in the method of tonal organization. Development of sentence syntax conditioned transition from khasmatonal to ekmelic mode. Emergence of epic poetry promoted oligotonal music. Introduction of literacy boosted the emergence of philosophy, law, and science—promulgating prescriptive theory of harmony and diatonic music.

“Cognitive consonance” function works in both, horizontal and vertical dimensions of music. Any ensemble attempt to sing a melody with fixed pitches would contain asynchrony, when one performer produces a new pitch while the other still carries the previous pitch—thereby converting melodic interval into harmonic. Just as a single pitch is prone to generate a 2-tone intonation, it is prone to generate a 2-tone vertical harmony. And this is where spatial representation comes into play: 2-tone vertical harmony is cross-modally equivalent to 2D pictorial representation, and 3-tone harmony (chord)—to 3D. In the same way it takes at least three objects to hint to three distinct levels of pictorial depth (Cook et al., 2008), three simultaneous tones imply a certain texture—both representations manifesting the same higher order cognitive scheme of 3-factor opposition that typifies the Renaissance astronomy, major/minor tonal organization, and linear perspective—all setting the framework for “triadic cognition”—the ability to reason by 3-way associations (“X in relation to Y in light of Z”; Cook, 2012, p. 12). Upgrade from dyadic thinking constitutes the greatest achievement of human civilization^57^, underlying all the technological achievements. Possibly, *tetradic thinking* is there to follow.

By cross-modal implication, musical intonation differs from verbal. Speech is not heard in terms of visualizing auditioned tones the way music is. The bifurcation point must have occurred during the oligotonal stage:

- Speech adhered to “low-cognition” standard of correspondence “higher pitches—good disposition” and “lower pitches—expression of aggression”—shared by communication of many mammals (Ohala, 2006).
- Music adhered to a “high-cognition” standard, present only amongst humans—already evident in ekmelic organization, where ascending inclination corresponds to stress/climax, while descending—to relaxation/resolution (Alekseyev, 1976, p. 129).

Synesthetic capacity, so vivid in music, must be responsible for re-wiring of the registral associations—and this should be viewed as yet another display of integrative nature of music: musical tones trigger 3D spatial representation, unlike “flat” sounds of speech^58^. Establishment of centripetal gravity and permanent tuning throughout an oligotonal song brought to life this “*hologram” effect*.

Melodic intonation is immensely important for human culture of thinking. Once generated, melodic intonation receives life of its own. It can be adopted by many music users and embedded into a mode^59^. It can be developed: by contribution of each of them. Historic development of intonation resembles epistemology and historic changes in syntax of language. Subsequently, musical mode functions as a “complex dynamic and self-regulated system” that operates on genetic-like principles of optimal adaptation to the cultural environment (Alekseyev, 1976, p. 113).

Analysis of intonations in a song enables reconstruction of the gravitational system observed by the creator of that song—in a way similar to a paleontologist's reconstruction of a fossil from a bunch of bones. Mode is capable of showing which intonations have stayed popular within a community of users over an extended period of time^60^. This makes it possible to generalize a gravitational scheme exercised in the music of that culture, and to formulate the cognitive style peculiar to a given historical socio-cultural formation. Understanding this cognitive style allows for extrapolating a method of musical order in organization of other cultural activities. Overall, music appears as a naturally formed testing ground for various principles of representation of reality, conscious and unconscious (Hubbard, 2007), used to prime emotional reactions to music idioms, thereby establishing and cultivating conventional standards of intellectual and emotional intelligence.

## Author contributions

The author confirms being the sole contributor of this work and approved it for publication.

### Conflict of interest statement

The author declares that the research was conducted in the absence of any commercial or financial relationships that could be construed as a potential conflict of interest.

## Footnotes

^1^The term “tonal organization” here encompasses not only what is called “tonality,” but every form of integrating musical tones together in a harmonious ensemble— including music where fundamental frequencies are not well marked in the spectral content, i.e., gamelan or throat singing.

^2^The term “mode” is a modern day concept applied retroactively to address the tonal coherence of a pitch set (PS) selected as the formative basis for music-making: each set adhered to a particular scale, probably associated with some tonal focus—unfortunately, the only surviving melody is insufficient to infer the modal characteristics of Babylonian music system (West, 1994).

^3^According to UET-VII-126 tablet, the open strings tuning (pitum) is G-A-B-C-D-E-F-G-A, producing the same IS (tone+tone+semitone+tone+tone+semitone) in either direction (Crickmore, 2009b).

^4^Pythagorean Triplets probably originated from the Babylonian tablets (Skinner, 2006, pp. 17–18). Sacredness of number 9 in Antiquity stemmed from the Babylonian Lunar calendar (Wiltse and Palmer, 2011). Babylonian proportions were perpetuated in the Parthenon (Kappraff, 2006).

^5^“Displacement” here refers to the last tone of the melodic 2nd which perceptually “erases the trace” of the initial tone in the short-term memory. Such effect seems to be universal (Larson and McAdams, 2004; Deutsch, 2012; Larson, 2012) and therefore applicable to the Ancient Greek music.

^6^Such as arpeggio passages.

^7^“Metric” division of a 4th is comfortably visible on a woodwind/string instrument through the placement of holes/frets—and relatable to the resulting changes in pitch (Beliayev, 1990). The difference between perfect 5th (702 cents) and 4th (498) produces major 2nd (204). The succession of 2nds marks the I, II, and III degrees, thereby defining the minor 2nd (90 cents) left out between the III and IV degrees (90 = 498−2 × 204).

^8^In Ancient Greek theory our Phrygian tetrachord was called Dorian, and viewed as the trademark of Greek civility, ideal for representation of universal harmony (Crickmore, 2006). This Greek “Dorian” is congruent to the Babylonian Isartu [“normal” in Akkadian (Kilmer, 1971)] mode (Duchesne-Guillemin, 1984), by its name suggesting its commonality in Mesopotamia.

^9^Nicomachus considered conjunct E-F-G-A-Bb-C-D the primordial scale comprised by conjoining tetrachords, only later disjointed by Pythagoras to form an octave scale (West, 1992, p. 176).

^10^Modal mutability of multiple anchors was explained in Part-1.

^11^Such reproduction of a progression of musical tones that retains the intervallic relations between all successive tones (C-D-E-F −> D-E-F#-G)—“natural” for singers who don't have perfect pitch.

^12^Such reproduction that adjusts the intervallic relations between the successive tones to match the PS (C-D-E-F -> D-E-F-G)—“learned” by all singers.

^13^Joseph Yasser also observed similar lineage in successive development of what he called “quartve,” “quintave” and octave equivalence in evolution of tonal organization (Yasser, 1948).

^14^Musicians in that study had easier time discriminating the dominant from the repeated tonic—indicating that the “dominant” function is learned through music-making.

^15^West (1981, p. 127) also describes a similar process of modal genesis of sister-modes by means of modal transposition, producing Dorian, Phrygian, and Lydian scales in Helladic Greece, according to the testimony of Alcman, 7th century BC.

^16^Variety of songs accompanied the liturgy in Babylonian temples, with no fewer than 6 different compositions (Löhnert, 2011)—at least some of which must have proceeded without stopping while requiring different modes for their liturgical expression.

^17^Something similar to this must have happened in Helladic music, where the diatonic genus apparently allowed for building the “sister-scales” from any of the tones of a system—and not just stable tones, as in Hellenic music (Franklin, 2002). Subsequently, “old” music was criticized for producing tunes that all sounded “the same” (probably due to the perceived lack of “expressive tuning” that resurfaced again in chromatic music of the 5th century BC).

^18^This is achieved by: (1) increasing the amount of strings on harp/lyre; (2) using the fingerboard to play closed strings on lute.

^19^In Mesopotamia, genre classification was the business of scribes, and proceeded according to the intended manner of performance, marked in notation with the help of the subscripts and rubrics (Rubio, 2009, p. 23).

^20^Sumerian culture marks onset of professionally produced music, coining the term 'šir' that implied authorship of a song (Krispijn, 2010).

^21^Evidently, all registered love-songs belonged to one of seven standard modes, three of which were by far more common for expression of love. Specialization required expertise from the musicians—to prepare music ahead of time, and their performance could score varying degrees of success, reflected by difference in wage (Ziegler, 2011).

^22^Because of its conservative nature (see Part-1), the pentatonic MPS lagged behind the heptatonic. The Old Babylonian tablet CBS-1766 postulated heptatonic tuning (Dumbrill, 2007) in the eighteenth century BC (Friberg, 2011), likely descending from the older tradition, perhaps the twenty sixth century, when the Lyre of Ur was made (Rowan, 2013).

^23^Testifying to universality of Mazel's theory of odd/even functional induction (Mazel, 1952) (see Part-1).

^24^Like Mesopotamian symmetry, this whole-tone division is foreign to local folk cultures of the Far East, supporting the point that opposition of village folk to palace/temple authored music corresponds to opposition of pre-MPS to MPS systems, and is a cross-cultural trait that has cognitive roots.

^25^Cuneiform writing was popular amongst commoners—cursive signs and stenography were widespread (Veldhuis, 2011). Functional and technical literacy were taught informally through apprenticeships and “community of practice” (Robson, 2008, p. 52).

^26^The issue of competence versus incompetence is expressly addressed in a survived dialogue between a scribe and his student, which asserts the necessity to learn whatever it takes to figure out a solution (Vanstiphout, 1997).

^27^The third type, pentachord-based, is discussed in the Appendix 2 in Supplementary Material.

^28^Hexáechos literally means “six modes” in reference to the smaller version of the principal Byzantine MPS—oktôçchos (Kholopov, 2006, p. 68). The Russian implementation of hexáechos is called “obykhod” (“common”), codified in 1772, but probably much older (Kholopov, 2004). The influence of obykhod on Russian folk music is tracked to the fifteenth century (Rudneva, 1994, p. 121).

^29^Throughout Antiquity and Middle Ages a common tool for conception and assessment of tonal organization was monochord with letters designating pitches (Bower, 2002)—a proof of musical thinking in terms of the “legitimate” PCs.

^30^An unstable mesotonal/multitonal degree can appear chromatic, misleading ethnographers in their transcriptions (Ambrazevičius and Wiśniewska, 2008). Correct spelling of pitches can be verified by computer analysis of the frequency of each discrete musical tone within a song, then defining its PS by mediating between the average values received by incrementing the degrees in the ambitus and the melodic intonations established by morphological analysis of a song (see Presentation-1 for an example).

^31^Thus, maqam encompasses both, inflections and alterations—possibly under Western influence (Yöre, 2012).

^32^However, it does not defy the overall pan-harmonization of the PCs in a MPS, since it is minuscule, limited usually to just few degrees (most notably VII), and affects mainly vocals and string instruments.

^33^Every music theory defines a finite number of ways to modulate from one mode to another. Omnipresence of such regulation indicates that music-users remember the typical “moves” from mode to mode (Koelsch et al., 2003).

^34^Interval-class set (ICS) is the collection of intervals that characterize PCS. Interval-set class (ISC) is the ICS of an MPS mode. Hearing chromatic modulation requires knowledge of the repertory of ICSs and ISCs.

^35^Chinese chromatic temperament most likely grew out of the practice of combining composite pentatonic scales (Bagley, 2005).

^36^Ancient music afforded what we call “accidentals” (Wulstan, 1971). The term “kampe” might have implied pitch bending alien to “harmonia”—“*exharmonic*” melodic deviations unidentifiable by reference to the departed mode (Franklin, 2013). The earliest notation of them is in the Zeno Archive papyrus, 250 BC (West, 1992, p. 196).

^37^Hellenic culture pioneered a semiotic system of “musical emotions”—specific affective states conventionally ascribed to specific musical structures (Woodward, 2011). The outline of this system I present in my paper: https://www.researchgate.net/publication/291974302_How_Emotion_Can_Be_the_Meaning_of_a_Music_Work.

^38^Enharmonic music and modulations poorly suited lyre and kithara which remained the principal tools of teaching music in Greco-Roman education (Marrou, 1956, p. 134). Lack of precise instrumental reference (aulos could not support didactic descriptions of microtonal tuning due to its construction (West, 1992, p. 96) must have been the reason for Aristoxenus to exclude enharmonic modulations altogether from his exhaustive list of modulations (Hagel, 2009, p. 44).

^39^Widely used, “soft” and “hemiolic” gradations blurred the theoretic differences between chromatic and enharmonic intervallic divisions (153).

^40^See Part-1, Appendix-2 in Supplementary Material.

^41^Setting the precedence for association of low register with stability and relaxation.

^42^Hellenistic chromatic music set the direction for Western civilization by pioneering the notions of error [kheiron] (Losev, 2000, p. 623), error-correction [diorthosis] (Barker, 2004, p. 342) and compression, implied in the idea of “complexity” [poikolia] that meant artificiality of melody, harmony or rhythm/meter, which upset unaccustomed listeners (Franklin, 2013). Technically, this “complexity” involved stuffing more expressive information into the available compositional space, making perception of music burdensome.

^43^Just like the melodic tones appear to move in a particular way, the sequence of depicted objects suggests to a viewer a particular vector of scanning an image—by the interaction of its perceived shape, size, and centeredness.

^44^At times, perspective and music attracted the same artist: Leonardo was equally versed in painting and in composition (Mantero, 2001), and applied “musical proportions” in his “Last Supper” (Brachert, 1971).

^45^Presence of “accents” is best revealed when few ethnomusicologists transcribe a song, producing notations of the same performance, which nevertheless feature different spelling of pitches (List, 1974).

^46^It remains to be tested, how much representatives of different geographic regions differ in their “local accents” of tuning into the tonal space of a Western key - and what tuning range defines the degrees of tonality cross-culturally.

^47^Parncutt and Cohen (1995) consider 40 cents the threshold of perceptibility of steps in a scale.

^48^A typical common practice period polyphonic-homophonic texture subordinates all parts/voices to a one-at-a-time melodic line (with possible dubbing), where each of the textural components receives its own discrete function: principal melody; secondary melody (imitative/contrasting); accompanying melodic figuration; chordal/dyadic accompaniment; bass; pedal/background layer (Kholopova, 1979, p. 37). That still keeps in place about 4-5 textural components.

^49^Paleolithic paintings suggests much time and effort placed into artistic training: art historians regard many discovered images as “pupil's drawings” and “rough drafts” for “master-works” (Hauser, 1999, p. 9)—which would be hard to accomplish without receiving privileges relieving an artist from food-seeking.

^50^Customs observed in Aboriginal society confirm the exclusive nature of pictorial image production: drawing presents subjects, which are associated with dreaming, entering a world of spirits and requiring restricted access of only the ordained adult male members of the tribe.

^51^Sophisticated theory is required by the very task of 3D-to-2D shape conversion: Schäfer (1919, p. 254) describes a German peasant protesting against the crookedness of the roof in painter's sketching of his house—all to be surprised with the “magic” upon completion of the drawing, when the picture looked just right.

^52^Although certain types of music can promote greater pitch acuity: the frequency resolution of professional Western classical string ensemble players is significantly higher: the finest discrimination reaches 1 cent, whereas the grossest error—20 cents (Shackford, 1962).

^53^Jordan and Shepard (1987) disclosed such mechanism when they microtonally adjusted the major scale tones to stretch over an octave, or rounded up the intervals between the degrees—both distortions were overlooked by listeners.

^54^Although, the term “cent” comes from the well-tempered system, there is no another convention to refer to the micro-intervallic distances between the tones.

^54^Db has been tuned lower than C# in vocal/string performance since at least the 1800s (Barbieri and Mangsen, 1991), presenting different PCs. Listeners' perception of vocal intonation encourages singers to exaggerate sharpening/flattening (Geringer et al., 2015)—to increase expressiveness (Sundberg et al., 2013)—therefore, vocal intonation distinguishes between 17 PCs in a key, and not 12, as commonly believed.

^55^Thus, the Lithuanian folklore bears traces of 3 stages of tonal development: (1) the proto-Indo-European strict pentatony; (2) the later hexatonic development; (3) the Balto-Slavic heptatony, of probable Hellenic influence (Leisi, 2002).

^56^For more information, the fuller version of this paper will be available at academia.edu.

^57^Remarkably, the birth of perspectival depiction, chromatic music and mathematical 3-factor opposition all concur in Hellenic Greece—expressed in the arithmetic, geometric, and harmonic proportions of music: which bind two extremes by a single mean term (Lawlor, 1982, pp. 80–82).

^58^Also, unlike music, prosaic speech cannot generate the experience of physical motion in a listener (Dalla et al., 2013).

^59^Computer simulation confirms possibility for a repertoire of intonations to evolve “from scratch” by one music-user imitating the intonation of another with whom he wishes to bond—thereby creating a network of users interacting with one another (Miranda, 2002).

^60^Ambrazevičius (2004) demonstrates how the acoustic analysis of pitch distribution in the recordings of folk music can reveal modal organization characteristic of different epochs, from modern diatonic to ancient “squeezed anhemitonics.”
