# Supplementary material for: Evolution of Tonal Organization in Music Optimizes Neural Mechanisms in Symbolic Encoding of Perceptual Reality. Part-2: Ancient to Seventeenth Century
Source: Front Psychol. 2016 Mar 30;7:211. doi: 10.3389/fpsyg.2016.00211 (PMC4813086; doi:10.3389/fpsyg.2016.00211)
Supplement: Supplementary file 1 [file Presentation1.pdf]

Demonstration 1.
Chromaticism: folk “natural” microtonal modes and alterations.

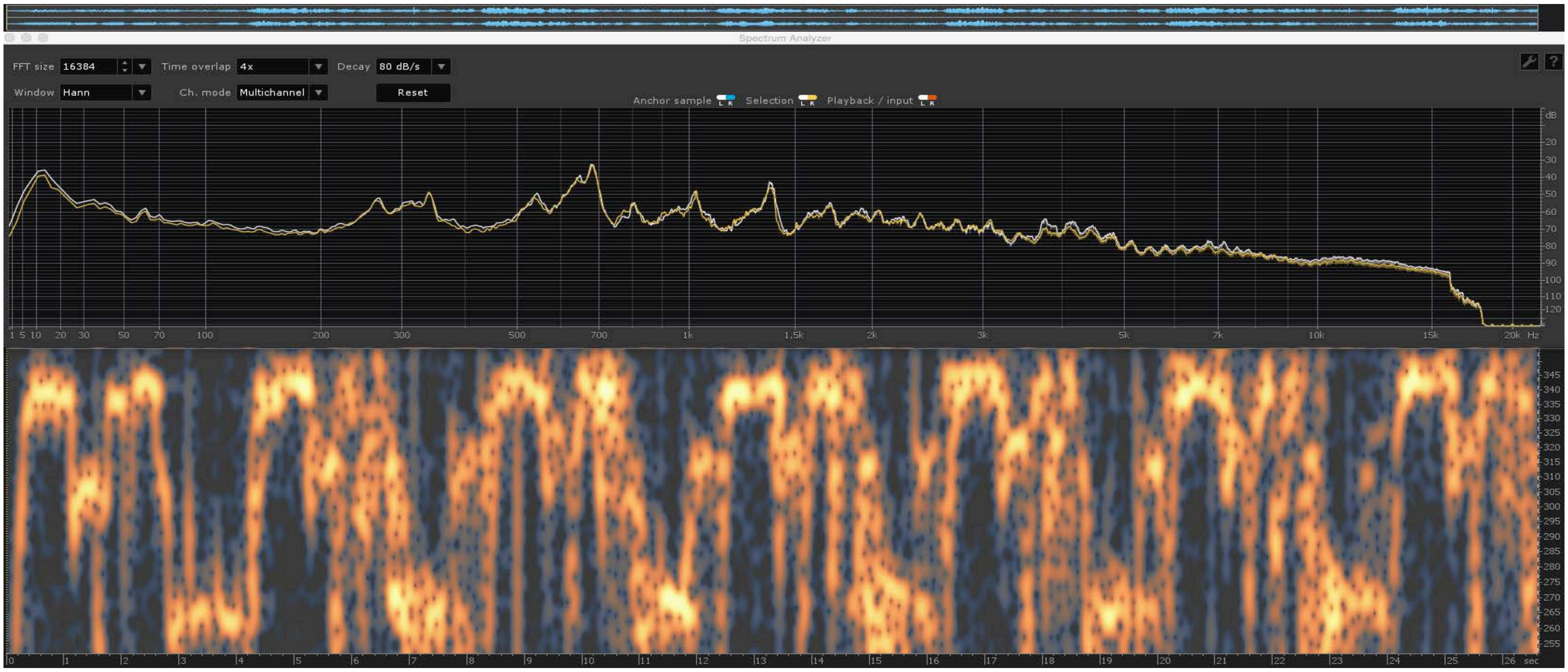

Press the "Play" button to audition the clip (available in Adobe Acrobat/Reader only).

(A) “Koliada” by n/a from the field recording of the choral village music in the Belgorod region, Russia, 1982, courtesy by Iraida Fedorova. Used by permission.

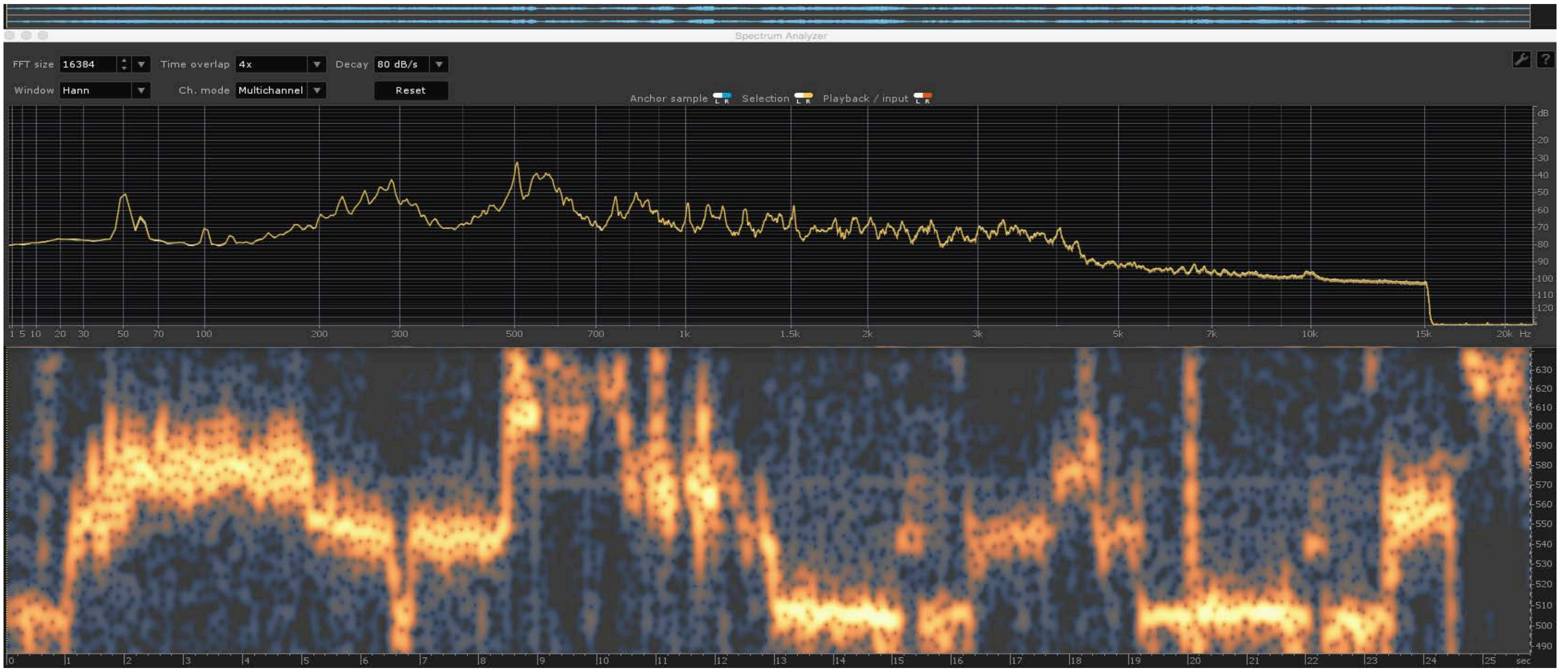

Press the "Play" button to audition the clip.

(B) "Montenegro: Epic Song" by n/a from the recording entitled Folk Music of Yugoslavia, FW04434, courtesy of Smithsonian Folkways Recordings. (p) (c) 1952. Used by permission

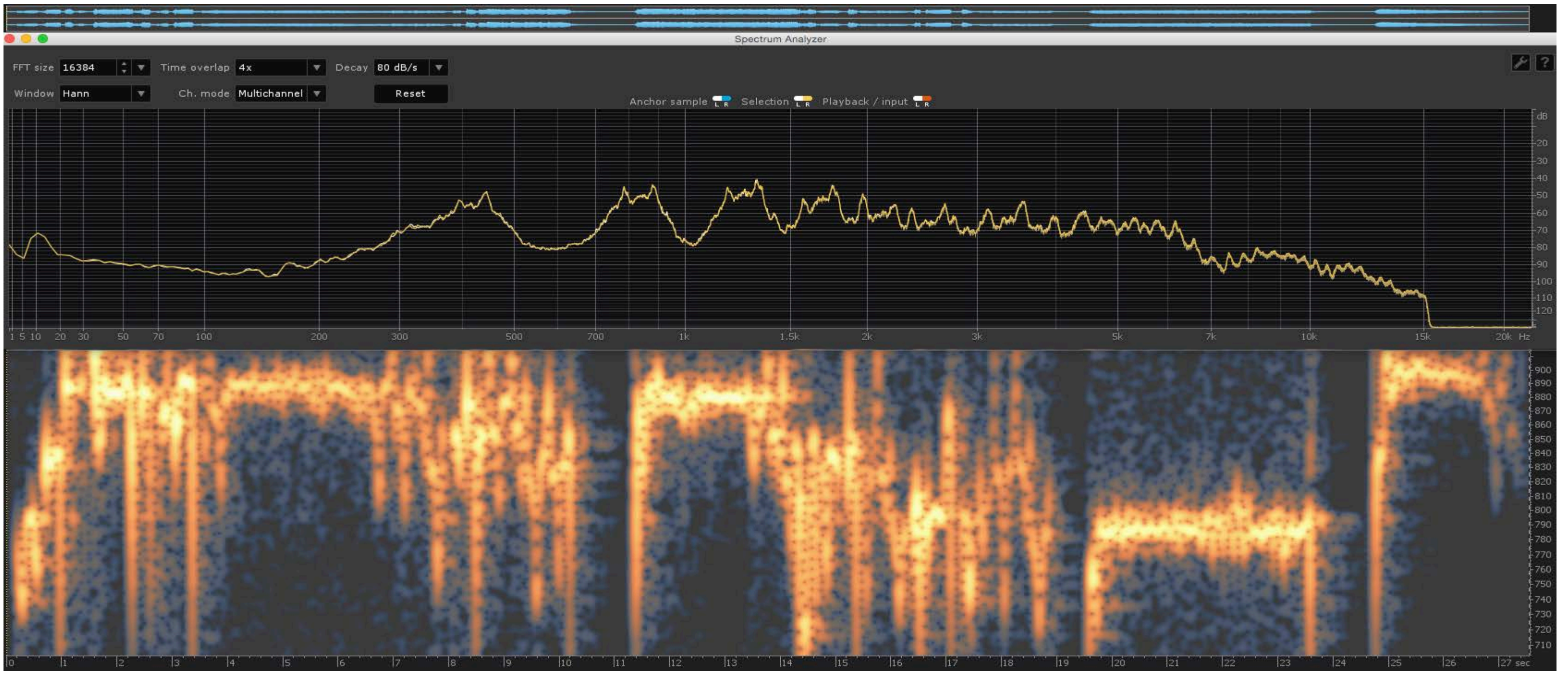

Press the "Play" button to audition the clip.

(C) “Falak-i Badakhshani" by The Badakhshan Ensemble from the recording entitled Music of Central Asia vol. 5: The Badakhshan Ensemble: Song and Dance from the Pamir Mountains, SFW40524, courtesy of Smithsonian Folkways Recordings. (p) (c) 2007. Used by permission.

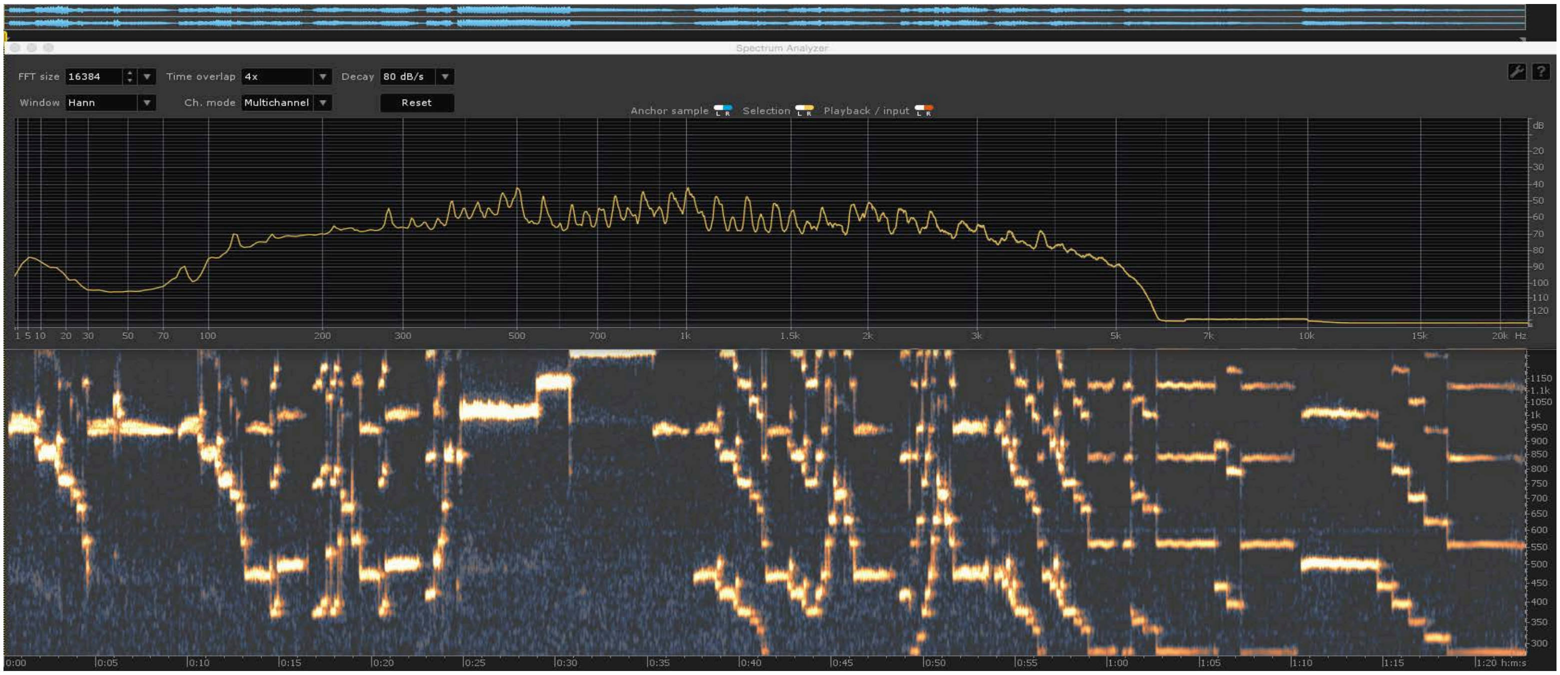

Press the "Play" button to audition the clip.

(D) Debussy – “Syrinx” for flute solo (1913) by Marcel Moyse, recorded in Paris in 1928, Jan.16. Public domain.

In this demonstration, four examples illustrate the notion of "naturalness" in non-diatonic use of semitones - which is conventionally qualified as "chromatic" in Western music theory. Examples **(A)** and **(B)** show modal organization without alterations, whereas examples **(C)** and **(D)** - with alterations. In case of example **(C)**, alterations are microtonal.

The sonogram (the low portion of the screenshot) shows the fundamental tone of the melodic line (occasionally, the firs partial tone is displayed above the fundamental, and can be recognized by its mirroring of the melodic line). The colors indicate the loudness of the audio: cyan for the softest, and yellow for the loudest levels. Numbers below the sonogram indicate time in seconds, and numbers on the right of the sonogram – the frequencies in hertz.

The spectrum analyzer (upper part of the screenshot) shows the average pitch values for the tones encountered throughout the entire audio clip. Each pitch value corresponds to the peak of the curves of the yellow line within a range between about 200 Hz and 900 Hz (1,200 Hz for the flute clip). The partial tones can be identified by their reproduction of the curvature shape of the fundamental tones.

(A) This is a South Russian *koleda* (Koliada) calendar song, part of an important winter ritual. Such songs usually feature the oligotonal mode based on a simple trichordic melodic formula (Zemtsovsky 1975; Krader and Zemtsovskii 1979). This genre is considered one of the oldest, related to archaic agricultural cults, constituting the most conservative layer in Slavic music, and is usually performed by a group of non-professional singers a'capella.

Here, is the list of the pitches in the order of their appearance in the audio clip:

E4+45(long), D#-30, E+33, F-35, D#-40->C+10, C+25, C+5.
E4+45(long), D#+13, F-22, [D+46/D#+25/F-30], D#+19, C+50, C+19, C+33, [C+16/D#+34].
F4-32(long), E-42, F-35, F-44=; [D#-31/F-49], [C-20/C#-38/D+38], C#-48, C#-46, D#+15.
E4+50(long), E-46, E+45, [D#-2/E+37], E+17, [C+40/D-10/D#+13], C#-40, [C-14/D#+24].
F4-25(long), E-48, [C-1/E+31], D#+45->F-35, [C-21/D#-41], C+20, C+27, D#+10.
F4-46(long), D#+20, E+44, [D+42/E+49], D#+19, [C+9/C#-45], [C+44/E-32].
F4-33(long), E-22, F-45, E+30, [C#-35/E+33]

Periods and line breaks indicate the end of a phrase. Numbers indicate the amount by which a pitch is greater or smaller than the standard pitch in the Western well temperament system, expressed in cents (1/100 of a semitone). Syllables are separated with commas. Arrow (->) marks the transition from one pitch to another over the same syllable. Square brackets indicate a sonance that functions as one entity. Slash (/) marks a cluster of pitches that constitute one sonance.

The list of pitches in the tempered scale can be used only as a starting point for the analysis of tonal organization. Nomination of tones as E, F, etc. is arbitrary, justified only for the purpose of comparative examination, and bears no connection to how performers categorize their pitches. Their categorization ought to be inferred by the analysis of:

- incremental distances between the discrete changes in pitch,
- correspondence of pitch value with the melodic direction
- position of pitch in the musical phrase (start, end, or middle)
- position of pitch in the syllable (start, end, or transition)

- articulated or portamento style production
- frequency of occurrence within the piece of music
- prevalence of specific combinations of pitches
- patterns of repetitions of specific motives
- melodic functionality of found patterns (i.e. auxiliary, acciaccatura)

Comparative analysis of these features allows to categorize the recorded pitches into degrees of a mode, and hypothesize the functionality of each of the degrees in terms of stability/instability ranking.

Thus, indications of E4+45 and F4-25 only superficially appear as different pitch values. In reality, their frequency is separated by meager 30 cents (a third of a semitone). Since the music structure of this song clearly features repetitions of the same formula, with minimal variations in tuning, the first tones that open the formula should be regarded as the same pitch class, and as a discrete degree in the mode. It is only that the mean value for tuning of this degree lies in between our conventional E and F.

Here is the list of all the pitches engaged in the fragment above:

C4 +10=; +25=, +5=; +50=; +19=; +33=; +16/=; -20/; +40/; -14/=; -1/; -21/; +20=; +27=; +9/; +44/=
C#4 -38/; -48=; -46=; -40=; -45/; -35/
D4 +46/; +38/; -10/; +42/
D#4 -30=; -40>; +13=; D#+25/; +19=; +34/=; -31/; +15=; -2/; +13/; +24/=; +45<; -41/; +10=; +20=; +19=
E4 +45(long)=; +33=; +45(long)=; -42=; +50(long)=; -46=; +45=; +37/; +17=; -48=; +31/; +44=; +49/; -32/=; -22=; +30=; +33/
F4 -35=; -22=; F-30/; -32(long)=; -35=; -49/; -25(long)=; -35=; -46(long)=; -33(long)=; -45=

The = sign indicates that the pitch terminates the syllable. The < sign indicates that the pitch proceeds up on the same syllable (on sing-out). The sign > indicates the descending sing-out. The / sign indicates that the pitch is presented simultaneously with other pitch(es) in a manner of a vertical interval. The sign /= indicates that such pitch terminates the phrase.

C#4 presents the variant of C4, because the lowest C# is just 2 cents higher than the highest C. All other C#s have low values. The range of the C degree is from -21 to +65. It ends a number of phrases and seems to execute the function of stable anchor, challenging the tonic. Its "contrary" function initiates mutability in this oligotonal mode.

D4 appears only in vertical harmony, which leads one to believe that it presents a "dirtily" intoned D#, since the music has a quite lively tempo, and most of the music is syllabic, with rather brief rhythmic duration of a syllable.

D#4 presents a wide range of values from -41 to +45.

E4 and **F4** are the variants of the same pitch class, as already explained above. Their range goes from E-48 to +75, being the widest, and exceeding a semitone. A big part of this must be the "dirty" heterophony in fast tempo, and accent placed on this tone (as revealed by the sonogram). Extra effort made must be responsible for the portamento "ins" and "outs" of this tone. Emphasis placed on the tone's duration and loudness, as well as its occasional appearance in one of the parts at the end of a phrase, mark it as "tonic."

This koliada's mode is trichordal, with 2 competing stable degrees and one unstable degree, complimentary to the "tonic." The mean value for the infrafix is C+22. The mean value for the unstable middle degree is D#+2. The mean value for the upper "tonic" is E+14. This mode might make an impression of containing alterations, but it remains "natural." All fluctuations in tuning are the inflections of degrees caused by intonation in a multipart setting. There are no systemic relations or patterns in sharpening or flattening a particular degree. Also, it is important to take into consideration that this music is completely "folkloric": it is performed by nonprofessionals on an all-inclusive basis, in the absence of any codified music theory. In such situation the margin of pitch error should be extended.

The increments between the I and II degrees are 280 cents, and 112 cents between the II and III degrees. The complimentary melodic function of D# towards E leads to the contraction of the increment between them, as opposed to the larger increment between the infrafix I degree, and the II degree that is functionally unrelated to it. The infrafix often ends up being "layered out," when one of the singers sustains it in the heterophonic texture, so that it forms a vertical interval together with D# or/and E. In this way, a melodic interval gets converted into a harmonic interval. This should be taken as yet another indication of the naturalness of the C-D#-E mode: altered degrees exercise the function of a "leading tone" towards an anchor, which implies the opposition of functions ("leading" as opposed to "stable"), and therefore should not comprise a vertical interval between these two tones. The presence of a vertical interval between two degrees testifies to the "naturalness" of these degrees.

The entire mode consists of 3 degrees, where the middle degree serves as a complimentary tone for the upper "tonic" – most obvious in the very beginning of a song, when it starts with a neutral tuning of the middle degree and only afterwards establishes the complimentary function.

Table 1: Tuning of the Degrees in the Koliada Mode.

Pitch Class	Pitch Range of a Degree	Mean Value	Upper Increment	Modal Function of a Degree
C4	86: -21 to +65	+22	280	Stable, infrafix, opposite function to E
D#4	86: -41 to +45	+2	112	Unstable, complimentary function to E
E4	123: -48 to +75	+14		Stable, "tonic"

Below, is the notation for the first 4 phrases of the audio clip, where each degree is represented by a dedicated line, from the lowest line for C4+22 to the highest line for E4+14:

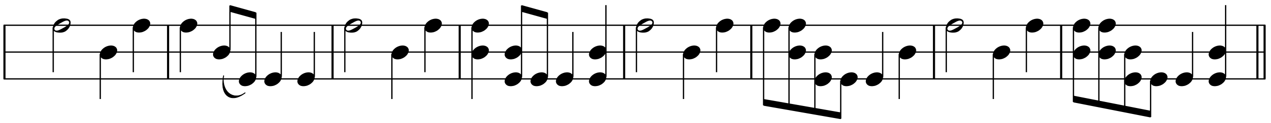

Fig.1. Tabulatura representation of the melody on the degrees of the Koliada mode. It is easy to see how horizontal intervals comprise the vertical intervals by "lagging" behind or "anticipating" a melodic intonation by one of the singers.

(B) Example No.2 is a heroic ballad from Montenegro, performed with the support of a *gusle* (bowed string instrument) that usually dubs the vocal line (Filipovic 1952). The epic poetry as a rule engages a formulaic structure, but this excerpt is an introduction executed in an improvisatory style. The music is sustained in a natural microtonal "chromatic" mode – where the word "chromatic" refers to the semitonal structure similar to the so-called "chromatic scale." Modes with numerous consecutive degrees separated by an interval close to a semitone are commonly found amongst numerous ethnic cultures of the mountainous areas of Balkans (Jordania 2006, 109).

Below, is the breakdown of pitches in phrases in the order of their appearance. Compared to (A), the phrases are significantly longer, and feature sing-out:

F3+41(gliss)->C#4-7->D-28(long)->C#-16->B3+33->C#4-34->D#+17, D#-14->F#-46->D#-28, D#-11->D+31->C#+46, D-6->D#+37->C#+43, D-11->C#+38->D#+38->C#+37, D-34->C#+28, C#+43->C+23->B3+43(long)->A3+30.
G#+33(gliss)->C+46->C#+32(long), D-3->F4-25->D-48->C#+5, [C#-13->C#+32]->C+41->B+29->A+45, D#+4->B3+41(long)->A#-49.
A-14(gliss.)->C4->C#+7(long), E4+6->D#+16->D+27>.

This is the list of all the occurrences of the pitches in the tempered scale and the inference of their modal pitch values:
F3 +41<
G#3 +33<
A3 +30=; +45=; -14<
A#3 -49=
B3 +33<; +43(long)>; +29>; +41(long)>
C4 +23>; +46<; +41>; 0<
C#4 -7<; -16>; -34<; +46=; +43=; +38<; +37=; +28=; +43>; +32(long)=; +5=; +22>; +7(long)=
D4 -28(long)>; +31>; -6<; 11>; -34>; -3<; -48>; +27>
D#4 +17=; -14<; -28=; -11<; +37>; +38>; +4>; +16>
E4 +6>
F4 -25>
F#4 -46>

The single occurrence of glissando from **F3** seems to constitute an accidental portamento device rather than a discrete modal degree. The same principle is engaged in the beginning of the 2nd phrase from **G#3**. Therefore, both pitches can be disregarded in modal analysis.

The lowest "meaningful" pitch is **A3** that once terminated the phrase, another time – terminated a syllable, and yet another time, started a phrase with portamento. A#3-49 should be regarded as the same PC as A3+45 because of its proximity in pitch and its terminating function for a phrase. That leaves the range of A+30 to +51 for the lowest degree, with the +40 mean value, marking it as stable.

B3 is the second degree, unstable, with a range between +29 and +43, mean +36. The prevailing functionality is to compliment the low A3.

C4 is another unstable degree, with mixed functionality: either as subordinate to B3 on the way down to A3, or as leading up to C#4. Its range is from 0 to +46, giving the mean value of +23.

C#4 is the most frequently used degree. Its range is the widest because of its functional richness: between -34 and +46. At times, it acts as a stable degree, most frequently terminating the syllables

and featuring long rhythmic values in two of such occasions. At other times, it is engaged in a descending or ascending motion. A wider tuning zone accounts for little sharpening or flattening in the corresponding intonations. The mean value for it is C#+6.

D4 is an unstable degree, complimentary to C#4. In most cases it leads down. Its range is within -48 and +31, with mean -8.

D#4 appears to be a relatively stable degree, twice terminating the syllables, and becoming balanced in descending and ascending uses. Its range is similar to D4: between D#-28 and +38, with the mean value at +5.

E4, **F4** and **F#4** all present the unstable degrees subordinate to D#4, with pronounced tendency to descend.

All in all, this mode features a clear cut microtonal organization of "natural chromatic" type, where each chromatic tone acts as a "natural" (non-altered) degree. The proof of this is the going over and leaping over the degrees while keeping their tuning (C#->B, C#->D#, D#-F#, D-F, F-C#, D#-B). Noticeable is the tetrachordal distribution of stability/instability (stable-unstable-unstable-stable) in the bottom part, and of even/odd (stable-unstable-stable-unstable) - in the kernel: A stable, B unstable, C unstable, C# stable, D unstable, D# stable, and E, F, and F# all unstable. That leaves A-C#-D# trichord as a consonance base for horizontal harmony.

The mutability between principal anchor C# and its infrafix A, seems to define the gravity in this mode. The 6 PCs C-C#-D-D#-E-F form the kernel of this mode, where A together with the complimenting B supports the kernel from underneath it, while F together with the complimenting F# provide extra auxiliary tones above the kernel. Such a 6-degree hemitonic mode is found in the neighboring Croatia (Marušić 2007) and was probably disseminated throughout the entire surrounding region at earlier times (Zlatić 1973).

As compared to **(A)**, the performance of **(B)** is less "democratic": not everyone can render epic ballads - they require considerable skills in singing, self-accompaniment, and versification. Instrumental support increases the precision of vocal intonation. Structurally, the tonal organization here presents a much greater complexity than in **(A)**, suggesting greater importance of pitch detail. Therefore, the discrepancies in pitch values here are to be paid greater attention to than in **(A)**.

The entire "chromatic" mode consists of 9 "natural" degrees, where the lowest degree is offset by a whole tone – to play into the "relaxing" paradigm of the low register, stemming from the Ancient Greek tradition (West 1992, 192) that is so vital in the Southern Balkan area (Katsanevaki 2011).

Table 2: Tuning of the Degrees in the Mode of the Montenegro Ballad.

Pitch Class	Pitch Range of a Degree	Mean Value	Upper Increment	Modal Function of a Degree
A3	21: +30 to +51	+40	196	Stable, infrafix, opposite function to C#
B3	14: +29 to +43	+36	87	Unstable, complimentary function to A

C4	46: 0 to +46	+23	83	Unstable, subordinate to B or leading to C#
C#4	80: -34 to +46	+6	86	Stable, "tonic"
D4	79: -48 to +31	-8	106	Unstable, complimentary to C#
D#4	66: -28 to +38	+5	101	Medium stable, free anchor
E4	n/a	+6	81	Unstable, complimentary to D#
F4	n/a	-25	79	Very unstable, subordinate to E
F#4	n/a	-46		Very unstable, subordinate to F

Below is the notation of the audio clip, which reflects the distribution of the melody in terms of the 9 degrees of a mode formulated above, from the lowest line, representing A3+40, to the upper line, representing F#4-46:

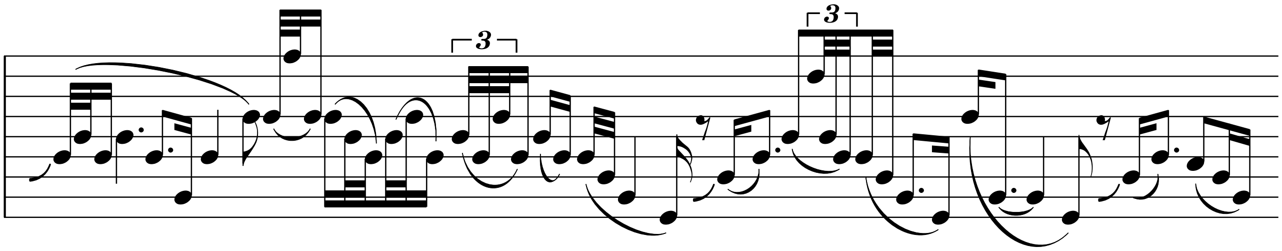

Fig.2. Tabulatura representation of the melody on the degrees of the Montenegro Ballad's mode. The stepwise intonations prevail. Relatively rare leaps occur in the beginning of a sing-out, accompanied with the short rhythmic values of the melismatic-like figures. Evident is the systemic character of the use of hemitonic degrees.

(C) Example No.3 is Falak from the Pamir region that is currently divided between Tajikistan and Afghanistan. Falak is a genre of professionalized traditional music, with highly virtuosic vocal technique, characterized by the subject of fatality. Falak is often included in the funeral rites, as well as in the performance of art songs of lyric poetry of tragic love content, usually sung to the Rubáiyát lyrics (Sakata 1983). Falak follows quite sophisticated orally transmitted music theory that branches from maqam (Azizi 2009), but gives much more prominence to microtonal organization (Galiakhmetova 2011).

Here, is the list of the pitches in the order of their appearance in the audio clip:

F4#+11->G-45, G#+3, A+17, A+17, G#+34->A+6, A-38, A-34->A+8, A-34->A+19->Bb-28->A-12->G#-13->A+16(long)->G#+17->A-17->Bb-21->A-15->G#-2->A-6->Bb-48->G#-3->G+5->G#+42, G#+21, A-10, A-47->Bb-4->A-50->G#-20>A-7->Bb->A-37>G#-20->F#+17->G#+20->A-17->G#-12->G+8->G#+39.
A4-27->G#+16->[A+9->A-16->A](long)->G#+22->A-11->Bb->A+9->G#+43->A-27->G+41->G#-49, F+46->G#-29, G#-7->G-21->G#+5, G#+31, G-20->F#+30->G#+25->A+22->G#-27->G+27->F#+8->G-2, G+43, G+29->A-34, G+16->F#+46, G+28->Bb-12->G#-18->G-40->G#-1->Bb-26->G#-14->G+33->F#-18->G+47.
F#-14->G+7(long)->Bb-47.
G#+17->A+34(long)->Bb-23->A-9->G#-16->A+22.

Below, is the list of all the occurrences of the pitches in the tempered scale and the inference of their modal pitch values:

F4 +46<
F#4 +11<; +17<; +30<; +8<; +46=; -18<; -14<
G4 -45=; +5<; +8<; +41<; -21<; -20>; +27>; -2=; +43=; +29<; +16>; +28<; -40<; +33>; +47=; +7(long)<
G#4 +3=; +34<; -13<; +17<; -2<; -3>; +42=; +21=; -20<; -20>; +20<; -12>; +39=; +16<; +22>; +43<; -49=; -29=; -7>; +5=; +31=; +25<; -27>; -18>; -1<; -14>; +17<; -16>
A4 +17=; +6=; -38=; -34<; +8=; -34<; +19<; -12>; +16(long)>; -17<; -15>; -6<; -10=; -47<; -7<; -37>; -17>; -27>; 0>(long); -11<; +9>; -27>; +22>; -34=; +34(long)<; -9>; +22=
Bb4 -28>; -21>; -48>; -4>; 0>; 0>; -12>; -26>; -47=; -23>

F4 is used only once in the function of “gliding in” towards the upper G#, not really well marked. Its starting pitch value is +46.

F#4 is similar in this supporting functionality: all its occurrences ascend, and only differ from F by the prevailing “complementary” function towards the upper G (or going over the degree to G#). F4 distinctly jumps over 2 degrees, and therefore should be regarded as the “exaggerated” version of F#4. Its pitch ranges between -18 to +60, with +21 as the mean value. G-45 and G-40 qualify as sharpened F#, because of their proximity to high F# and ascending directionality.

What is marked as **G4**, in fact, seems to contain 2 pitch classes: high G and low G. The margin between high and low Gs seem to go between the ascending +26 and descending +16. Then, the low G receives a range between -21 and +16, with the mean value of -3, and is directionally neutral (about equal in distribution of ascending and descending directions). The high G is more often directed up and gives +38 as the mean value.

G#4 and **A4** constitute the kernel of the mode: they are most frequently used within the sample of music which I provided. They also most frequently terminate the syllables. G#-49 stands out by 20 cents from the closest negative G#, and therefore qualifies as sharpened for ascending motion high G. Without it, the low G#s can be separated from high G#s at +5 (terminating the syllable, and is therefore neutral). Above it, all G#s are ascending, and this difference justifies the distinction between low and high versions.

Low G# serves as an anchor point for the ascending motion from high G. High G# bears a “complimentary” function towards A. The A-47 should be added to the high G# PC - making its mean value +34. The same applies to the instances of A-37 and -34: the former assists to the low G#, while both appearances of the latter assist to A, The mean value for low G# is -12.

A4 seems to comprise a single pitch class with a range from -27 to +22. Its mean value is -17. The long note, A+34, at the end of the sample, and the descending A+29, should be regarded as flattened **Bb4**. Most Bbs are descending and therefore functionally support A by providing a “complimentary” degree. Their mean value stands at -36.

So, overall, A4 appears to hold the entire mode by being the most frequently used pitch, and providing the end of a syllable more often than other degrees. Bb, F, and F# are complimentary degrees. Low G is more stable than high G, and low G# is more stable than high G#. This, and variability in tuning, leads one to think about high G and high G# as alterations rather than degrees, in their own right.

The increments between different versions of G and G# are the smallest and feature more pronounced directionality. High G and high G# are also overall shorter in rhythm. Harmonically, alternations within the kernel G-A generate the greatest tension. Therefore, it is plausible to recognize this song's mode as microtonal F#-G-G#-A-Bb with micro-chromatic alterations of G and G#.

Highly demanding technical skills make falak a professional genre. Its genesis from maqam which has a literate music theory, its use of instruments tuned according to mathematically supported standards, and its use of classical Persian poetry, make falak a very likely candidate to use chromatic alterations. Systemic violations of the prescribed by the music theory rules of tuning for each of the degrees in a "legitimate" mode charge melodic motion with increased tension that satisfies the need of falak singer to project tragic expression.

The condensed modal nucleus, where the "natural" degrees are separated by slightly more than a semitone, causes alterations to become mictrotonal, at a size smaller than an increment between the "natural" degrees - following the same model as alterations in diatonic MPS music.

Importantly, the tonal map inferred from acoustic measurements of the melodic intonations in this sample of falak substantially differs from the traditional (oral) music theory that has accompanied the historic development of falak: according to such oral music theory, falak is supposed to follow the maqamat tetrachord organization (Azizi 2009).

Concise intonational analysis of the tuning as executed by a computer software, however, demonstrates consistent use of microtonal gradations, with variety of melodic functions that range from auxiliary to opposing, involving leaps and leaped returns to the same degree. Evident from Fig.3, five hemitonic degrees are used consistently throughout music to provide predominantly stepwise melodic motion. Leaps are very scarce here, limited to the very beginning of a phrase, or to the function of a melodic "accent" in an embellishment.

The progression of 5 consecutive semitones, however, contradicts the codified music theory of maqam, which follows the diatonic principle of distributing degrees within a tetrachord base (Shumays 2013). It is very likely that the vibrato and appoggiatura embellishments that characterize maqam as a musical phenomenon, and are performed according to practice rules rather than theory rules (Yöre 2012) could have eventually produced a new scheme of modal organization, different from the original foundation of maqam.

This is yet another illustration of how the practical result in music-making can go quite far from the theory that nurtured that music – a common discrepancy in case of any music, notwithstanding folk music. It is possible that at some point falak's melody was ruled by anchoring on maqamat's tetrachords' tones. However, melismatic elaboration evidently took precedence in melodic organization, bringing in new functionality.

The mode consists of 5 degrees, two of which (G4 and G#4) afford alterations. The hemitonic structure of the steps of the mode makes these alterations microchromatic, with the pitch value about the size of a quarter-tone – smaller than the pitch value of the normative degrees.

Table 3: Tuning of the Degrees in the Mode of the Falak.

Pitch Class	Pitch Range of a Degree	Mean Value	Upper Increment	Modal Function of a Degree
F#4	78: -18 to +60	+21	76	Unstable, complimentary to G and G#
G4	37: -21 to +16<br>21: +26 to +47	-3<br>+38	41<br>50	Low G is medium stable, local anchor<br>High G is unstable, complimentary to low G#
G#4	34: -29 to +5<br>50: +16 to +66	-12<br>+41	53<br>62	Low G# is stable, opposite function to A<br>High G# is unstable, leading function to A
A4	61: -27 to +34	+3	79	Stable, tonic
Bb4	85: -47 to +38	-4		Unstable, complimentary to A

Below, is the notation of the beginning of this falak, where the lowest line represents the lowest F#4+21, and the highest line represents Bb4-4. Alteration of degrees is indicated through placing the note-heads in between the lines. As evident in the notation, the majority of occurrences of alteration constitute an auxiliary *melisma*: the melodic embellishment comprised by brief alternation of the principal tone and a tone closely above or below it - in a manner of a passage. Fast rhythmic figures mark such occurrences. The slurs indicate distribution of musical tones per syllable of lyrics.

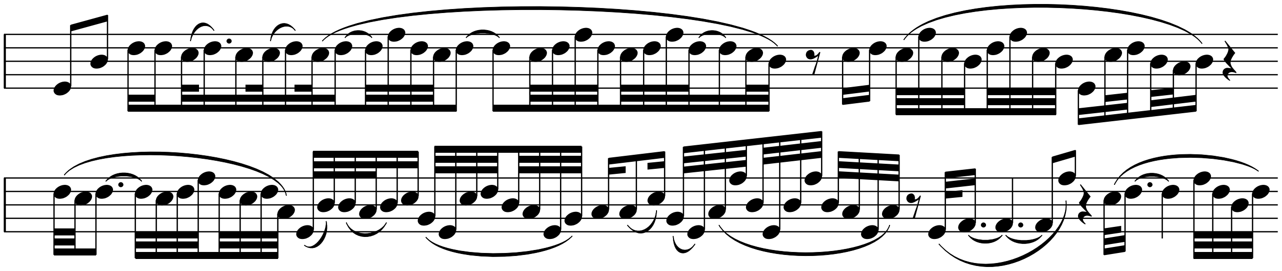

Fig.3. Tabulatura representation of the melody on the degrees of the Falak's mode. It is easy to see how microchromatic alterations consistently work to compliment the neighboring degrees by means of auxiliary tones, changing tones or escape tones melodic patterns.

(D) "Syrinx" by Debussy has attracted attention of numerous musicologists by its engagement of all 12 chromatic tones, despite the obvious presence of stability/instability in the tonal organization of this music (indicated by its composer through his notation that places 5 flats in the key signature – implying the key of Db Major or Bb Minor).

The tonal organization of Syrinx is usually defined as double-tonic Bb/Db with a special contribution of the whole-tone scale built on both of these tones (Cogan and Escot 1986, 92–101; Ewell 2005; Cobussen 2005). In the terminology that I put forth in my paper, this organization would be qualified as chromatic MPS with mutability of 2 anchors: Db and Bb.

The analysis of the exact tuning of the performance of "Syrinx" confirms this conclusion, and demonstrates the presence of MPS, which contributes to an organization distinctly different from that of folk uses of "chromaticism."

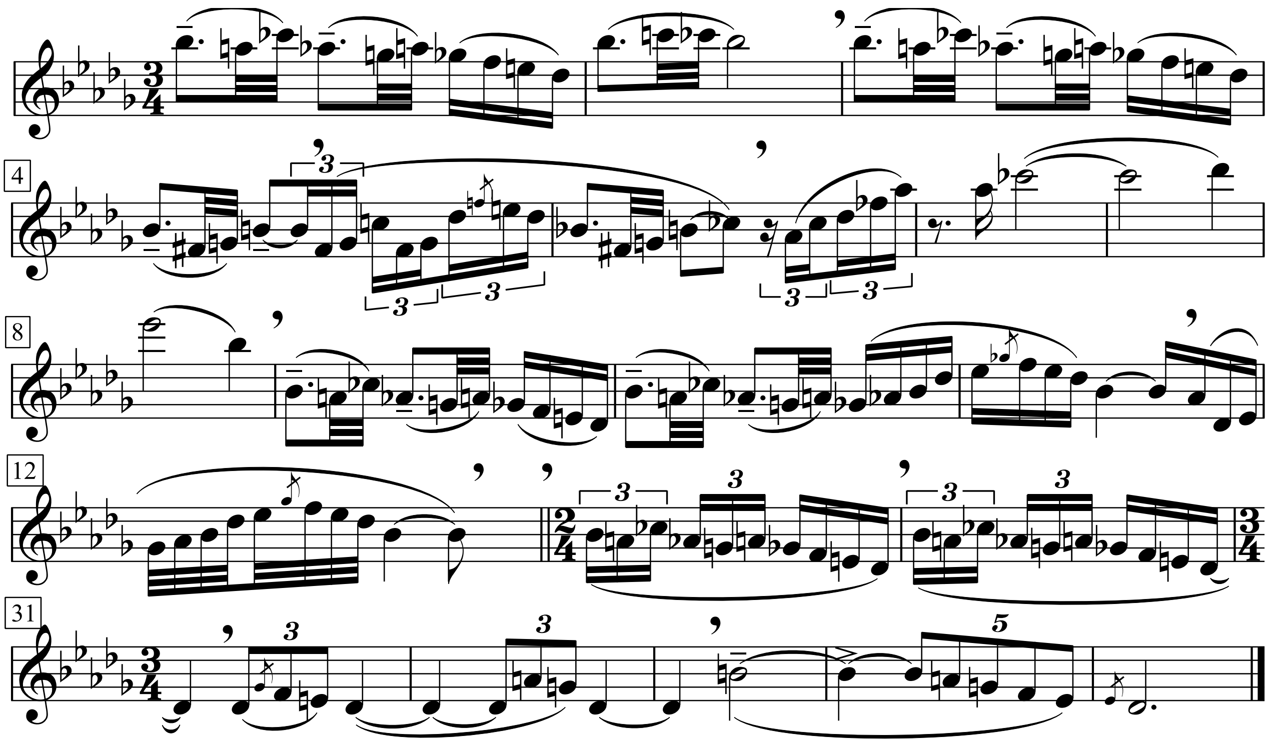

Fig.4. The notation of the music of the sound clip of Example No.4 (with the cut at the end of Bar No.12).

This clip features the performer who was personally entrusted by Debussy to edit his composition. The actual pauses, made by the performer, are reflected by periods. In order to facilitate comparison of this piece with **(A)**, **(B)**, and **(C)** examples above, I have cut off the middle section of "Syrinx" (Fig.4) together with the beginning of the recapitulation (respectively, bars 13-25, and 26-28, of the original score). This, I have done because according to the tradition of Western classical music, this piece features tonal development in its middle section (a very typical deviation toward the "subdominant key," in this case, Gb) – which has no analog in traditional folk music.

Exposition and conclusion sections of Western classical music, in contrary, usually feature the greatest tonal/modal coherence, and are therefore more comparable with the folk material. Since the purpose of this presentation is to demonstrate "chromatic" features in the music whose function is to "expose" a musical material rather than "develop" it, I found it permissible to limit the modal analysis of "Syrinx" to the non-developmental sections of its music form.

Here, is the list of the pitches in the order of their appearance in the audio clip:

Bb5(long)+41->A+34->B+26->Ab+26(long)->Ab-31->A+47->Gb+43->F+34->E+34->Db+40,
Bb5(long)+48->C6+28->B+29->Bb+10(long).
Bb5(long)+38->Bb-45>B+48->Ab+28(long)->Ab-31->A+46->Gb+35->F+33->E+32->Db+31->Bb4+25(long)->Gb+18->G+22->B+22.
Gb4+28->G+29->C5+37->Gb4+33->G+27->Db5+32->F5+44->E+28->Db+48->Bb4+21(long)->Gb+37->G+19->B+24(long).
Ab4+29->B+50->Db5+40->E+48-> Ab5+25, Ab+37->B+32(long)->Db6+18->Eb+44(long)-

>Bb5+31.
Bb4(long)+24->A+32->B+26->Ab+31(long)->G+50->A+27->Gb+24->F+22->E+9->Db4+31.
Bb4(long)+11->A+23->B->Ab+26(long)->G+34->A+24->Gb+26->Ab+29->Bb+24->Eb5+18->Gb5+31->F+47->Eb+29->Db+23-Bb4+18(long).
Ab4+23->Db4+26->Eb+22->Gb4+23->Ab+31->Bb+27->Db5+31->Eb+24->Gb+29->F+48->Eb+26->Db+26->Bb4+36 (long).
Bb4+23->A+37->B+38->Ab+37->G+27->A+13->Gb+35->F+27->E+11->Db4+11.
Bb4+18->A+18->B+36->Ab+39->G+39->A+26->Gb+27->F+14->E+22->Db4+19(long).
Db4+19->Gb4+7->F+20->E+27->Db+20(long)->A+5->G+17->Db+14(long).
B4+34(long)->A+8->G+14->F+7->Eb+10->Db+11(long).

The period breaks indicate the ends of the musical sentences, marked by the performer with pause. The -> sign reflects which tones are connected by "legato" articulation.

The list of all the occurrences of the pitches in this fragment, from low to high:

Db4 +31=; +26<; +11=; +19(long)=; +19<; +20(long)<; +14(long)=; +11(long)=
Eb4 +22<; +10>
E4 +9>; +11>; +22>; +27>
F4 +22>; +27>; +14>; +20>; +7>
Gb4 +18<; +28<; +33<; +37<; +24>; +26<; +23<; +35>; +26>; +7>
G4 +22<; +29<; +27<; +19<; +50<; +34<; +27<; +39<; +17>; +14>
Ab4 +19<;+31(long)>; +26(long)>; +29<; +23>; +31<; +37>; +39>
A4 +32<; +27>; +23<; +24>; A+37<; +13>; +18<; +26>; +5>; +8>
Bb4 +25(long)>; +21(long)>; +24(long)>; +17(long)>; +24<; Bb+18(long)=; +27<; +36(long)=; +23>; +18>
B4 +22=; +24(long)=; +50; +26>; +5>; +38>; +36>; +34(long)>
C5 +37>
Db5 +40=; +31>; +32<; +48>; +40<; +23>; +31<; +26>
Eb5 +18<; +29>; +24<; +26>
E5 +34>; +32>; +28>; +48<
F5 +34>; +33>; +44>; +47>; +48>
Gb5 +43>; +35>; +31>; +29>
Ab5 +26(long)>; -31(=G+69)<; +28(long)>; -30(=G+69)<; +25=; +37<
A5 +34<; +47>; +46>
Bb 5+41(long)->; +48(long)<; +10(long)=; +35(long)>; -45(=A+55)<; +31=
B5 +16>; +29>; +48>; +32(long)<
C6 +28>
Db6 +18
Eb6 +44(long)>

Here, the low **Db4** is clearly the "tonic", intended in this capacity by the composer (who notated the key signatures with 5 flats) as well as by emphasizing Db through the use of long notes and its placement at the end of phrases. The pitch range for Db is from +11 to +31, with the mean value +21.

Eb4 is unstable, from +10 to +22, with the mean value of +16. **E4** is also unstable, from +7 to 27, with the mean value of +17.

F4 appears to be more stable than the E and Gb surrounding it due to the use of Gb as the acciaccatura. F has a range of +7 to +27, with the mean value of +17.

Gb4 is unstable, usually leading to F or Ab. Its range is +7 to +35, with the average +21. **G4** is also unstable, usually working as an auxiliary tone for the Ab throughout most of the piece. The very

ending is the exception, where G proceeds into F (to stress the whole-tone pattern). G has a range from +14 to +50, with the mean of +17.

Ab4 is a relatively stable degree, marked by longer notes and by the complimentary function of the surrounding tones: G and A. Its range is +23 to +39, with the mean value of +31.

A4 is unstable like G, serving an auxiliary melodic function for Ab or Bb. The notable exception is the very end, where it proceeds to G (to comprise a whole tone progression). A has a range from +5 to +37, with the mean +21.

Bb4 is the second most stable anchor after Db. It often terminates the phrase and features long notes. Its range is: +17 to +36, with the mean +27.

B4 supports lower Bb with an exclusively descending function (except in bar 5, where it is notated as Cb, and appears more stable because of longer value and termination of the phrase). Its range is from 0 to +50, with the average about +25.

C5 is used only once, making an impression of an accidental (towards Db). Its value is +37.

Db5 is the octave equivalent of the low "tonic," and therefore enjoys a stable function, although in its register it never stays long. Its range is only +23 to +48, with the mean +35.

Eb5 is unstable like its low octave equivalent throughout the beginning and the concluding sections of the piece (it acquires a stable function in the middle section, omitted in my sequence). Its range is +18 to +29, with the mean about +23.

E5 is relatively more stable than E4, because of the acciaccatura function of F5 (bar 5). It is mostly directed down, ranged between +28 and +48, with the mean +38.

F5 alternates in its stability: it is stable in the conclusion because of the Gb5 acciaccatura use, although it seems to become unstable in bar 5 and the middle section that I omitted here. It is always directed down, indicative of its supporting role. Its mean value is between +34 and +48, with mean +41.

Gb5 is unstable, "complimenting" the lower F by always descending. Its range is +29 to +43, with mean value +36. **G5** is used twice in the opening two phrases (if to judge by their similarity with the rest of entries of the same melodic material in other parts of the piece - despite its tuning that is closer to Ab5). Its values should be taken as G+69. It should be regarded as unstable, auxiliary to Ab5.

Ab5 is a stable degree, marked by longer rhythm values and one occurrence which terminates the phrase. Its range is from +25 to +37, with mean +31.

A5 is unstable, executing a complimentary function to Bb5 or Ab5. It appears 4 times, the last of which is tuned slightly higher, closer to Bb - but should be considered as "A" because of its position in the principal theme, based on all of its repetitions. Its range is +34 to +55, giving about +44 as the mean value.

Bb5 is stable, featuring many long notes and few terminations of the phrases. Its range is +10 to +48, with +26 as the average.

B5 alternates from unstable to a more stable function: in the beginning it supports Bb5, but in bars 6-7, the long sustained tone makes it sound as an anchor tone. Its range is from +16 to +48, with mean value of +32.

C6 is used only once as an unstable tone resolving into Bb. +26. **Db6** is used once as a neutral tone. +16. **Eb6** is used once as an anchor. +44.

Below, the table lists all 20 degrees engaged throughout the music in the audio clip – disregarding octave equivalence. This is done with the purpose to identify functional discrepancies between the PCs in different octaves, as well as discrepancies in their tuning.

Table 4: Tuning of all the Degrees in the Syrinx mode.

Pitch Class	Pitch Range of a Degree	Mean Value	Upper Increment	Modal Function of a Degree
Db4	20: +11 to +31	+21	195	Stable, tonic
Eb4	12: +10 to +22	+16	101	Unstable, complimentary to Db or F
Fb4	20: +7 to +27	+17	100	Unstable, subordinate to Eb
F4	20: +7 to +27	+17	95	Relatively stable, complimentary to Db
Gb4<br>G4	28: +7 to +37<br>36: +14 to +50	+22<br>+32	105<br>98	Unstable, complimentary to F. G or Ab<br>Unstable, auxiliary to Ab
Ab4	20: +19 to +39	+29	92	Stable, opposite function to Db
A4	32: +5 to +37	+21	102	Unstable, complimentary to Ab or Bb
Bb4	19: +17 to +36	+27	98	Second stable, opposing function to Db4
Cb5<br>C5	50: 0 to +50<br>n/a	+25<br>+37	112<br>98	Very unstable, auxiliary to Bb4<br>Unstable, auxiliary to Db5
Db5	15: +23 to +38	+35	188	Stable
Eb5	11: +18 to +29	+23	115	Unstable
Fb5	20: +28 to +48	+38	103	Unstable, complimentary to Eb or Db
F5	14: +34 to +48	+41	95	Alternating between stable and unstable
Gb5<br>G5	14: +29 to +43<br>+69	+36<br>+69	133<br>62	Very unstable, auxiliary to F5<br>Very unstable, auxiliary to Ab5
Ab5	12: +25 to +37	+31	113	Stable
A5	21: +34 to +55	+44	82	Unstable, complimentary to Ab or Bb
Bb5	38: +10 to +48	+26	106	Stable
Cb6<br>C6	22: +16 to +48<br>n/a	+32<br>+28	96<br>90	Alternating between unstable to stable<br>Unstable
Db6	n/a	+18	226	Neutral

Eb6	n/a	+44		Stable

As the table reveals, most of the occurrences of the degrees are octave equivalent in tuning (within 14 cents difference) and in their functionality. Only 4 degrees are slightly off in relation to their different octave versions: Eb6 is 21 cents higher, Fb5 is 22 cents higher, F5 is 24 cents higher, and A5 is 23 cents higher – all are sharpened in the higher register. G5 features the greatest deviation: 35 cents higher. All these degrees are unstable, which should explain their deviation in tuning. Their instability is exaggerated through sharpening.

In classical music tradition, higher register is usually associated with greater tonal tension, which prompts stronger emphasis on "leading" intonations – the G5-Ab5 pair in the Syrinx mode presents such dedicated "leading" functionality. This explains the sharpest tuning of G5. Yet another confirmation of this explanation is the noticeable decrease of intervallic distance between G5 and Ab5, and between A5 and Bb5 – much more so in the higher than in the lower octave. Similar trend is evident between C6 and Db6.

On the whole, the mode contains 3 "leading" tones, G, C and A: G-Ab, C-Db, and A-Bb (in the order of intensity of the "leading" function, as it follows from tuning). These "leading" tones establish 3 modal anchor tones, which receive the biggest rhythmic and metric stress within the entire composition: Ab, Db, and Bb. Their functional relation is typical for classical Western tonality, as well as for folk multitonal mode (see part I of my paper).

- Db/Bb constitute the low VI-I degrees mutability, giving priority to the I degree (Db).
- Db/Ab mark the opposition of I-V degrees, where "dominant" (Ab) indicates supremacy of "tonic" (Db).
- Bb/F exposes yet another axis, where VI degree (Bb) has its own "dominant" (F), but that "dominant" receives significantly less weight than its counterpart Ab. Especially in the higher register, F is used as an unstable degree on number of occasions.

Another important prompt comes from the notation: Bb, Ab, and Cb are the only PCs that receive articulation markings: *tenuto* and *accent*. Cb plays a pivotal role in the modal mapping of "Syrinx." It executes the function of auxiliary tone in relation to Bb. Together with the "leading" A, it encodes the modal formula of the composition: *Bb-A-Cb* – which should be regarded as a peculiar embellishment of the "escape tone" type: unaccented auxiliary tone that is approached stepwise from an anchor tone and resolved by a skip in the opposite direction.

This Bb-A-Cb formula is marked out by the articulation through the slur throughout the score. Bb constitutes the anchor, followed by two altered auxiliary tones on its each side, on the way down toward the lower Ab, which acts as yet another anchor tone that reproduces the same formula.

Both "escape tone" formulas, together, mark the whole-tone harmonic relation: one for the anchors, and another – for the unstable tones. Here, Cb executes the function of the switcher: whenever it is necessary to alternate gravity from Bb to Db, it turns from Cb to B natural (actually spelled out in this enharmonic change in bar 5) – in order to "lead" into C, which in turn "leads" to Db.

This “leading” modal capacity of “B natural” is revealed at the very end of the composition, in the whole-tone descending progression from B natural all the way down to Db. The whole-tone scale takes the “unstable” row of the initial modal “escape tone” formula and brings it to its ultimate resolution without breaking the whole-tone pattern.

The “Syrinx mode” then reserves chromatic alterations for certain degrees, most of which remain octave equivalent and equalized in tuning, so that PCs do not shift in pitch values when the alternations between Db and Bb occur. In this capacity, the “Syrinx mode” contrasts the Falak mode from the previous example and illustrates the MPS style modal chromaticism.

Below, is the notation of the beginning of the “Syrinx” on an 8-line staff which represents 8 degrees of an octatonic mode, with 3 degrees that can be altered: Gb-G, Ab-A, and Cb-C. Of them, “G” and “C” always remain unstable. “A” can fluctuate from a stable state - when it acts as an anchor in the “orbit” of Db - to an unstable state - when it becomes the leading tone towards Bb, - anytime the gravity alternates towards Bb. In the reproduction of the “escape tone” formula from the Ab, this tone becomes enharmonically equated to “B double flat.”

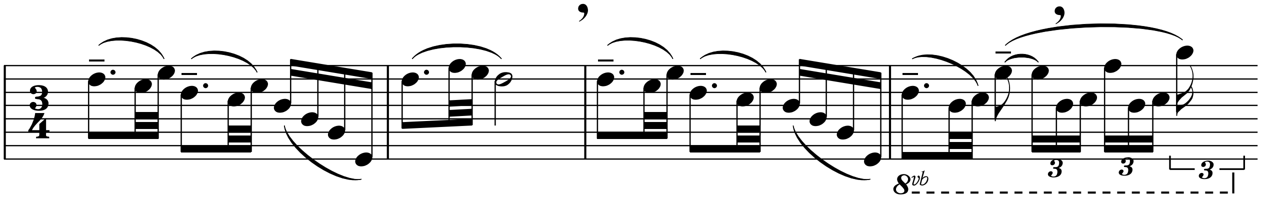

Fig.5. Tabulatura representation of the opening of “Syrinx” on the octave-equivalent degrees. It is easy to see how the “changing tones” pattern of the altered tones (the slurred group of 3 notes) generates the “escape tone” relationship to the following slurred group.

Three alterations are reserved for degrees with higher number – imitating the modal structure of the melodic minor mode, where the VI and VII degrees are subverted to sharpening and flattening, depending on the direction of the melodic line. This arrangement tends to produce tension in higher registers – evident in slight tuning discrepancies between the different octave versions of G and A, possibly influencing also F and E.

The bottom degrees remain more relaxed, especially the characteristic modal intonation F-Fb-Db, with Fb never directed up toward F. Such accumulation of tension in higher degrees, and relaxation of lower degrees, remind one of the non-octave hypermode (see Appendix II). Altogether, the contrast between normative and altered hemitonic degrees constitutes the peculiarity of the “Syrinx mode.”

As we see, the concept of alteration is relative to the intervallic typology employed in a given music system. There is no place for alteration in ekmelic modes that use unfixed indefinite pitches. Alteration requires definite pitch and presence of PSC.

In emmelic modes, alteration sharpens or flattens the normative pitch value for a given unstable degree, usually by about half of the smallest increment between two neighboring degrees in the mode. However, it is important to remember that mesotonal and multitonal modes can become “compressed,” and adopt a semitone as a normative “step” size between all or most degrees of the mode.

The resultant mode is better described as "microtonal": "micro" here refers to smaller than "normal" intervallic increments between the PCs within a PS. Professionalization of culture can institute "chromatic" alterations of natural semitonal degrees in such a mode, introducing genuine *microtonal accidentals*. Such alterations should be distinguished from the chromatic alterations within the diatonic MPS mode, as well as from the varieties of chromatic MPS mode.

REFERENCES:

Azizi, Farogat A. 2009. "Makom and Falak as the Phenomena of Professional Traditional Musical Art of the Tajiks [Маком и фалак как явления профессионального традиционного музыкального творчества таджиков]." Novosibirsk, Russia: Novosibirsk State Conservatory named after Glinka.

Cobussen, Marcel. 2005. "In(-)formations the Meaning of Paratextual Elements in Debussy's Syrinx." *Muzikološki Zbornik* 41: 55–70.

Cogan, Robert, and Pozzi Escot. 1986. *Sonic Design: Practice and Problems*. Publication Contact International.

Ewell, Laurel Astrid. 2005. "A Symbolist Melodrama: The Confluence of Poem and Music in Debussy's 'La Flûte de Pan.'" Morgantown, West Virginia: UMI Dissertation Services.

Filipovic, Milenko S. 1952. *Folk Music of Yugoslavia*. New York: Folkways Records. doi:FW04434 / FE 4434.

Galiakhmetova, Azaliya. 2011. "Makom and Falak - Genres of Traditional Music of Oral Transmission [Маком и Фалак – жанры традиционной музыки устной традиции]." *The Courier of the Moscow State University of Culture and Arts [Вестник МГУКИ]* 2: 217–21.

Jordania, Joseph. 2006. *Who Asked the First Question? The Origins of Human Choral Singing, Intelligence, Language and Speech*. *The Origins of Human Choral Singing, Intelligence, ...*. Tbilisi, Georgia: Logos.

Katsanevaki, Athena N. 2011. "Chromaticism: A Theoretical Construction or a Practical Transformation?" *Muzikologija: Casopis Muzikoloskog Instituta Srpske Akademije Nauka I Umetnosti* 11: 159–80. doi:10.2298/MUZ1111159K.

Krader, Barbara, and Izalii I. Zemtsovskii. 1979. "Melodika Kalendarnykh Pesen (The Melodics of Calendar Songs)." *Ethnomusicology* 23 (2): 346. doi:10.2307/851474.

Marušić, Dario. 2007. "Reception of Istrian Musical Traditions." *Музикологија: Часопис Музиколошког института Српске академије наука и уметности.* 7 (January): 185–98. http://www.doiserbia.nb.rs/img/doi/1450-9814/2007/1450-98140707185M.pdf.

Sakata, Hiromi Lorraine. 1983. *Music in the Mind: The Concepts of Music and Musician in Afghanistan*. Kent State University Press. https://books.google.com/books?id=nwfFsKew9FIC.

Shumays, Sami Abu. 2013. "Maqam Analysis: A Primer." *Music Theory Spectrum* 35 (2). Oxford University Press: 235–55. doi:10.1525/mts.2013.35.2.235.

West, Martin L. 1992. *Ancient Greek Music*. New York, London: Oxford University Press.

Yöre, Seyit. 2012. "Maqam in Music as a Concept, Scale and Phenomenon." *Journal of World of Turks* 4 (3): 267–86. EBSCO accessory # 84609213.

Zemtsovsky, Izaly. 1975. *The Melodics of Calendar Songs [Мелодика календарных песен]*. Leningrad: Muzyka [Музыка].

Zlatić, Slavko. 1973. "The Bugarenje in Northern Istria [Bugarenje Sjeverne Istre]." *Kaj: Časopis Za Književnost, Umjetnost I Kulturu* 6 (6): 50–54.
