# Supplementary material for: Evolution of Tonal Organization in Music Optimizes Neural Mechanisms in Symbolic Encoding of Perceptual Reality. Part-2: Ancient to Seventeenth Century
Source: Front Psychol. 2016 Mar 30;7:211. doi: 10.3389/fpsyg.2016.00211 (PMC4813086; doi:10.3389/fpsyg.2016.00211)
Supplement: Supplementary file 2 [file Presentation2.pdf]

Demonstration 2.
Chromaticism in the modern hemiolic mode.

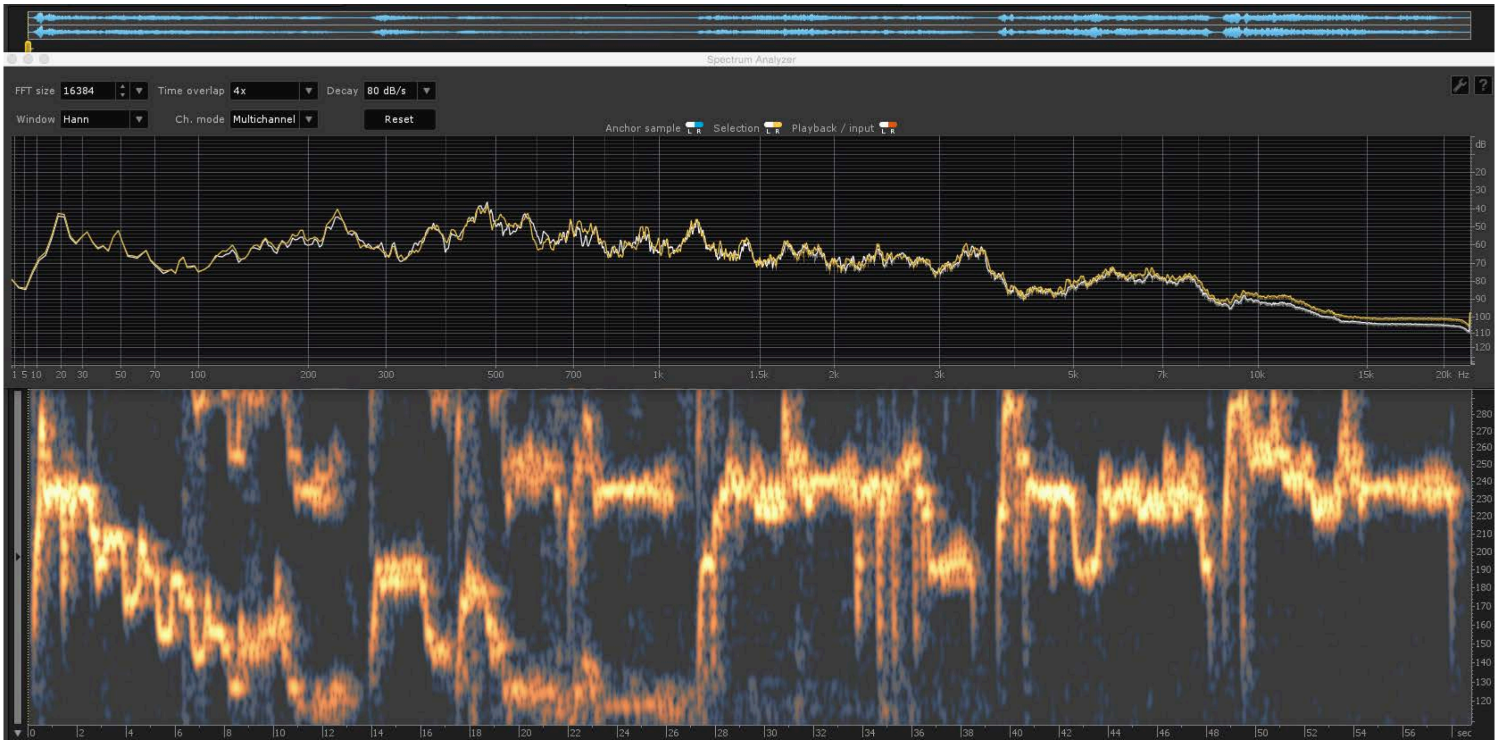

Press the "Play" button to audition the clip (available in Adobe Acrobat/Reader only).

Audio Ex.-1."B'utho According to St. Ephraim, 8th Mode (Thminoyo)" by Ado Abdelmasih, Aziz Gunel, Ibrahim Aksan from the recording entitled Syrian Orthodox Church: Tradition of Tur Abdin in Mesopotamia, UNES08075, courtesy of Smithsonian Folkways Recordings. (p)© 1973. Used by permission. Here, I have selected 4 complete musical sentences that represent all the characteristic modal intonations present in the entire track.

This demonstration is designed to show how the modern existing Near Eastern monody inherits the lineage from chromatic Ancient Greek music in its tonal organization. The supplication "B'hutho" ("Lord have mercy upon us") that I have chosen as an example for this analysis was composed by St. Ephraim (306-373), and was captured in the performance in the tradition of Tur Abdin by priests of the 5th century Church of St. Barsoum in Midyat, Turkey (Touma 1973). Its connection with the Ancient Greek system should not necessarily be taken as direct line of descent. Although the modal system for the Syriac chant, oktoechos, indeed seems to have Greek origins (Jeffery 2001), it is quite possible that in turn the modal organization of Ancient Greek music was influenced by the Syriac music culture, stemming from more ancient Assyrian culture, and appropriated by Greeks through Lydia (Franklin 2008).

In both scenarios, the modern day Syriac chant stands as the result of the evolution of tonal organization that proceeds from diatonic to chromatic MPS system, following the path of development of the Ancient Greek music from diatonic to chromatic system (West 1992). In fact, Ancient Greek employment of all 3 genera: diatonic, chromatic, and enharmonic can be regarded as the archetype of tonal organization for the entire Mediterranean region and adjacent geographic areas, and not only the property of Systema Teleion.

In 1883 the Patriarchal Musical Commission of the Ecumenical Patriarchate in Constantinople pronounced the diatonic, chromatic, and enharmonic genera of 8 modes to constitute the "pure form" of Christian tradition of music organization – as opposed to the later influence of Western tonality that "contaminated" (in the eyes of Orthodox theologians) Western Christian denominations (Lind 2012, 68).

In this Eastern tradition, each of the modes receives its favorable genus: *diatonic* genus is reserved for I, IV, V, and VIII echoi, *enharmonic* – for III and VII, and *chromatic* – for II and VI echoi (Scurtu and Tutu 2011). The biggest exception from the list of Orthodox churches that accept the chromatic and enharmonic genera is the Russian church - which the rest of Orthodox churches see as manifestation of Westernization of Russia, instituted by the church reform of Peter the Great. However, Russian ecclesiastic authorities explain their rejection of chromaticism differently: by their adherence to the principles of the Fathers of the Church who were explicit in their disapproval of any form of music but hymns and anthems in diatonic modes (Kutuzov 2008, 43–51).

Most historians agree that chromaticism of oktoechos is a later development, usually dated back to the 18th century (Wellesz 1954), and put it on the account of the influence from neighboring cultures cultivating maqam – through mediation by Turkey (Koço 2013). However, the hypothesis of Turkish import of chromaticism meets few objections. First, originally, chromatic music has not occupied much place in Turkish folk song, according to the earliest field-recordings (Hornbostel 1975, 1:91–114), and even in such cases where chromatic or microchromatic inflections do take place, they are clearly based on strong diatonic foundation (Belaiev 1935). Second, the earliest documented use of chromatic formula in Byzantine music comes from the 13th century (Tillyard 1935, 30–36) when Seljuks only started gaining political power. Third, there is evidence that kithara and aulos, the primary Hellenic "chromatic" instruments, were still in use in Byzantium by the 7th century, and the 10th century frescoes show musicians playing rabab-like bowed instruments, suggesting that the ban on instrumental music was not that strict and therefore did not serve to altogether exterminate chromatic music (Beaton 1980).

Ioannis Zannos believes that there was not any interruption in continuity of the cultivation of chromatic music once it was forged in the 5th century BC, and that as it was losing popularity in Western Europe, it was gaining popularity in the East. So, maqam should be viewed as an Eastern reincarnation of the Ancient Greek music (Zannos 1990). The tonal organization of music in Mashriq region can hardly be subdivided in ethnic types – ever since Antiquity, a variety of music traditions have been nurtured on the same soil fertilized by the Pythagorean tradition (Racy 2008).

Byzantine musical tradition is no exception: it came into being as a cultural compromise between the local tradition of Constantinople and Greek-speaking monasteries in Palestine – setting the framework of putting canonic lyrics onto music, which was subsequently adopted by all Medieval Christian denominations (Jeffery 1995, 6). As such, this tradition must have been democratic enough to absorb the modal intonations that circulated in the cultural environment: the Syriac and Coptic music cultures have remained folk, where psalms formed the basis of popular education - one of the first things taught to children (Taylor 2009).

It is highly doubtful if at the time common people were trained enough to tell by ear which intonation was "chromatic" and therefore unsuitable for a good Samaritan. It is much more

likely that all sorts of intonations stayed in use, but only the diatonic ones were exposed as exemplary for their ethical values, approved by the church authorities. Philippics of the Fathers of the Church, like Clement of Alexandria, directed against the chromatic music (Hermas et al. 2007), most likely had impact more on the musically educated clergy and cantors of the church rather than their congregations.

The sample of modern Syriac chant, analyzed here, seems to satisfy the criteria for its historic relation to Ancient Greek roots, while containing chromatic alterations and augmented 2nd between two neighboring degrees of its mode. The following analysis of the tuning of the degrees of this mode generally confirms the ethnomusicological reports of commonality of the combination of:

- characteristic modal intonation of the augmented 2nd,
- chromatic alterations, and
- microtonal inflections,

as traits in tonal organization of two traditional modes of Syrian Chant: Tminoyo and Shbi'oyo (Lundberg 1997).

The sonogram (lower portion of the screenshot) shows the fundamental tone of the melodic line (occasionally, the first partial tone is displayed above the fundamental tone, and can be recognized by its mirroring of the melodic line) within the range between 110 and 290 Hz. The colors indicate the loudness of the audio: cyan for the softest, and yellow for the loudest levels. Numbers below the sonogram indicate time in seconds, and numbers on the right of the sonogram – the frequencies in hertz.

The spectrum analyzer (upper part of the screenshot) shows the average pitch values for the tones encountered throughout the entire audio clip. Each pitch value corresponds to the peak of the curves of the yellow line.

Here, is the list of the pitches in the order of their appearance in the audio clip:

Db4-24, Bb+8, Bb-4, Bb-48, Bb+4, Bb-46, Ab+20, G-22, Ab-7->F-18->Ab-50->Gb+23->Eb+16->Gb-11->F-20->D-42->F-26->Eb+12->D+18->C-46->Eb-8->D-5(long)->Eb+18->E+38->Gb-16->Eb+3->D-38->C-47->Bb+6(long).
Gb3-17(long)->F-38->Eb-7->D-11, F+24->E-40->D+13->B2+44(long)->Bb-42, A-27, B+32, Db+15, B+47, Bb+18(long).
Gb3+50, G-25, A+25, Bb-11->B-46, Bb+12, A+12, Bb+20->B-27->Bb-12->A+28->Bb->A+11->Bb+3->Db4+2->C-34->B-42->C-31->B-32->Bb+35->Bb-33->B-48(long), G-11, Bb+13, Bb3+42, Bb+40, Bb+34, B+11, Bb+3, A+12, Gb+43(long).
A3+11->Db4+5, Db+27, C-50, Bb+35, Bb+2, Bb+3, Bb-20->Gb+37->B-31->Bb-25, A+14->Bb-8->Bb+47->Bb-13, A+21->Bb-20->A+24->B+11->Bb+5->A+13->Bb+2->B-17->Bb-11->A+19->G-30, Db4+39, D-5->C-25->D-7->C-35->B-38->B+23->B-43->Bb-42->C-17->D-4->C-29->B+25->Bb+28->Bb-23->Bb+25.

Periods and line breaks indicate the end of the phrase. Numbers indicate the amount by which a pitch is greater or smaller than the standard pitch in the Western well temperament system, expressed in cents (1/100 of a semitone). Syllables are separated with commas. Arrow (->) marks the transition from one pitch to another over the same syllable. Square brackets indicate a sonance that functions as one entity. The underlined pitches that share the same name exhibit clear auxiliary relation, and should therefore be regarded as different degrees.

The same procedure as the one implemented in Demonstration-1 is applied here. The list of all pitches will be ordered from the lowest to the highest, and marked as to the exact tuning and melodic circumstances of its appearance:

Here is the list of all the pitches engaged in the fragment above:

A2 -27=
Bb2 +6(long)=; -42=; +18(long)=
B2 +44(long)>; +32=; +47=
C3 -46<; -47>
Db3 +15=
D3 -42<; +18>; -5(long)<; -38>; -11=; +13>
Eb3 +16<; +12>; -8>; +18<; +3>; -7>
E3 +38<; -40>
F3 -18<; -20>; -26>; -38>; +24>
Gb3 +23>; -11>; -16>; -17(long)=; +50=; +43(long); +37<
G3 -22=; -25=; -11=; -30=
Ab3 +20=; -7>; -50>
A3 +25<; +12=; +28<; +11<; +12=; +11<; +21<; +24<; +13<; +19>
Bb3 +8=; -4=; -48=; +4=; -46=; -11<; +12=; +20<; -12>; 0>; +3<; [+35> -33]<; +13=; +42=; +40=; +34=; +3=; +35=; +2=; +3=; -20>; -25=; [-8<; +47>; -13]=; -20>; +25>; -11>; -42<; [+28< -27> +25]=
B3 -46=; 27>; -42<; -32>; -50(long)=; +11=; -31>; -45>; +28>; -17>; [-38< +23> -43]>; +25>
C4 -34>; -31>; -50=; -25<; -35>; -17<; -29>
Db4 -24=; +2>; +5=; +27=; +39=
D4 -5>; -7>; -4>

The = sign indicates that the pitch terminates the syllable. The < sign indicates that the pitch proceeds up on the same syllable (on sing-out). The sign > indicates a descending sing-out. The square brackets show the auxiliary embellishing patterns with pitches nominated by the same name, indicating that they should be regarded as different PCs.

A2 and **Bb2 -42** appear to share the same modal functionality, and should be regarded one unstable degree, complimentary to Bb2.

Bb2 is clearly featured as stable in the first couple of sentences, terminating both of them and containing two long notes. Also, its unusually narrow range for the low register (12 cents) testifies towards its stability.

B2 is melodically emphasized as a gapped degree in relation to D2. Because of this and its complimentary function to Bb2, B should be spelled out as **Cb**. Two occurrences of C3-46 and -47 should be regarded as higher versions of the same Cb degree.

Db3 occurs only once in the capacity of an auxiliary tone in relation to Cb3 (like Western “melodic minor” that comes to replace the “harmonic minor” in the ascending auxiliary motion). This narrow specialization suggests its auxiliary role as an alteration of a normative degree D3.

D3 plays the role of the “leading tone” toward the alternative “tonic” Eb3. Because of the secondary function of Eb3, as compared to the primary “tonic” Bb3, the increment separating the “leading” D is wider (140 cents) than increments of A2 (90) and especially A3 (69) which lead into Bb3.

Eb3 behaves as a "dominant" modal tone, challenging the Bb "tonic." Its secondary position is obvious from the fact that it never terminates any phrases - nor any syllables! Nevertheless, it clearly attracts a bunch of unstable tones: D3, Fb3, and F3.

E3 is better renamed as **Fb3**. In fact, it appears only once, passing from Eb3 in ascending motion toward Gb3. The E3-40 is engaged in the passing progression between F3 and D3, and therefore has to be respelled Eb. F3 seems to present an extra version of the same degree whose function is to supply the passing motion between two stable anchors: Eb and Gb. It is only that Fb specializes in passing in one direction, whereas F - in the opposite direction.

Gb3 works as a medium stable degree, never challenging Bb or Eb, but rather supporting the upper Bb - in a sense of complimenting its tonicity. It presents a characteristic "gapped" intonation in relation to upper A3 (most bright at the termination point of the 3rd sentence).

G3 forms an isle of critical tonal tension in the center of the modal kernel. It is just 63 cents away from the stable Gb, which is the shortest increment in the entire mode! In the absence of any resolutions of G3 into Gb3, we cannot accept either of them as discrete degrees. G should be regarded as the alteration of Gb. Thus, the passing progression Gb-G-A constitutes a characteristic modal intonation with a function to maximally sharpen the tonal tension by supporting the "leading tone" A for the entire mode. Yet, additional function of G3 is to provide an auxiliary motion for Ab. At one point G3 acts as an independent anchor point, when Cb4 leaps to it in order to leap up again to Bb4.

Ab3 works as an unstable anchor point, often housing the auxiliary intonation with support of the lower G3. In the first sentence it challenges Bb3 as a temporary anchor point in the descending motion. At times it becomes altered into A3 in order to provide an auxiliary melodic motion toward Bb3. The few low Bb3s should be regarded as sharpened A3s, because of their membership in a clearly auxiliary progression: i.e. Bb+28->Bb-27-> Bb+25 should be regarded as Bb-A-Bb. A is also important in providing the gapped intonation toward Gb3 (as in the end of the third sentence) and in providing the function of a leading tone to the "tonic" Bb3.

Bb3 is the most frequently used tone. Most of its uses terminate the syllable. The last sentence is terminated by it. I distinguish between the two octave versions of Bb, because they are not identical by the melodic function - which is quite common in the "Mediterranean tonality" (Manuel 1989). We already saw how the lower A2 is less of a leading tone than the higher A3. Bb3 also differs from Bb2 by being much more frequently used, but mostly with the shorter notes, and often arriving at Bb3 as a result of an ascending motion. Subsequently, Bb3 displays greater tonicity than Bb2, since its function of resolution is engaged to a much greater extent. Together, both Bbs outweigh Eb in their gravitational value in the entire mode.

B3 is another "disturber" in the modal kernel - like G3. At times it acts as an auxiliary tone to another unstable tone, C4. However, B3 cannot be considered as an alteration of Bb, because Bb is the "tonic," and that B3 is frequently involved into generating auxiliary and passing intonations toward Bb3 - which functionally divides them into different degrees. Because of much greater share of such intonations in the overall melody, as opposed to complimenting C, it is logical to spell B3 as **Cb4**. Higher Cb4 differs from the lower Cb3 by richer functionality and absence of gapped intonations.

C4 is octave inequivalent: there is no C3 involved in any characteristic intonations throughout the composition. This is not the only inequivalent degree in this mode: A2 and Ab3 form a "false relation," characteristic of hypermodes. Upper C4 is less unstable than Db4, D4, and Cb4. Occasionally, it carries an anchoring function with the help of B3. One of the instances of B3 has to be respelled into C4 because of the auxiliary functionality of the progression B3-38< B3+23> B3-43>, which implies B3+23 serving as flattened C4.

Db4 and **D4** present different versions of the same degree: Db is reserved to compliment Cb, while D - to compliment C. Alternation between these two paths of melodic embellishment constitutes much of the climactic elaborations in the highest register of this mode.

Table 1: Tuning of the Degrees in the B'utho Mode.

Pitch Class	Pitch Range of a Degree	Mean Value	Upper Increment	Modal Function of a Degree
A2	86: -27 to +58	+22	90	Unstable, complimentary to Bb2
Bb2	12: +6 to +18	+12	102	Stable
Cb3	22: +32 to +54	+14	201	Unstable anchor, gapped to D3
Db3<br>D3	n/a<br>55: -42 to +13	+15<br>-14	71<br>140	Very unstable, auxiliary to Cb3<br>Very unstable, leading into Eb3
Eb3	68: -8 to +60	+26	112	Stable, alternative to Bb
Fb3<br>F3	n/a<br>62: -38 to +24	+38<br>-7	55<br>136	Unstable, passing from Eb3 < to Gb3<br>Unstable, passing from Gb3> to Eb3
Gb3<br>G3	67: -17 to +50<br>19: -30 to -11	+17<br>-20	63<br>95	Medium stable, gapped, compliments Bb3<br>Unstable, passing to A3, or auxiliary to Ab3
Ab3<br>A3	70: -50 to +20<br>62: +11 to +73	-15<br>+42	157<br>73	Unstable anchor<br>Very unstable, auxiliary to Bb3
Bb3	70: -20 to +50	+15	68	Stable (tonic)
Cb4	56: -45 to +11	-17	74	Very unstable, auxiliary to Bb3 or C4
C4	60: -77 to -17	-47	61	Unstable anchor
Db4<br>D4	63: -24 to +39<br>3: -7 to -4	+8<br>-5	87	Unstable, complimentary to Cb4<br>Unstable, complimentary to C4

Representation of the entire clip in terms of these degrees would render the following notation, where each line of the staff indicates a particular degree, from the lowest A2 to the highest D4.

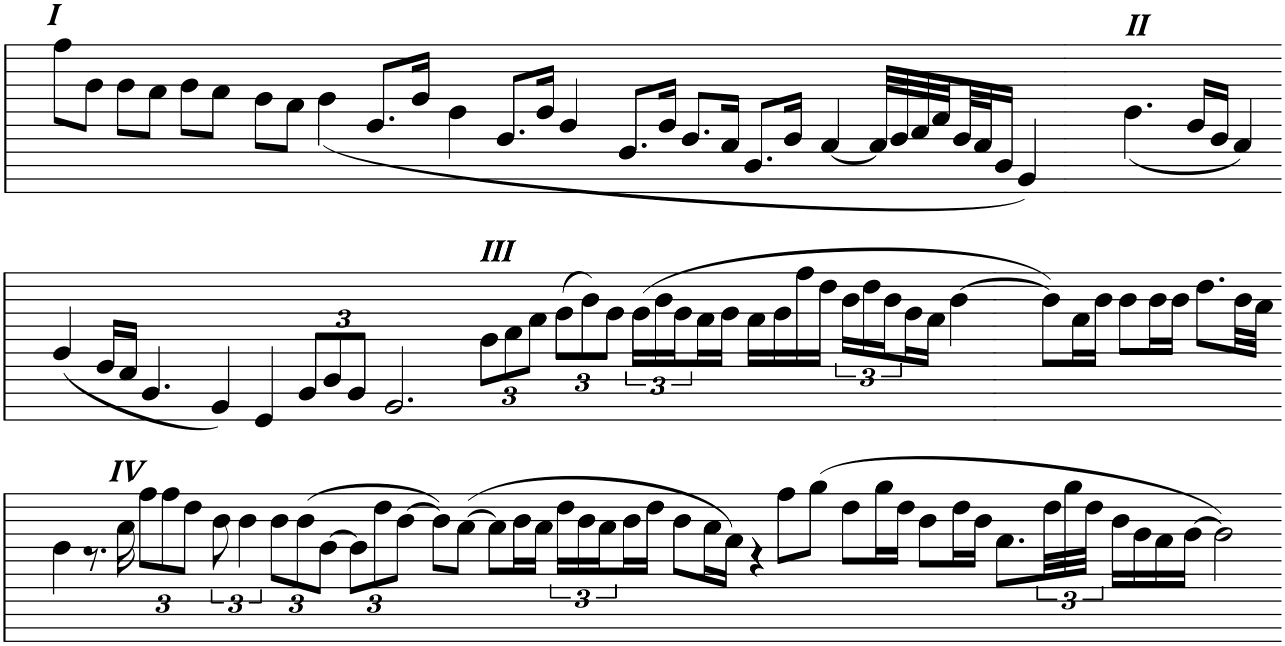

Fig.1. Tabulatura representation of the melody on the degrees of the B'utho mode. Note-heads placed between the lines indicate alterations. The sentences are marked with the Roman numerals. The slurs reflect sing-outs.

The overall disposition of the degrees formulated in the table above seems to comply with the intervallic relations formed within the melodic line, as it is evident from the Fig.1.

This mode is clearly chromatic, based on tetrachordal subsets, and features complex hierarchic structure implemented around the framework of 4 stable degrees. Of them, Bb acts as the most stable tone, Eb – as the second most stable tone, at times challenging the superiority of Bb, and Gb as a complimenting anchor to Bb.

Unstable degrees also feature subordination as well as coordination. Thus, C acts as the least unstable degree, whereas A as the most unstable degree. Each tetrachordal subset possesses its own anchor, and they are all related to one another, forming subordinative ranks.

The functions of degrees are not completely octave equivalent. Especially the presence of C in the upper register only, and the false relation between low A and high Ab resemble modal organization in non-octave hypermode (see Appendix-2). This is not surprising, if to remember that the hypermodal organization is also typical in the Orthodox chant, and stems from the same Ancient Greek source as does the chromatic system.

The similarity of the B'utho mode to the upper part of the Ancient Greek Systema Metabolon in chromatic genus is stunning. The nucleus of the "B'utho mode", comprised by its upper subsets (from F3 to D4) exactly matches the intervallic structure of the tetrachord Synemmenón and the tetrachord Mesôn. All the degrees perfectly coincide. The only significant difference is their gravitational map: see Fig.2.

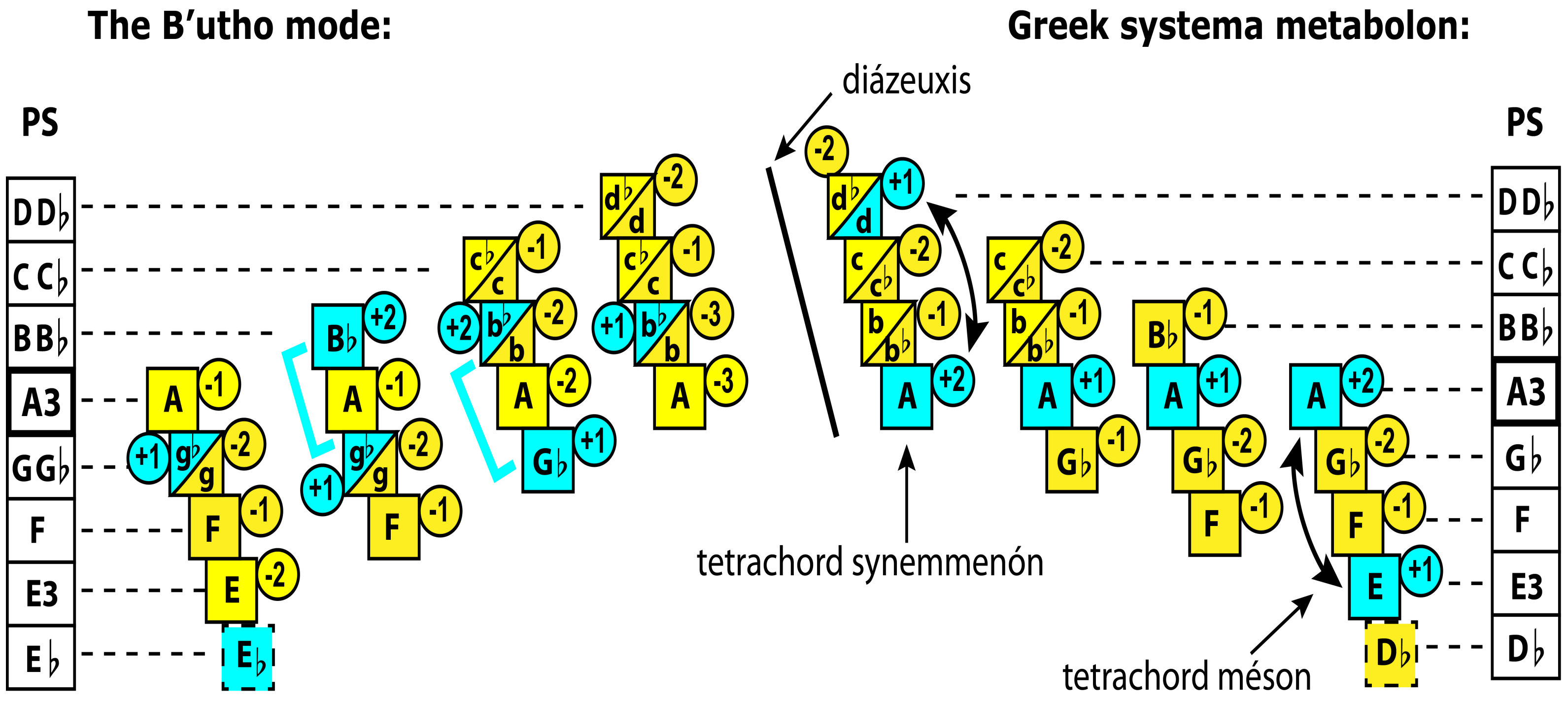

Fig.2. Comparison of the tonal organization in the B'utho mode and the Greek *Systema Metabolon* (West 1992, 223). The blue color indicates stable degrees, while the yellow color – unstable degrees. The numbers in the circles show gravitational grading: greater positive number means more stable degree, greater negative number – more unstable degree. Those degrees that have different versions (alterations) are split diagonally. The columns on the sides demonstrate that the pitch sets for the B'utho mode and the *Systema Metabolon*.

Evidently, the pitch organization is the same for the upper 6 PCs. The differences start from E3 (Fb3) downward. The B'utho mode reproduces the middle tetrachord below the nucleus in disjunct manner: the tetrachord Bb-Cb-D-Eb basically reproduces the tetrachord F-Gb-A-Bb – with the only exception of the absent intermediary degree - low C (that would be functionally equivalent to G in the middle tetrachord). In *Systema Metabolon* the lower tetrachords are connected in conjunct manner, producing different pitch subset (C-Db-E-F).

The difference between the B'utho mode and the two upper tetrachords of Systema Metabolon is most pronounced in the distribution of gravity.

- Ancient Greek system stresses A3 (*Mesa*) as the "tonic," reserving E3 (*Hypate*) and D4 (*Néte)* for the alternative anchors, subordinate to the "tonic." This creates modal mutability (reflected on the Fig.2 above, by the curved arrows).
- The B'utho mode, on the other hand, places the center of gravity on Bb3, and holds A3 as a very important tone – a "leading tone" to the tonic. It receives the supporting stable tone Gb, which never challenges Bb. Their complimentary relation is reflected by the blue bracket.

Subsequently, the B'utho mode is slightly more centripetal than the Ancient Greek system. It is also more hierarchic: its highest tetrachord features 3 gradations of instability. Characteristically, the tension in the B'utho mode is the greatest at the highest point of the ambitus.

Such close correspondence, despite greater tension and greater tonicity of the B'utho mode, cannot but lead to the conclusion that it presents a later historic development of the same "chromatic" prototype. This, in turn, suggests the organic intra-relation between the modern hemiolic modes of "Mediterranean tonality" and the Ancient Greek chromatic MPS system.

It seems that intonational analysis of frequencies of the recorded music work is capable of resolving many problems currently faced by researchers of ethnic music. The biggest problem is that musicologists have traditionally zoomed into scales in their analysis of cultural similarities between different types of music, and pay little attention to typology of melodic intonations – which results in numerous false attributions that are still widely circulating in the ethnomusicological literature (Pennanen 2008). Even the music composed in relatively strict compliance to the rules of the documented music theory, in fact, demonstrates discrepancies in its tonal organization from the postulated rules – including such relatively "well-disciplined" culture as maqam (Bozkurt et al. 2009).

The clash between theory and practice has been known in every music culture that has a documented music theory. Very few musicians make music by treatises. Most musicians make music by ear. Thus, Turkish music theory utilizes division of octave into 24 microtones (some theorists break it even in smaller increments), but in reality Turkish musicians compose music not by summing microtones together but by stitching together familiar diatonic modes and then using expressive tuning to emphasize certain intonations (Belaiev 1935). The same seems to take place in the maqam music created by Egyptian and Syrian musicians (Shumays 2009).

Syriac chant makes no exception. The apparent contradiction between its diatonic framework outlined in the music theory of oktoechos and the microtonal practice observed in almost all modern performances (Jarjour 2015) is in reality just two sides of the same coin – the principle of tonal organization that re-enacts the Ancient Greek trichotomy of *harmonia.* Each tetrachord affords chromatic and microchromatic modifications of the underlying diatonic framework.

REFERENCES:

Beaton, Roderick. 1980. "Modes and Roads: Factors of Change and Continuity in Greek Musical Tradition." *The Annual of the British School at Athens* 125: 1–11.

Belaiev, Victor. 1935. "Turkish Music." *The Musical Quarterly* 21 (3): 356–67.

Bozkurt, Barış, Ozan Yarman, M. Kemal Karaosmanoğlu, and Can Akkoç. 2009. "Weighing Diverse Theoretical Models on Turkish Maqam Music Against Pitch Measurements: A Comparison of Peaks Automatically Derived from Frequency Histograms with Proposed Scale Tones." *Journal of New Music Research* 38 (1): 45–70. doi:10.1080/09298210903147673.

Franklin, John Curtis. 2008. "'A Feast of Music': The Greco-Lydian Musical Movement on the Assyrian Periphery." In *Anatolian Interfaces: Hittites, Greeks and Their Neighbors*, edited by Billie Jean Collins, Mary R. Bachvarova, and Ian C. Rutherford, 193–203. Oxford UK: Oxbow Books.

Hermas, Tatian, Theophilus, Athenago, and Clement. 2007. *The Ante-Nicene Fathers: The Writings of the Fathers Down to A. D. 325*. Edited by Alexander Roberts. Vol. 2. New York: Cosimo, Inc.

Hornbostel, Erich M. von. 1975. *Opera Omnia*. Edited by Klaus P. Wachsmann, Dieter Christensen, and Hans-Peter Reinecke. Vol. 1. The Hague, Netherlands: Martinus Nijhoff.

Jarjour, Tala. 2015. “Syriac Chant at the Negotiation of Source and Method in the Two Music–‘ologies.’” *Yearbook for Traditional Music* 47: 45. doi:10.5921/yeartradmusi.47.2015.0045.

Jeffery, Peter. 1995. *Re-Envisioning Past Musical Cultures: Ethnomusicology in the Study of Gregorian Chant*. Chicago, IL: University of Chicago Press.

———. 2001. “The Earliest Oktōēchoi: The Role of Jerusalem and Palestine in the Beginnings of Modal Ordering.” In *The Study of Medieval Chant: Paths and Bridges, East and West: In Honor of Kenneth Levy*, edited by Peter Jeffery, 147–210. Woodbridge, Suffolk, UK: Boydell & Brewer Ltd.

Koço, Eno. 2013. “Byzantine Chant, the Ison, and Arbëresh Liturgical Chant.” In *Local and Global Understandings of Creativities: Byzantine Chant, the Ison, and Arbëresh Liturgical Chant*, 266–81. Cambridge, UK: Cambridge Scholars Publishing.

Kutuzov, Boris P. 2008. *Russian Znamennyi Chant [Русское знаменное пение]*. Moscow: Andrei Rublev [Андрей Рублев].

Lind, Tore Tvarnø. 2012. *The Past Is Always Present: The Revival of the Byzantine Musical Tradition at Mount Athos*. Lanham, MD: Scarecrow Press. https://books.google.com/books?id=VPWjSeLibr4C.

Lundberg, Dan. 1997. “Welcome to Assyria: Your Land on the Cyber space—Music and the Internet in the Establishment of a Transnational Assyrian Identity.” *Etnomusikologian Vuosikirja* 10: 13–28.

Manuel, Peter. 1989. “Modal Harmony in Andalusian, Eastern European, and Turkish Syncretic Musics.” *Yearbook for Traditional Music* 21: 70–94. doi:10.2307/767769.

Pennanen, Risto. 2008. “Lost in Scales: Balkan Folk Music Research and the Ottoman Legacy.” *Muzikologija*, no. 8: 127–47. doi:10.2298/MUZ0808127P.

Racy, A. J. 2008. “Overview of Music in the Mashriq.” In *The Concise Garland Encyclopedia of World Music: The Middle East, South Asia, East Asia, Southeast Asia*, edited by Ellen Koskoff, 2:810–22. New York: Routledge.

Scurtu, Bogdan, and C. Tutu. 2011. “Romanian Ochtoechoi and Similarities to Middle Eastern Modes and Practices: A Case Study (part I).” *Scientific Bulletin of the Transilvania University of Brasov* 4 (53/2): 89–98.

Shumays, Sami Abu. 2009. “The Fuzzy Boundaries of Intonation in Maqam: Cognitive and Linguistic Approaches.” In *Conference of the Society for Ethnomusicology; 2009 Annual Meeting (54th): Mexico City, Mexico, November 19-22, 2009*, edited by Brenda M. Romero. Indianapolis: Indiana University Press. http://maqamlessons.com/analysis/media/FuzzyBoundaries_MaqamIntonation2009.pdf.

Taylor, David. 2009. “The Psalm Commentary of Daniel of Salah and the Formation of Sixth-Century Syrian Orthodox Identity.” In *Religious Origins of Nations? The Christian Communities of the Middle East*, edited by B. ter Haar Romeny, 89:65–92. Leiden, The Netherlands: Brill Academic Pub.

Tillyard, Henry Julius Wetenhall. 1935. *Handbook of the Middle Byzantine musical notation*. Copenhagen: Levin & Munksgaard.

Touma, Habib Hassan. 1973. *Syrian Orthodox Church: Tradition of Tur Abdin in Mesopotamia*. Paris: Audivis-UNESCO. doi:UNES08075 102.

Wellesz, Egon. 1954. “Byzantine Music and Its Place in the Liturgy.” *Proceedings of the Royal Musical Association* 81st Sessi (1954-55): 21–38. http://www.jstor.org/stable/766125.

West, Martin L. 1992. *Ancient Greek Music*. New York, London: Oxford University Press.

Zannos, I. 1990. “Intonation in Theory and Practice of Greek and Turkish Music.” *Yearbook for Traditional Music* 22 (1990): 42–59.
