# Supplementary material for: Evolution of Tonal Organization in Music Optimizes Neural Mechanisms in Symbolic Encoding of Perceptual Reality. Part-2: Ancient to Seventeenth Century
Source: Front Psychol. 2016 Mar 30;7:211. doi: 10.3389/fpsyg.2016.00211 (PMC4813086; doi:10.3389/fpsyg.2016.00211)
Supplement: Supplementary file 3 [file DataSheet1.zip › Appendices I-VIII/Appendix I. Opposition of Folk and Palace-Temple Music in Antiquity.docx]

# Appendix-I: Opposition of Folk and Palace/Temple Music in Antiquity

The following is a summary of available evidence collected from multiple sources to support the idea that multimodal diatonic organization of Mesopotamian music that emerged in urban culture at the end of the 3^rd^ millennium BC stood in opposition to the previously existing heptatonic and pentatonic schemes, cultivated in folk village culture. The new temple/palace music could have initially utilized the musical material of the local folk traditions, reorganizing them by means of a new mathematically verified music theory – but eventually developed into completely new method of music composition, strongly contrasting the earlier folk methods.

We know from Athenaeus, Pollux, Pausanias, and Plutarch that work-songs and agricultural season-songs were cultivated on Greek territories, but they left no description of their sound (Mathiesen 1999, 155). As compared to volumes of writing on Greek art music, this lack of attention to folk songs indicates a trivial view of it. Aristoxenus and Philolaus mentioned that during the Archaic period Greeks were using “gapped” scales, which could have been folk hexatonic or pentatonic modes that did not receive any thorough descriptions in later literature because Classical period Greeks looked at such scales as inferior (defective) as compared to octave diatonic species, and therefore, not deserving of attention (Franklin 2002).

In Aristophanes’ Frogs, Euripides makes a parody on Aeschylus’ lines from Agamemnon by mingling them with gibberish words, working himself up by kithara-singers’ compositions. Dionysus objects, comparing parody-music to a “work-song he picked up from Marathon or somewhere” (Landels 2002, 57). The intended sarcasm targets Aeschylus’ adherence to old traditional music, opposing it to the “up-to-date” style by Euripides, and “work-song” within this context is clearly employed in a debasing connotation – most likely as an expression of general disregard for non-artistic folk style works, an attitude prevalent in Classical Greece (Barker 1990). Then it is logical to conclude that original ethnic *harmoniai* were associated with specific melodic intonations in the manner of folk modes: Heraclides Ponticus’ claims that “the sequence of melody that the Dorians used to perform they called the Dorian *harmonia*” (West 1992, 178). Then, Aristophanes was picking on the supposed “primitivism” of such folkloric modal intonations.

The earliest Ancient music theories were at the pinnacle of near-religious authority. Performance practice of Babylonian musicians was certainly disciplined by temple and court supervisors (Ziegler 2011), leaving very limited space for expression – in contrast to folk music. In Classical Greece, art music acquired a much more popular social status, and was widely practiced at households and civic public locations. This caused a surge of innovation, leaving music theory in a position to catch up with practice. Aristoxenian music theory should be viewed as an attempt to rationalize contemporary conventions and reconcile them with the legacy of the past. Hence, its tonal methodology was only partially reflective of contemporary standards. It is likely that music theorists had just started inferring principles of tonal composition from common practice of spontaneous improvisation in existing keys and scales (Mathiesen 1999, 126). Longer samples of Greek music demonstrate typical traits of improvisation, such as lack of periodicity, symmetry and repetition of patterns, except limited patterning of rhythm.

1. Audio: Mesomedes’ Hymn to the Sun, 2^nd^ century AD. A chain of ongoing melodic innovation (out of 19 lines, only 3: lines 5, 17, and 19, contain similar melodic material). <http://bit.ly/1LgM1wN>

Absolute majority of survived Greek fragments feature improvising style, whether they were intended for solo or choir performance. In this, they oppose the overall formulaic aptitude in European folklore: stability and commonality of cultural establishment that characterizes folk tradition often transpires into the prevalence of variation of a melodic formula as the principal method of folk composition (Zemtsovsky 1987).^[[1]](#footnote-1)^

1. Audio: Ne iz Sadu, bylina. Repetitions of the same melodic formula, with little variations, constitute the entire composition of an epic genre. <http://chirb.it/gDk8cr>

Out of 61 existing samples of Ancient Greek music (Pöhlmann & West 2001) only one features formulaic arrangement - an inscription from Epidaurus (West 1992, 209). It carries the text of a hymn with musical notes over the first verse only, continued after the end of a poetic line, suggesting instrumental flourish. Other verses contain accents in places different from the first verse. Since no music notes are written over the text, musician must have had to improvise adjustments on the fly, following the previous model. Since variation in this improvisation could have been substantial, even the Epidaurus inscription cannot be regarded as an example of “formulaic song.”

West (212) connects the flourish of non-repetitive music with the growing popularity of instrumental music: already in the 6^th^ century BC Sakados of Argos won the Pythian Games by performing a piece that depicted Apollo's defeat of the monstrous serpent in 5 sections of free form. The genre of dithyramb housed a free style of virtuosic performance in place of the older strophic responsion (D’Angour 2011, 201). From the 5^th^ century, melodic versatility, metrical complexity, and ornamentation were the most appreciated compositional features (D’Angour 2006). Abandoning the formulaic composition answered the public demand for originality.

It seems that music education, once instituted around the 7^th^ century BC in Sparta as means of cultivating civility (Rainbow 1995), put in place a new compositional method, different from the previous ones. Damonian ideas of moral education of citizens by means of composing music in modes that are beneficial for society (i.e. pseudo-Plutarch reports that Damon “invented” the Lydian mode) won recognition in the Hellenic world (Lord 1978). It seems that Lycurgus and Damon “revolutionized” traditional Greek music by employing music as an ideological tool.

Homer, in his epics, describes music that strongly resembles folklore: music-making is predominantly communal, without any indication of distinction between performers and audiences, no mentioning of music teaching, non-professional Achilles acts as a composer (Anderson 1979). Homeric singing itself appears to stem from the Indo-European tradition of Rigveda hymns, where pitched tones and musical intervals supported a stylized form of narration, with melodic contour being governed by the accents of the words (West 1981). Such singing was definitely formulaic.

1. Audio: Reconstruction of Homeric rhapsode epic singing with the accompaniment on phorminx, a 4-string lyre, by S. Hagel and G. Danek. <http://bit.ly/1TR8CHK>

Sometime after the 7^th^ century, lyric poets upgraded lyres at first to 7, and then to 9 strings, exploring the enharmonic modes. Tonal refinement took the limelight away from the bard tradition. Rhapsodes were not regarded as musicians, and the modal organization of their accompaniment was not discussed in any contemporary treatises (ibid.). Development of multitonal modes must have fueled the transition from formulaic to freer open-end structures, evident in the shift from older strophic to non-strophic composition in the late 5^th^ century, inspired by the idea of exploring expressive contribution of music (Scott 1982).^[[2]](#footnote-2)^

Melodic formula served as the principal formative agent that dictated to a folk musician what kind of music to make: entire genres, ethnic styles, and geographic regions can be characterized by popularity of a set of specific formulas (Zemtsovsky 1987). Perhaps, this formative power appeared unattractive to the Classic Greek music-maker who distinguished himself as art-musician, and looked down at the stereotypicity of folksongs.

This attitude must have originated in Mesopotamia. Three millennia of history provide no record of non-literate Babylonians carrying traditions of the past – the very few exceptions, such as the Sargon legend, (Drews 1974) are irrelevant to music. Peasant art occupied negligible position in Mesopotamian fine art, smaller than in other Eastern civilizations (Hauser 1999, 1:23). From rare depictions on 3^rd^ millennium BC plaques and seals, we know that shepherds piped or strummed on lutes while herding, but such music received little attention at courts and temples (Sachs 2008, 59).^[[3]](#footnote-3)^

The same applies to agricultural work-song in Egypt (Emerit 2013, 9). Absolute majority of depictions show harps or lyres held by deities or worshippers, texts call these instruments “holy,” and excavations uncover them in or near cult rooms (Lawergren 1997). What distinguished this sacrality from earlier shamanic taboo instruments (like the animal skin to frame-drums of Siberian ethnicities), was the supernatural status of certain aspects of tonal organization rather than that of the instrument per se: in Mesopotamia, intervals were connected to seasons; in Egypt, each planet had its special note, and mistake in rendering a modal intonation threatened to corrupt the musical “charm” (Farmer 1925).

Thirty hymns dedicated to the Sumerian King Shulgi, 21^st^ century BC, disclose that he perhaps was the first to pursue the institution of a new type of music based on his own “rules” of tuning. He was most likely the one who introduced interval inversion (Shaffer 1981). If so, Shulgi’s music must have radically differed from folk music, usually devoid of ICS. Indeed, the Mesopotamian music theory employed organization that is highly untypical for known folkloric cultures. Thus, the earliest documented music system (22^nd^ century BC, Nippur tablets) featured symmetrical design, where the mode was constructed from the central point towards the margins by the succession of symmetric intervals (Dumbrill 2008).

Symmetric design is evident in the depictions of giant Egyptian lyre, 16^th^ century BC, played by two blindfolded musicians, and its contemporary Hittite lyre from Inandik. Richard Dumbrill suggests that symmetric pentatonic tuning system must have been mathematically formulated at earlier times. Leon Crickmore insists that the tonal system of the entire Mesopotamian region was intrinsically symmetrical, and the family of modes used in Hurrian Hymns has been defined symmetrically so that ascending and descending scales each inverts the other (Crickmore 2009). Babylonian system introduced a new type of diatonicity in place of folk diatonic modes - true “ladders of pitches” that worked the same way reciprocally – whether going up or down (Crickmore, in press). Such music must have presented a stark contrast to the folk music of the shepherds, and motivated the adherents of "learned" music to look down at music "unlearned" and profane.

Then, Shulgi’s reforms, such as institutionalization of technique of playing seven musical instruments, foundation of new genres, (i.e. prayers, ballads, and supplications, associated with these instruments), delegating the task to disseminate music composed in these genres to scribes, and ordering his subjects to perform his music in cult-places (Klein 1981) – all, must be regarded as an anti-folk cultural campaign. This may not have been aimed specifically to reduce the consumption of folk music, but the outcome of this propaganda effort would necessarily denigrate the previously existed music standards as “vulgar” compared to those emanated from gods or kings.

Shulgi put in place professional musical administration throughout his kingdom to handle ritual, court, military, and marriage music, with the help of full-time music employees (Michalowski 2006a), setting the model for other rulers to follow.^[[4]](#footnote-4)^ In this new authoritarian model, artisans evidently did not enjoy their work: historic records reveal that they often defected, so that capturing and returning them back to the employment site presented an administrative problem (Gunter 1990). Such situation is drastically opposite to traditional folk art, where the music maker is hardly ever compelled to make music.

A number of studies of lullabies in Mesopotamian literature bring into perspective the transformation of folk genre into the authored art object, similar to the usage of folk prototypes in Western Romantic culture.^[[5]](#footnote-5)^ The purpose of performance drew the dividing line: folkloric cultures are very diverse in setting goals of performance in relation to its social applications, whereas Sumerian music is very definite in focusing on its purpose to incite pleasure or pity in gods (quite unusual for folklore) – in addition to its universally popular entertainment goal (Ziegler 2011).

It is quite common for archeologists to underline the contrast between the ecosystem of Nile, with its regularity of annual flood that promoted very stable unifying religious base and orderly model of Egyptian statehood, and the unpredictable Tigris and Euphrates, often producing flooding calamities which instituted cults centered around the issue of pacifying a destabilizing supernatural force (Knapp 1988, 23). Subsequently, Mesopotamian music had evolved along the two-genre axis of praising/laudatory hymnody and lamentation, reflected in categorization and genre affiliation of musica instruments (Shehata 2014). This specialization was evident from early times in professional organization of temple and palace music (Franklin 2007).

Anne Löhnert (Löhnert 2011) explains how lamentation grew to occupy the central position in Sumerian culture over a span of 2,000 years, by providing music to convince gods that humans are already suffering a lot, and therefore there is no need to impose more suffering on them. In addition to songs of praise, laments were supposed to result in divine favor by pacifying the “mean” deities and securing protection from the “good” deities. This conviction led to institution of professional lamenters (*gala* or *kalû)* whose job was to periodically “remind” gods of human troubles and the establishment of lamenting formulas in all principal genres of literature and music. According to Löhnert, along with professional lamenting on behalf of the community, the entire population actively partook in private lamenting and collective laudations in honor of the gods.

It appears that the entire course of development of the lamentation genre started from emulating pre-existing folkloric funeral laments, grew its own syntactic and semantic typology, and ended up by serving as a “shell” for a wide range of content, including burlesque – whatever that suits a particular personal consumption (Cooper 2006). Such development progressively abandons the stereotypical principles of oral folk art and gets closer and closer to the production of authored original composition.

The biggest novelty of new music was that its intonations were not the outcome of collective creativity of thousands of music-users, as it was in folk music, but the product of individual creativity by an author, be it Shulgi or an unknown “work-for-hire” composer. Names of better compositions and composers were preserved: Tapsihuni, Urhiya, Puhiya, and Ammiya, so were the names of the scribes responsible for the choice of source materials for notation: Ammurabi and Ipsali (West 1994, 171). Apparently, notation required not only expertise, but also responsibility in accuracy of information.

Babylonian culture was historically the first to break away from the anonymity of folk culture. Although authored works mostly belonged to the kings, some works of literature contained self-identification, i.e. Kabti-ilani-Marduk, author of the *Epic of Erra* (c.1,000 BC), and were told from the first person’s point of view for greater emotional immediacy, disclosing originality in conception and style (Foster 2011). Already, the High Priestess Enheduana from Ur (23^rd^ century BC) describes her creative process in her hymn “The Exaltation of Inanna” in terms of having inspiration by communicating to the goddess Inanna, and then delivering the poem in the same sense as “delivering a newborn baby” (Binkley 2004, 50). Such parallel implies the idea of personal relation of the author to her work, as well as the idea of ownership, since Babylonian law granted both parents the right to give their children in pledge for their debts, or to sell them into slavery (Westbrook & Beckman 2003, 50).^[[6]](#footnote-6)^

Traits of precision, selectivity and authorship are highly untypical for folk culture where all performers engage in collective music behavior - in an egalitarian spirit. Akkadian music system, supported by Sumerian achievements in math, must have sharply contrasted folk practices of the time (Michalowski 2010). Their opposition was honed by the fact that Sumerian language, on which music education and practice strongly depended, was declining already by the mid 3^rd^ millennium, and around the 21^st^ century BC it was essentially dead – oral communication in it seized to exist except in a few locations (Woods 2006). By Shulgi’s time, Sumerian became the script and instruction language reserved for a narrow circle of scholars, akin to Sanskrit, and by the time of Hurrian Hymns, it survived only in schooling of scribes and temples (Michalowski 2006b). This makes the music of these temples likely to sound esoteric to Akkadian, Hurrian, Amorite, and Elamite commoners, very different from their folk music. It must have been the strong religious authority that had been earned by the temple music responsible for its remarkable longevity: thus, gala/kalu’s Sumerian liturgy was handed down until the turn of 3^rd^ and 2^nd^ centuries BC (Shehata and Hagel 2012). Big part of it must have been the association of this music with cosmology and ethic ideas (see Appendix III).

The spread of the idea of superiority of “highbrow” educated music over “lowbrow” folklore must have followed traces of the spread of popularity of Mesopotamian harp (Franklin 2006): in Near East harps, lyres, and lutes came in favor at least since 3,000 BC. By the zenith of Indus civilization harp reached India, and after the opening of the Silk Road in the 2^nd^ century BC it was imported into China (Lawergren 2010). And Silk Road was not the first course in routing Western cultural influence: since 1920s sinologists have been talking about the penetration of Bronze Age Mesopotamian culture into China through Siberia (Barinova 2013, 58). Traditional Chinese system of tuning is remarkably close to Pythagorean (Goodman & Lien 2009), and stands apart from all the alternative tuning systems of Eastern Asia that were not influenced by China (Maceda 1990). Tradition of performing heroic epic poetry with the lyre accompaniment reached as far North as Germany and England (Lawson 2010). The ancient Hittite and Hattic words for harp have been adopted in relation to musical terms by almost all European languages, from Northern Europe to Ethiopia (Ivanov 1999). Greeks inherited Babylonian lyre along with its tuning, composition, and performance practices, as well as scales – without giving Babylonians any credit. Instead, they invented myths about the Greek origin of lyre (Duchesne-Guillemin 1984) – testifying to the prestigious view of lyre. The cyclical idea of the circle of 5^ths^ and Pythagorean method of deriving consonant tones by means of fractions most likely descended from Babylon (West 1994), and characterize those cultures that place special importance on tonal harmonization.

Just as sexagesimal division was adopted internationally for measurements of degrees and time, Babylonian tuning cycle came to serve as an international standard for tonal organization in many music systems – akin to the Ancient form of "equal temperament" (Franklin 2007). Along with it, spread the concept of refined art-music, celebrated as aesthetically superior to “unsophisticated” folk song, because of its required expertise – in opposition to egalitarian spirit of the folk song.

REFERENCES:

Anderson, Warren D. 1979. “‘What Song the Sirens Sang’: Problems and Conjectures in Ancient Greek Music.” *The Royal Musical Association Research Chronicle* 15: 1–16.

Barinova, Elena. 2013. *Ethnocultural Contacts of China with Ethnicities of Central Asia in Antiquity and Middle Ages [Этнокультурные контакты Китая с народами Центральной Азии в древности и средневековье]*. Moscow: Russian Academy of Science.

Barker, Andrew. 1990. “Public Music as ‘Fine Art’ in Archaic Greece.” In *Antiquity and the Middle Ages*, edited by James McKinnon, 45–67. Man & Music. London: Palgrave Macmillan UK. http://link.springer.com/chapter/10.1007/978-1-349-21157-9_2.

Binkley, Roberta. 2004. “The Rhetoric of Origins and the Other: Reading the Ancient Figure of Enheduanna.” In *Rhetoric before and beyond the Greeks*, 47–64. SUNY Press.

Cooper, Jerrold S. 2006. “Genre, Gender, and the Sumerian Lamentation.” *Journal of Cuneiform Studies* 58: 39–47.

Crickmore, Leon. “The Ubiquity of the Diatonic Scale.” In *Proceedings of ICONEA Conference 2014*. Oxford UK: The Oriental Institute.

———. 2009. “The Tonal Systems of Mesopotamia and Ancient Greece: Some Similarities and Differences.” In *The Archaeomusicological Review of the Ancient Near East*, edited by Richard Dumbrill and Myriam Marcetteau, 1:1–16. London.

D’Angour, Armand. 2006. “The ‘New Music’: So What’s New?” In *Rethinking Revolutions Through Ancient Greece*, edited by Simon Goldhill and Robin Osborne, 264–83. Cambridge, UK: Cambridge University Press.

———. 2011. *The Greeks and the New: Novelty in Ancient Greek Imagination and Experience*. Cambridge, UK: Cambridge University Press.

Drews, Robert. 1974. “Sargon, Cyrus and Mesopotamian Folk History.” *Journal of Near Eastern Studies* 33 (4): 387–93.

Dumbrill, Richard. 2008. “Evidence and Inference in Texts of Theory in the Ancient Near East.” In *Proceedings of the International Conference of Near Eastern Archaeomusicology, ICONEA, The British Museum, December 4–6, 2008*, edited by Richard Dumbrill and Irving Finkel, 105–16. London: Iconea Publications.

Emerit, Sibylle. 2013. “Music and Musicians.” *UCLA Encyclopedia of Egyptology*. University of California Press.

Farber, Walter. 1990. “Magic at the Cradle: Babylonian and Assyrian Lullabies.” *Anthropos: Internationale Zeitschrift Für Völker-U Sprachenkunde*.

Farmer, Henry George. 1925. “The Influence of Music: From Arabic Sources.” *Journal of the Royal Musical Association*.

Foster, Benjamin R. 2011. “The Person in Mesopotamian Thought.” In *The Oxford Handbook of Cuneiform Culture*, edited by Karen Radner and Eleanor Robson, 117–39. Oxford, UK: Oxford University Press.

Franklin, John Curtis. 2002. “Diatonic Music in Greece: A Reassessment of Its Antiquity.” *Mnemosyne* 55: 669–702.

———. 2006. “Lyre Gods of the Bronze Age Musical Koine.” *Journal of Ancient Near Eastern Religions* 6: 463–82.

———. 2007. “The Global Economy of Music in the Ancient Near East.” In *Sounds of Ancient Music*, edited by Joan Goodnick Westenholz, 27–37. Jerusalem: Bible Lands Museum.

Goodman, Howard, and Edmund Lien. 2009. “A Third Century AD Chinese System of Di-Flute Temperament: Matching Ancient Pitch-Standards and Confronting Modal Practice.” *The Galpin Society Journal* 62: 3–24.

Green, M.W. 1976. “The Eridu Lament.” *Journal of Cuneiform Studies* 30 (3): 127–67.

Gunter, Ann C. 1990. “Artists and Ancient Near Eastern Art.” In *Investigating Artistic Environments in the Ancient Near East*, edited by Ann C. Gunter, 9–17. Washington DC: Arthur M. Sackler Gallery, Smithsonian Institution.

Hauser, Arnold. 1999. *The Social History of Art: From Prehistoric Times to the Middle Ages*. Vol. 1. New York: Psychology Press.

Ivanov, Viatcheslav V. 1999. “An Ancient Name of the Lyre.” *Archiv Orientální* 67 (4): 585–600.

Klein, Jacob. 1981. “The Royal Hymns of Shulgi King of Ur: Man ’ S Quest for Immortal Fame.” *Transactions of the American Philosophical Society* 71 (7). New Philosophical Society: 1–48.

Knapp, Arthur Bernard. 1988. *The History and Culture of Ancient Western Asia and Egypt*. Belmont, CA: Wadsworth.

Krispijn, Theo. 2010. “Musical Ensembles in Ancient Mesopotamia.” In *Proceedings of the International Conference of Near Eastern Archaeomusicology, Held at the British Museum, December 4-6, 2008*, edited by Richard Dumbrill and Irving Finkel, 125–50. London: Iconea Publications.

Landels, John G. 2002. *Music in Ancient Greece and Rome*. London: Routledge.

Lawergren, Bo. 1997. “Mesopotamia, Musical Instruments.” Edited by Ludwig Finscher. *Die Musik in Geschichte Und Gegenwart: Allgemeine Enzyklopädie Der Musik*. Kassel, Germany: Bärenreiter and Metzler.

———. 2010. “Harps on the Ancient Silk Road.” In *Conservation of Ancient Sites on the Silk Road*, edited by Neville Agnew, Proceeding, 117–24. Los Angeles, CA: The Getty Conservation Institute.

Lawson, Graeme. 2010. “Floruit and Extinction in Ancient Music’s Material Record.” *Ethnomusicology Forum* 19 (2): 241–47. doi:10.1080/17411912.2010.508642.

Löhnert, Anne. 2011. “Manipulating the Gods: Lamenting in Context.” In *The Oxford Handbook of Cuneiform Culture*, edited by Karen Radner and Eleanor Robson, 402–17. Oxford: Oxford University Press.

Lord, Carnes. 1978. “On Damon and Music Education.” *Hermes* 1 (1978): 32–43.

Maceda, José. 1990. “In Search of a Source of Pentatonic Hemitonic and Anhemitonic Scales in Southeast Asia.” *Acta Musicologica* 62 (2-3): 192–223.

Marcelle Duchesne-Guillemin. 1984. “A Hurrian Musical Score from Ugarit: The Discovery of Mesopotamian Music.” In *Sources from the Ancient Near East*, edited by Ciorgio Buccellati and Marilyn Kelly-BucceIlati, 2:22. Malibu, CA: Undena Publications.

Mathiesen, Thomas J. 1999. *Apollo’s Lyre: Greek Music and Music Theory in Antiquity and the Middle Ages*. Lincoln, NE: University of Nebraska Press.

Michalowski, Piotr. 2006a. “Love or Death? Observations on the Role of the Gala in Ur III Ceremonial Life.” *Journal of Cuneiform Studies* 58 (2006): 49–61. EBSCO accession # 25532677.

———. 2006b. “The Lives of the Sumerian Language.” In *Margins of Writing, Origins of Culture: New Approaches to Writing and Reading in the Ancient Near East*, edited by Seth L. Sanders, 159–86. Chicago, IL: The Oriental Institute of the University of Chicago.

———. 2010. “Learning Music: Schooling, Apprenticeship, and Gender in Early Mesopotamia.” In *Musiker Und Tradierung Studien Zur Rolle von Musikern Bei Der Verschriftlichung Und Tradierung von Literarischen Werken*, edited by R. Pruzsinszky and D. Shehata, 8:199–239. Vienna: Lit Verlag.

Ojamaa, Triinu. 2003. “Composition Principles in Forest Nenets Music.” *Studia Musicologica Academiae Scientiarum Hungaricae* 44 (1/2): 249–56.

Pöhlmann, Egert, and Martin L. West. 2001. *Documents of Ancient Greek Music: The Extant Melodies and Fragments*. Clarendon Press. https://books.google.com/books?id=2butngEACAAJ&pgis=1.

Rainbow, Bernarr. 1995. “The Challenge of History.” *Philosophy of Music Education Review* 3 (1): 43–51.

Reiner, Erica. 1985. *Your Thwarts in Pieces, Your Mooring Rope Cut: Poetry from Babylonia and Assyria*.

Sachs, Curt. 2008. *The Rise of Music in the Ancient World, East and West*. New York: Dover Publications.

Scott, W. 1982. “Non-Strophic Elements in the Oresteia.” *Transactions and Proceedings of the American Philological Association* 112 (1982): 179–96.

Shaffer, Aaron. 1981. “A New Musical Term in Ancient Mesopotamian Music.” *Iraq* 43 (1): 79–83.

Shehata, Dahlia. 2014. “Sounds from the Divine: Religious Musical Instruments in the Ancient Near East.” In *Music in Antiquity: The Near East and the Mediterranean*, edited by Joan Westenholz, Yossi Maurey, and Edwin Seroussi.

Shehata, Dahlia, and Stefan Hagel. 2012. “Music, Ancient Near East.” Edited by Roger S Bagnall, Kai Brodersen, Craige B Champion, Andrew Erskine, and Sabine R Huebner. *The Blackwell Encyclopedia of Ancient History*. Hoboken, NJ, USA: John Wiley & Sons, Inc. http://doi.wiley.com/10.1002/9781444338386.wbeah01140.

West, Martin L. 1981. “The Singing of Homer and the Modes of Early Greek Music.” *The Journal of Hellenic Studies* 101: 113–29. doi:10.2307/629848.

———. 1992. *Ancient Greek Music*. New York, London: Oxford University Press.

———. 1994. “The Babylonian Musical Notation and the Hurrian Melodic Texts.” *Music & Letters*, 161–79.

Westbrook, Raymond, and Gary M. Beckman. 2003. *A History of Ancient Near Eastern Law*. Leiden, The Netherlands: Brill Academic Pub.

Woods, Christopher. 2006. “Bilingualism, Scribal Learning, and the Death of Sumerian.” In *Margins of Writing, Origins of Culture: New Approaches to Writing and Reading in the Ancient Near East*, edited by Seth L. Sanders, 91–120. The Oriental Institute of the University of Chicago.

Zemtsovsky, Izaly. 1987. “On Melodic Formula in Russian Folklore [О мелодической ‘формульности’ в русском фольклоре].” In *Ethnographic Origins of Folkloric Phenomena: Russian Folklore [Этнографические истоки фольклорных явлений. Русский фольклор]*, edited by V. Yeremina, 14:117–28. Leningrad: Nauka.

Ziegler, Nele. 2011. “Music, the Work of Professionals.” In *The Oxford Handbook of Cuneiform Culture*, edited by Karen Radner and Eleanor Robson, 288–312. Oxford: Oxford University Press.

1. This is not to say that folk music cannot put into use “free form” of improvisatory type, but such forms are usually limited to specific genres, and take a lesser share than formula-based forms. Also, “free” forms as a rule still contain frequent repetitions of a few modified motives – i.e. shaman songs of Forest Nenets (Ojamaa 2003). [↑](#footnote-ref-1)
2. Of course, this transition must have taken quite some time - Iliad and Odyssey were immensely popular across Ecumene (i.e. according to Plutarch, young Alcibiades hit his teacher for not owing Homer's books). Thousands of their verses in the rigid stichic form were traditionally sung to lyre accompaniment, where music had to compensate for uniformity of poetic prosody. This is where the folkloric formulaic method could have been developed by adopting multiple melodic formulas, modulations, and instrumental interludes. Similar strategies of refreshing monotonous meter can be observed in modern day epic singing (West 1992, 209). [↑](#footnote-ref-2)
3. Krispijn (2010) reports that almost all the Mesopotamian depictions of ensembles music from the 2^nd^ millennia BC belong to the realm of folk music. However, most of these depictions come from big powerful cities (i.e. Larsa and Ebla), and should therefore not be mistaken for representation of village folklore. Urban folk music is usually a lot closer to music of upper classes than to village culture – the trend that is evident in Ancient Mesopotamian poetry (Farber 1990). It is much more likely that the court/palace music of the 3^rd^ millennia had made a deep impression on the urban folk music of the 2^nd^ millennia BC. [↑](#footnote-ref-3)
4. The position of the chief musician appointed personally by the King for supervision of music production is found not only during the Old Babylonian period, but is typical for Mesopotamian courts of other periods (Ziegler 2011, 297) – with evidence attesting to the presence of one chief musician in each provincial capital city. It is possible that in his musical aspirations Shulgi himself followed some predecessor – kings of earlier dynasties delivered lamentations and prayers in temples (Green 1976), and Sumerian prayers were traditionally sung (Michalowski 2010). [↑](#footnote-ref-4)
5. Thus, Erica Reiner (Reiner 1985, 97) analyzed and interpreted the miniature Akkadian poem (c.1,000 BC) about the “Heart Grass”, showing that this magic incantation in fact was a lullaby, formatted to the religious Babylonian culture incantation formula according to what was a standard (i.e. listing 5 wishes for the speaker’s welfare and appealing to the deity). This integration of the folk genre of lullaby into the corpus of magic healing poetry, representative of the “educated” temple culture, is found in older literature (1950-1530 BC). Walter Farber describes an Old Babylonian 11-line lullaby that already utilizes quite sophisticated expressive devices, such as finishing a line with a word forming two relationships, one variable and another invariable (Farber 1990). Originality of poetic expression sets this poem as artistic implementation of the prototypical folk model, effectively transforming it into an artwork of magical literature, questing the god to use his power of invocation to put the crying baby to sleep. According to Farber, this transformation is typical for 15 Akkadian incantations from tablets of the first millennium BC. [↑](#footnote-ref-5)
6. Literary analysis of many Akkadian and Sumerian literary works reveals individual features of style (Foster 2011, 132) – and even a common literary “thank you” formula implies personalization: “I will engrave pictures of you on my arm!” [↑](#footnote-ref-6)
