# Supplementary material for: Evolution of Tonal Organization in Music Optimizes Neural Mechanisms in Symbolic Encoding of Perceptual Reality. Part-2: Ancient to Seventeenth Century
Source: Front Psychol. 2016 Mar 30;7:211. doi: 10.3389/fpsyg.2016.00211 (PMC4813086; doi:10.3389/fpsyg.2016.00211)
Supplement: Supplementary file 3 [file DataSheet1.zip › Appendices I-VIII/Appendix II. Non-octave hypermode.docx]

# Appendix-II: Modal Characteristics of Non-octave Hypermode

Hexáechos presents the simplest and most consonant implementation of hypermode (Fig.3A). The term hexáechos literally means “six modes” and refers to the smaller version of the principal Byzantine MPS – oktōēchos (eight modes). Its concept originated in Ancient Greek Systema Metabolon that united 3 tetrachords in an overlapping manner to avoid *diezeuxis* (a disjunction between the two middle tetrachords), thereby producing a row of 11 tones: A-B-C-D-E-F-G-A-Bb-C-D, with the false relation B/Bb (Kholopov 2006, 68). The idea of integrating all the tones of the pitch system in order to avoid disjunction between that system’s subsets has forged a special type of tonal organization, characterized by *treating the entire ambitus of the system as a single mode*.

Because of its relative compactness this 11-tone set could be comprehended in the integrity of most or all of its constituent tones as a stand-alone mode – especially in a multi-part setting where the ambitus of 11 tones would be broken in 2 or more registers, reserved for each of the parts. As a result of the Hellenistic cultural influence, “hypermode” earned its place in many European cultures. The Russian implementation of hexáechos presents the simplest and the most coherent hypermodal organization, and therefore makes a good model for understanding its modal principles. It is called “obykhod” (“common”) and was codified in the 5-volume 1772 edition of the Holy Synod, although it must have been used in liturgy much earlier (Kholopov 2004). In parallel to ecclesiastical use, obykhod made a deep impression on Russian folk music. It occupied the central position in Russian folklore of 15-17^th^ centuries, very much shaping the national style (Rudneva 1994, 121).

Hexáechos theory divides the entire compass into 4 trichords (in Russian, called “soglasiye” - accordance) of identical IS (tone-tone), called "simple," "dark," "light," and "ultra-light," with the characteristic false relation between Bb and B of the marginal trichords (Schidlovsky 2009). This false relation serves to identify the mode in the manner similar to tritone in the diatonic MPS system: in hypermodes the “tonic” is defined in reference to the false relation. The importance of false relation is reflected in the practice of using the key signature of B natural and B flat in modern ecclesiastic notation of chants by cantors of Russian Orthodox Church.

All tones in the hypermode are defined by names of tones of two central trichords, suggesting their centrality in the mode.^[[1]](#footnote-1)^ This testifies to a centripetal gravity, albeit weak. Modal functions are reproduced here over the 4^th^ rather than octave^[[2]](#footnote-2)^ – following the model of the Byzantine trochos (wheel) system (Kholopov 2004). Each of three basic tones can become the gravitational anchor, producing 6 obykhodnyi modes: major, minor, and diminished (ukosnyonnyi), each with authentic and plagal inclinations.

Brevity of a trichord exponentially increases the same tendency that made pentatony less gravitational than heptatony (Aleksey Nikolsky 2015): the entire mode easily breaks into 4 small components, each with its own anchor. Poly-anchoring is often combined with poly-modality of inclinations: a song in one obykhodnyi mode can easily manifest major, minor, and diminished inclinations within the same verse or chorus (Rudneva 1994, 141). In addition to the competition between anchors, the trichord segmentation causes yet another very important modal feature: it discourages leaps larger than 3^rd^ – which deprives the melodic contour of its formative role.

Compared to the MPS music, hypermodal melodic line becomes too smooth to be remembered, with too many stable tones. However, what would have been the downside for a secular MPS composition, driven by concerns for originality, has turned out to be beneficial for ecclesiastic implementation, where concordance and meditativeness is of special value. These features are especially pronounced in the monodic implementation of Znamennyi chant (Brazhnikov 1974, 2:6–8).

Unlike in the Gregorian chant, strict diatonic stepwise melodies of hexáechos give little importance to *finalis* and *repercussa* in phrases - suggesting its older origin, when the plainchant had not yet developed functional melodic specialization of different segments of a tune (Kholopov 1988, 178). The reason for greater homogeneity was stylistic preference for greater restriction of tonal tension in melodic expression of the hexáechos melodies as opposed to the Gregorian chant. Thus, Znamennyi chant melodies use neither alteration nor modulation (Kutuzov 2008, 43–51), that are quite typical for Gregorian chant (Heckenlively 1900, 52). The exceptions to intervallic diatonicity in Russian liturgical music are few, such as occasional use of F sharp and B flat in the 3^rd^ mode in the priestless communities of Old Believers (Vladyshevskaya 2006, 275).

Prominence of *finalis* and *repercussa* reflect increase in tonal tension, requiring compositional arrangement to distribute it between the starting and ending points of the music work. This is the process that has historically shaped the structural organization in a Gregorian melody. Hexáechos turns out to be simply too low in tension in order to support such organization. Its more contemplative nature, in stricter adherence to the ascetic tastes in music of the Fathers of the Church (as compared to more permissive Carolingian aesthetics) corresponds to simpler melodic elaboration and greater overall harmonicity of the modal organization.

Non-octave hypermode effectively disperses gravity, avoids tension, and conserves the mode in a way similar to pentatonic system^[[3]](#footnote-3)^ – which is likely to be responsible for lasting affiliation of diatonic-based non-octave hypermodes with religious chants in very different music cultures. Christian ideology followed Plato’s lines, condemning sensuality and refinement associated with chromatic genus of Ancient music (Heckenlively 1900, 33). This divergence was very pronounced after Christianity became the official religion of Byzantine. Crystallization of hypermodal organization as an antipode of chromatic organization most likely took place some time during 3^rd^ century AD, as a result of spread of monastic movement and growing popularity of psalmody – psalm-reading that occasionally took place in preaching of Old Testament in earlier centuries was transformed into psalm-singing at every Eucharistic service of the late 4th century (McKinnon 1990). The growing demand probably brought to life newly created melodies for New Testament.

Association of chromatic music with Dionysian pagan roots made any surviving in Near East remnants of non-diatonic music unacceptable for Christians – rejection of it was vocally advocated by St. Clement of Alexandria (Hermas et al. 2007). The new Christian merits, requested by St. Clement and St. Augustine, received musical implementation in the musical style coined by John of Damascus, based on Dorian and Phrygian *harmoniai* – and was consolidated into the paradigm of liturgical music for orthodox Christianity through the series of canonic rules adopted by the V-VII Ecumenical Congresses, 7^th^ century AD, especially in the 75^th^ rule of the VI Council (Meshscherina 2000, 89).

The outcome of new cultural policy was the decided opposition of ecclesiastic and secular tonal organizations, expressed through the change of liturgical music from singing of the entire congregation, typical for early Christians, to employment of specially trained and selected professional singers – and strict commitment to the dedicated Church modes (Aleksandr V. Nikolsky 1916). If for whatever reason it was necessary to engage a known secular tune in the music for a service, that tune had to be specially arranged by an expert in order to lose its affinity with the Earthly world. Hypermodal organization should be seen as an instrument of such “cleansing,” when the modal intonations peculiar to secular genres were deprived of their “earthly” gravity and distilled into abstract intonations of the modal subsets that constituted a hypermode. Heightened harmonicity of hypermodal IS was regarded as realization of Divine unity and integrity, thereby opposing the idea of chromatic alterations, associated with the sensual implications of the pagan chromatic music. Here music closely followed the tradition of *hesychasm* – a psychological technique of diverting attention from senses to an inward seclusion in order to achieve an experiential knowledge of God - the first mentioning of which appears in the 4^th^ century AD, in writings of Evagrius of Pontus (Ware 2000, 90).

Schism did not affect the Christian attitude to the chromatic music in any substantial way. Like Eastern Orthodox of the time, Western Medieval Catholic music theory clearly favored diatonic genera: out of 143 monochord divisions published between years 1000 and 1500, only 13 contained chromatic tunings (Herlinger 2002). Music explicitly “chromatic” in sound found its way in Western ecclesiastic genres not earlier than the 16^th^ century, in the form of *musica reservata* (Meier and Dittmer 1956). For Eastern liturgy, on the other hand, “chromatic tetrachords” started permeating the Greek church at around the same time (Zannos 1990), receiving recognition in the Chrysanthos reform (Barsky 2014) – culminated in the decision of the Ecumenical Patriarchate in Constantinople in 1883 to pronounce the chromatic and enharmonic genera to constitute the “pure form” of Christian tradition of music organization (Lind 2012, 68).^[[4]](#footnote-4)^

Perhaps due to the initial diatonic bias, Western Medieval music theory also came to embrace the non-octave hypermode modal principle, most pronounced in ecclesiastical music of the Early Middle Ages. Technically speaking, music theory had to solve the problem of handling natural “chromatic” inflections coming from the practice of folk music (see Demonsrtation-1). Performers of plainchant were also often performers of folk tunes, some of which could be chromatically inflected. The practice of oral transmission of chants was exceedingly common for early Medieval Christian liturgies and made inclusion of folk intonations and perhaps even the entire tunes possible (Jeffery 1995, 50–86) – in a way similar to what is known as deliberate policy of the local church officials during the high Middle Ages in many Western countries (Wilson-Dickson 1992, 46).

The hypermodal organization that bounded false relation to different registers was instrumental in addressing the problem of making the intervallic structure of an already existing tune fit the PS of the Church modes. Practically, a tune was tried from different degrees of the entire ambitus of a music system until the best fit for its IS was found (Bower 2002). This harmonic principle of providing the position for “extra” semitones without cluttering them in the same register sometimes led to substantial enrichment of the PCs. Thus, the 18-tone Dasian scale (G2-A2-Bb2-C3-D3-E3-F3-G3-A3-B3-C4-D4-E4-F#4-G4-A-B4-C#5) accumulated 4 false relations: “diatonic” (non-chromatic) augmented octaves Bb/B, F/F#, C/C#, broken apart by tetrachords, plus optional chromatic inflection E/Eb (Spiess 1957).

The false relations stand out to a much greater extent in the polyphonic implementations of hypermodal organization. Spacing of parts across the entire ambitus exposes the non-octave tones: each becomes assigned to a dedicated part, and the chances for them to sound very close to each other are much greater than in monody, where a singer has to climb up 7 steps in order to hit the non-octave tone. The more proximal are the non-octave tones in a music work, the greater is the tonal tension. In a multi-part setting, hypermode turns out to be considerably less restrictive on tension than in a monodic setting.

Multi-part setting makes hypermode come very close to all-permissiveness of pentatonic modes in respect to the vertical harmony that arises between the parts – with characteristic patterns of parallel 4^ths^ and 4^th^–chords in polyphonic implementations of pentatony (Kubik 2005). Melodic consonance of each of the hypermodal parts allows for vertical harmony strongly resembling polyphonic pentatony: 4^th^-chords, clusters, and parallel 2^nds^ and 7^ths^.

Trichordal design, common for pentatony, as much as for Slavic obikhod, stresses the 4^th^ as the “tonic” consonance. Many works end on the 4^th^-chords, sometimes in parallel: D-G-C resolves into C-F-Bb at the end of “Joyful Light” (175). Such “chords” were explained as “consonant” in contemporary music theories, and Solfa exercises of the 16^th^ century even included the 4^th^–chord arpeggios (190). Beliayev considered 4^th^, 5^th^, and their difference tone, major 2^nd^, to be the staples of Ancient Russian vertical harmony (Beliaev 1959).

Each of the parts may receive its own anchor tone that often cluster vertically – i.e. Cherubic Hymn in Uspenskii’s collection opens with the following anchors: A in the bass, D in the baritone, and E in the tenor (Uspenskii 1971, 170). Poly-anchoring dramatically weakens gravitational unity in a hypermode, producing a “brooding” effect.

1. Audio: The Little Entrance “Come, Let Us Worship” [Priidite, poklonimsia], 2-part Znamennyi chant. E3-Bb3 ambitus of the upper part, and A2-F3 of the lower, with B/Bb false relation. <http://bit.ly/1IO8mlX>

The more parts, the greater the tonal tension due to compression of the false relations that are distributed throughout the ambitus – plus greater harshness of harmonic intervals layered on top of each other.

1. Audio: The Little Entrance “Come, Let Us Worship,” 3-part Putevoi chant. The same melody as in the previous example is arranged in 3 parts (with the B3-F4, C4-G5, and C4-Bb4 ranges, respectively), where the middle part leads. The cluster C/D/E ends the song. <http://chirb.it/JNft2v>

Not only that hypermodal polyphony tends to enrich vertical harmony beyond the triadic base, it also manifests a peculiar chromatic tendency. Old Russian treatises inform about the existence of a 5-trichord system, with an extra upper trichord Eb-F-G that adds the diminished octave E/Eb as a second false relation (Rudneva 1994, 150). Tension can go up even further in folk implementations of obykhodnyi mode: Western Slavic folk songs can feature up to 7 trichords (124). “False” relations can be transposed an octave down in the practice of polyphonic performance, if a singer’s vocal range does not allow to hit the “false” tone in its “right” register. In such situation, the melody acquires what Rudneva calls a *shimmering 3^rd^* (150) or *shimmering 6^th^*. None of which should be regarded as chromaticism, since “false tones” are modally legitimized and perceived as relatively “stable” – any of them could potentially serve to terminate a phrase or a music work.

1. Audio: This day Christ is born in Bethlehem, 3-part Putevoi chant. A relatively rare demonstration of shimmering degrees in the 17^th^ century polyphony of Russian Orthodox Church implemented in the “mutations” (technical term for something akin to modulation) of the 2^nd^ mode (“glas”) between different parts (Kondrashkova 2012). <http://bit.ly/1ORjFsM>

Folk cultures of those nations that imported the Byzantine modal system assimilated non-octave hypermode and capitalized on its “elastic” tension. Common Slavic feature is such phrasing where the melodic line starts high and gradually collapses towards the end (9). Such tendency seems to originate from Ancient Greek music, inherited by the Byzantine chant, transmitted to local Orthodox chants of the neighboring nations and assimilated in their folk musics.

Greeks were accustomed to thinking of scales as descending, reflected in their notational denomination (naming notes in descending alphabetic order); they perceived descending tetrachordal melodic motion as more harmonious than ascending, describing the lower tones as sounding more noble and consonant than the higher tones (West 1992, 192). Such registral convention received a canonic status in the hypermodal tonal scheme: i.e. the Znamennyi chant music theory is explicit in regarding the lowest register as the most relaxed. The cantors of the Old Believer denominations, that are the most conservative followers of the Byzantine tradition in Russia, describe the “*mrachnoye soglasiye*” (dark concordance) to be requiring quiet and simple style of delivery, whereas “*tresvetloye soglasiye*” (the utmost light concordance) - the firmness and accentuation of tones (Denisov 2015, 272). Similar connotations must have been acquired by hypermodal folk traditions.

In Slavic folk music the obykhodnyi modes have been as common as Dorian and Mixolydian. For example, the song "Molodka, molodka" No.13, from Balakirev's collection uses the plagal minor obykhodnyi mode (Balakirev 1895, 30). A number of Russian composers employed obykhodnyi modes in symphonic music: i.e. Rimsky-Korsakov - in the choir *Wonderful Heavenly Queen* in *The Tale of the Invisible City of Kitezh* (Act III, Scene 1, No.167). Non-octave music construction remains very much alive in modern Russian classical music.^[[5]](#footnote-5)^

Other offsprings of Byzantine chant contain even more complex modal structure in their hypermodal implementations. Greater complexity seems to stem from polyphonic methods of arrangement for the canonic melodies, subsequently becoming the model for local folk musicians (Oniani 2010). Here, most spectacular are Georgian tetrachordal or pentachordal hypermodes - depending on whether the mode is built by a conjoining 4^ths^ or 5^ths^ (Gogotishvili 2010).

The backbone of both types is the Mixolydian heptatonic mode.^[[6]](#footnote-6)^ In *tetrachordal* implementation, it starts on the I degree of major mode, which serves as the primary hypermodal inclination (See Fig.3B in the main paper). The lowest tetrachord is plagal, featuring two unstable degrees in a row, the upper of which serves as a leading tone. All tetrachords are conjunct, thereby easily invoking gravitational alternation between their lowest tones. However, the central tetrachord often engages the “tonic” 3^rd^, which opposes the unstable triad, built on the leading tone.

Symmetric melodic motion between the middle and the marginal tones of this triad characterizes this mode’s harmony (Gogotishvili 2003). This functionality induces hierarchic relations, where the unstable function plays a formative role. The upper tetrachord can counterbalance the tonic by its basic tone, alternating from unstable to stable state – then emphasizing the tip of the ambitus. However, overall, the E/Eb diminished octave secures centripetal gravity.

1. Audio: Kakhuri nana. Lullaby. Georgian tetrachordal hypermode with the diminished octave G#/G. <http://chirb.it/yqEtCD>

The *pentachordal* Georgian hypermode (Fig.4 below) stitches the conjunct pentachords that promote odd/even functionality throughout most of the ambitus, except its upper end. This mode assigns a formative power to the stable triad, which surrounds the unstable 3^rd^. Gravity easily alternates between the lowest and the central pentachords, causing bi-functionality of the IV degree in the central pentachord (expressed in the common 4^th^–chord F-Bb-C). The upper tetrachord distinguishes this mode by introducing the augmented octave Eb/E that pushes the melodic inertia outwards (Eb-D E-F), making gravity centrifugal (Gogotishvili 2010). The Ionian version sets the model for three transpositions (Dorian, Phrygian, Lydian).

Pentachordal modes usually sound more meditative than tetrachordal, due to the integrative influence of hierarchic stability. Tetrachordal modes produce impression of more of reliance on the “dominant” harmony due to their hierarchic instability.


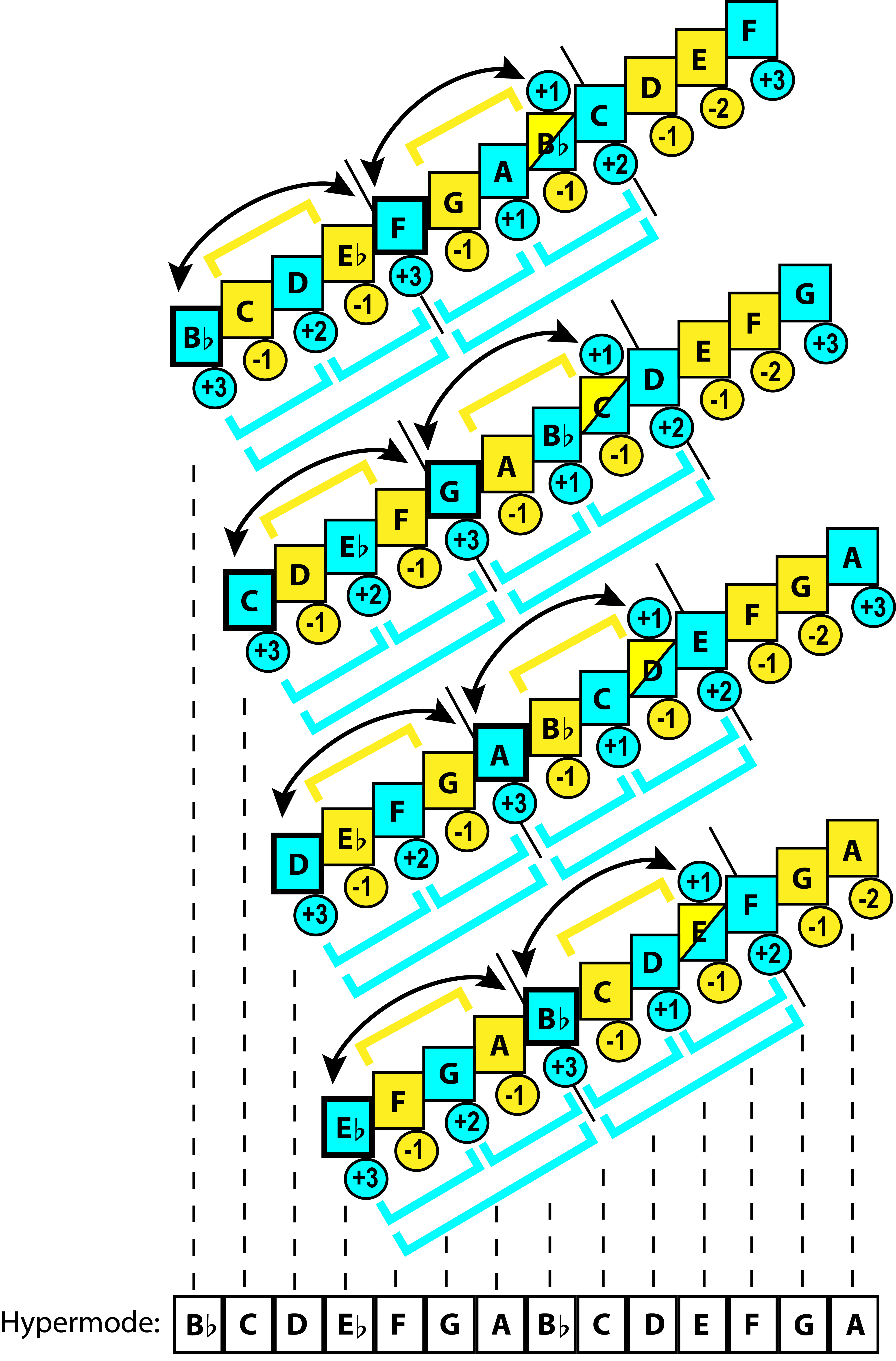
**Figure 4. Four Georgian pentachordal non-octave hypermodes: Ionian (Bb), Dorian (C), Phrygian (D), and Lydian (Eb)**. Like tetrachordal hypermodes, pentachordal hypermodes are based on *subset inequivalence*: each subset contrasts the other two in its gravitational design. The marking conventions are the same as in the previous figure. Stable tones form a triad that encapsulates the unstable dyad (as opposed to the tetrachordal modes in Fig.3B). Contrary to tetrachords, pentachords institute hierarchy of stable rather than unstable degrees. As a rule, central pentachord alternates in gravity with the lowest pentachord, which often outweighs and causes the IV degree in the central pentachord to switch its function from unstable to stable. This affords 4^th^-chord structures (F-Bb-C) common for this mode. The upper tetrachord compliments the central pentachord. The augmented octave (Eb/E in Ionian version) determines the overall *centrifugal* gravity of this hypermode. The other three versions follow the same tonal mapping.

1. Audio: Alilo, Guria. Pentachordal mode with the augmented octave Fb/F in polyphonic implementation. <http://bit.ly/1JTrOz7>

Augmented octave is not bound to Georgian music. Uzeir Hajibeyov identified the principles of modal construction in Azerbaijanian mugam music which are very similar to Gogotishvili’s pentachord and tetrachord modes – based on equivalence of 4^th^, 5^th^ and major or minor 6^th^ – almost all of the typical modes bearing the false relation, i.e. diminished octave in Shur, and augmented octave in Humayun (Gadjibekov 1957). Something very similar characterizes the Armenian traditional music (Pashinian 1973). In his monumental monograph dedicated to investigation of tonal organization in monodic music, Christofor Kushnaryov came to the conclusion that any compound modal structures employed within the folk tradition is likely to invoke the formative capacities of the circle of 5^ths^ and 4^ths^, and combine it with the principle of surrounding an anchor tone by symmetric tritones in a modular manner, generating the centripetal gravity (Kushnaryov 1958, 372).

In 1929, Mikhail Gnesin conducted research of the Hebrew chant repertoire and identified a hexaechos system similar to Byzantine, where five out of six modes engaged “false relation” between augmented Bb/B or Eb/E (Zemtsovsky 2012). The nature of this non-octave organization is purely melodic and monodic, driven by expressive tuning where the high registers B-C and E-F are sharpened, whereas the low Eb-D and Bb-A are flattened.

Another implementation of the pentachord system with the augmented octave is found in the Tvisongur celebrational genre of Iceland (Bukofzer 1940). It is possible that the hypermode principle was forged independent of Greek influence in some other cultures: the 1910’s Chinese phonogram of a *sheng* composition reveals the PS with “false relation” D/Db (Bb-C-D-Eb-F-G-Ab-Bb-C-Db-Eb), sticking out like a sore thumb in an otherwise predominantly pentatonic culture, which the transcriber puts it on the account of “false” tuning of the upper Db by “faulty” performers (Fischer 1911, 168). However, yet another case of consistent registral “false relation” Ab/A is evident in another phonogram from the same collection (184), making it look like a deliberate modal principle rather than a “mistake.” Beliayev noticed a similarity between the obykhodnyi scale and the 9-tone scale of Chinese pipa (lute with fixed modes): G-A-Bb-B-C-D-Eb-E-F (Beliayev 1990, 368) – perhaps this similarity could have transpired into generation of “false relation” in *pipa* music, where the trichord organization (G-B, C-E, F-A) would become modally formative.

Hypermodality promotes polyphonic music by engaging parts in highlighting the false relation between registers and stressing different anchors across a wide ambitus. The resultant textures do not have a “melody” per se – the “melody” is dissolved in texture as the conglomerate of all the participating parts (Jordania 2006, 103). In this way *hypermodal polyphony* differs from melody-driven *heptatonic heterophony*, where melodic variants between the participants do not splice the texture in the permanent musical streams that would be sustained from the starting point to the end of the music.

The landmark of hypermodal organization is that it institutionalizes the opposition of musical tonal organization to that of speech.^[[7]](#footnote-7)^ Hypermode permanently fixes apex of tension at the highest register, and its nadir – at the lowest register – in polar opposite to intonation in both, human as well as animal vocal communication (Ohala 2006). Hypermode presents the first decided breakaway from natural biological roots of music into the new territory of specialized musical semantics based on harmonic postulates of advanced musical civilization. Noteworthy, such “burning of bridges” occurs under the aegis of Christianity, characterized by antinomy between educated morality and wilderness of natural state.

Christian culture should be seen as the cultural force that necessitated hypermodal organization as a working compromise in the divergence of the diatonic and chromatic MPS systems. Just as much as the chromatic music system was brought to life by the aesthetic need in chromatic *modulation* (see Appendix III), the hypermodal system was brought to life by the aesthetic need in *avoiding modulation*. Capacity of hypermode to integrate multiple modal subsets of various structures is used as an alternative to modulation in the MPS. Historic development of non-octave hypermode is clearly marked by its lasting affinity with religious chants, which must have had a pronounced impact on the unique way a hypermode handles tension.

REFERENCES:

Balakirev, Milii. 1895. *Collection of Russian Popular Songs [Сборник русских народных песен]*. Leipzig: Edition Belaieff.

Barsky, Vladimir. 2014. *Chromaticism*. Routledge. https://books.google.com/books?id=jGJ9AwAAQBAJ.

Belaiev, Victor. 1963. “The Formation of Folk Modal Systems.” *Journal of the International Folk Music Council* 15: 4–9. doi:10.2307/836227.

Beliaev, Viktor. 1959. “Early Russian Polyphony.” In *Studia Memoriae Belae Bartók Sacra*, edited by Benjamin Rejeczky, 311–32. New York, London: Boosey and Hawkes.

Bower, Calvin M. 2002. “The Transmission of Ancient Music Theory into the Middle Ages.” In *The Cambridge History of Western Music Theory*, edited by Thomas Christensen, 136–67. Cambridge, UK: Cambridge University Press.

Brazhnikov, Maksim V. 1974. *Monuments of Znamennyi Chant [Памятники знаменного распева]*. Vol. 2. Leningrad: Muzyka [Музыка].

Bukofzer, Manfred F. 1940. “Popular Polyphony in the Middle Ages.” *The Musical Quarterly* XXVI (1): 31–49. doi:10.1093/mq/XXVI.1.31.

Chrysanthos, and Kaitē Rōmanou. 1973. *Great Theory of Music by Chrysanthos of Madytos*. Translated by Kaitē Rōmanou. Bloomington, IN: Indiana University.

Cook, Scott. 2000. “Consummate Artistry and Moral Virtuosity: The ‘Wu Xing 五行’ Essay and Its Aesthetic Implications on JSTOR.” *Chinese Literature: Essays, Articles, Reviews* 22: 113–46. doi:10.2307/3109445.

Denisov, Nikolai. 2015. *Old Believers’ Liturgical Vocal Culture. The questions of typology. [Старообрядческая богослужебно-певческая культура. Вопросы типологии]*. Moscow: Progress-Traditsiya.

Fischer, Erich. 1911. “Beiträge Zur Erforschung Der Chinesischen Musik. Aus Dem Phonogrammarchiv Des Psycholog. Instituts Der Universität Zu Berlin.” *Anthologies of the International Musical Society* 12 (2 Jan.-March): 153–206.

Gadjibekov, Uzeir. 1957. *The Foundations of Azerbaijanian Folk Music [Основы азербайджанской народной музыки]*. 2nd ed. Baku: Azmuzgiz [Азмузгиз].

Gogotishvili, Vladimer. 2003. “On Some Characteristics of Mode-Intonational Scales in Kartli-Kakhetian Long Table Songs.” In *Proceedings of the First International Symposium on Traditional Polyphony (2-8 October 2002)*, edited by Rusudan Tsurtsumia and Joseph Jordania., 312–23. Tbilisi, Georgia: Tbilisi State Conservatoire.

———. 2010. “On Authentic and Plagal Types of Monotonic (Non-Octave)Scales in Georgian Traditional Vocal Polyphony.” In *Echoes from Georgia: Seventeen Arguments on Georgian Polyphony (Focus on Civilizations and Cultures)*, edited by Rusudan Tsurtsumia and Joseph Jordania, 147–56. New York: Nova Science Publishers.

Heckenlively, Lura. 1900. *The Fundamentals of Gregorian Chant*. Tournai, Belgium: Society of St. John Evangelist, Desclée & Co.

Herlinger, Jan W. 2002. “Medieval Canonics.” In *The Cambridge History of Western Music Theory*, 168–92. Cambridge, UK: Cambridge University Press.

Hermas, Tatian, Theophilus, Athenago, and Clement. 2007. *The Ante-Nicene Fathers: The Writings of the Fathers Down to A. D. 325*. Edited by Alexander Roberts. Vol. 2. New York: Cosimo, Inc.

Jeffery, Peter. 1995. *Re-Envisioning Past Musical Cultures: Ethnomusicology in the Study of Gregorian Chant*. Chicago, IL: University of Chicago Press.

Jordania, Joseph. 2006. *Who Asked the First Question? The Origins of Human Choral Singing, Intelligence, Language and Speech*. *The Origins of Human Choral Singing, Intelligence, …*. Tbilisi, Georgia: Logos.

Kholopov, Yurii. 1988. *Harmony: A theoretic course [Гармония: теоретический курс]*. Moscow: Muzyka [Музыка].

———. 2004. “Obychodnyi Modes and Polyphony [Обиходные лады и многоголосие].” In *Christian Culture: Past and Present; 2000 Anniversary of Birth of Christ [Христианская культура: прошлое и настоящее. К 2000олетию Рождества Христова]*, edited by Guliantiskaya N.S., 39–54. Moscow: Gnessin Russian Academy of Music [Российская академия музыки имени Гнесиных].

———. 2006. *Musical-Theoretic Systems [Музыкально-теоретические системы]*. Moscow: Kompozitor.

Kondrashkova, L.V. 2012. “Three-String Christmas Stikhira ‘This Day Christ Is Born in Bethlehem’ [Троестрочная рождественская стихира ‘Днесь Христос в Вифлееме.’” *Courier of the Orthodox Saint-Tikhon Humanitarian University [Вестник Православного Свято-Тихоновского гуманитарного университета]* 3 (9): 173–90.

Kubik, Gerhard. 2005. “The African Matrix in Jazz Harmonic Practices.” *Black Music Research Journal* 25 (1/2): 167–222. http://www.jstor.org/stable/30039290.

Kushnaryov, Christofor. 1958. *Matters of History and Theory of Armenian Monodic Music [Вопросы истории и теории армянской монодической музыки]*. Edited by Robert Atayan. Art Instit. Moscow: Gos Muz Izdat [Гос. муз. изд-во].

Kutuzov, Boris P. 2008. *Russian Znamennyi Chant [Русское знаменное пение]*. Moscow: Andrei Rublev [Андрей Рублев].

Lind, Tore Tvarnø. 2012. *The Past Is Always Present: The Revival of the Byzantine Musical Tradition at Mount Athos*. Lanham, MD: Scarecrow Press. https://books.google.com/books?id=VPWjSeLibr4C.

List, George. 1961. “Speech Melody and Song Melody in Central Thailand.” *Ethnomusicology* 5 (1): 16–32.

McKinnon, James W. 1990. “Christian Antiquity.” In *Antiquity and the Middle Ages*, edited by James W. McKinnon, 68–87. London: Macmillan Publishers.

Meier, Bernhard, and L. A. Dittmer. 1956. “The Musica Reservata of Adrianus Petit Coclico and Its Relationship to Josquin.” *Musica Disciplina* 10: 67–105.

Meshscherina, Yelena G. 2000. *Musical Culture of Russia during the Middle Ages [Музыкальная культура Средневековой Руси]*. Moscow: Znaniye [Знание].

Nikolsky, Aleksey. 2015. “Evolution of Tonal Organization in Music Mirrors Symbolic Representation of Perceptual Reality. Part-1: Prehistoric.” *Frontiers in Psychology* 6 (1405). doi:http://dx.doi.org/10.3389/fpsyg.2015.01405.

Nikolsky, Aleksandr V. 1916. *The Brief Outline of the History of Liturgical Singing during the I-X Centuries AD [Краткий очерк истории церковного пения в период I-X вв]*. Sankt-Petersburg: Jurgenson [Юргенсон].

Ohala, John J. 2006. “The Frequency Code Underlies the Sound-Symbolic Use of Voice Pitch.” In *Sound Symbolism*, edited by Leanne Hinton, Johanna Nichols, and John J. Ohala, 325–47. Cambridge, UK: Cambridge University Press.

Oniani, Ekaterine. 2010. “On the Polyphony of Georgian Chant.” In *Proceedings: The Fifth International Symposium on Traditional Polyphony: 4–8 October, 2010, Tbilisi, Georgia*, edited by Rusudan; Tsurtsumia and Joseph Jordania, p. 381–86. Tbilisi, Georgia: Tbilisi State Conservatoire.

Pashinian, Eduard. 1973. “Universal Super-Modal System in Armenian Music [Универсальная суперладовая система в армянской музыке].” *Historical Philological Journal of the National Academy of Sciences of Armenia*, no. 3: 194–212.

Rudneva, Anna. 1994. *Russian Traditional Musical Works: Essays on the Theory of Folklore [Русское народное музыкальное творчество: очерки по теории фольклора]*. Moscow: Kompozitor [Композитор].

Schidlovsky, Nicolas. 2009. “Sources of Russian Chant Theory.” In *Russian Theoretical Thought in Music*, edited by G. D. McQuere, 83–108. Rochester, NY: University of Rochester Press.

Solovyova, Polina. 2013. “The Role of the Obihod Scale in the Harmonic Organization of Russian Music [О роли обиходного звукоряда в ладовой организации русской музыки].” *Musical Academy [Музыкальная академия]*, no. 2 (January): 83–98.

Spiess, Lincoln Bunce. 1957. “An Introduction to the Pre-History of Polyphony.” In *Essays on Music in Honor of Archibald Thomson Davison by His Associates*, 11–15. Cambridge, MA: Harvard University.

Uspenskii, Nikolai. 1971. *The Samples of Ancient Russian Art of Singing [Образцы древнерусского певческого искусства]*. 2nd ed. Leningrad: Muzyka [Музыка].

Vladyshevskaya, Tatiana V. 2006. *Musical Culture of Ancient Russia [Музыкальная культура Древней Руси]*. Moscow: Znak.

Ware, Kallistos. 2000. *The Inner Kingdom*. Crestwood, New York: St Vladimir’s Seminary Press.

West, Martin L. 1992. *Ancient Greek Music*. New York, London: Oxford University Press.

Wilson-Dickson, Andrew. 1992. *The Story of Christian Music: From Gregorian Chant to Black Gospel: An Authoritative Illustrated Guide to All the Major Traditions of Music for Worship*. Minneapolis, MN: Fortress Press.

Zannos, I. 1990. “Intonation in Theory and Practice of Greek and Turkish Music.” *Yearbook for Traditional Music* 22 (1990): 42–59.

Zemtsovsky, Izaly. 2012. “M. Gnesin on the Modal System of Jewish Music [М. Ф. Гнесин о системе ладов еврейской музыки].” *Scholarly Bulletin of the Moscow Conservatory* 4: 6–25.

1. The tones of the two middle trichords receive their special names that reflect their modal functions: 1) Dark Ut is called “very low,” 2) Dark Re “low,” 3) Dark Mi “light,” 4) Light Ut “dark,” 5) Light Re “higher” and 6) Light Mi “high.” These functions are marked by the abbreviation of the first letter in the notation (i.e. GN “gorazdo nizko” – “very low” for Dark Ut). The marginal trichord tones use the same names as the central trichords, with addition of special signs: Simple Ut is marked by the sign “x” in front of the letters GN, and the same subscript “x” is placed in front of the abbreviation for Dark Re and Mi. Ultra-light Ut is marked as M with a dot on top of the letter M (“Mrachno” – “dark”), and the other two tones receive a dot on top of the first letter of their abbreviation (Kholopov 1988, 180). This notation is indicative of the centrality of two middle trichords, and derivation of marginal trichords from the center. [↑](#footnote-ref-1)
2. Each “concordance” (*soglasiye*) contains an identical structure of 3 tones, called “ut”, “re” and “mi” (named after the Western Guidonian hexachord system). Each tone is identified by its position in relation to the neighboring degrees: Ut is separated from the lower degree by a semitone, and from the higher degree by a tone; Re – by a tone on both sides; and Mi – by a tone underneath and a semitone above. The entire melody is made of these 3 functions alone, each one of which is capable of taking the lead and becoming “*finalis*”. [↑](#footnote-ref-2)
3. Their close relation is evident in Russian folklore, where earlier pentatonic intonations form the nucleus of melodies conceived in obykhodnyi modes after the 15^th^ century (Rudneva 1994, 121). [↑](#footnote-ref-3)
4. This new canon of neo-Byzantine chant constituted an abandonment of the hypermodal principle of tonal organization in favor of the chromatic organization of a newer hemiolic type (see Appendix IV). The ability to easily shift back and forth between the diatonic and chromatic genera of the II and VI modes, and between the diatonic and enharmonic genera of the III and VII modes made chromatic/enharmonic modulation a commonplace for the Eastern Orthodox plainchant, also permitting whole range of chromatic alterations practically in any of the modes (Chrysanthos and Rōmanou 1973). [↑](#footnote-ref-4)
5. Yurii Butsko (b. 1938) elaborated the obykhodnyi mode into a 12-tone system by adding trichords above and below the main scale. This technique was introduced in his Polyphonic Concerto (1969), and was used almost in every work of his thereafter, as well as works of other Russian composers (Solovyova 2013). [↑](#footnote-ref-5)
6. This formative role of the Mixolydian mode for the hypermodal organization mimics its similar function in the genesis of the regular heptatonic modes and diatonic system (Belaiev 1963). [↑](#footnote-ref-6)
7. Earlier forms of speech/music tonal opposition are confined to the diatonic MPS of cultures that have tonal languages. Thus, in Thai language the pitch contours for lyrics coincide with those for folk tunes, but tend to mismatch for songs of court music (List 1961). Since pentatony features higher harmonicity than heptatony, pentatonic cultures promote stronger unnaturalness of “high-art” melodic intonation, perpetuated by Confucius in his opposition of two levels of music in delivering “happiness” to the commoner by pleasing him versus delivering “happiness” to the nobleman – by elevating him (Cook 2000). Pentatonic MPS of cultures with tonal languages are most likely to have preceded hypermodes, but featured more of case-to-case tonal opposition, bound by pronunciation of certain words. [↑](#footnote-ref-7)
