# Supplementary material for: Evolution of Tonal Organization in Music Optimizes Neural Mechanisms in Symbolic Encoding of Perceptual Reality. Part-2: Ancient to Seventeenth Century
Source: Front Psychol. 2016 Mar 30;7:211. doi: 10.3389/fpsyg.2016.00211 (PMC4813086; doi:10.3389/fpsyg.2016.00211)
Supplement: Supplementary file 3 [file DataSheet1.zip › Appendices I-VIII/Appendix III. Alteration as expression of aesthetic emotion.docx]

# Appendix-III: Alteration as Expression of Aesthetic Emotion

The first documented evidence of semiotic ascription of specific emotional attributes to specific music structures is presented by the Ancient Greek doctrine of ethos (Shestakov 1966). Different patterns of meter/rhythm as well as different melodic modes, each was held to convey a particular “ethos” (literally, “character”). In essence, ethea should be regarded as the first implementation of “*musical emotions*” (Juslin and Västfjäll 2008) – discrete emotional states primed to specific patterns of melodic or rhythmic organizations by means of repetitive use in music practice, according to public conventions.^[[1]](#footnote-1)^

Emergence of “musical emotion” was made possible by the development of a new listening culture that can be characterized as “*structural hearing*” – practice of detection of melodic direction and the concise intervallic distances between the tones of a melody (Lippman 1964, 160). Such style of listening strongly contrasted the holistic discrimination between different melodic contours in known repertory of melodies – the prevalent listening style in most folk cultures.

Although the doctrine of ethos received the greatest exposure in the Ancient Greek civilization, its origins are certainly much older (Farmer 1965).^[[2]](#footnote-2)^ The idea of "ethos" originates from the very idea of "harmonia," and characterizes the entire region from Greece to Central Asia (Franklin 2002a). Harmony, according to Classical Greek sources, is a "unification of things that on a lower level appear to be dissimilar or unrelated," and therefore lacking in order (Mathiesen 1984). This unification equally applies to planets, humans, and musical tones.

Platonic notion of mimesis explains how pitch and rhythm in music execute the same principles as those in the celestial order, and thereby exercise their power over the listeners in the same way that the sun and the moon do. As such, music obtained an astrological importance: listening to a particular mode was equivalent to being influenced by a particular combination of planets and stars. In fact, PCs were associated with specific planets and deities in Greek, Chinese, and Indian music systems (Sachs 1957).

The birth of this astrological connection took place in Babylon, and was spread across Europe and Near East by the Pythagoreans. Pythagoras, recognized as the father of mathematics, music, and ethics, learned his crafts from Chaldeans (Farmer 1965). Although Babylonian cosmological teaching proved to be so influential, it was not the only source for the concept of "music of the spheres" (James 1995). Along with the idea of ethos, the teaching of cosmological harmony was found in all the world’s chief "learned" MPS-based music systems: Western (Meier 1990), raga (Rowell 1981), yayue (Thrasher 2008), maqam, and dastgah (Pacholczyk 1996). Connection of tonal organization in music to social order and cosmological principles has worked as a backbone for music cultures of Near and Far East, as well as India (Manuel & Blum 2011).

Imposition of prescriptive modal theory was certainly absent in prehistoric music – just as it is absent in music of surviving hunter-gatherer's societies. The village musician does not think in terms of abstract rules – his music-thinking is concrete and materialistic: he merely follows a known melodic model, which might allow to alter a given tone, add a "chain" interval, or transit to another melodic model in a different mode. This type of thinking is song-oriented. The typology pioneered in Babylon was mode-oriented. Because of this, Babylonian culture favored polymodal instruments. Retunable instruments were prestigious: lyres and harps were deified in rituals and as poetic elaboration by the same poet-priests who used them (Franklin 2006). Harps and lyres were buried in Royal tombs, depicted in hands of deities and played at courts, whereas pipe instruments were considered common (Lawergren 2000) – and such discrimination pertained throughout the archaic Antiquity. A Sumerian proverb said: “A disgraced musician becomes a piper, a disgraced lamentation-singer becomes a flutist!” (Ziegler 2011).

Helladic culture shared the Babylonian convictions. Greek mythology assigned lyre to Apollo, and pipes to satyr Marsyas, who presumably had picked pipes from the ground where Athena casted it away as ugly, after inventing it (Keer 2004). Aristotle condemned the use of pipes in education because of their “orgiastic” reputation (pipers’ vulgarity was proverbial in Greece) and pipe’s obstruction of “normal” way of using one’s mouth to verbally express himself (Csapo 2011). The underlying reason for this long-standing prejudice could be that early pipe could play only a few modes, thereby being restricted in ethos. Harp/lute allowed for retuning, and was therefore initially more versatile in choice of modes (Hagel 2009, 56).^[[3]](#footnote-3)^

Yet another important reason, brought out by Plato, was the greater precision in tuning and performance on string instruments, that produced tones in strict accordance to the mathematical theory – as opposed to volatility of intonation on woodwinds and possibility of pitch-bending, forcing the performer to strive to (in Plato’s words) “hunt out the right tuning for each and every note” (Barker 2009).

Noteworthy, aulos gained popularity greater than any string instrument - even noble Athenians were taking aulos lessons (P.Wilson 1999) – but only after a series of technical modifications enabled it to *play modulations*, while harp required to stop the music in order to retune its strings (Franklin 2013). Ease of playing chromatic alterations and ability to play multiple modes evidently constituted great value in the eyes of Ancient Greeks, which eventually outweighed imperfections and inconsistencies of chromatic tuning.

The historic development of tonal organization from fixed *prescriptive* diatony to flexible “*descriptive*”^[[4]](#footnote-4)^ chromatic system ran hand in hand with an important change in aesthetic values of musical composition: away from numerical cults (akin Tetraktys) to humanistic theatricality.

By and large, *prescriptive modal theory*, together with conventions of ethos, conserved the *diatonic polymodal* system. This conservation has proved to be a powerful historic force in the development of tonal organization within "learned" music systems across all cultural differences – quite internationally. Any mathematically defined music tuning system necessarily fixes singular values for each degree of a MPS, which enforces a *pointillistic* (using Garbuzov's term) understanding of a normative pitch (Garbuzov 1950).^[[5]](#footnote-5)^

Until very recently, whenever deviation from the normative pointillistic tuning took place, variability of a certain degree in pitch was regarded as "imperfection" by classical music theory (Rags 1980). Aspirations of performers to employ expressive tuning was frowned upon by the theoretic authorities, and viewed perhaps as an "inevitable evil." So, music practice remained an arena for an ongoing struggle between the *prescriptive* harmonic norm and the “*subscriptive*”^[[6]](#footnote-6)^ melodic intonations that attracted admiration of performers and justified breaking theoretic rules.

Besides installing a pointillistic standard of pitch, Mesopotamian music theory influenced the posterity yet in another important way – by pioneering and promoting orchestral music. Equalization of degrees within a diatonic MPS moderated the gravity by flattening tonal hierarchy and regulating expressive tuning. This was beneficial for ensemble music, especially for orchestral performance that called for greater uniformity, restricting the use of intra-modal inflections.

Orchestral playing must have had a cleansing effect on diatonicity. The prehistoric flautist could satisfy the need for diversification by simply bending a given pitch closer to the new anchor. Orchestral musician in a 15-piece orchestra, described in literary texts of the 2^nd^ millennium BC (Krispijn 2010), must have been much more disciplined in his intonation. The concept of diatonic ISC, new to Babylon, was likely to encourage all participating musicians to keep their tuning in sync and in strict correspondence to the requirements of a particular genre.

Such standard was to the advantage of the orchestral Mesopotamian music, but to the disadvantage of solo performance – as it turned out, when lyrical poetry and tragedy started growing in importance in Ancient Greece. Helladic music system must have initially closely followed the Mesopotamian one, but broke away some time at the turn of the 6^th^ and 5^th^ centuries BC (Franklin 2002b). Stefan Hagel rightfully stresses that the Babylonian and Greek notations served different purposes (Hagel 2005):

- The notation of Hurrian hymns is based on notation of dichords (a harmonic interval regarded as a single percept), indicating that the notation tablets were intended for the *instrumentalists*, where one of the dichordal notes possibly reflected the tone of the melody;
- The Greek notation was designed for a *singer* – out of all surviving documents only one can be interpreted as notating a discrete accompaniment line, otherwise Greek symbols of instrumental notation merely transliterate symbols of vocal notation.

This distinction stems from the fact that the accompaniment in Classic Greek tradition was primarily executed by a single instrument, even in accompaniment for the choir, in contrast to the Mesopotamian music that favored large ensembles (ibid.). Consequently, Mesopotamian music theory was governed by vertical harmony: it was most important for all the participating musicians to be aware of the correct harmony in order to play in tune – with that knowledge even the performance by a very large orchestra could have been coordinated by a “conductor” – a Sippar tablet suggests that hand signals were used to refer to the name of a mode that was to be performed (Kilmer 1984, 77). Therefore, the Babylonian tuning system was based on just tuning (Crickmore 2009), most beneficial for chords and poorly suited for melodies. Greek music, in contrary, was based on Pythagorean tuning that is most favorable to melodic implementation (Barbour 2004).

Bound by purity of the harmonic intervals, Mesopotamian and earliest Greek music must have stayed rigidly diatonic, generally avoiding modulations – and in doing that following the modal principles of folk village cultures. But it seems that already in Archaic Greece, the newly formed Greek national identity in music had to do with generous use of modulations and non-diatonic tunings (Hagel 2005). The earliest reported mode, the so-called *Spondeion scale* that was used to accompany the libation rites featured a special case of modal organization with a prominent tritone (Mathiesen 1999, 357–61) and apparently served as the source for emergence of the enharmonic genus (Winnington-Ingram 1928). The ultimate takeover by the chromatic and enharmonic genera in Hellenic public musical taste had to do with concerns for originality and intensity of artistic expression. By Plato's times, criticism of monotony of "correct" traditional music became common in public discourse (Franklin 2002b) - orthodox diatonic music began making a stale and unremarkable impression due to its perceived shortcoming in tonal tension. In emotionally intense genres, such as tragedy, musicians as a rule resorted to enharmonic and chromatic genera.^[[7]](#footnote-7)^

Thereby chromaticism was earning a positive reputation. Contrary to inflections in village folk cultures, polymodal alteration had aesthetic roots: it was appreciated for its rhetoric capacity to convince the listeners in virtual tension of a play or a poem.

Genuine folk culture usually lacks “aesthetic” evaluation in its pragmatics. Especially archaic folklore misses three important criteria of the “aesthetic” notion (Merriam 1964, 259):

- folk listener does not distance himself from the perceived song, and cannot remove himself from music in order to examine it as an autonomous entity;
- folk artist does not manipulate music form for its own sake – he does not make creative decisions as to how to configure the elements of musical composition in order to assemble the whole;
- folk community does not attribute emotion-producing qualities to music structures alone.

Archaic folklore is recognized by its syncretic unity of the song, singer, and listener, where expression is believed to emanate from the subject of the song, and music structures are inseparable from ritualistic verbal formulas, determining a peculiar listening style that is characterized by the absence of a concept of a “music work,” where music is perceived as the process of creativity rather than its result (Sheikin 2002, 4–7).

Singers of such music are not capable of estimating the pitch aspect of musical expression separately from that of lyrics – even when asked – and do not know the notion of “error” in relation to their music (Ojamaa & Ross 2011). Any music expression that uses music forms familiar to the listeners is typically perceived as “right.” Neither music structures are detachable from their genre frames: shaman melodies are never performed outside of shamanic rites, and dance melodies are inseparable from the bodily motions associated with them (Dobzhanskaya 2012). Archaic music culture of Aboriginal ethnicities of Siberia, such as Nganasan, is based on the animistic system of beliefs according to which *sound* is the attribute of a live matter as opposed to *silence* that represents a dead matter (Dobzhanskaya 2016). In such a worldview there is no “wrong” musical tone – every audible sound by definition “exists.” Yurii Sheikin qualifies this aesthetics as "Cartesian": "I sing therefore I am" (2002, 304).

The birth of notion of musical error concurs with introduction of chromatic music. The earliest account of reference to “error” in music seems to be pseudo-Aristotle’s “Problems” – the term “*cheiron*” is used there in reference to mishandling melodic intervals while singing out-of-tune (Losev 2000, 4:623), which seemed to imply a logical error, when an inappropriate item contradicts the surrounding context. The notion of musical error requires distinction between musical and verbal components in singing – something that usually stays inseparable even in advanced folk music cultures (Zemtsovsky 1987). In Ancient Greek culture, they were evidently separate already in the 6^th^ century BC: as Andrew Barker tells us, “when Pindar sent poetic manuscripts to distant patrons, he also sent clear instructions about the manner of performance and the style of the music, and even a proxy director, carefully rehearsed in advance by Pindar himself” (Barker 1990).

Here again, as in the case of similarity between patterns of acquisition of vocal skills by infants and early stages of tonal evolution (Nikolsky 2015), we observe similarity between processing of the pitch/lyrics combination by infants and by performers of music that features early types of tonal organization. Developmental research discloses that in infancy phonetic and melodic information is processed together, and the ability to segregate them emerges later in life (Lebedeva & Kuhl 2010). Evolution of song could very well follow the same path.

Perception of music in early folk culture is extremely direct: almost every imaginable part of life is expressed through music and becomes “one” with music. Alekseyev describes songs reserved for the state of being happy, dissatisfied, humorous, becoming sick, falling in a maniac state, falling-asleep songs, taiga- or coast-songs, and even death song of terminally ill Yakut, which scared off little children who overheard his singing (Alekseyev & Nikolayeva 1981, 61).

Much of such singing is aimed at no one but the singer himself, and therefore has no need in “convincing” anybody in anything – merely reflecting on one’s current physiological state. A certain activity often suggests singing all by itself: thus, in Nganasan culture riding a reindeer sledge is unthinkable without an accompanying road-song (*tararsa baly*) (Dobzhanskaya 2016). And whenever singing is addressed to the audience, the latter perceives that the singer either speaks for himself, if he uses his “normal voice,” or believes that some spirit is taking possession of that singer, in order to communicate through his mouth – if he sings with a “strange voice” (Novik 2004, 272). In both cases, the message of a song is completely “direct”: the song means exactly what the lyrics say.

Dissanayake (2015) qualifies such aesthetic as “naturalistic” and grounds it in the “aesthetic primitives” of entrainment, play, and ratification. Aesthetic aspect in appreciation of “direct music,” as intended by its creator, is secondary to the utilitarian aspect, and usually constitutes a general attitude towards the topic (i.e. fear of calamity or love to the motherland). In archaic folk cultures, such as music of Aboriginal ethnicities of Siberia and Far East, singing someone else’s song is considered inappropriate and even potentially dangerous, capable of inflicting trouble on the impropriator’s well-being (Dobzhanskaya 2016). In more advanced folk cultures, performers do sing favorite “cover songs,” in which case the aesthetic aspect becomes more prominent and involves evaluation of authenticity in reproduction of a conventional model: a listener appreciates creativity of a musician in faceting his own rendition of intonations of familiar melody or genre, filling it with his personal emotional expression (Zemtsovsky 1979) – which still remains a form of “direct speech” rather than theatric impersonation.

A folk music work is evaluated through the “aesthetic” prism only when the song is stylized by a professional non-folk musician, and is intended for an audience that is aware of fictiveness of music presentation (that the singer just follows a “script” of a song, and does not speak for himself) – however, it is questionable whether calling such music “folklore” is accurate (Moore 2011). Perhaps, it is necessary to reserve a special category of “implied authorship” typical for those advanced folk cultures that develop distinction between “composer” and “interpreter”^[[8]](#footnote-8)^ – such as Tatar, Kazakh, and Mongolian cultures, starting from at least the 17^th^ century (Zemtsovsky & Kunanbayeva 2011).

1. Audio: Balabekov – Shal ani, Kazakh lyric song. According to Zemtsovsky, this song belongs to a traditional genre “25 years,” where the singer impersonates an 80-years old man, overwhelmed by diseases and absorbed in memories of his youth. Aesthetic emotion in advanced folk culture. <http://chirb.it/JJJgEE>

Offering an archaic folk song to the modern Western audience triggers “aesthetic” evaluation as well, which should be regarded as a perceptory bias, unintended by an original archaic music-maker (Zemtsovsky 1983).^[[9]](#footnote-9)^ Matthew Gelbart (2011, 203) demonstrates that the notion of “folk music” as a retainer of oral national tradition was forged during the late 18^th^ century to counterbalance the agency of the individual genius, whose mission was seen to aestheticize the collective through “great art,” thereby merging individual and the national. Remarkably, Johann Herder, the ideologist of folklore, saw the model for extraction of popular features for creation of “noble” and “living” (200) artworks in Ancient Greek poetry, exemplified in Homer’s works.

It seems that chromatic modulation (modulation combined with alteration) presents a reliable criterion to distinguish between “aesthetically” driven forms of art and primordial folk art.^[[10]](#footnote-10)^ It receives theoretic description in Ancient Greek treatises of the 4^th^ century BC,^[[11]](#footnote-11)^ and occupies an important position in music systems based on Ancient Greek theoretic heritage, such as maqam. At least from the 13^th^ century on, Arabic treatises regulate ways of passing from one mode to another via special subsidiary modes called forth to either extrapolate from the main modes or to elaborate on them (Marcus 1992).^[[12]](#footnote-12)^

Modulation is found in music systems that were formed completely independent of Greeks. The concept of *xuangong* (modal shift) in the yayue music was documented during the early Tang dynasty (Xiaodun, Xiaohui & Shijun 2004). Both, maqam and yayue, cultivate aesthetic judgment in listeners and the compositional construction of music, from a set of pre-selected elements by the performing musician.

What appears to be the first historic account of distinction between “aesthetic” art and “natural” folk tradition of melody-making comes from the 14^th^ century scholar Ibn Khaldun, who distinguished between the simple style of Bedouins and the art of learnt singing, capable of delivering twelve different modes of addressing from the artist to his audience (Blum 1975). These twelve modes apparently constitute twelve styles of impersonations – evidence of the growing complexity and specialization in application of aesthetic emotions. Earlier Greek-influenced music systems most likely followed suit.

If Helladic musician intuitively observed some oral “music theory” in reproducing a known melodic model within the conventional modal framework, Hellenic musician calculated which tones, modes, and rhythms to choose out of the allowed assortment in order to express an ethos appropriate for a given situation, according to the “science” of melic composition (Mathiesen 2008). Chromatic modulation was a part of it.

Of course, in practice, it is not always easy to delineate between the two. Cultural exchange erodes the boundaries. Beliayev (Belaiev 1963) reports that modulations are not uncommon for advanced folk cultures.

1. Audio: Ah Ya Helw Ya Masaleeny, Syrian folk wedding song. Modulation from A to D that seems to be influenced by maqam rules. <http://chirb.it/zArMb2>

Such development occurs primarily in cultures where string music occupies a prominent role, and wind instruments lag behind strings in their technical proficiency (as it happened with lyre and aulos in Ancient Greece). Chromatization usually corresponds to professionalization of music-making, where learning an instrument requires “talent” and involves dedicated apprenticeship for extended period of time – listeners of this music usually socially distinguish themselves from performers and evaluate music from the position of appreciating the performer’s execution. Such attitude should be regarded as a pre-requisite of “aesthetic” mode of listening, and segregation of the performer/listener roles is a germ of “aesthetic” consumption of music.

Modern Western popular music presents a case similar to folklore, where a culture of expression in many popular styles/genres features some hybridized form of perception, transitional from “folk” to “aesthetic” mode: i.e. the rock culture implies accessibility of performing rock music to any listener – normative rock idioms are expected to be technically easy enough for an average fan to reproduce them on the guitar, keyboard, or vocally. Like folk music, rock music is definitely not cultivated through notation and treatises, but by ear (Miller 2009) - yet some styles of rock utilize the aesthetic appreciation of virtuosic performance on electric guitar, which then becomes highly professionalized (Walser 1992).

Remarkably, rock music also acquires *composite* modal organization - what David Temperley calls "supermode": a set of adjacent scale degrees, related by the circle of 5^ths^, extending from low VI to high VII degrees, excluding low II and high IV – this pitch set is found in 700 rock songs (Temperley 2011). It is used for modal transpositions (and possible modulations) that give rock music a strong taste of modality. Furthermore, modal organization in rock music closely resembles that of pentatonic tonality. The melodic line in rock is based on what Temperley calls pentatonic "union scale," C-D-Eb-E-F-G-A-Bb (Temperley & de Clercq 2014) – which is identical to Beliayev's composite major/minor pentatonic mode (Belaiev 1963).

Even more complex can be the *polymodal composite* organization. Folk practice is malleable to assimilation. It readily borrows the idea of combining characteristic portions of popular modes in cultures with strong exposure to “learned” music systems. Research by Bartok uncovered the folk practice of assembling a melody out of elements of multiple modes – which served as the prototype for Bartok’s own music (Antokoletz 1984, 27).

Technically speaking, such method of modal construction is intermediate between “modulation” per se and composite modality, featuring instant alternations between completely different modal subsets within exactly the same register, i.e. G-A-Bb-C-Db, G-Ab-B-C, G-Ab-Bb-C and G-A-B-C (Hornbostel 1975, 1:107). A song can make a case of either modulation between a few discrete modes, or a single composite melodic mode, depending on:

- how consistent is the use of certain tetrachords within a composition,
- the span of their usage,
- their correspondence to a particular register or/and melodic direction,
- their contribution to the identity of the tune.

Composite melodic mode can be quite complicated: i.e. it can reserve one set of tetrachords for ascending motion, and another – for descending. Also, the connection of tetrachords can switch between conjoined and disjoined. Other subset units can get involved: trichords or pentachords. In the folk music of nations with advanced art music, tonal organization can resemble kaleidoscope of modal subsets in Brownian motion of contrasting, varying and substituting each other – usually interpreted by performers as brief “accidental” modulations (Yöre 2012).

However, even in advanced non-Western music systems that feature professionalism, notation and codified music theory, alteration usually remains rather limited – in contrast to the Ancient Greek music and Western classical tradition. Thus, in his theory of mugam, Uzeir Hadjiveyov reserves alteration for only the modes of Rast, Shur and Segah, further limiting them to a few specific degrees, i.e. lowering of the VI and raising of the IV degrees – otherwise leaving the opportunity of alteration only for the auxiliary tones in melodic embellishments, such as G-F#-G (Gadjibekov 1957, 100).

Chromatic system, which was certainly unknown to Babylonians (Gurney 1994), should be seen in light of progressive aesthetization of tonal order. Certain types of proportionality were upheld as more beautiful than others. The Babylonian music system was abstract metaphysical: their modes were made of conjunct tetrachords that shared a mutual tone, producing heptachords. Hence, 7 and 4 were sacred numbers, with cosmological symbolic meaning attributed to them. The Greek music system was concrete phenomenological. Greek modes were made of disjunct tetrachords, producing octave species – which left 4 as the only sacred number. Absence of a mutual tone allowed for greater diversity of interval classes that could be combined in a PS for a composition.

It looks like Babylonian tuning was designed to fit cosmologically important numbers, using sexagesimal arithmetic and tone-numbers taken from their standard tables of reciprocals and divisors – resulting in “just tuning” as reference (Crickmore 2009). Just tuning (the term "just" referring to the purity of intervals) is notorious for causing serious problems for melodic performance: just intonations work poorly in modulation, and cause singers to flatten the melodic line, gradually drifting away from the initial tuning – unless at some place they would break away from the just intonation (Barbour 1938).^[[13]](#footnote-13)^

The limitations of just tuning seem to be well known to the Ancient Greeks. Thus, Plato’s writings in the “Republic” and five other dialogs (“Symposium”, “Timaeus”, “Critas”, “Statesman”, and “Laws”) contain the allegory between social organization and just tuning, presenting the latter as unattainable in real life practice – unless some “moderation” of the rules takes place (Mcclain 1974).

Pythagorean tuning, employed by Greeks, was much more practical: it was defined experimentally (Barker 2007) with the help of a special testing instrument, monochord, suited to continuous singing a’capella, and worked perfectly well for a monodic setting (Barbour 1933). Therefore, the historic development from Babylonian “metaphysically” driven divinization of 4 and 7 to Greek “physically” driven (by experimental support) divinization of 4 should be viewed as evolution from “abstract” aesthetics to “actual” aesthetics.

Subsequently, Babylonian aesthetic emotion must have entailed more direct and permanent attribution: connection between certain features of ethos and certain musical structural patterns was rigid and clear-cut, defined in an astrological impersonal manner. Greek aesthetic emotion included a phenomenological aspect – it was driven by the actual experience of a musical sound and accounted for the pragmatics of perception. Greek aesthetic emotion involved *impersonification* and was closely related to theater. However, as Andrew Barker points out, the immediate cause for emergence of musical theatricality must have been wide spread of musical competition – the first signs of which can already be identified at around 700 BC, when Hesiod won the prize for song at the king's funeral games at Chalkis in a kitharodia contest (Barker 1990). The task of impressing the listeners while beating other contenders subdued the art of musical composition to rhetoric principles not that different from principles of judicial rhetoric formulated by Aristotle (Aristotle 2004).

Abandoning the formulaic composition answered growing demand for originality – and avoidance of modal stereotypicity became part of the new game. In the 6^th^ century BC Sakados of Argos won the Pythian Games by depicting on pipe, in 5 dedicated movements, how Apollo defeated the monstrous serpent (West 1992, 212). Timotheos imitated the storm in his composition “Nauplios,” and Semele’s cries in his “Birthpangs of Semele” (D’Angour 2011, 201). Such music definitely followed for sound-painting of a plot rather than the conventional melodic intonations from folk songs.

Kitharists learned this new composition method from pipers: i.e. Lysander of Sicyon developing *syrigmos* (sound imitation of hissing snake) amongst other “special effects” in kithara playing technique. The widespread practice of kithara accompaniment for solo singing paved the way for free form and “special effects” to enter genres of dithyramb, and become typical for the actor’s arias in tragedies (214). The flexible rules of the music competitions, set to encourage inventiveness, took precedence over the rigid modal rules of earlier diatonic, as well as unwritten modal rules of folk music (Barker 1990).

Authorship has promoted radical development in expressive means of all arts, as compared to their state prior to Hellenic civilization. As radical was the rise of chromatic order in Athenian music during the 5^th^ century BC, pretty much reflecting the antithesis of diatonic conventionality vs. chromatic originality. Timotheos proclaimed a self-conscious disregard for traditional music: “Get lost, ancient Muse!” (D’Angour 2011, 201). Instead, he proudly asserted his own technical proficiency and innovation, setting artistry as role model for generations to come (Csapo & Wilson 2009).

The shift of popularity from diatonic music, exemplified by “noble” string instruments, associated with philosophy and knowledge, to chromatic music, exemplified by “vulgar” aulos, associated with pleasure – during the course of the 5^th^ century BC, appears to mark a historic transition from collective prescriptive aesthetic emotional approach to individualistic descriptive emotional approach – which once forged was there to stay (Franklin 2013). For at least the next half-millennium, enharmonic and chromatic genera exceeded the diatonic genera in popularity, the latter of which was viewed as too predictable and unimpressive (Franklin 2002b).^[[14]](#footnote-14)^

Institution of the periodically held pan-Hellenic musical competitions^[[15]](#footnote-15)^ had a formative influence on Ancient Greek literature and music for both, artists and audiences (Rotstein 2012). Smaller competitions were held locally during religious festivals and important communal events in the form of choir competitions, often sponsored by prosperous citizens (Mark 1995). Almost every major city featured its own musical contest of some kind, completely open to foreigners who often won – which encouraged integration of multiple folk traditions into some kind of pan-Hellenic mainstream of musical art of composition (Barker 1990).

The competitive spirit of Greeks made musical competitions no less fierce than sport competitions (Kennell 1995, 223).^[[16]](#footnote-16)^ Such competitive spirit must have involved originality in distribution of tension in melody, causing noticeable switching from earlier “diatonic rules,” bound by the letter of musical law, to new “chromatic rules,” bound by the spirit of musical law. Evidently, the latter was by far more flexible than the former. We know that in the 5^th^ century BC Athens, modulation became the issue of controversy – not because of its novelty (earlier practice must have allowed for diatonic modulations), but because of its excessive use, alterations, and violation of prosodic rules (Franklin 2013).

1. Audio: Katolophyromai fragment from Orestes by Euripides, from papyrus, 3^rd^ century BC. Lamentation in chromatic Lydian mode, attributed to Euripides, who was strongly criticized at his lifetime for excessive modal complexity. <http://bit.ly/1g3VzB5>

Chromaticism as “sweetening” of intervals by illuminating a subtle delicacy in tonal shading served to evoke states ranging from “pleasant” to “lugubrious” (using Aristides’ words) (Franklin 2005) – essentially, aesthetic emotions. It is this function of entertaining and catering to the likes of the crowd that the critics of chromaticism viewed as “effeminization,” threatening to reduce the ability of citizens to defend their state from the enemy. Greater expressiveness of chromatic shading was criticized for sensuality, more appropriate for female rather than male gender (Csapo 2011).

Adherents of traditional diatonic music perceived chromaticism as a departure from enlightening ethics in favor of pure entertainment. Plato saw the divider in observance of cosmogonic order by diatonic music-users versus libertinism of chromatic music-users, seduced by poets who succumbed to “lawless innovation,” possessed with “inordinate delights,” and affirming that music can only be “judged of rightly by the pleasure of the hearer” (Plato 2013, 68).

However, the underlying dividing line seems to be not so much between ethical values and entertainment, as it might appear from the well-known philippics by Plato and Aritotle, but between two types of emotional content referenced by music: music representing “concrete” human emotions (emotional state of a particular person in a particular situation) in a *theatrical* manner, versus music representing ideal emotional states (an “abstract” emotion taken as generalization of a particular emotional experience amongst all people) in a *religious* manner.

The choice for chromatic/enharmonic genera only followed the choice for theatrical manner of expression. Thus, greater excitement and instability, required by the genre of dithyrambic *kampai*, were the reason why Philoxenos admitted his failure to compose a dithyramb in the diatonic Dorian mode, as told by Aristotle (Henderson 1957). All known works by Timotheos were descriptive of a particular story or event (what today would have been regarded as “program music”), and there was a fierce ongoing competition between professional musicians as to who could tell a story through music in a more exciting way – most notably between Timotheos and Philoxenos (Power 2013).

Moreover, theatricality of chromatic music must be responsible for the emergence of first voices of criticism of the Classical theory of ethos, starting from the Hibeth Papirus of the 4^th^ century BC (Anderson 1966, 147–149), followed by the treatises “Against the Musicians” by Sextus Empiricus and “On Music” by Philodemus (Ferguson 2003). Philodemus, the most adamant opponent of the orthodox view that melodic and rhythmic structures were capable of inducing certain emotions in listeners, specifically talked about enharmonic and chromatic music (397). The point of his attack was that “no melody has its character by nature, only by convention” (402), and therefore no claims of connection between cosmology and music were valid, whereas all meaning traditionally found in music was, in his view, suggested by the lyrics.

This critique was the expression of Epicurean materialistic philosophy, applied onto the field of music, reducing it to the physical phenomena of sounds, while discarding any psychology of musical perception as inherently “subjective” and insubstantial to music (Halliwell 2009, 250–6). It is highly likely that this “atomistic” understanding of music was prompted by the realization of general “fakeness” of theatric representation of reality, where an actor only pretends as though he is doing something, and spectators choose to believe him. Debunking musical ethos would have hardly been possible without the parallel between theatrical impersonation and musical expression – highlighted in the chromatic Athenian New Music.^[[17]](#footnote-17)^

Evidently, chromaticism corresponds to significant increase in aesthetic functionality of music composition, and reduction in the attribution of cosmogonic animism to music – which was a remnant of the folk belief in the supernatural power of music. Once discovered by Greeks, chromatic shading proved to be so useful that it could not be forgotten despite the anti-chromatic campaigns of the Fathers of the Church. Even in the most anti-chromatic culture of the Old Believers’ Orthodox music that rejected the Nikonian reform, sticking to the early Christian tradition, occasional alteration of F sharp makes its way in the alleluia sections of psalms, probably to mark their elevated excitement (Vladyshevskaya 2006, 268).

The increased expressive capacities of chromatic alteration have caught attention of musicians coming from all sorts of backgrounds, from village to palace or temple, across the Mediterranean region and adjacent areas. Whether by means of folk-style oral transmission or via music theory derivative from Ancient Greek cultural sources, such as maqam (Zannos 1990), chromatic alteration turned into a window of showing emotional “spectacle” to the music user.

1. Audio: Martinete, Spain. This folkloric genre of cante flamenco and the older cante jondo is characterized by the subject of suffering from prosecution, and intense chromatic elaboration (all possible alterations within the tonic pentachord), which nevertheless does not obscure strong tonicity. <http://chirb.it/DhaAny>

It could be said that the aesthetics of the diatonic system was based on admiration of the harmonious integration of diverse elements, “submerging their differences in a cooperative union” (Barker 2010) - association of beauty with proportionality, and ugliness with disproportionality, subduing the former to the latter. Athenian New Music replaced this cosmogonic numerological aesthetics with admiration for impersonation of humanistic character traits, interwoven into a dramatic development. Once launched, this theatric treatment of musical composition won sufficient public interest to stay alive by passing from one culture to another, including folk and “high art,” adapting techniques of alteration to a local tradition, while maintaining the principle of stimulating the listener by means of cognitive dissonance, triggered by alteration.

Christian aesthetics managed to resist this “dissonant” influence for quite a while after St. Clement of Alexandria adopted the Ancient doctrine of the “music of the spheres” along with belief in its connection to the ethic qualities, contributing to the emergence of the idea of long cherished “heavenly music” (Cosgrove 2006). However, theatric attitude to music slipped out to the Medieval Western histrionic plays, mysteries and festivals, where through the secular poetic genres it found its way back to the ecclesiastic music in the form of *musica reservata* in the 16^th^ century (Palisca 1959). In the East, all the Orthodox Churches except Russian, by the 19^th^ century adopted chromatic music that operated on not theatrical but rather “visionary” principles of evoking spiritual bliss in the audiences (see Appendix IV). All in all, chromatic alteration has retained its affinity with aesthetic mode of listening to music, determined by the rhetoric function to suggest a particular emotional state to the listener, while disposing him toward embracing this influence.

REFERENCES:

Alekseyev, Eduard, and Nadezhda Nikolayeva. 1981. *Samples of Yakut Vocal Folklore [Образцы якутского песенного фольклора]*. Yakutsk: Academy of Sciences of USSR.

Anderson, Warren D. 1966. *Ethos and Education in Greek Music: The Evidence of Poetry and Philosophy*. Cambridge, MA: Harvard University Press.

Antokoletz, Elliott. 1984. *The Music of Béla Bartók: A Study of Tonality and Progression in Twentieth-Century Music*. Berkeley, CA: University of California Press.

Aristotle. 2004. *Rhetoric*. Whitefish, Montana: Kessinger Publishing.

Barbour, James Murray. 1933. “The Persistence of the Pythagorean Tuning System.” *Scripta Mathematica* 1. s.n: 286–304.

———. 1938. “Just Intonation Confuted.” *Music & Letters* 19 (1): 48–60.

———. 2004. *Tuning and Temperament: A Historical Survey*. New York: Dover Publications.

Barker, Andrew. 1990. “Public Music as ‘Fine Art’ in Archaic Greece.” In *Antiquity and the Middle Ages*, edited by James McKinnon, 45–67. Man & Music. London: Palgrave Macmillan UK.

———. 2007. *The Science of Harmonics in Classical Greece*. Cambridge, UK: Cambridge University Press.

———. 2009. “Text and Sense at Philebus 56A.” *The Classical Quarterly* 37 (01): 103. doi:10.1017/S0009838800031694.

———. 2010. “Mathematical Beauty Made Audible: Musical Aesthetics in Ptolemy’s Harmonics.” *Classical Philology* 105 (4): 403–20.

Belaiev, Victor. 1963. “The Formation of Folk Modal Systems.” *Journal of the International Folk Music Council* 15: 4–9. doi:10.2307/836227.

Blum, Stephen. 1975. “Towards a Social History of Musicological Technique.” *Ethnomusicology: Journal of the Society for Ethnomusicology* 19 (2): 207–31.

Christesen, Paul, and Donald G. Kyle. 2013. *A Companion to Sport and Spectacle in Greek and Roman Antiquity*. Vol. 8. John Wiley & Sons.

Cosgrove, Charles H. 2006. “Clement of Alexandria and Early Christian Music.” *Journal of Early Christian Studies* 14 (3): 255–82. doi:10.1353/earl.2006.0049.

Crickmore, Leon. 2009. “The Tonal Systems of Mesopotamia and Ancient Greece: Some Similarities and Differences.” In *The Archaeomusicological Review of the Ancient Near East*, edited by Richard Dumbrill and Myriam Marcetteau, 1:1–16. London.

Csapo, Eric Godfrey. 2011. “The Economics, Poetics, Politics, Metaphysics and Ethics of the ‘New Music.’” In *Music and Cultural Politics in Greek Andchinese Societies*, edited by Dimitrios Yatromanolakis, 1:65–132. Cambridge MA: Harvard University, Department of the Classics.

Csapo, Eric Godfrey, and Peter Wilson. 2009. “Timotheus the New Musician.” In *The Cambridge Companion to Greek Lyric*, edited by Felix Budelmann, 277–93. Cambridge, UK: Cambridge University Press.

D’Angour, Armand. 2011. *The Greeks and the New: Novelty in Ancient Greek Imagination and Experience*. Cambridge, UK: Cambridge University Press.

Dissanayake, Ellen. 2015. “‘Aesthetic Primitives’: Fundamental Biological Elements of a Naturalistic Aesthetics.” *Aisthesis* 8 (1): 5–24.

Dobzhanskaya, Oksana. 2012. “On the Concept of ‘Music’ in Regards to Archaic and Early Folkloric Cultures [О понятии «музыка» применительно к архаичным и раннефольклорным культурам].” In *XVI Tzarskoselskiye Readings [XVI Царскосельские чтения]*, edited by V. N . Skvortsov, 16-III:256–59. Sankt-Petersburg: Leningrad State University named after Pushkin.

———. 2016. “The Live Makes Sounds, the Dead Is Silent [Живое - звучит, мертвое - молчит].” *Anthropology and Archeology of Eurasia*. in print.

Falkenhausen, Lothar von. 1992. “On the Early Development of Chinese Musical Theory: The Rise of Pitch-Standards.” *Journal of the American Oriental Society* 112 (3): 433–39. doi:10.2307/603079.

Farmer, Henry George. 1965. “The Éthos Of Antiquity.” *Islamic Studies* 4 (1): 25–30.

Feldman, Walter. 1993. “Ottoman Sources on the Development of the Taksîm.” *Yearbook for Traditional Music* 25: 1–28.

Ferguson, Everett. 2003. “The Art of Praise: Philo and Philodemus on Music.” In *Early Christianity and Classical Culture: Comparative Studies in Honor of Abraham J. Malherbe*, edited by Abraham J. Malherbe, John T. Fitzgerald, Thomas H. Olbricht, and L. Michael White, 391–428. Leiden, The Netherlands: Brill. https://books.google.com/books?id=UEwRAQAAIAAJ.

Franklin, John Curtis. 2002a. “Harmony in Greek and Indo-Iranian Cosmology.” *The Journal of Indo-European Studies* 30 (1-2): 1–25. http://www.moisasociety.org/de-musicis/harmony-greek-and-indo-iranian-cosmology.

———. 2002b. “Diatonic Music in Greece: A Reassessment of Its Antiquity.” *Mnemosyne* 55: 669–702.

———. 2005. “Hearing Greek Microtones.” *Ancient Greek Music in Performance. Vienna: Wiener Studien Beiheft* 29: 9–50.

———. 2006. “Lyre Gods of the Bronze Age Musical Koine.” *Journal of Ancient Near Eastern Religions* 6: 463–82.

———. 2013. “Song-Benders of Circular Choruses’- Dithyramb and the ‘Demise of Music.” In *Song Culture and Social Change: The Contexts of Dithyramb*, edited by P. Wilson and B. Kowalzig, 213–36. Oxford: Oxford University Press.

Gadjibekov, Uzeir. 1957. *The Foundations of Azerbaijanian Folk Music [Основы азербайджанской народной музыки]*. 2nd ed. Baku: Azmuzgiz [Азмузгиз].

Garbuzov, Nikolai. 1950. *Zonal nature of tempo and rhythm [Зонная природа темпа и ритма]*. Moscow: Academy of Science of USSR [Изд-во Академии наук СССР].

Gelbart, Matthew. 2011. *The Invention of “Folk Music” and “Art Music”: Emerging Categories from Ossian to Wagner*. Reprint ed. Cambridge: Cambridge University Press.

Gurney, O. R. 1994. “Babylonian Music Again.” *Iraq* 56 (1994): 101–6.

Hagel, Stefan. 2005. “Is Nîd Qabli Dorian? Tuning and Modality in Greek and Hurrian Music.” *Baghdader Mitteilungen* 36: 287–348. http://cat.inist.fr/?aModele=afficheN&cpsidt=17961761.

———. 2009. *Ancient Greek Music: A New Technical History*. New York: Cambridge University Press.

Halliwell, Stephen. 2009. *The Aesthetics of Mimesis: Ancient Texts and Modern Problems*. Princeton, NJ: Princeton University Press.

Heimonen, Marja. 2003. “Music Education and Law: Regulation as an Instrument.” *Philosophy of Music Education Review* 11 (2). Indiana University Press: 170–84.

Henderson, Isobel. 1957. “Ancient Greek Music.” In *Ancient and Oriental Music*, edited by Egon Wellesz, 336–403. London; New York: Oxford University Press.

Hornbostel, Erich M. von. 1975. *Opera Omnia*. Edited by Klaus P. Wachsmann, Dieter Christensen, and Hans-Peter Reinecke. Vol. 1. The Hague, Netherlands: Martinus Nijhoff.

James, Jamie. 1995. *The Music of the Spheres: Music, Science, and the Natural Order of the Universe*. 1st ed. 19. New York: Copernicus.

Juslin, Patrik N., and Daniel Västfjäll. 2008. “Emotional Responses to Music: The Need to Consider Underlying Mechanisms.” *Behavioral and Brain Sciences* 31 (05). doi:10.1017/S0140525X08005293.

Katz, Jonathan. 1996. “Music and Aesthetics: An Early Indian Perspective.” *Early Music* 24 (3): 407–20. http://www.jstor.org/stable/3128258.

Keer, Ellen van. 2004. “The Myth of Marsyas in Ancient Greek Art: Musical and Mythological Iconography.” *Music in Art* 29 (2): 20–37.

Kennell, Nigel M. 1995. *The Gymnasium of Virtue: Education & Culture in Ancient Sparta*. Chapel Hill, NC: University of North Carolina Press.

Kilmer, Anne Draffkorn. 1984. “A Music Tablet from Sippar (?): BM 65217+ 66616.” *Iraq* 46 (2): 69–80.

Kosiewicz, Jerzy. 2004. “Ancient and Contemporary Olympic Games: Religious and Anthropological Aspects.” In *European Integration and Sport*, 121–30. LIT Verlag Münster.

Krispijn, Theo. 2010. “Musical Ensembles in Ancient Mesopotamia.” In *Proceedings of the International Conference of Near Eastern Archaeomusicology, Held at the British Museum, December 4-6, 2008*, edited by Richard Dumbrill and Irving Finkel, 125–50. London: Iconea Publications.

Lawergren, Bo. 2000. “Extant Silver Pipes from Ur, 2450 BC.” In *Musikarchäeologie Früher Metallzeiten*, edited by E. Hickmann and I. Laufs, 2:121–32. Rahden, Germany: Verlag Marie Leidorf.

Lebedeva, Gina C., and Patricia K. Kuhl. 2010. “Sing That Tune: Infants’ Perception of Melody and Lyrics and the Facilitation of Phonetic Recognition in Songs.” *Infant Behavior and Development* 33 (4). Elsevier Inc.: 419–30. doi:10.1016/j.infbeh.2010.04.006.

Lippman, Edward Arthur. 1964. *Musical Thought in Ancient Greece*. New York: Da Capo Press.

Losev, Aleksey. 2000. *History of ancient aesthetics: Aristotle and late classics [История античной эстетики: Аристотель и поздняя классика]*. Vol. 4. Moscow: Folio.

Manuel, Peter, and Stephen Blum. 2011. “Classical Aesthetic Traditions of India, China, and the Middle East.” In *The Routledge Companion to Philosophy and Music*, edited by Theodore; Gracyk and Andrew Kania, 245–56. Abingdon, Oxfordshire: Routledge.

Marcelle Duchesne-Guillemin. 1984. “A Hurrian Musical Score from Ugarit: The Discovery of Mesopotamian Music.” In *Sources from the Ancient Near East*, edited by Ciorgio Buccellati and Marilyn Kelly-BucceIlati, 2:22. Malibu, CA: Undena Publications.

Marcus, Scott. 1992. “Modulation in Arab Music: Documenting Oral Concepts, Performance Rules and Strategies.” *Ethnomusicology* 36 (2): 171–95.

Mark, Michael L. 1995. “Music Education History as Prologue to the Future.” *Bulletin of Historical Research in Music Education* 16 (2): 98–121.

Mathiesen, Thomas J. 1984. “Harmonia and Ethos in Ancient Greek Music.” *Journal of Musicology* 3 (3): 264–79.

———. 1999. *Apollo’s Lyre: Greek Music and Music Theory in Antiquity and the Middle Ages*. Lincoln, NE: University of Nebraska Press.

———. 2008. “Greek Music Theory.” In *The Cambridge History of Western Music*, edited by Thomas Christensen, 109–35. Cambridge, UK: Cambridge University Press.

Mcclain, Ernest G. 1974. “Musical ‘Marriages’ in Plato’s ‘Republic.’” *Journal of Music Theory* 18 (2): 242–72.

Mehta, Tarla. 1995. *Sanskrit Play Production in Ancient India*. Delhi: Motilal Banarsidass Publishers.

Meier, Bernhard. 1990. “Rhetorical Aspects of the Renaissance Modes.” *Journal of the Royal Musical Association* 115 (2): 182–90.

Merriam, Alan P. 1964. *The Anthropology of Music*. Evanston, IL: Northwestern University Press.

Miller, Kiri. 2009. “Schizophonic Performance: Guitar Hero, Rock Band, and Virtual Virtuosity.” *Journal of the Society for American Music* 3 (04): 395. doi:10.1017/S1752196309990666.

Moore, Allan. 2011. “The End of the Revival: The Folk Aesthetic and Its ‘mutation.’” *Popular Music History* 4 (2009): 289–307. doi:10.1558/pomh.v4i3.289.

Morphy, Howard. 2005. “Aesthetics across Time and Place: An Anthropological Perspective on Archaeology.” In *Aesthetics and Rock Art*, edited by Thomas Heyd and John Clegg, 51–60. Aldershot, Hampshire: Ashgate.

Nikolsky, Aleksey. 2015. “Evolution of Tonal Organization in Music Mirrors Symbolic Representation of Perceptual Reality. Part-1: Prehistoric.” *Frontiers in Psychology* 6 (1405). doi:http://dx.doi.org/10.3389/fpsyg.2015.01405.

Novik, Yelena. 2004. *Rite and Folklore in Siberian Shamanism: An Experiment in Correlation of Structures [Обряд и фольклор в сибирском шаманизме: Опыт сопоставления структур]*. Moscow: Eastern Literature, Russian Academy of Science [Восточная литература РАН].

Ojamaa, Triinu, and Jaan Ross. 2011. “The Perceived Structure of Forest Nenets Songs: A Cross-Cultural Case Study.” *Psychomusicology: Music, Mind & Brain* 21 (1/2): 159–75. doi:100.1037/h0094010.

Pacholczyk, Jozéf. 1996. “Music and Astronomy in the Muslim World.” *Leonardo* 29 (2): 145–50.

Palisca, Claude V. 1959. “A Clarification of ‘ Musica Reservata ’ in Jean Taisnier ’ S ‘ Astrologiae ,’ 1559.” *Acta Musicologica* 31 (4): 133–61.

Plato. 2013. *Laws*. New York: Dover Publications.

Power, Timothy. 2013. “Kyklops Kitharoidos: Dithyramb and Nomos in Play.” In *Song Culture and Social Change: The Contexts of Dithyramb*, edited by P. Wilson and B. Kowalzig, 237–56. New York: Oxford University Press.

Rags, Yurii. 1980. “The Concept of Zonal Nature of Musical Hearing by N.A. Garbuzov [Концепция зонной природы музыкального слуха Н.А. Гарбузова].” In *Garbuzov N.A. - Musician, Researcher and Pedagoge [Гарбузов Н.А. - Музыкант, исследователь, педагог]*, edited by Yurii Rags, 11–48. Moscow: Muzyka [Музыка].

Rotstein, Andrea. 2012. “Mousikoi Agones and the Conceptualization of Genre in Ancient Greece.” *Classical Antiquity* 31 (1): 92–127. doi:10.1525/CA.2012.31.1.92.

Rowell, Lewis. 1981. “Early Indian Musical Speculation and the Theory of Melody.” *Journal of Music Theory* 25 (2): 217–44. doi:10.2307/843650.

Sachs, Curt. 1957. “Muses and Scales.” In *Essays on Music in Honor of Archibald Thomson Davison by His Associates*, 3–8. Cambridge, MA: Harvard University.

Shakhnazarova, Nelli. 1983. *Music of East and Music of West: Types of Musical Professionalism [Музыка Востока и Музыка Запада: Типы музыкального профессионализма]*. Moscow: Muzyka [Музыка].

Sheikin, Yurii. 2002. *History of music culture of Siberia peoples: comparative-historic investigation [История музыкальной культуры народов Сибири: сравнительно-историческое исследование]*. Moscow: Eastern Literature, Russian Academy of Science [Восточная литература РАН].

Shestakov, Biacheslav. 1966. *From Ethos to Affect: History of Musical Aesthetics from Antiquity to the 18th Century [Музыкальная эстетика западноевропейского средневековья и Возрождения]*. Moscow: Muzyka [Музыка].

Shumays, Sami Abu. 2013. “Maqam Analysis: A Primer.” *Music Theory Spectrum* 35 (2). Oxford University Press: 235–55. doi:10.1525/mts.2013.35.2.235.

Temperley, David. 2011. “Scalar Shift in Popular Music.” *Music Theory Online* 17 (4).

Temperley, David, and Trevor de Clercq. 2014. “Statistical Analysis of Harmony and Melody in Rock Music.” *Journal of New Music Research* 43 (2): (i) – (i). doi:10.1080/09298215.2013.839525.

Thrasher, Alan R. 2008. *Sizhu Instrumental Music of South China: Ethos, Theory and Practice*. Edited by Barend J. ter Haar. Vol. 84. Leiden, Boston: Brill.

Vladyshevskaya, Tatiana V. 2006. *Musical Culture of Ancient Russia [Музыкальная культура Древней Руси]*. Moscow: Znak.

Walser, Robert. 1992. “Eruptions: Heavy Metal Appropriations of Classical Virtuosity.” *Popular Music* 11 (03): 263. doi:10.1017/S0261143000005158.

West, Martin L. 1981. “The Singing of Homer and the Modes of Early Greek Music.” *The Journal of Hellenic Studies* 101: 113–29. doi:10.2307/629848.

———. 1992. *Ancient Greek Music*. New York, London: Oxford University Press.

Widdess, Richard. 1993. “The Geography of Rāga in Ancient India.” *The World of Music* 35 (3): 35–50. http://www.jstor.org/stable/43616472.

Wilson, Peter. 1999. “The Aulos in Athens.” In *Performance Culture and Athenian Democracy*, edited by Simon Goldhill and Robin Osborne, 58–95. Cambridge, UK: Cambridge University Press.

Winnington-Ingram, R. P. 1928. “The Spondeion Scale.” *The Classical Quarterly* 22 (02): 83–91. doi:10.1017/S0009838800000446.

Winter, Irene. 2010. *On Art in the Ancient Near East*. Vol. 2. Leiden, The Netherlands: Brill Academic Pub.

Wright, Owen. 1974. “Music.” In *The Legacy of Islam*, edited by Joseph Schacht and Clifford Edmund Bosworth, 2nd ed., 592. Oxford, UK: Clarendon Press.

Xiaodun, Wang, Sun Xiaohui, and Chang Shijun. 2004. “Yuebu of the Tang Dynasty : Musical Transmission from the Han to the Early Tang Dynasty.” *Yearbook for Traditional Music* 36: 50–64.

Yanov-Yanovskaya, Natalia. 1999. “One Culture Two Traditions [Одна культура две традиции].” *Musical Academy [Музыкальная академия]* 3: 21–27.

Yöre, Seyit. 2012. “Maqam in Music as a Concept, Scale and Phenomenon.” *Journal of World of Turks* 4 (3): 267–86. EBSCO accessory # 84609213.

Zannos, I. 1990. “Intonation in Theory and Practice of Greek and Turkish Music.” *Yearbook for Traditional Music* 22 (1990): 42–59.

Zemtsovsky, Izaly. 1979. “On Creative Nature of Folklore [О творческой природе фольклора].” In *Stylistic Trends in Soviet Music 1960-70s [Стилевые тенденции в Советской музыке 1960-70 годов]*, 137–47. Leningrad: Lenuprizdat.

———. 1983. “Song as a Historic Phenomenon [Песня как исторический феномен].” In *Popular Song: Problems of Study [Народная песня. Проблемы изучения]*, edited by V. Gusev, 6:22–35. Leningrad: Saint Petersburg State Theatre Arts Academy [ЛГИТМИК].

———. 1987. “On Melodic Formula in Russian Folklore [О мелодической ‘формульности’ в русском фольклоре].” In *Ethnographic Origins of Folkloric Phenomena: Russian Folklore [Этнографические истоки фольклорных явлений. Русский фольклор]*, edited by V. Yeremina, 14:117–28. Leningrad: Nauka.

Zemtsovsky, Izaly, and Alma Kunanbayeva. 2011. “Homo Lyricus, or Lyrical Song in Ethnomusicological Stratigraphy of ‘Folk Culture’ [Homo Lyricus , или лирическая песня в этномузыковедческой стратиграфии «фольклорной культуры»].” In *Classic Folklore Today. Proceedings of the Conference "90 Years Anniversary of B.N.Putilov’s Birthday*, 199–249. Sankt-Petersburg: Dmitri Bulanin.

Ziegler, Nele. 2011. “Music, the Work of Professionals.” In *The Oxford Handbook of Cuneiform Culture*, edited by Karen Radner and Eleanor Robson, 288–312. Oxford: Oxford University Press.

1. For detailed explanation of the relation between musical emotion and aesthetic emotion and their role in musical semiosis, see my paper “How Emotion Can Be the Meaning of a Music Work” (2016) at: https://www.researchgate.net/publication/291974302_How_Emotion_Can_Be_the_Meaning_of_a_Music_Work [↑](#footnote-ref-1)
2. The assumption that the idea of “ethos” was invented by Damon, which has won some recognition, is based on assertions made in later literature, which are better regarded akin to the claim that Greeks invented the lyre – as a form of cultural self-promotion (Duchesne-Guillemin 1984). [↑](#footnote-ref-2)
3. The situation started changing with the gradual introduction of mechanical devices, such as sliders and sleeves, employed in aulos playing. Such accessories were becoming progressively more popular from Classical times onward. Together with complex fingering, such techniques enabled large leaps, passages, and abrupt modulation (Hagel 2009, 337), giving aulos a technical edge over string instruments. Remarkably, such change concurred with the shift in the public image of aulos music, which from the 4^th^ century on became the favorite medium for artistic expression in purely instrumental music (Franklin 2013). [↑](#footnote-ref-3)
4. The distinction here lies between Babylonian, Pythagorean, and Platonic authorities *prescribing* a specific tuning as “correct” – to be followed by all the performers – and the Aristoxenian and later music theorists *describing* music practices that they observed in use, and trying to infer a most rational implementation of tuning that would satisfy the need for artistic expression while remaining mathematically coherent. [↑](#footnote-ref-4)
5. Hence, introduction of “reference bells” for concert pitch tuning in Ancient China (Falkenhausen 1992) – notwithstanding evidence for concert pitch tuning practices in Ancient Greece (Hagel 2009, 69). [↑](#footnote-ref-5)
6. “Subscriptive” attitude contrasts both, prescriptive and descriptive, as mentioned above, by reflecting the choice of the performer to *subscribe* to a certain tuning style intuitively, following the perceived need of a particular musical expression. *Subscriptive* tuning approach of the performer compliments the *descriptive* approach of the music theorist. On the other hand, *prescriptive* approach of the performer compliments the *prescriptive* approach of the theorist and/or the music teacher. [↑](#footnote-ref-6)
7. The enharmonic genus is possibly as ancient as the diatonic genus in Ancient Greek tradition (West 1981) – but implemented in a “diatonic” fashion back then: by sticking to the enharmonic degrees and minimizing modulations – in this capacity contrasting the music conceived in chromatic manner. The chromatic genus could have been forged through the experiments with modulation (as explained by Stefan Hagel in personal communication). [↑](#footnote-ref-7)
8. The term “composer” here is used in a generic sense of the author who puts together musical structures into a configuration that did not exist before. In practice, Western notion of “composer” substantially differs from the Central Asian notion of “*bastakor*” – the creator of monodic music works in oral tradition (Yanov-Yanovskaya 1999). This contradistinction can be generalized to speak of two different models of professionalism: Western and Eastern (Shakhnazarova 1983, 18). [↑](#footnote-ref-8)
9. The resultant aesthetic translation of an artwork is perhaps the inevitable link in the ongoing lifecycle of art, where the only living reality is the “now,” and any past experience is evoked in present terms. However, for effective semiosis, it is important for a modern observer to keep the aesthetic contemplation as close as possible to what is known about the parent culture that produced the observed artifact (Morphy 2005). [↑](#footnote-ref-9)
10. It should be noted that chromaticism is not “required” by aesthetic attitude – it merely highlights the importance of aesthetic judgment for a music system. [↑](#footnote-ref-10)
11. It is possible that the *practice* of chromatic modulation could have been forged earlier, and transmitted orally from teacher to pupil. The concomitant of chromatic alteration – aesthetic emotion must have existed by the end of the 3^rd^ millennium BC in Mesopotamia, where certain artifacts were recognized as “artful” and/or “ingenious,” appreciated for their embellishment as a result of the skill in its making (Winter 2010, 2:279). Not surprising, Stefan Hagel infers that the music of Hurrian hymns must have known one type of modulation, achieved by altering F into F# through retuning of the lyre/harp - based on his comparative analysis of frequency of occurrence of the interval names in the Hurrian texts, plotted against Hurrian procedure of tuning (Hagel 2005, 335). [↑](#footnote-ref-11)
12. In the 14^th^ century, there was the genre *kull al-nagham* ("all the modes") designed to pass through different modes so that all 17 tones of the gamut of octave would be used within one piece of music (Wright 1974, 499). Modulations and alterations in maqamat are “regular”: each maqam is characterized by a finite number of common modulations that can be named and catalogued – they all serve the primary function of stressing a new tonicization in a particular way (Shumays 2013). Persian dastgah closely followed maqam, allowing vocal genre of *kolliyat* (literally, meaning “as a whole,” “in its entirety”) to illustrate musically puns with the names of the modes: i.e. accompanying lyrics with prominent epithet of “right” with switching to the mode “*Rast*” (translated as “right, true”), or, accord epithet “large” with switching to the mode “*Bozorg*” (large). As a result, by the 17^th^ century, Turkish *kolliyats* could include up to 30 modulations within a single composition (Feldman 1993). [↑](#footnote-ref-12)
13. This should be regarded as yet another proof of relative rarity of interim modulation within a music work in Babylonian and earlier Mesopotamian cultures. [↑](#footnote-ref-13)
14. It has to be noted that chromatic/enharmonic genera were used mostly by professionals, while everyone else kept using diatonic music. In a few notable exceptions, such as the use of enharmonic music in tragedies, amateur choirs did handle the tonal intricacies, but required much practicing and became an object of competition in Ancient Greek society. Even for the professionals the importance of diatonic music was retained: the typical curriculum of study of music started from the Dorian diatonic mode. Such situation probably contributed to passive admiration of chromaticism by the general public – and cultivation of aesthetic emotions. [↑](#footnote-ref-14)
15. Pythian Games were held every 4 years in Delphi to celebrate Apollo’s victory over Python – as a counterpart to Olympic Games (which also included music in dramatic poetry competition, as well as in trumpet performance) – and featured solo singing with accompaniment of aulos (Kosiewicz 2004). Other important festivals that included music competitions were Isthmian Games held biannually on the Corinthian Isthmus, dedicated to Poseidon, and Nemean Games in Argolia in the honor of Zeus (Christesen & Kyle 2013, 183). [↑](#footnote-ref-15)
16. In Ancient Greece singing was a civic duty in religious ceremonies, festivals, and competitions - in which every citizen was supposed to partake. Ancient Romans inherited the Greek tradition, but as performance became more complex and required higher qualification, professional music performance was left to the slaves (Heimonen 2003). [↑](#footnote-ref-16)
17. It is interesting to note that the rise of the Indian musical tradition, as in the Greek musical tradition, occurred in parallel to the rise of theatric tradition in Sanskrit play, where the typology of the expression of affects on stage most probably influenced the formation of ethos for different ragas (Mehta 1995, 248). Nine Classic *rasas* of Indian traditional theater, dance, and music: namely, love, laughter, fury, compassion, disgust, horror, heroism, and amazement – each, related to a deity and color, seem to present the framework of the aesthetic system quite equivalent to those based on “ethos” (Katz 1996). Remarkably, Richard Widdess points out that the III and VII degrees of non-Vedic Carnatic music were named after the non-Aryan Western ethnicities (*gandhara* and *nisada)*, and were treated differently from the other 5 heptatonic degrees by having an option of being sharpened by approximately a semitone in a manner of an accidental (Widdess 1993). This behavior, “exotic” to Indians (and therefore called by a foreign name), could have been the result of “theatricalization” of the traditional Aryan folk mode. [↑](#footnote-ref-17)
