# Supplementary material for: Evolution of Tonal Organization in Music Optimizes Neural Mechanisms in Symbolic Encoding of Perceptual Reality. Part-2: Ancient to Seventeenth Century
Source: Front Psychol. 2016 Mar 30;7:211. doi: 10.3389/fpsyg.2016.00211 (PMC4813086; doi:10.3389/fpsyg.2016.00211)
Supplement: Supplementary file 3 [file DataSheet1.zip › Appendices I-VIII/Appendix IV. Hemiolic mode as Post-Hellenistic Development of Chromatic System.docx]

# Appendix-IV: Hemiolic Mode as Post-Hellenistic Development of Chromatic System

Despite the lack of historic evidence of origin of the *hemiolic*^[[1]](#footnote-1)^ music, it is quite likely that hemiolic modes, found in modern traditional music of Near East and Central Asia, are descendants of the Ancient Greek tradition (Zannos 1990). Where and how exactly could the transmission have occurred is hard to tell. Pre-Islamic music of Arabs was considerably influenced by the Sabaeans of Harran, especially in transference of the Pythagorean tradition to the “first Peripatetic in Islam,” Al-Kindi (Farmer 1925). Sabaeans were in active trade with Greeks in the 5^th^ century BC, when they adopted the Greek numerical notation system (Chrisomalis 2010, 108). It seems plausible that Sabaeans adopted Greek enharmonic music along with the Dionysian cult, associated with this music in Greece: there are indications that the Sabaic national god Almaqah, represented with vine, related to Dionysus, and tradition of wine drinking while listening to music, similar to Greek *symposion*, must have been common in Sheba (Maraqten 1993). The Sabaean colony was discovered at Al-Ula, Dedan, South-West of Egra, testifying that Sabaeans had cultural presence in the North-West Hijaz during Hellenistic times (Nevo 2003, 68).

The region of Hijaz, on the other hand, is known as the cradle of Arabic musical tradition, producing a school that retained its formative influence well into the 12^th^ century AD, while descending from the earlier pre-Islamic *qaynah* school (Touma 1996, 5–12). The *qaynah* culture is remarkably similar to the Greek *symposion*, with its combination of wine drinking and aesthetic appreciation of secular music, performed on flute, oboe, and lute, by professional entertainers at private parties – which would have maintained the cultural ground for Dionysian tradition, marked from its birth by affiliation with the enharmonic genus. The Hijaz performance school, founded during the 7-8^th^ centuries, probably inspired the choice of the name for the Hijaz mode, since many earliest modes of maqamat were named after geographic regions (Farmer 1929, 203). Hijaz mode was described by Safi al-Din al-Urmawi in 1293, and was mentioned even 200 years earlier (Neubaer 1992, 597).

The Hijaz tetrachord with its augmented 2^nd^, gave life to the entire “Hijaz genre” (using Touma’s expression), represented by 8 modes that are characterized by the descending pattern of 2^nd^s (minor-augmented-minor) leading to the finalis tone, emulating the hijaz mode (Touma 1996, 33).

Hijaz maqam (after Touma): G-A-B¼b-C-D-Eb-F3-G-A-Bb-C-D-E-F ascending, and

G-F-E-D-C#-Bb-A-G-F#-Eb-D descending direction.

This hijaz mode can be regarded as the backbone for the whole Mediterranean region, penetrating most, if not all, ethnic cultures adjacent to it (Manuel 1989a). This “common” modal construct is defined by Peter Manuel as the “Phrygian tonality”, which he considers a product of the interaction of numerous modal traditions of pre-Moorish Spain and Arabic countries and which is expressed by two modes, equivalent to Bayati and Hijaz maqamat:

Hijaz: E - F - G# - A - B - C¼# - D up; and E - D - C - B - A - G# - F - E down.

Bayati: E - F¼# - G - A - B - C - D up; and E - D - C - B - A - G - F¼# - E down.

1. Audio: Maqam Hijaz G-Ab-B-C-D-E¼b(Eb)-F-G <http://bit.ly/21ItSDr>
2. Audio: Maqam Bayati G-A¼b-Bb-C-D-Eb-F-G <http://bit.ly/1RMcK7C>

These two modes seem to comprise a system, where their characteristic melodic intonations can interchange within the same work while keeping the mutual harmonic reliance on the “dominant” sound of the “tonic” E, when the latter appears as though it is supporting the upper A, although A does not receive nearly as much stress as E, which clearly acts as finalis in this music (Manuel 1986). The prototype for such integration seems to come from the practice of modulation from a hemiolic to a diatonic tetrachord/pentachord in maqamat.

1. Audio: Nouba malauf, Tunis. The hemiolic Hijaz mode D-Eb-F#-G-A-B¼b-C suddenly gives way to the diatonic Busalik mode D-E-F-G-A-(Bb)-C. <http://chirb.it/9zcEGr>

In 1951, Igor Sposobin proposed to qualify such modes as a special group of “*dominant modes*” due to the peculiar role of the systemic tonicization of seemingly unstable tone. These modes have relatively weak I degree (tonic) and unusually strong IV degree – hence their collective title “dominant” suggests that their I degree executes the function of “dominant” towards the IV degree. Sposobin listed 4 of such modes, all united by their special feature of comprising a “dominant” relation towards the IV degree minor triad (Sposobin 1969, 101):

1. E-F-G-A-B-C-D-E (minor diatonic),
2. E-F-G#-A-B-C-D-E (hemiolic),
3. E-F-G#-A-B-C-D#-E (double-hemiolic) and
4. E-F#-G#-A-B-C-D-E (major diatonic).

This mutual “minor” coloring of the implied “tonic” chord on the IV degree (A-C-E), along with the inverted tonic/dominant functionality and propensity to terminate the musical sentences/sections with the “Phrygian cadence” (IV6-V), whenever arranged in homophonic fashion, these three factors consolidate all 4 modes into a *single key*.

1. Audio: Solea, Andalusia. The typical example of interchangeability of the melodic tetrachords and triads built in 4 modes: Bb-C-D-Eb-F-Gb-Ab-Bb, Bb-Cb-D-Eb-F-Gb-Ab-Bb, Bb-Cb-D-Eb-F-(Gb)-A-Bb and Bb-Cb-Db-Eb-F-Gb-Ab-Bb. <http://chirb.it/34xHd7>

Any of these 4 modes can easily interchange with another within a section of a musical composition, and even a musical phrase – in a way similar to how the minor key in Western tonality can house 3 modes: natural, harmonic, and melodic. Of course, such “modulations” are most pronounced in the music that is arranged in a homophonic manner.

1. Audio: Hajibeyov – Sensiz (Without you). The dominant key is retained throughout most of the song, with the modes E-F#-G#-A-B-C-D#, E-F#-G#-A-B-C#-D, E-F#-G#-A-B-C-D, E-F-G#-A-B-C-D and E-F#-G-A-B-C-D# (the only exception is a little episode in relative minor C#-D#-E-F#-G#-A-B#). The key signatures in the score indicate E major. <http://bit.ly/1JQlSVg>
2. Audio: Mussorgsky – Nad Rekoi (Over the river). The dominant key sustains throughout the entire composition that is notated in C# major. The modes include C#-D#-E#-F#-G#-A-B, C#-D-E#-F#-G#-A#-B, C#-D-E-F#-G#-A#-B, C#-D-E-F#-G-A-B, C#-D#-E#-F#-G#-A#-B, C#-D#-E-F#-G#-A-B# and C#-D-E-F#-G#-A-B#. <http://chirb.it/Dtm4g0>
3. Audio: Bizet – Andaluza (Entr’acte to Act IV of Carmen). Although the music is notated in D minor, it remains in the dominant key of A from the beginning to the very end, involving the following modes: A-Bb-C#-D-E-F-G, A-Bb-C-D-E-F-G, A-B-C#-D-E-F-G and A-B-C#-D-E-F-G#. <http://chirb.it/2cDKd9>

Three examples above illustrate the use of the “dominant key” in the musical arrangement of traditional Persian poetry (a ghazal by Nizami), an art song of the Western classical music, and a symphonic arrangement of a flamenco tune. They all feature homophonic texture, which highlights the hierarchic vertical harmonic organization and tells them apart from the earlier example of the Hijaz mode. The question whether an orthodox maqam implementation can constitute a “dominant key” should be left for the maqam theorists to answer.^[[2]](#footnote-2)^

What is obvious here though is that whenever the texture becomes more complex than monody, and especially if the chords are used in the musical arrangement, the characteristic features of the “dominant key” perceptually stand out – in music that otherwise might come from very distant cultural traditions. The triads on the I, VII and IV degrees (in the order of reducing functional importance) are the most prominent markers in harmonic progressions in such a key, with the I triad emphasized metrically the most, and the IV triad used sparingly, on weaker metric time, not to overthrow tonicity of the I triad.

*Major* inclination of the I triad is important in stressing superiority of I over IV that carries *minor* inclination: major tonality is known to be more psychologically stable than minor tonality. This has been established by empirical research (Vuvan and Schmuckler 2011) as well as the historic tradition of music theory, ever since Zarlino formulated major and minor triads in 1558 (Lester 1977) – reflected in his choice of titles “major” versus “minor.” The “dominant key” can house the major VI (E-F#-G#-A-B-C#-D in Hajibeyov), but it is usually not supported by the major triad on the IV degree, acting more like a chromatic melodic alteration.^[[3]](#footnote-3)^

Of course, my generalization highlights only the features characteristic for the identity of a dominant key. In reality, the vertical harmonic content in the examples above contains many more harmonies and harmonic relations. Especially Mussorgsky’s romance is extremely rich in generating fresh sounding progressions of vertical harmonies. Bizet’s arrangement also features brief modulation from “dominant A major” to F major, as well as Hajibeyov’s modulation from “dominant E major” to C# minor. Nevertheless, on the whole, the dominant key definitely maintains its own “sound,” identified by ear through its reverted tonic/dominant functionality (shaded by the major/minor contrast) and the prevalent Phrygian cadences, while noticeable absence or near absence of the harmonic progressions alternative to Phrygian. Hajibeyov’s work is the most exemplary of the dominant key functionality, where the harmonic progressions are reduced to rather a single Phrygian formula which functions more like a harmonic “drone” throughout extended portions of the score. Similar implementation can be found in almost all examples of flamenco music.

It seems that the “dominant key” should be considered as integration of two different intervallic typologies in relation to the same modal skeleton – something very similar to the practice of application of different genera onto the same *harmonia* in Ancient Greek theory. In a way very similar to how Greek Dorian tetrachord generated 3 versions:

- diatonic E-F-G-A,
- chromatic E-F-Gb-A, and
- enharmonic E-F¼b-G¾b-A,

the “dominant key” tetrachord generates 3 versions:

- two diatonic - E-F-G-A and E-F#-G#-A, and
- one hemiolic – E-F-G#-A.

This bifunctionality is extremely pronounced in flamenco music. A number of specialists suggest the origin of this bifunctionality to lie in the Greek *systema teleion* – marking its pronounced proneness to the descending harmonic resolution as the indicator of the Greek origin (Shulze and Skiera 1990). The flamenco music could have imported Greek organization through Arabic or/and Roman mediation, both of which left a mark on Spanish culture in opposition to the Ecclesiastic line of diatonic influence (Sanlucar, Whitehead, and Alcantara-Rojas 2011).

Another possible route could have been the crystallization of the Andalusian cadences from the Byzantine liturgical music preserved by the Mozarabic church in Cordoba until the 13^th^ century – a route that crossed Sicily (Thompson 1985), where similar to Andalusian type cadences are found in folk music: such as the diatonic dominant mode in “A la campagnola” song and the melodic dominant mode with chromatic alterations in “Sirbi” (Garofalo 1995). The likely source for such influence was the musical system of the Sicilian-Albanian liturgical repertoire – it is modal and follows the Byzantine theory of oktoechos (Garofalo 2004).

Kliment Kvitka provided one of the most comprehensive reviews of the literature on the distribution of hemiolic modes in his essay “On the descent of chromaticism in the music of Slavic peoples” - tracing modern folk hemiolic modes to Ancient Greece (Kvitka 1971, 1:312–325). His expert opinion on Slavic tradition is of special value in light of a new sociological theory claiming that the hemiolic intervallic type to be of folk Slavic origin (Pennanen 2008), while its author seems to be unaware of Kvitka’s research.

Medieval Arabic treatises demonstrate their authors’ familiarity with Platonic, Aristotelian, and Euclidian music theory (Rosenthal 1966), as well as Pythagorean and Aristoxenian – all of which did cause a following amongst Arabic music theorists (Farmer 1930). Greek genera were appropriated in Arabic music and acquired their own semantic denotations: chromatic was associated with the expression of “generosity, freedom, and courage,” while enharmonic - with “mournfulness, grief, and reservedness,” according to Al-Khwarizmi, 10^th^ century (Farmer 1925).

The idea of unity of astronomic, alchemic and medical fields, where numerical proportions embedded in musical modes would be viewed as having power to affect the listeners’ physiological state, was pivotal for medieval Arabic and Persian views on music (Pacholczyk 1996), and the notion of ethos (ta’tir) still remains very important as of today (Cohen and Katz 2006, 20). It is quite possible that the idea of diversifying the ethos of a mode by means of changing its genus could have attracted Arabic and Persian musicians during Middle Ages, and forged their musical thinking in terms of gapped versus diatonic melopoeia. Following the poor distinction between chromatic and enharmonic genera in Ancient Greek practice (West 1992, 255), Arabic and Persian implementations also could have observed soft transitions between the exact size of the gap, thereby reducing 3 genera to just 2.

Today, still hemiolic structure can be found combined with microtonal inflections of the degrees of a mode, such as in Tminoyo and Shbi'oyo modes of the Syrian chant (Lundberg 1997).

1. Audio: B’utho, Supplication by St. Ephraim, Syrian Orthodox chant, Tminoyo mode. Chromatic alterations with microtonal inflections: A-Bb-Cb-D(Db)-Eb-F-Gb(G)-Ab(A)-Bb-Cb-Db. <http://chirb.it/cvyFOy>

The Greek Orthodox music theory, following the Chrysanthos reform, adopted the tuning standard for the enharmonic genus that was reserved primarily for the 3^rd^ mode – defining it as concisely as the Ancient Greek theorists did in their treatises, although implemented with slight difference, divorcing the ascending and descending varieties of enharmonic genus to two different tetrachords (Chrysanthos and Rōmanou 1973, 105–108).

1. Audio: Kekragion (a short proper troparion), prefaced by the demonstration of the enharmonic and diatonic genera of the 3^rd^ mode. The augmented 2^nd^ constitutes the modal intonation I-II (here Eb-F#), especially in the ascending direction. <http://bit.ly/1Uki5Fn>

However, the exact amount of microtonal adjustments in the practice of modern performers of maqam-based traditions seems to lack strict adherence to any consistent rules formulated by music theorists (Bozkurt et al. 2009). In the tradition of maqamat, the augmented 2^nd^s are usually tweaked by the performers: stretched or shrunken in order to exaggerate the melodic tension or relaxation of the ascending or descending melodic intonation (Marcus 1993). Then, indeed, a musician faces only binary choice of intervallic typology: whether or not to resort to the gapped or diatonic genera.

It is quite reasonable to assume that the hemiolic intervallic typology is a simplified derivative of chromatic/enharmonic genera of Ancient Greek music system, defined along the same line of opposition to a more “normative” diatonic genus. Of course, the semantic affiliations of the hemiolic modes would substantially vary between different cultures. However, hemiolic melic characteristics seem to demonstrate certain uniformity across surprisingly wide array of territories: Andalusia, Maghreb, Levant, Turkey, Greece, Balkan, entire Central Asia, plus transnational Gypsy^[[4]](#footnote-4)^ and Jewish traditions. Peter Manuel proposes a good umbrella term for the “dominant family” of diatonic/gapped modes – calling it “*the Mediterranean tonality*” (Manuel 1989a). This term accurately reflects the distinct opposition of the music produced in the “dominant keys” to that of the Western tonality, the influence of which seems to produce “commercial” forms of the Mediterranean tonality, inauthentic in their artistic expression (Manuel 1989b). In the past, too, the mainstream culture propagated by the Western Catholic Church, found itself in opposition to the music of Mediterranean tonality, such as *Cante Jondo* (Sanlucar, Whitehead, and Alcantara-Rojas 2011). Barbara Thompson also points that the existing flamenco tradition is a joined product of Gypsy, Arab, Jewish, and Indian ethnic cultures (Thompson 1985) – all frowned upon by the Western ecclesiastical authorities.

Certainly, Western influence was one of the factors that shaped the Mediterranean tonality when it started reaching international acclaim in the 18^th^ century: the Hijaz-like melodic intonations, including the microtonal inflections, were placed on the basic tonic/dominant triads of Western music, defining the chordal axis for this music. However, even the explicit use of triads in the accompaniment of hemiolic melodies does not turn such music into a Western tonal key, because its melody is still conceived in a purely modal approach – without any contribution of progressions of “implied chords” which governs melopoeia in Western tonality. Most Westernized works of Arabic or Persian traditional music employ chords in a manner of a *drone* (which is strongly rooted in Mediterranean tradition): as a tonicizing combination of tones, prevalent in the support of melody, only occasionally diversified by some “applied dominant” chord – better described as “an interrupted drone” rather than a progression of functional harmony – where heterophonic organization in texture outweighs homophonic contribution of chords (Nettl 1972).

According to Peter Manuel (Manuel 1989a), the first documentation of Mediterranean tonality came rather late (1830s) and had to do with the growing popularity of the piano home music in Romania, inspiring adaptation of “Turkish” chromatic style to the broken chordal accompaniment (with Alberti figuration) – perpetuated in manuscripts of “linear notation” for piano. Creators of such manuscripts were aware of their “Westernized” style, apparently seeing it as culturally prestigious. Growing popularity of their adaptations probably set the precedence for similar treatment of “Turkish”-style ensemble music, supplying the chromatic melody with chords. International prestige of orchestral music and Western musical instruments must have motivated local musicians to assimilate Western system of chords, which had a “quantization” effect on microtonal inflections. Therefore, more modern implementations of the Mediterranean tonality are often tonally “simplified”: free from micro-chromaticism.

1. Audio: Doina si Sirba, Bucharest, Romania. The hemiolic music in the “dominant mode” D-E-F#-G-A-Bb-C#-D-E-F, with a pronounced drone functionality of the G minor triad, performed by the brass band, recorded in 1905. <http://www.loc.gov/jukebox/recordings/detail/id/7135/>

The ancient opposition of diatonic and chromatic music that sparked such fiery debates in the 5^th^ century BC Athens, seems to have developed into a decided divergence between the Mediterranean and Western tonalities AD.

For a long time, Western European music was isolated from the pool of music where “Mediterranean tonality” had been forming by the “chastity belt” of Christian ideology, extremely hostile towards Pagan professional music, especially instrumental and even more so, chromatic – as St. Clement of Ohrid alarmed against “tenderizing harmonies with their refined decorations, which covertly instill in people’s minds addiction to luxury and dissoluteness” (Shestakov 1966, 97). Clement of Alexandria equated chromatic music with drunkenness and prostitution (Hermas et al. 2007, 2:249) – probably aiming at the association of chromatic genus with music performed at *symposion*. His Logos Christology included a musical cosmology based on the Babylonian/Pythagorean philosophy of the music of the spheres (Cosgrove 2006), restoring it back to its diatonic origins. St. Augustine of Hippo followed Clement’s steps instituting Christian anthem and prayer as principal forms of practicing Christianity – which all promoted diatonic MPS. Greek chromatic intervals were discussed in the treatises on music theory (Herlinger 2002), but left aside in music practice, primarily because of their complexity – until the Renaissance (Atkinson 2008, 258), when influential Vicentino set a goal to restore the chromatic genus for artistic purposes (Maniates 1993).^[[5]](#footnote-5)^

Before the Renaissance, the needs that required more dramatic expression than diatony could afford were usually satisfied by utilizing hypermodal principles (see Appendix-2), materialized in the Guidonian hexachord system. Guidonian organization observed the hypermodal principle of locking B and Bb to different registers in order to prevent their proximity within the same musical phrase, while maintaining the modal integrity of the composition. The idea of fitting a pre-existing tune into that part of the MPS where the intervallic structure of the tune would not demand the alterations of the normative degrees (Bower 2002) reflects concern for modal integrity, typical for hypermodal organization. However, the idea of modulation from mode to mode constituted the opposite trend in Western music, generating chromatic alterations of the accidental type. It looks like the source for this style of harmonic arrangement came from secular art song, which favored specific modes, unlike the contemporary plainchant (where modes were rather evenly distributed within the chant repertoire), and often combined two modes in one tune, sometimes modulating for a number of times (Tischler 1999).

Carolingian art became more permissive towards emotional expression than the Orthodox canons of contemporary Byzantine culture. Subsequently, Gregorian chant made frequent use of modulations and alterations justified by necessity to mark the meaning of the liturgical words (Heckenlively 1900, 52).

1. Audio: “Astiterunt reges terrae,” Antiphon for Good Friday, modulation involving switch from minor 3^rd^ A/C to major 3^rd^ A/C#. <http://bit.ly/1RnOcEQ>

Acceptance of such modulations in otherwise quite rigorous early Christian music could be explained by its peculiar semantic effect that met the preferences of the Carolingian aesthetics. Modulations obscure the gravitational map of the music work, and make the chant appear more of “ethereal”^[[6]](#footnote-6)^ – *free of worldly gravity*. This feature is also expressed in the ecclesiastical architecture of High Middle Ages: in the flying buttress, Gothic arch, and ribbed vault – all as though denying gravity, eager to ascend to the skies – traceable to the 11^th^ century Islamic influences on the Roman legacy, more pronounced on the Italian soil as opposed to the traditional affiliation of Gothic spirit with France (Kluckert 2004). Similar roots, although implemented differently, are evident in the Eastern Orthodox *kalophonic chant* (Stathis 2014), often described in terms of its weightlessness – denial of tonal gravity was apparently regarded as a feature that distinguished the “Heavenly” ecclesiastic music from its “Earthly” secular counterpart (Martynov 1994, 123).

Weightlessness achieved by means of modulation became the preferred manner of the “dispersed” gravitational style adopted by the Western plainchant. Some chants even caused problems of modal identification: in early 10^th^ century, Abbot Regino of Priim, was complaining of some antiphons that started in one mode, continued in another and ended in a third (Hiley 1990). Neither Frankish nor Old Roman repertories seemed to have followed strict modal classification comparable to Byzantine oktoechos. Greater liberty in modulation was to some extent regulated after the 13^th^ century, yet still exceeded much more conservative attitude to modulation and chromaticism in the contemporary Eastern orthodox churches. Overall, Western plainchant remained rather “frozen” in keeping the limits for how far chromaticism was allowed to go – staying immune to advents of “chromatic styles” in Western polyphonic music, and earning reputation of somewhat culturally deficient music comparing to the art music of a time. Eventually, Gregorian chant ended up by defaulting back to its Medieval state through the reform that instituted so-called Solesmes style (Combe 2008).

Eastern plainchant followed diametrically opposite historic path in its attitude towards chromatic music and gravity. By the 19^th^ century most Eastern orthodox churches embraced chromaticism as well as hemiolic modes (Lind 2012, 68) viewing it as restoration of the Ancient Greek roots, along with official adoption of ison (practice of adding a drone part to the melody) in plainchant performance and notation (Koço 2015). Weightlessness achieved by means of melismatic ornamentation and modularity of the melic structures that carry idiosyncratic modal features became the preferred manner of the “intertwining” gravitational style of the Eastern plainchant of the late Middle Ages. Unlike the Eastern Orthodox chant, Western Christianity never allowed the use of decidedly non-diatonic hemiolic modes – taking the stand between the Russian Orthodox plainchant that did not allow any chromaticism at all (Kutuzov 2008, 43–51), and other Orthodox churches.

The system of chromatic alterations in Western music grew anew, from ground zero during early Christianity, driven mainly by the development of polyphony after the 12^th^ century. Its base was laid by the elaboration of “false relation” by reproducing hexachordal-like mutation from different tetrachords (Atkinson 2008, 129). The notion "chromatic" lost its ancient denotation of a genus, and was understood merely as an aberration from the diatonic norm. Music, made without chromatic tones, received the title "*musica recta*" (proper music), and the progressions that involved chromatic tones were referred to as "*musica ficta*" (contrived music), looked upon as an imperfection necessary for writing good-sounding polyphony (Tischler 1973).

While chromaticism, disqualified from genus into accidental (indeed performed by accident, to the performer’s liking), was gradually evolving into what ended up as chromatic tonality by the 17^th^ century, the old-Greek style chromatic modes were reinstituted in the Christian East. In the 13^th^ century, Byzantine chant included an unofficial chromatic mode (Beaton 1980). Ever since the late Middle Ages, Greek secular music was assimilating tonal structures coming from maqamat, and since the 17^th^ century this process started making an imprint on the Orthodox church music (Zannos 1990). Similar development occurred in the neighboring Orthodox Churches. Thus, in Romanian chant the diatonic Second Mode of the Oktoechos E-F-G-A-B-C-D was replaced with the hemiolic M-F-G#-A-B-C-D (Moisil 2011) – with similar substitutions in Bulgarian and Serbian chant.

1. Audio: My soul, Bulgarian chant, Second mode, hemiolic adaptation. Instead of the canonic diatonic G-Ab-Bb-C-D-Eb-F, hemiolic G-Ab-B-C-D-Eb-F is used. <http://bit.ly/1KBegp1>

If “Greek” music style channeled hemiolic music to the Eastern plainchant, “Moorish” style exported it to Spain (Farmer 1963), so that as the new Spanish style gained popularity in the West, so did hemiolic modes – especially through the spread of guitar music (Manuel 1986). Flamenco music that came into limelight in the 18^th^ century exhibits traits that can be traced back to Ancient Greek music treatises (Sanlucar, Whitehead & Alcantara-Rojas 2011), and so do mountaineer’s Balkan cultures – including their affinity with micro-intervals (Petrović 1994). Gypsy music bridged Europe with Indian traditional music (Hornbostel 1975, 1:168) – yet another source of hemiolic intervallic structure and microtonal inflections (Kvitka 1971, 1:326-330). India could be considered the Southern Asian border of the area affected by the Ancient Greek influence, brought by the conquest of Alexander the Great. An alternative source of hemiolic music across the globe provided the Hebrew chant once it absorbed the Greek and Arabic modal principles (Subirats 2006), with its most popular hemiolic “*Ahava rabbah gust*” mode and “*Mi Shebeirach gust*” mode, both constituting the *nusach* of a prayer service (Engel 1904).

The resultant habitat of hemiolic modes is vast: Kvitka (1971, 1:313–316) lists Italian, French, Basque, Danish, Swedish, Norwegian, German, Finnish, Scottish, Polish, Slovak, Romanian, Greek, Hungarian, Ukrainian, Belorussian folk musics as importers of hemiolic modes, not to forget the post-Renaissance classical music.

Hemiolic structure easily coexists with chromatic tendencies due to modulations encouraged by plagal relations, i.e. A-B-C-D-E-F-G#-A (tonic A) in ascending motion, and A-G-F#-E-D#-C-B-A (tonic E) in descending. In his study of Synagogal music, Mikhail Gnesin had examined modes in conjunction with expressive tuning practice, and found that 5 out of 6 most common Hebrew modes often engaged modulations, to the extent that the mode itself could be called “modulating” – and include enharmonic stylization by the use of microtones (Zemtsovsky 2012). Similar oscillations between two modal anchors, each grounding its own subset, with the characteristic satellite chromatic tones, can be found in Balkan orthodox chants.

1. Audio: Holy Spirit, Serbian chant. “Modulating” hemiolic inflections between low C (C-Db-E-F-G-Ab-B-C) and higher F (F-G-Ab-Bb-C-Db) are chromatically enriched (the C subset with F#, and the F subset with A, B, and D natural) throughout this hymn, with both tones used as the finalis. <http://chirb.it/nAhAFg>

Such organization inherits centripetal gravity of *multiple* anchors from Ancient chromatic system. One consequence of habituation to such a system is great refinement of interval detection. Experimentally, it was shown that reference to the chromatic scale prior to the task of identification of melodic intervals increases acuity in interval estimation while reducing tonicity in perceived pitches – in contrast to reference to the diatonic scale (Tsuzaki 1991). Greater acuity leads to greater variability in expressive tuning: performers have an incentive to emphasize intervallic differences between different PC members, so the chromatic genera that feature the richest interval set also have the greatest deviations from standard tuning (Delviniotis, Kouroupetroglou & Theodoridis 2008).

Chromatic coloration can be extremely intense in Mediterranean tonality, serving as the principle means of melopoeia – bypassing the diatonic anchors altogether.

1. Audio: Glory, Through the Prayers of St. Sava, Serbian chant. Chromatic PS: E-F-F#-G-G#-A-B-C <http://bit.ly/1GN1mTs>

What unites Mediterranean tonality with the Ancient Greek chromatic system is reliance on the *clustering principle*: MPS is divided in near-equal-size component subsets (tetrachords/trichords), and each obtains a *pyknon* zone, where the altered tones are squeezed together to generate “abnormal” tension – while leaving the rest of the subset comfortably spaced.

1. Audio: Makam Beste-Nigar on saz lute. This composition features the pyknon (F#-G-Ab ) that plays an important role in the melody by generating much tension in the center of the ambitus: B-C#-D-E-F#-G-Ab-B-C-D-Eb-F#. <http://bit.ly/1LWgVyr>

Majority of examples of chromatic Mediterranean tonality, with or without microtonal coloration, are based on tetrachordal divisions and microtonal intonation-driven alterations of the intra-tetrachordal degrees.

1. Audio: Let Every Breath, Greek chant, Macedonia. Characteristic modal intonations that include major/minor 3^rd^ in two tetrachords, on G and D. <http://bit.ly/1bLW4wT>

There is a line of inheritance that links Mediterranean tonality with non-octave hypermode through the employment of a chain principle of stitching together different modal subsets (trichords, tetrachords and/or pentachords), which can occur in disjunct, conjunct, or sometimes even double-conjunct connection (sharing 2 mutual tones between two adjacent subsets, as it takes place between the Segah, Saba, and Chargah tetrachords in the Beste-Nigar makam of Turkish traditional music). This similarity probably originates from hypermodal and hemiolic organizations each having their common ancestry in the Ancient Greek Perfect System. Whenever the modal subsets turn out to comprise the “false relation” between the subsets’ lower and upper parts, a maqam obtains the characteristic “hypermodal” sound: its upper zone tends to accumulate tension, while its lower zone often houses greater relaxation, most evident in cadences.

1. Audio: Maqam Saba on oud solo. The lowest tetrachord Saba (built on D) overlaps with the tetrachord Hijaz (on F), and receives another disjointed tetrachord Hijaz (on C), forming a 10-tone mode D-Eb-F-Gb-A-Bb-C-Db-E-F, with false relationship between higher and lower D’s (diminished octave) and E’s (augmented octave). <http://bit.ly/1HI1418>

Typical hypermodal elasticity of tension can exist in hemiolic tetrachords chained together.

1. Audio: Cherubic Hymn, Greek chant, First Mode. The increase in tension for ascending melodic motion by the degrees of a non-octave mode that incorporates two hemiolic upper tetrachords: (B)-C-D-E(Eb) + F-Gb-A-Bb(B) + C-Db-E-F. <http://bit.ly/1grBlkn>

However, registral build-up of tension towards the trebles remains a secondary trait of tonal organization for the hemiolic modes, which inside of tetrachords operate very much like the Ancient Greek chromatic genera. The primary energy source for hemiolic melody remains the maximization of tonal tension within a tetrachord: the gapped tones repel each other, while becoming more attracted to the marginal anchor tones. This tendency essentially manifests the same gravitational force as the chromatic modulations in Ancient Greek music: they both take advantage of the heptatonic approach to harmony (Nikolsky 2015) – generating greater tension to obtain greater resolution.

Inequality in distribution of “shaded” and “lightened” zones across the MPS increases anchoring in each subset by stressing subordination relations between its tones. In Ancient Greek music system, *phthongoi kinoumenoi* (“movable” notes - chromatic tones in the middle of a tetrachord) were subordinate to *phthongoi hestotes* (“fixed” - marginal tones), and coordinated to one another; whereas *hegemon* (the highest tone) was considered prime in a tetrachord, which thereby acquired two levels in hierarchy of stability (Kholopov 2006, 71).

Unstable tones were also hierarchically related: upper of them (*lichanos)* could subordinate to another (*parhypate*), especially when both were at the bottom of a modally transposed tetrachord (*barypyknoi*). Such subordination probably originated from greater permanence in tuning of the *parhypate*: it maintained its tuning in both most important genera, diatonic and chromatic, receiving alteration only in enharmonic genus – while the *lichanos* was altered in every genus.

This chromatic hierarchy, once forged by the Ancient Greeks as a modal principle of melopoeia, set the model for both, Western tonality and Mediterranean tonality. Tonal keys are characterized by such organization where less stable non-diatonic tones are attracted to diatonic ones, creating melodic inertia (Krumhansl 1979). The presence of this layer of organization tells tonality from modality (Nikolsky 2015) – whenever a mode executes the primary means of tonal organization in a musical composition, it employs the modal inflections in certain “tendency tones” (melodic intonations that are characteristic for that mode), allowing for the chromatic alterations only in the advanced diatonic MPS systems. However, even in the diatonic MPS, none of the sister-modes embeds a layer of chromatic alterations, which would be subordinated to a diatonic layer. Chromatic alterations remain isolated, each existing autonomously for just a single diatonic degree.

Greek chromatic system was the first known documented music system that featured tonal keys. They must have originated in the diatonic system around the 6^th^ century BC: Dorian, Phrygian, and Lydian keys came first, followed by Hypodorian, Hypophrygian, and later, Hypolydian (Hagel 2009, 7). However, these keys, originally quite similar to modern diatonic keys, became tonally convoluted by the chromatic genera, obscuring the gravitational framework of the diatonic anchors. That must have been the reason for special prestige associated with the chromatic and especially enharmonic musics, and, on the other side of the coin, responsible for their ultimate decline in the Western Roman Empire. To put it simply, chromatic and enharmonic keys turned out to be too complex for audiation, and therefore yielded to simpler diatonic keys. In this process, the chromatic layer of a key became lost in the West.

In the East, however, this chromatic layer could have survived through conversion into the assortment of trichords, tetrachords and pentachords of different intervallic structures: diatonic, chromatic, as well as microchromatic. These “rasterized” chromatic and enharmonic particles turned into “chunks” of melodic information, serving as melodic “bricks” for construction of musical composition. Having a finite assortment of modal subsets did address the issue of audiation, making it possible to process the melodic chunks of various intervallic typology as harmonic filling of a particular gravitational framework. Eventually, after centuries of exercising in audiation of such chromatic and enharmonic filling of a diatonic frame, the new Mediterranean key evolved – where the chromatic and microchromatic layers ended up embedded in the dedicated modal subsets.

At present, Mediterranean tonality is implemented in keys that can feature tonicity on par with Western tonality (extremely strong in *cante flamenco* or in Romanian *doina*), and carry numerous layers of tonal hierarchy. Thus, flamenco implies chords, where root tones constitute a different class than the rest of the chordal tones – also implying non-chordal tones as well. Or, neo-Byzantine oktoechos implies ison, which comprises a discrete layer of anchor tones used as harmonic reference in relation to the tones of a melody. The principal difference of Mediterranean keys from Western keys is that the former do not assign the III and V degrees with tonic function. Instead, they usually stress the gravity of the IV or V degree in a mode through marking the lowest tone of a disjunct or conjunct tetrachord. Their unstable degrees usually have more nuanced intervallic relations and complicated ranking than the unstable degrees in the Western tonality.

Throughout all of its implementations, chromatic music incites greater directionality and complexity in melodic motion, as compared to the diatonic music. But hemiolic chromaticism promotes perhaps the most complex melodic structures due to richer relations between the unstable degrees in a mode and dependence of functionality of degrees on the direction of melodic motion, their disposition in the overall ambitus and their place in melodic syntactic structures (onset, climax, or end points in a phrase).

Such complex system of coordination/subordination charges hemiolic chromatic melody with heavy load of information – heavier than in a typical melody in Western tonal composition (where directionality, registral position, and position in a phrase do not systemically determine the choice of intervallic values). Heavy compression of melodic information makes it cognitively problematic to dress a Mediterranean melody in a homophonic or polyphonic texture in a manner common to Western classical music.

Centrality of vocal monody - melody whose thematic content receives intricate improvisatory elaboration - has become the landmark of Mediterranean music (Blum 2002). Its chromaticism promotes in performers and listeners musical thinking in terms of organicity/inorganicity of specific tones in relation to a given PS. Every modulation necessarily involves a tone that would signal that the previous PS is no longer functional, and the melody proceeds towards some new unknown PS, which the listener should be looking for. Such signal-tones were discussed yet by Cleonides (1^st^ century BC), and are accounted for in maqam which is derivative from Ancient Greek system – as the concept of “borrowed note”: a tone that does not belong to the PS and might supply tonal recoloration (alteration) or serve as a pivot for modulation (Sultan 1988).

Seyit Yöre (2012) suggests to reserve a special term for entrance of such tones – “alienation” – to distinguish them from modulation and alteration. Karl Signell demonstrates how altering a tone in Turkish makam can execute different tonal functions: establishing the exposition section of a melody; inserting a foreign tone (“borrow a tone”) to support or ornament the dominant tone; inserting a foreign tetrachord to diversify and emphasize a passage; stitching different sections together; or generating a compound makam (Signell 1977, 67–112).

Western monody also treated chromatic tones as “alien” to diatonic tones regarding the latter as “natives” in a mode. Gregorian chant shared the same theoretic foundation with the early Byzantine chant in overall rejection of chromaticism and allowing it exclusively in alternations of Bb and B in order to avoid the melodic tritone (Heckenlively 1900, 33) – a tradition that probably dates back to the Babylonian music system, notable for its aversion to tritone (Kilmer and Tinney 1996).^[[7]](#footnote-7)^

However, Western plainchant has stayed quite restrictive in its policies for “legal aliens.” It reserved chromatic alterations mostly for cadential purposes (Homan 1964). In Western secular medieval monody, the use of chromatic alteration was freer – probably due to the absence of religious concern for emotional moderation. Thus, the modes of troubadour and trouvère melodies were frequently modified by chromatic alterations (including also non-B tones, such as C#) for the purposes of beautification (Westrup 1954, 231). Such use should be regarded as a form of intensifying the aesthetic emotion expressed by lyrics.

Starting from the 10^th^ century, rise of polyphony in the West radically altered chromatic practices, introducing new rules that mostly had to do with the technicality of combining multiple melodic lines without creating undesirable harmonic intervals (Tischler 1973). Such technique became known as musica ficta. According to it, essentially, chromatic alteration was viewed as an insertion of a foreign tone into an otherwise harmonious diatonic subset of the MPS – done quite irrationally, following an aesthetic intuition to make the melody more expressive of a certain character (Brothers 1997, 11), and therefore varying from case to case, even between different manuscript copies of the very same composition. From a harmonic angle, all of these forms of chromatic organization supply the creator of music with a wide array of options for conceiving original melodic construction, effective in conveying emotional information via a stock of diatonically based conventions, while embellishing this expression with idiosyncratic nexus of chromatic alterations.

1. Audio: Solage – “Fumeux fume par fume”, rondeau for 3 male voices (c.1370s). Unusually intense chromatic polyphony presents a moody, garrulous, irascible and melancholic character, suggesting the simile between the waiving chromatic melodic lines and heavy smoke that prevents clear vision (Unruh 1983). Just the opening sing-out of the first word “fumeux” uses G, A, Bb, B, C, Db, D, Eb, E, F and F#. <http://bit.ly/1RCskFI>

All in all, it seems that both, Western and Eastern chromatic implementations, share the heritage of Ancient Greek chromatic music in relation to their affinity with expression of greater tonal tension, associated with communicating aesthetic emotions. However, each implementation has elaborated its own angle, directing harmonic organization towards different goals.

Western music focused on the aspect of vertical harmony, trying to forge the laws of optimal encoding of vertical relations between multiple melodic lines. In pursuing this goal, chromatic organization never crossed the lines of diatonic organization – not even in the music that appear the most “chromatic” in its sound, such as most of Gesualdo’s motets, which bombard the listener with alterations that all make sense only within very distinct modal frames (Sabaino and Mangani 2013).

1. Audio: Gesualdo – “Se la mia Morte Brami”, madrigal for 5 voices (1613). The opening sentence “Se la mia morte brami, crudel, lieto ne moro” (if you long for my death, cruel one, happily shall I die) contains 11 tones: G-Bb-D-F-Eb-E-Ab-A-B-C-C#, the 12^th^ tone, F#) appearing just 4 bars later. <http://bit.ly/1OhCLdz>

The diatonic foundation of Western music became challenged only in the XX century by invention of the alternative chromatic methods of organization.

In contrast, Eastern hemiolic modes from their earliest traceable times have presented a *harmonic system alternative to diatonic*. The Eastern Orthodox church documents are explicit about that, defining a separate place for diatonic and chromatic, as well as enharmonic implementations (Lind 2012, 68). Each of them was preferable for each of the official modes in the oktoechos (Scurtu and Tutu 2011).

The historic development of Mediterranean tonality seems to proceed in the direction of greater complexity of intonations laid into the framework of the tetrachordal degrees – abstaining from polyphonic and homophonic textures altogether, and zooming into the expression of a single melodic stream that receives only limited support from the drone tone part and possible heterophonic inflections of the supporting choir (Koço 2015) or an accompanying instrument (Ahrens 1973), possibly an ensemble of instruments – usually driven by the idea of dubbing the melody in parallel 4^ths^ or 5^ths^ (Picken 1953). At any rate, Mediterranean key serves to expose solely one melodic stream, albeit extremely rich in information and often free from patterns of repetition – following the “stream of consciousness” model, most pronounced in the genres of spontaneous vocal improvisation, such as Mawwāl or Qawwāli.

- The hemiolic chromaticism of Eastern cultures nourishes complexity of structures of *horizontal* harmony.
- The diatonic chromaticism of Western classical tradition fuels primarily complexity of *vertical* harmony.

These specializations originate from the gravitational distribution within the modal subset of a MPS:

- Since Western chromaticism was originally accidental in nature, it followed the *trichordal* scheme, where the chromatic degree would be jammed between two diatonic degrees. Such scheme emphasizes the contrast between even and odd degrees, promoting triad induction.
- Since Mediterranean chromaticism presented a permanent form of modal organization, it was more regular, modally determined (by melodic direction or intervallic style – i.e. stepwise vs. leap intonation). It generally followed the *tetrachordal* scheme, where *two* chromatic degrees would be encapsulated between two diatonic degrees.

The pentachordal organization also can take place in Mediterranean modes. Such is *Nawa Athar* C-D-Eb-F#-G pentachord in maqam theory – it lays the foundation of the Nakriz genre (Touma 1996, 34):

Nakriz: B¼b-C-D-Eb-F#-G-A-Bb-C-D-Eb-F#-G up, and G-F#-Eb-D-C-Bb-A-G-F#-Eb-D-C down

1. Audio: Maqam Nakriz A-B-C-D#-E-F#-G-A in this implementation emphasize the V degree and the Hijaz tetrachord B-C-D#-E. <http://bit.ly/1LXJt5q>

However, the tuning practices amongst musicians are not any different in relation to the Nakriz music than they are for the Hijaz music: the tones of the augmented 2^nd^ are often contracted or shrunken, emphasizing the chromatic shading towards the target anchor tone (Marcus 1993). Hemiolic similarity of Nakriz and Hijaz is evident from the fact that they both share basically the same set of PCs (Hijaz from D resembles Nawa Athar from C), and therefore are often paired by modulation (Manuel 1989a).

The position of the hemiolic gap in Nakriz is also enclosed *inside* the subset: Eb does not bear tonic function (unlike the Western C minor of which Nakriz might remind one). Moreover, whenever Nakriz melody receives an accompaniment, it usually displays stronger modal features (often limited to drones) in comparison to the use of chords in Hijaz (ibid). Manuel underlines that the patterns of accompaniment in Mediterranean music do not necessarily mean borrowing of chords of Western music: especially in dance music the musicians are supposed to spontaneously extend the compositions to support the dance, which promotes experimentation by joining together riffs, drones, alternative melodies or passages in contrasting modes and/or tonics. However, in all cases the choice for exact pitch of accompaniment is dictated by melodic features present in the solo melody – and nothing remotely similar to the tonal plan or harmonic progression of Western tonality.

The last issue to point out is that although Mediterranean tonality inherits the tetrachordal design of the Ancient Greek chromatic system, its gravitational maps differ in relation to their treatment of the gap (Katsanevaki 2011). The Ancient Greek gap separated the stable tone from the unstable: the upper tone was permanent in tuning, while the lower tone was changeable. Furthermore, overall, the gravity was directed downwards, promoting descending melodic motion toward the end of a melodic phrase, and associating the low register with resolution, whereas high register – with tension.

The Mediterranean key gap, as a rule, involves two unstable tones. This difference between the Mediterranean hemiolic and Ancient Greek modal organization I identify in Presentation-2 (see the Supplemental files) through acoustic measurements and morphological analysis of the recording in the Thminoyo mode of Syriac chant. That mode is tonicized in Bb and emphasizes melodic gap Cb-D at the bottom of the ambitus, while featuring many chromatic inflections in the upper tetrachords – quite similar to the Hijaz genre.

Both tones of the gap in the Hijaz tetrachord type (low II and high IV degrees) are unstable, and comprise the “leading” relationship with the neighboring stable tones: the upper unstable tone is the ascending “tendency tone”, while the lower unstable tone – the descending “tendency tone.” Both unstable tones then are characterized with flexibility in tuning and strong reliance on the melodic context: i.e. in ascending motion their pitch value is likely to slightly vary from that of the descending motion. Therefore, hemiolic mode potentially contains more tension than the Ancient Greek chromatic mode: hemiolic tetrachord readily supports melodic motion in both directions, and all melodic typology – including wavelike, skiplike, and zigzagging – is likely to generate expressive tuning, thereby emphasizing the detail in melodic context and exaggerating tension and relaxation.

1. Audio: Etude in dorios harmonia minor system in enharmonion genus. Reconstruction of the improvisation in the enharmonic Dorian mode by the Tabouris Ensemble. Relatively low tonal tension. <http://bit.ly/1U6nql0>
2. Audio: Iakovos Protopsaltis - Doxology in Barys Tetraphonos Mode, chanted by Archon Protopsaltis Thrasyvoulos Stanitsas. Strong harmonic tension is evident in this Neo-Byzantine implementation of the chromatic/enharmonic organization: Gb-A-Bb-Cb(C)-D(Db)-Eb-F-F#-G-A#, with 3 hemiolas (low Gb-A, mid Cb-D, and high G-A#) and prominent pyknon (F-F#-G, constituting 3 discrete degrees). <http://chirb.it/NPmMac>

Subsequently, hemiolic tetrachordal organization features greater gravitational diversity, easily subordinating one stable tone to another, just as much as subordinating one unstable tone to another – and flexing the hierarchic relations depending on the melodic direction and intervallic style. Together with the possibility for modulation and alteration, hemiolic mode then constitutes a more dynamic scheme of tonal organization, in comparison to the Ancient Greek chromatic system that was designed as means of rationalizing and simplifying the capricious microtonal intonations of the older enharmonic modes – thereby inheriting the descending tendency of the enharmonic melodic motion (West 1981).

This tendency was apparent to Ancient Greeks, according to the “Problems” by pseudo-Aristotle, where the problem No.33 poses the question: why do the descending melodies sound more harmonious than the ascending melodies – and suggests the answer that the lower tones appear more noble and euphonic in comparison with the higher tone (Aristotle and Mayhew 2011).^[[8]](#footnote-8)^ The fact that the Greek notation system followed the nomination scheme based on a downward alphabetic series leads to believe that the Greeks thought of their scales as descending (West 1992, 192).^[[9]](#footnote-9)^ The descending design is also evident in the production of chromatic and enharmonic genera from diatonic genus *exclusively* by means of lowering the normative diatonic degrees. Helmholtz stresses out that it is the position of the smallest interval in a mode that designates the preferable melodic direction – which for the Ancient Greeks was pointing down (Helmholtz 1885, 286) due to the flattening rather than sharpening of the modal alterations.

The smallest interval acts like a “leading tone” by setting the direction for the resolution of tension (Roederer 2008, 184). Experimental research confirms Helmholtz’ conclusion, indicating that the “leading” directionality originates from the expressive tuning by the performers who as a rule increase the first interval length, and proportionally decrease the last in ascending scales, while inverting this treatment in descending scales, thereby stressing melodic inertia (Delviniotis, Kouroupetroglou & Theodoridis 2008). Prevailing flattening of the diatonic scheme that served as a skeleton for the Ancient Greek music system was likely to set in place the prevailing descending inertia in the Greek tetrachords, marked by what David Huron termed the “tendency tones” (Huron 2006, 160).

Following this logic, the possibility of placing the smallest interval at the top as well as at the bottom of the Hijaz tetrachord, further emphasized by means of expressive tuning that can compress a semitone at either side of a tetrachord (Marcus 1993) would indicate the affordance for both, ascending as well as descending directionality. However, from generative aspect in relation to the alternative maqam tetrachords, the Hijaz tetrachord is likely to favor the ascending direction, since its gap can be produced by sharpening its III degree (i.e. the Kurd tetrachord E-F-G-A modulating into the Hijaz E-F-G#-A).^[[10]](#footnote-10)^ What is crucial here is that the Ancient Greek system favored only one, whereas the Mediterranean tonality easily affords both directions.

However, it should be noted that the hemiolic modes can sound surprisingly close to the Ancient Greek chromatic music. Hemiolic typology does permit to shift the anchoring function from the lowest tone in Hijaz tetrachord to the chromatic semitone underneath it – in the way of the Hijazkar mode (D#-E-F-G#-A-B-C-D#). The resulting structure will strongly resemble the Ancient Greek chromatic Dorian mode.

1. Audio: Lolazorume, Tajikistan. This lyrical song features very consistent modal implementation of two hemiolas, each with its own tetrachordal organization: D#-E-F-G# / A#-B-C-D# - strongly resembling the Ancient Greek Dorian mode. <http://bit.ly/1HJjo83>

On the other hand, the very first use of hemiolic-style gap could have occurred *within the Ancient Greek tradition* as variation of the traditional chromatic shading. The Berlin 6870 papyrus, dated by 156 AD, contains a fragment from an Ajax tragedy, which features a special modal scale with the sharpened VII degree that comprises an augmented 2^nd^ in relation to the degree below it (the “Hijaz”-like tetrachord C#-D-E#-F#) (Pohlmann and West 2001, 58–9).^[[11]](#footnote-11)^ Stefan Hagel (in private correspondence) pointed out yet another occurrence of the Hijaz-type tetrachord in the surviving Ancient Greek compositions: in Athenaeus’ Delphic Paean (D-Eb-F#-G, generated by raising F, although not engaged as a tetrachordal melodic unit - F# and D are mutually exclusive in the vocal phrases).^[[12]](#footnote-12)^ Athena Katsanevaki lists a number of sources that testify that the missing part of book III of “Harmonics” by Aristoxenus, in fact, explained the alternative possibilities for a pyknon to be positioned at the top of the tetrachord or to be split in two small intervals placed on both sides of the gap (Katsanevaki 2011).^[[13]](#footnote-13)^ However, such structures, even if they happened to be employed in a melody, must have been an exception rather than the rule of melopoeia – although the gapped tetrachord of the Aristoxenian theory could have penetrated the folk music practice of the local ethnicities in the Balkan region and Turkey, and prompted experiments in shifting the hemiolic gap in relation to a specific tonal center.

Yevgenii Gertsman underlines that methodologically Byzantine musicology did not break with the Ancient Greek foundation: the music was still processed “atomisticly,” in terms of the smallest element of an intervallic distance between two adjacent tones (Gertsman 1988, 66–72). Once diatonic and shaded intervallic typologies made a notch in music theory and practice, there is no reason to believe that the enharmonic and chromatic genera suddenly seized to exist: if it is plausible that sophistication of their learning could have motivated their avoidance by the indulgent Romans, on the Near Eastern soil, from where Romans used to import slaves skillful in arts, the chromatic style had much greater chances to make an imprint on local traditions.

Early Christians were very tolerant in cultural matters, and whichever intervallic traits had made it into the local cultures were likely to sustain unless they violated the Christian ethos (that took after the Platonic Dorian) (67). The anti-chromatic rhetoric materialized into the Church policy only in the 7^th^ century AD (Meshscherina 2000, 79) – leaving enough time for the hemiolic typology to obtain solid ground in folk music. A glimpse into such music is provided by Christodoulos Halaris in his album “Akritika,” which is the paramilitary force formed out of militaristic ethnicities from Isauria, Pisidian mountains, and Gothograeci from Asia Minor, who were granted privileges by the Byzantine authorities in exchange for protecting the borders from Arabs.

1. Audio: “A Border Guard was Building a Castle,” Cappadocian traditional song, arranged in the Byzantine style to represent the Akritic music of the 9^th^ – 14^th^ centuries AD. The song is based on a formula that alternates between hemiolic and diatonic modes: G-Ab-B-C-D-F and G-Ab-Bb-C-D-F. <http://bit.ly/1V0wabI>

All things considered, it seems that there is less difference between the Mediterranean hemiolic and Ancient Greek chromatic music than between the Mediterranean and Western tonalities. Just as much as the Western key embeds a bulk of information on the intervals, chords, and their typical progressions and distribution between voices/parts, the Mediterranean key embeds a bulk of information on the tetrachords, trichords, and pentachords available for melodic development at different positions in the ambitus, different direction of melodic motion, different position in a music form, and different rhythmic pattern. This music strongly relies on modular design, most well defined in the compositional practices of maqam (Simms 2003, 11) – with substantially greater share of re-using pre-existing melodic material, both of micro-level (common melodic intonations) and macro-level (common turns for melodic phrase), in comparison with Western tonality. Compositional modularity is most evident in the genre of *nawbah* (suite), where motifs are often combined to form 3-, 4-, and 6-sided modules in symmetric designs that is strikingly close to the calligraphic diamonds or hexagons so common in ornamental decorations in architectural interior design and book illustrations (al Faruqi 1985).

Western art of composition was never driven by such ornamental geometry. Western artists found inspiration in much more global implementation of geometry in the form of proportionality in axonometric projections, such as the “musical proportions” advocated by Alberti, which then were applied onto large-scale proportions of sections of music form, taking advantage of visual representation of the score on a cartella (Reynolds 1987). Or, the architectural proportions could have been taken as the base for the polyphonic arrangement of parts, such as Guillaume Dufay reproduced the proportions of the new Cathedral of Santa Maria del Fiore in a motet composed for its opening (1436) (Trachtenberg 2001). Composition of that sort relies on the deduction logic, where the details of the composition are inferred from a general principle:

- Composition of Western tonality is conceived from general to particular (with the help of notation) resulting with the music structure that is considered optimal and finite in presentation of a subject, and expected to be faithfully reproduced by different performers;
- Composition of Mediterranean tonality is conceived from particular to general (through spontaneous improvisation) resulting with the music structure that is considered one of possibilities of presenting a subject, not to be reproduced by different performers.

Yet another important difference is that Western tonal composition relies to a much greater extent on configuration of the micro-level melodic structures in order to produce a *novel* sounding macro-structure.

Demand for originality has had formative influence on Western concept of authorship and “invention” from Renaissance times (Long 2003), and originality of musical themes has been a determinant factor in acknowledgement of Western composers from J. S. Bach onward (Simonton 1980). The entire course of 16-20^th^ century development of composition practices reveals progressive reduction of productivity in reverse proportion to increase in originality (Kozbelt 2008). The emergence of the composer-centered “work-concept” as the primary means of aesthetic evaluation of Western art music (Talbot 2000) concurred with the emergence of tonal keys in the 18^th^ century, and this was not a mere coincidence. A Western composer was expected to design a musical space according to the science of music in order to convince the listener in reality of the musical emotions – this task at least partially resembled activity of an engineer, and entitled the author with the right of ownership of his intellectual property. In 1739 Johann Mattheson expressed this view in his advices to a good Kapellmeister, where he justified borrowing of someone else’s material only if it was returned “with interest” - i.e., “one must so construct and develop imitations that they are prettier and better than the pieces from which they are derived” (Mattheson and Harriss 1981, 298).

Modularity of maqam and similar to it Mediterranean traditions simply do not provide sufficient means for a composer to invent anything “original” – exuberance of phrases and motifs available for elaboration makes the culture of music-making here somewhat “verbose”: in a way similar to how a very excited person says a lot more than necessary in order to make a point – plus an old tradition of eloquence prevalent in Persian and Arabic literature, where generous quoting is regarded as a virtue. In this tradition, musical material is thought of as “utilitarian” – meant to be utilized by anyone needing it. In fact, the verbose character of this culture is directly related to the microchromatic versatility of its modes: Sami Shumays testifies that he himself uses at least 12 tones between his lowest in tuning Eb and his highest E, depending on which mode is currently active in the melodic development (Shumays 2009). And what supplies him in knowing what tuning is right is his memory of the melodic models for each of the modes – Shumays compares melodic models with the glossary of words, remembered in correct pronunciation, and reports that in Egypt and Syria he has encountered many ordinary people who could sing hundreds of art songs in “expressive microtuning” of the corresponding mode. Wide adoption of high standards modal erudition can be compared with the spread of expertise in lyric poetry, when a reader can easily come up with hundreds of quotes from his favorite poets.

What is “original” in this type of music-making is not the realization of a particular “*design* of a virtual musical space,” but *style* of joining the compositional elements together – in essence, a personal interpretation of a known mode (or group of modes) with its characteristic modules presented in the “right” pitch, rhythm, and form (Nettl 1974). Then reproduction of the same mode by the same musician would constitute the “same” composition even if the recorded scores of the performance would reveal substantial structural differences.

Much of this compositional difference between Western and Mediterranean tonalities originate in their gravitational differences. Unfortunately, it is hard at this point to define the exact discrepancies in their tonal organization because of their influence on each other (Zannos 1990) and the historic proximity that obstructs identification of their characteristic tonal traits. There are plenty of compositions of Western tonal music arranged to represent “Spanish,” “Jewish,” “Gypsy,” or “Eastern” music, which quite closely reflect the modal characteristics of the Mediterranean tonality (see Bizet’s example in the beginning). On the other hand, any attempts to arrange Mediterranean music for a band are likely to involve some kind of Westernization of harmony. Such “marginal” music literature considerably blurs the distinction between what constitutes Western “classical music” tradition, and what – a “Mediterranean” core. However, as a whole, a couple of important observations can be made:

- The *trichordal alteration* scheme of the Western tonality promotes triadic vertical harmony and generates complex vertical hierarchy of stable and unstable tones – supporting the duophonic thinking in conception of music, prominent in Western compositional practices from the 15^th^ century on (Moll 2014), and still present in homophonic textures, which are usually conceived as melodic voice versus bass, with some harmonic filling (Hindemith 1942, 113).
- The *tetrachordal alteration* scheme of the Mediterranean tonality promotes modular melodic organization and generates complex horizontal harmony, while reducing vertical harmony to the bare minimum – supporting monophonic thinking in conception of music, which remains faithful to the textural approach instituted by the MPS cultures of Antiquity.

The outcome of vertical hierarchic relations is that both, tension and relaxation can form different gravitational schemes at different layers of texture. For example, the tones in a chord can employ one set of relations, where the root tone would outweigh the other tones, while in the bass line this root tone would constitute a very unstable tone, whereas the top tone of this chord would constitute a stable tone in the melodic line.

1. Audio: Brahms - Piano Quartet in C minor, opus 60, second movement, Scherzo. Visualization by Stephen Malinowski demonstrates the distribution of the stable and unstable tones across the texture by means of harmonic coloring: using the color wheel to represent the circle of 5^ths^ (blue-to-orange representing tonic-to-VII, and yellow-to-green to represent the chromatic alterations). The listener has to continuously renegotiate the stability and instability values for all the voices and parts of rather complex texture. <http://bit.ly/1LwvmUw>

Cultivation of such frame of reference would develop the skill to simultaneously process parallel streams of information, where a single tone could receive different gravitational values in different textural layers. Theoretically, consistent exercising of this capacity is likely to promote the ability to quickly estimate the arrangement of multiple objects from a particular angle, and facilitate such operations as axonometric projections.

Processing of Mediterranean tonality does not cultivate such a capacity. Instead, it cultivates erudition of melodic intonations, motifs, and phrases, as well as the knowledge of basic modal units for melodic and rhythmic construction of music. In this scheme of listening, attention focuses primarily on the horizontal aspect of organization, assigning the cognitive resources of the listener to span over longer durations of music form: thus, in Persian dastgah the relationship of the components in a *gusheh* (a section of a dastgah with its specific theme and ambitus) often resembles the relationship between the *gushehs* in the entire composition (Nettl 1974). Improvisation within the Mediterranean traditions can involve large building blocks as well as medium and small sizes. Producing and consuming this type of music is likely to develop a singular view, zooming into the longitudinal disclosure of melodic events.

There is experimental evidence of different strategies employed by Western and Arab listeners in perception of tonal organization in the taqsim (modal instrumental improvisation) (Ayari and McAdams 2003). Western musically trained listeners (performers and musicologists) do not perceive the hierarchical melodic organization of taqsim, which can result in not hearing modulations and modal elaborations: in such cases listeners are aware of the presence of some kind of development of melodic microstructures, but are incapable of perceiving them independently. Arab musically trained listeners basically succeed in detection of the melodic organization in the taqsim, and differ from Western listeners by focusing on *evolution* of the improvised ideas throughout the composition: grouping together the detected small modal images into lengthy sequences, and capturing the manner in which the large-scale musical ideas are presented in the taqsim. Ayari and McAdams identify 5 prevailing listening strategies:

1. discrimination of pitches, intervals, and ornaments based on their contribution to tension or stability;
2. seeking a coherent link between the tones to form syntactic sequences appropriate to each phase of the development in music form;
3. identification of microtuning that marks modal functionality of a tone in a modal cell, and relation of it to the microtuning in the previous cell;
4. reducing a set of similar units to identify the modal core and generative root, recognizing a particular color and general air of the modality;
5. tracking the dynamic progression of the phrase to establish coherence between phrases and distinguish the sequences that belong to the same mode but differ in modal detail.

Evidently, this approach to comprehension of a musical composition is characterized by detection of the larger-scale changes and identification of familiar modal structures and microstructures. Focusing on the longitudinal relations of motifs and intonations demands a combination of great attention to the tuning detail and great memorization skills.

To answer such needs a special listening style has been forged amongst connoisseurs of the Middle Eastern music. Gilbert Rouget describes it as the attentive and concentrated listening aiming to attain a kind of illumination of divine contact – a sort of trance with heightened mental awareness (Rouget 1985, 270). This listening style is not limited to Sufi music alone, but covers the Islamic music in general, stemming from the Pythagorean roots, as established by al-Farabi, according to which music is a spiritual revelation (not necessarily religious) of cosmic energy, charged with some ethos, and therefore requiring a special treatment on the part of both, performers and listeners (Leaman 2004, 106). The notion of *tarab* – something akin to ecstasy from being overwhelmed by a particular affect evoked by an art object, usually aural – is central in this culture, equally applying to secular and sacred music (Racy 2004, 12). Tarab refers both, to a class of music designed to produce an affect and to the effect produced by such music – which makes tarab into “musical elation” – musical, since it is usually triggered by hearing of a mode or of an interaction of melody with a metric pattern (136). Tarab can also be regarded as a bliss, achieved by intense mental fixation on a particular musical mode (134). The performers are supposed to generate this fixation in themselves and then pass it on to listeners, who can be affected to the extent of being possessed by a tarab of a particular tune for days (Shannon 2013).

Transmission of tarab is definitely a phenomenological reality in the practice of traditional Arabic music, reflected by the concept of “*saltanah*” – the state of self-absorption: initiated when a musician captures the innate feeling of a musical mode in his improvisation and communicates it to the audience (Racy 2004, 123). The latter is looking forward to hear the presence of *saltanah*, letting musicians know once *saltanah* is detected. The state of tarab in the audience during listening is highly visible and audible (including jumping, clapping, dancing, moaning or sighing, and verbally exclaiming), and directs the performers in their choice of development in the improvised composition (Cosentino et al. 1987, 23).

Tarab, in fact, constitutes the aesthetic core that supports the stalk of Egyptian, Saudi, and Syrian musical traditions, advertently or inadvertently providing the point of reference for all the neighboring branches of the Mediterranean tonality. The stronger the tarab of a musical performance, the higher esteem for its performers. Absence of the tarab discredits the music. Tarab sets very direct criterion for music appreciation that can be easily utilized over different genres and types of music. As a peculiar type of aesthetic emotion, tarab can be remembered, reproduced, and mentalized by a person who is experiencing it. Communication of tarab through a musical “glossary” of folk melodic intonations and motifs then becomes a simple test in administration of aesthetic judgment – in stark contrast to a very complex “science” of aesthetic judgment in the tradition of Western classical music (Scruton 1997).

Tarab provides a rather stable and long-living reference tool: it is supported by the tradition of Qur’anic chanting - as long as recitation of Qur’an remains the axis of international devotional practice of Islam, there is no reason to expect any decline of the Tarab culture (Racy 2004, 224). In contrary, the advance of modern technology allows for wide distribution of Tarab music and popularization of records of the most expressive Tarab artists who are now deceased. Cultural globalization offers channels for any music, even the one that is not that popular in its local vicinities to win public acclaim in distant parts of the world via network of festivals, clubs, TV, internet (Shannon 2003). Tarab music wins international audiences and starts becoming a multi-cultural phenomenon. Thus, Tarab musicians cooperate with Jewish musicians in their album productions in Israel (Brinner 2009, 158). Jonathan Shannon reports that in Syrian city Aleppo, a renowned multicultural musical center of the Middle East, tarab plays an important role in Syrian “aesthetics of authenticity” that sets the cultural standard of being different from the West – what Syrians call “oriental spirit” (Shannon 2013). Products of other cultures are validated according to this standard: i.e. the Arabo-Andalusian music produced by the Spanish musicians is judged by the Syrian audiences in reference to the presence or absence of tarab in the auditioned performance to indicate whether the music belongs to “oriental” or Western domains (Shannon 2007).

Tarab is not alone in the coalition of Mediterranean musics. Spanish flamenco has forged something quite similar to tarab known as “duende” (soul). Great Spanish poet, Federico Garcia Lorca, who also is renowned for his collaboration with Manuel de Falla in the research of Andalusian folk music, saw duende as the principal aesthetic feature that distinguished Spanish *cante jondo* as the cultural archetype. Lorca cited an old maestro of guitar: “the duende is not in the throat, the duende climbs up inside you, from the soles of the feet,” explaining that duende is “not a question of ability, but of true, living style, of blood, of the most ancient culture, of spontaneous creation” (Lorca 2002, 263). This “duende” is clearly in polar opposite to the classicistic norms “dictated by the muse” – forming an alternative to style, which in Christopher Maurer’s words: “appeals to spontaneous understanding, with little, if any, conscious effort” (Lorca 2010, X). In his lecture “The Historical and Artistic Importance of Primitive Andalusian Song known as *Cante Jondo*” (1922) Lorca considered chromatic irregular siguiriya, with its dominant “Phrygian” key, to constitute the oldest *cante jondo* form, preceding flamenco, and originating from Byzantine chant with the catalyst influence of Moorish and Jewish musics after the 8^th^ century, ultimately shaped by the Gypsy contribution in the 15^th^ century (Walters 2007). The notion of duende seems to come indeed from the old-time flamenco authorities: thus, the legendary singer, Manuel Torre (1878 – 1933), from the Jerez school, regarded duende as “black sorrows” (*penas negras*) in a sense of personal musical emotion (Thompson 1985).

The historic paths of Western and Mediterranean tonalities diverge in promoting quite different philosophies:

- Vertical harmony in textural organization of Western tonal composition and in implied chords and chordal progressions of the melodic line, cultivates tonal hierarchy that assigns different stability/instability values to the tones detected in a single slice of the musical texture – including different values assigned to the very same PC (i.e. as a voice in a chord and as a member of a melodic phrase) – which requires somewhat “pluralistic” outlook from the listener to perceive an instance of a tone in a few “hypostacies” simultaneously – as well as high speed of auditory scene analysis;
- Horizontal harmony in primarily monodic organization of “Mediterranean tonal” composition cultivates tonal hierarchy that assigns only one stability/instability value at a time to every melodic tone (however, renegotiating this value for every subsequent occurrence of this PC, depending on the reference to the repertory of modal intonations and motifs) – which engages “monistic” outlook, with no need in instant segregation of audio streams, but heavy demands on memory and erudition.

If the Western listener gets used to quickly grasping many different pieces of information, which can be paradoxical to one another, and momentarily sort them out to fall in different classes, the “Mediterranean” listener gets used to zooming into one stream of information and holding in memory the melodic details over very long spans, continuously relating detected melodic sequences to the known entries from his “musical glossary.” This difference in listening corresponds to the difference in composition. Western composers design music to reflect multiple states at the same time – most obvious in polyphonic genres, but evident in homophonic textures as well (i.e. the Quartet “Bella Figlia Dell'Amore” from Rigoletto by Verdi, where each of the participants expresses his/her own state). Creators of “Mediterranean music” design works of great melodic complexity that may involve intricate symmetric arrangement of motifs (al Faruqi 1985) and reach a gigantic size, where a single composition would last for more than 6 hours (Pacholczyk 1993).

The aesthetic emotions in each of these types of music also differ: Western tonality promotes *emotional theater* in a manner of a movie: even in the miniature genres like song it is common to represent the mise-en-scène of a particular emotional state, event, or situation, where the spectator observes them “from aside,” aware of the theatric “fakeness.” Mediterranean tarab places the spectator right in the same frame as the musicians generating aesthetic emotions – the spectator receives “the real thing,” which is not realistic in a “mise-en-scène” sense, but its abstracted affectation engages the most direct action on the listener.

Therefore, the Mediterranean listener takes musical things “for real” – and that could be the divider between the recent exacerbation of cultural conflict between the values of Western and Islamic lifestyles. Different cognitive schemes embedded in Western tonality could foster pluralism and develop the ability to critically examine a situation from multiple angles before inferring an optimal approach to that situation. The fixedness and directedness of the listening style, cultivated by the Mediterranean tonality, might be responsible for the institution of a cognitive scheme based on commitment to a single point of view, and estimation of long streaks of information in relation to this sole aspect – all taken with passion. The decided opposition to the Western theatricality that is most pronounced in the art produced within the Catholic tradition, provides a common ground for *tarab* of maqam, together with its derivatives, *duende* of flamenco, and *hesychasm* of Orthodox music. Although *hesychasm* made its reputation as an ascetic instrument of diverting attention from senses to an inward seclusion to establish an experiential connection to God, its technique of focusing on nothing but the ultimate reality of God makes *hesychasm* essentially a feeling – a feeling that is supposed to replace all thoughts (Ware 2000, 102). Such a powerful feeling often takes the shape of elation or ecstasy, quite similar to the experience of tarab while listening to the recitation of the Qur’an. Orthodox authorities, like St. Maximus the Confessor, are explicit in that emotions and affections are not to be excluded from Christian worship for constituting an organic part of human nature, and that Christian prayer, in fact, “should be animated by *eros*, intense and fervent longing for the Divine, so that our worship becomes truly an expression of erotic ecstasy” (62).

St. Gregory Palamas, the chief theologist of hesychasm, taught that during prayer, our faculty of direct spiritual awareness exalts the flesh to “commune together with the soul in the Divine” (63). The music of the prayer was supposed to animate the words and fill them up with emotions to reach the state of “worship of the mind in the heart” (64). The *kalophonic chant* was designed to satisfy such need for mystic exploration into the mysteries of the Heavenly world (Stathis 2014, 56–70). Elaborated during the 12-13^th^ centuries in Byzantium, it spread to the neighboring Orthodox churches. Even in Russia, where ecclesiastical music has always remained strictly diatonic, the kalophonic chant maintained the characteristic Byzantine trait of refined “intertwining” of the generously embellished melodic tropes, elaboration of which ran quite autonomously from lyrics, presenting a peculiar “intertwining” of the textual and melodic elements – displaying strong ornamental properties (Martynov 1994, 114). Musical ornamentation here reflected the same tendencies as Balkan geometric ornamentation found in the architecture of Greek Orthodox churches and in the manuscript illuminations. The pronounced opposition of such geometric ornamentation to a supposed “illusionism” of the Western art occupies an important place in the rhetoric of the Orthodox aestheticians (Florensky 1996). Its roots date back to the 8^th^ and 9^th^ centuries iconoclast movement in Byzantium, inspired by the influence from Islam and Judaism (Brubaker and Haldon 2011, 264–272).^[[14]](#footnote-14)^

It should be underlined that cosmological aspect of “music of the spheres” has remained the property of solely Islamic and, to a lesser extent, Eastern orthodox musical traditions. Eight principal modes of Byzantine oktoechos and all of its derivatives are integrated into an 8-week liturgical cycle, so that chants in the 1^st^ mode are sung throughout the 1^st^ week, chants in the 2^nd^ mode – through the 2^nd^ week, etc. – thereby, observing the calendar order. The West never adopted this arrangement – not even when its authorities became set on the idea of borrowing the oktoechos system from Byzantine in the 9^th^ century (Hiley 1990).

The adherence to the principles of musical ethos in modal organization, together with modularity and ornamentalism of the motivic organization in a monodic texture that entirely focuses on the long-term span of a music work characterize both, the domain of maqam/dastgah and the domain of Eastern Orthodox music. Their other common trait is found in the verbosity of both cultures. Just as maqam music requires extensive erudition in common “sayings” by different music masters in each of the known modes in order for a musician to come up with “good” music (Shumays 2009), kalophonic emancipation of melody opens a new period in history of Orthodox liturgical music – a period characterized by the spread of “musical lexicon” treatises, such as the “Chironomic singing exercises” by John Koukouzelis, that listed the modes, tropes, melodic figures, and provided their explanations and music illustrations (Martynov 1994, 60). Modular music form, ornamental melic design, and “verbose” expressive tuning of musical idioms, together, support a manner of expression that is distinctly different from the Western theatricality of classical music.

Hemiolic modes could have played a principal role in supporting the functional opposition to the diatonic modes within the cognitive framework of Mediterranean tonality – providing the most obvious antinomy to Western music (whose diatonic modes are quite similar to the diatonic modes of Mediterranean musics). Yet another important reason for salience of hemiolic interval typology could be its heightened ethos. According to Racy, Hijaz has a reputation of one of the most important and most ecstatic modes in the Eastern maqamat: “a mode that enters into the make up of all other modes” (Racy 2004, 108). Racy reports of the expert musicians emphasizing the critical importance of microtonal adjustments in the intervallic structure of the Hijaz mode for the tarab effect to take place: prohibitive (not to lower the II degree above the tonic excessively), as well as prescriptive (to flatten the III degree by one comma while sharpening the II degree by another comma). Perhaps the hemiolic intervallic typology is there to embody the historic transition from a one-point alteration to a two-point alteration, identified for the Greek region by Athena Katsanevaki (Katsanevaki 2011). Two-point adjustment of the gapped tones in a hemiolic “dominant key” could be exactly the binding agent in pairing of the hemiolic “dominant” key with the diatonic “dominant” keys, so common in flamenco, folk music of the Mediterranean region and the Arabic, Persian, Turkish, and Gypsy art music. The “normal” levels of the emotional expression could be handled by the diatonic genus, whereas the heightened levels would prompt musicians to increase the size of the “alteration zone,” thereby exponentially increasing tension.

REFERENCES:

Aarden, B.J. 2003. “Dynamic Melodic Expectancy.” Ohio State University.

Ahrens, Christian. 1973. “Polyphony in Touloum Playing by the Pontic Greeks.” *Yearbook of the International Folk Music Council* 5: 122. doi:10.2307/767498.

al Faruqi, Lois Ibsen. 1985. “The Suite in Islamic History and Culture.” *The World of Music* 27 (3): 46–66.

Aristotle, and Robert Mayhew. 2011. *Problems*. Vol. 1. Cambridge, MA: Harvard University Press.

Atkinson, Charles M. 2008. *The Critical Nexus: Tone-System, Mode, and Notation in Early Medieval Music*. Oxford, UK: Oxford University Press.

Ayari, Mondher, and Stephen McAdams. 2003. “Aural Analysis of Arabic Improvised Instrumental Music (Taqsm).” *Music Perception* 21 (2): 159–216. doi:10.1525/mp.2003.21.2.159.

Beaton, Roderick. 1980. “Modes and Roads: Factors of Change and Continuity in Greek Musical Tradition.” *The Annual of the British School at Athens* 125: 1–11.

Bharucha, Jamshed J. 2002. “Neural Nets, Temporal Composites, and Tonality.” In *Foundations of Cognitive Psychology: Core Readings*, edited by Daniel Levitin, 455–80. Cambridge MA: Bradford Books MIT Press.

Blum, Stephen. 2002. “Hearing the Music of the Middle East.” *The Garland Encyclopedia of World Music*. USA: Garland.

Bower, Calvin M. 2002. “The Transmission of Ancient Music Theory into the Middle Ages.” In *The Cambridge History of Western Music Theory*, edited by Thomas Christensen, 136–67. Cambridge, UK: Cambridge University Press.

Bozkurt, Barış, Ozan Yarman, M. Kemal Karaosmanoğlu, and Can Akkoç. 2009. “Weighing Diverse Theoretical Models on Turkish Maqam Music Against Pitch Measurements: A Comparison of Peaks Automatically Derived from Frequency Histograms with Proposed Scale Tones.” *Journal of New Music Research* 38 (1): 45–70. doi:10.1080/09298210903147673.

Brinner, Benjamin. 2009. *Playing across a Divide: Israeli-Palestinian Musical Encounters*. Oxford University Press. https://books.google.com/books?id=A2MlJa193Z4C.

Brothers, Thomas David. 1997. *Chromatic Beauty in the Late Medieval Chanson: An Interpretation of Manuscript Accidentals*.

Brubaker, Leslie, and John Haldon. 2011. *Byzantium in the Iconoclast Era, C. 680-850: A History*. Cambridge, UK: Cambridge University Press.

Chrisomalis, Stephen. 2010. *Numerical Notation: A Comparative History*. Cambridge University Press.

Chrysanthos, and Kaitē Rōmanou. 1973. *Great Theory of Music by Chrysanthos of Madytos*. Translated by Kaitē Rōmanou. Bloomington, IN: Indiana University.

Cohen, Dalia, and Ruth Katz. 2006. *Palestinian Arab Music: A Maqam Tradition in Practice*. Chicago, IL: University of Chicago Press.

Combe, Pierre. 2008. *The Restoration of Gregorian Chant: Solesmes and the Vatican Edition*. Translated by Theodore Marier and W. Skinner. Washington DC: Catholic University of America Press.

Cosentino, Donald, John Kennedy, Douglass Price-Williams, Jihad Racy, and Johannes Wilbert. 1987. “Trance, Music and Music/trance Relations: A Symposium, UCLA, June 3, 1987.” *Pacific Review of Ethnomusicology* 4: 1–38.

Cosgrove, Charles H. 2006. “Clement of Alexandria and Early Christian Music.” *Journal of Early Christian Studies* 14 (3): 255–82. doi:10.1353/earl.2006.0049.

Cuddy, Lola L. 1997. “Tonal Relations.” In *Perception and Cognition of Music*, edited by Irène Deliège and John A. Sloboda, 330–52. Hove, UK: Psychology Press.

Delviniotis, Dimitrios S., Georgios Kouroupetroglou, and Sergios Theodoridis. 2008. “Acoustic Analysis of Musical Intervals in Modern Byzantine Chant Scales.” *The Journal of the Acoustical Society of America* 124 (4): EL262–69. doi:10.1121/1.2968299.

Engel, Joel. 1904. “Jewish Music [Еврейская музыка].” *Dictionery of Music*. Russia: P. Jurgenson.

Ewell, Philip A. 2012. “Rethinking Octatonicism: Views from Stravinsky’s Homeland.” *Music Theory Online* 18 (4).

Farmer, Henry George. 1925. “The Influence of Music: From Arabic Sources.” *Journal of the Royal Musical Association*.

———. 1929. *A History of Arabian Music to the XIIIth Century*. London: Luzac And Company.

———. 1930. “Greek Theorists of Music in Arabic Translation.” *Isis*.

———. 1963. “The Oriental Impingement on European Music.” *Islamic Studies* 2 (3): 337–42.

Ferreira, Manuel Pedro Ramalho. 1997. “Music at Cluny: The Tradition of Gregorian Chant for the Proper of the Mass—Melodic Variants and Microtonal Nuances.” Princeton University.

Florensky, Pavel. 1996. *Iconostasis*. New York: St. Vladimir’s Seminary Press.

Gadjibekov, Uzeir. 1957. *The Foundations of Azerbaijanian Folk Music [Основы азербайджанской народной музыки]*. 2nd ed. Baku: Azmuzgiz [Азмузгиз].

Garofalo, Girolamo. 1995. “Traditional Rural Songs in Sicily.” *Música Oral Del Sur: Revista Internacional*, no. 1: 65–89. http://www.centrodedocumentacionmusicaldeandalucia.es/opencms/documentacion/revistas/articulos-mos/traditional-rural-songs-in-sicily.html.

———. 2004. “Music and Identity of Albanians in Sicily: Liturgical Byzantine Chant and Devotional Musical Tradition.” In *Manifold Identities: Studies on Music and Minorities*, edited by Ursula Hemetek, 271–88. Cambridge, UK: Cambridge Scholars Press. https://books.google.com/books?id=awgJ2LtnU6oC.

Gertsman, Yevgenii. 1988. *Byzantine Musicology [Византийское музыкознание]*. Leningrad: Muzyra.

Gurney, O. R., and Martin L. West. 1998. “Mesopotamian Tonal Systems: A Reply.” *Iraq* 60 (1998): 223–27.

Hagel, Stefan. 2005. “Is Nîd Qabli Dorian? Tuning and Modality in Greek and Hurrian Music.” *Baghdader Mitteilungen* 36: 287–348. http://cat.inist.fr/?aModele=afficheN&cpsidt=17961761.

———. 2009. *Ancient Greek Music: A New Technical History*. New York: Cambridge University Press.

Heckenlively, Lura. 1900. *The Fundamentals of Gregorian Chant*. Tournai, Belgium: Society of St. John Evangelist, Desclée & Co.

Helmholtz, Hermann von. 1885. *On the Sensations of Tone as a Physiological Basis for the Theory of Music*. Translated by Alexander John Ellis. London: Longmans, Green and Co.

Herlinger, Jan W. 2002. “Medieval Canonics.” In *The Cambridge History of Western Music Theory*, 168–92. Cambridge, UK: Cambridge University Press.

Hermas, Tatian, Theophilus, Athenago, and Clement. 2007. *The Ante-Nicene Fathers: The Writings of the Fathers Down to A. D. 325*. Edited by Alexander Roberts. Vol. 2. New York: Cosimo, Inc.

Hiley, David. 1990. “Plainchant Transfigured.” In *Antiquity and the Middle Ages*, edited by James W. McKinnon, 120–42. London: Palgrave Macmillan.

Hindemith, Paul. 1942. *The Craft of Musical Composition, Book I: Theoretical Part, 4th Edition*. Translated by A. Mendel. New York: Assoc. Music Publishers.

Homan, Frederic W. 1964. “Final and Internal Cadential Patterns in Gregorian Chant.” *Journal of the American Musicological Society* 17 (1): 66–77. doi:10.2307/830030.

Hornbostel, Erich M. von. 1975. *Opera Omnia*. Edited by Klaus P. Wachsmann, Dieter Christensen, and Hans-Peter Reinecke. Vol. 1. The Hague, Netherlands: Martinus Nijhoff.

Huron, David. 2006. *Sweet Anticipation: Music and the Psychology of Expectation*. Cambridge, MA: MIT Press. http://www.amazon.com/Sweet-Anticipation-Psychology-Expectation-Bradford/dp/B00C6P2AWS/ref=sr_1_2?s=books&ie=UTF8&qid=1418199773&sr=1-2&keywords=Sweet+anticipation%3A+Music+and+the+psychology+of+expectation.

Katsanevaki, Athena N. 2011. “Chromaticism: A Theoretical Construction or a Practical Transformation?” *Muzikologija: Casopis Muzikoloskog Instituta Srpske Akademije Nauka I Umetnosti* 11: 159–80. doi:10.2298/MUZ1111159K.

Kholopov, Yurii. 1988. *Harmony: A theoretic course [Гармония: теоретический курс]*. Moscow: Muzyka [Музыка].

———. 2006. *Musical-Theoretic Systems [Музыкально-теоретические системы]*. Moscow: Kompozitor.

Kilmer, Anne Draffkorn, and Steve Tinney. 1996. “Old Babylonian Music Instruction Texts.” *Journal of Cuneiform Studies* 48: 49–56.

Kluckert, Ehrenfried. 2004. “Gothic Architecture in Italy.” In *The Art of Gothic : Architecture, Sculpture, Painting*, edited by Rolf Toman and Achim Bednorz, 242–51. Koln, Germany: Konemann.

Koço, Eno. 2015. *A Journey of the Vocal Iso(n)*. Cambridge, UK: Cambridge Scholars Publishing.

Kozbelt, A. 2008. “Performance Time Productivity and Versatility Estimates for 102 Classical Composers.” *Psychology of Music* 37 (1): 25–46. doi:10.1177/0305735608090846.

Krumhansl, Carol L. 1979. “The Psychological Representation of Musical Pitch in a Tonal Context.” *Cognitive Psychology* 11 (3): 346–74. doi:10.1016/0010-0285(79)90016-1.

———. 1990. *Cognitive Foundations of Musical Pitch*. New York: Oxford University Press. doi:10.1121/1.404005.

Kutuzov, Boris P. 2008. *Russian Znamennyi Chant [Русское знаменное пение]*. Moscow: Andrei Rublev [Андрей Рублев].

Kvitka, Kliment V. 1971. *Selected Works [Избранные труды]*. Edited by Goshovskii V. L. Vol. 1. Moscow: Sovetskii Kompozitor [Сов. композитор].

Leaman, Oliver. 2004. *Islamic Aesthetics: An Introduction*. Edinburgh: Edinburgh University Press.

Lester, Joel. 1977. “Major-Minor Concepts and Modal Theory in Germany: 1592-1680.” *Journal of the American Musicological Society* 30 (2): 208–53.

Lind, Tore Tvarnø. 2012. *The Past Is Always Present: The Revival of the Byzantine Musical Tradition at Mount Athos*. Lanham, MD: Scarecrow Press. https://books.google.com/books?id=VPWjSeLibr4C.

Long, Pamela O. 2003. *Openness, Secrecy, Authorship: Technical Arts and the Culture of Knowledge from Antiquity to the Renaissance*. Baltimore: JHU Press.

Lorca, Federico G. 2002. “From Play and Theory of the Duende.” In *Twentieth Century Theatre: A Sourcebook*, edited by Richard Drain, 263–65. London: Routledge.

———. 2010. *In Search of Duende*. New York: New Directions.

Lundberg, Dan. 1997. “Welcome to Assyria: Your Land on the Cyber space—Music and the Internet in the Establishment of a Transnational Assyrian Identity.” *Etnomusikologian Vuosikirja* 10: 13–28.

Maniates, Maria R. 1993. “Nicola Vicentino’s Reconstruction of the Ancient Greek Genera.” *Revista de Musicología* 16 (3): 16–36.

Manuel, Peter. 1986. “Evolution and Structure in Flamenco Harmony.” *Current Musicology* 42: 46–57.

———. 1989a. “Modal Harmony in Andalusian, Eastern European, and Turkish Syncretic Musics.” *Yearbook for Traditional Music* 21: 70–94. doi:10.2307/767769.

———. 1989b. “Andalusian, Gypsy, and Class Identity in the Contemporary Flamenco Complex.” *Ethnomusicology* 33 (1): 47. doi:10.2307/852169.

Maraqten, Muhammad. 1993. “Wine Drinking and Wine Prohibition in Arabia before Islam.” In *Proceedings of the 26th Seminar for Arabian Studies, Manchester on 21st - 23rd July 1992*, 95–115. Oxford UK: Archaeopress.

Marcus, Scott. 1993. “The Interface between Theory and Practice: Intonation in Arab Music.” *Asian Music* 24 (2): 39–58.

Martynov, Vladimir I. 1994. *History of Liturgical Singing [История богослужебного пения]*. Moscow: Rio Fa.

Mathiesen, Thomas J. 1999. *Apollo’s Lyre: Greek Music and Music Theory in Antiquity and the Middle Ages*. Lincoln, NE: University of Nebraska Press.

Mattheson, Johann, and Ernest Charles Harriss. 1981. *Johann Mattheson’s Der Vollkommene Capellmeister: A Revised Translation with Critical Commentary*. Translated by Ernest Charles Harriss. Ann Arbor, Mich.: UMI Research Press.

Meshscherina, Yelena G. 2000. *Musical Culture of Russia during the Middle Ages [Музыкальная культура Средневековой Руси]*. Moscow: Znaniye [Знание].

Meyer, Christian. 1996. *Mensura Monochordi: La Division Du Monochorde (IXe-XVe Siècles)*. Paris: Klincksieck.

Moisil, Costin. 2011. “Romanian vs. Greek-Turkish-Persian-Arab: Imagining National Traits for Romanian Church Chant.” *Muzikologija* 11: 118–32. doi:10.2298/MUZ1111119M.

Moll, Kevin N. 2014. “Toward a Comprehensive View of Compositional Priorities in the Music of Dufay and His Contemporaries.” In *Counterpoint and Compositional Process in the Time of Dufay: Perspectives from German Musicology*, edited by Kevin N. Moll, 3–63. Abingdon, Oxfordshire: Routledge.

Nettl, Bruno. 1972. “Persian Popular Music in 1969.” *Ethnomusicology* 16 (2): 218. doi:10.2307/849722.

———. 1974. “Thoughts on Improvisation: A Comparative Approach.” *The Musical Quarterly* 60 (1): 1–19. http://www.jstor.org/stable/741663.

Neubaer, E. 1992. “Music in the Islamic Environment.” In *History of Civilizations of Central Asia*, edited by Clifford Edmund Bosworth and Asimov, 4:2:712. Motilal Banarsidass Publ.

Nevo, Yehuda D. 2003. *Crossroads to Islam: The Origins of the Arab Religion and the Arab State*. Amherst, NY: Prometheus Books.

Nikolsky, Aleksey. 2015. “Evolution of Tonal Organization in Music Mirrors Symbolic Representation of Perceptual Reality. Part-1: Prehistoric.” *Frontiers in Psychology* 6 (1405). doi:http://dx.doi.org/10.3389/fpsyg.2015.01405.

Pacholczyk, Jozéf. 1993. “Early Arab Suite in Spain: An Investigation of the Past Through the Contemporary Living Traditions.” *Revista de Musicología* 16 (1): 358–66. http://www.jstor.org/stable/20795894.

———. 1996. “Music and Astronomy in the Muslim World.” *Leonardo* 29 (2): 145–50.

Pennanen, Risto. 2008. “Lost in Scales: Balkan Folk Music Research and the Ottoman Legacy.” *Muzikologija*, no. 8: 127–47. doi:10.2298/MUZ0808127P.

Petrović, Ankica. 1994. “The Eastern Roots of Ancient Yugoslav Music.” In *Music \ Cultures in Contact: Convergences and Collisions*, edited by Margaret J. Kartomi and Stephen Blum, 13–20. Basel, Switzerland: Gordon and Breach Publishers.

Picken, Laurence. 1953. “Instrumental Polyphonic Folk Music in Asia Minor.” *Proceedings of the Royal Musical Association* 80 (1): 73–86. doi:10.1093/jrma/80.1.73.

Pohlmann, Egert, and Martin L. West. 2001. *Documents of Ancient Greek Music: The Extant Melodies and Fragments*. Oxford: Oxford University Press.

Racy, A. J. 2004. *Making Music in the Arab World: The Culture and Artistry of Tarab*. Cambridge, UK: Cambridge University Press.

Reynolds, Christopher. 1987. “Musical Evidence of Compositional Planning in the Renaissance: Josquin’s ‘Plus Nulz Regretz.’” *Journal of the American Musicological Society* 40 (1): 53–81. doi:10.1525/jams.1987.40.1.03a00030.

Roederer, Juan G. 2008. *The Physics and Psychophysics of Music: An Introduction*. Berlin, Heidelberg: Springer Science & Business Media.

Rosenthal, Franz. 1966. “Two Graeco-Arabic Works on Music.” *Proceedings of the American Philosophical Society* 26 (4): 268–73. doi:10.1016/S0016-0032(38)92229-X.

Rouget, Gilbert. 1985. *Music and Trance: A Theory of the Relations Between Music and Possession*. University of Chicago Press. https://books.google.com/books?id=NzT90FcmrI4C.

Sabaino, Daniele, and Marco Mangani. 2013. “Counterpoint and Modality in Gesualdo’s Late Madrigals.” *Philomusica on-Line*. http://riviste.paviauniversitypress.it/index.php/phi/article/viewFile/1617/pdf.

Sanlucar, Manolo, Corey Whitehead, and Javier Alcantara-Rojas. 2011. “The Speculative Theories of Manolo Sanlucar: The Greek Origins of Flamenco Music.” *International Journal of the Humanities* 9 (7): 7–19.

Scruton, Roger. 1997. *The Aesthetics of Music*. New York: Clarendon Press.

Scurtu, Bogdan, and C. Tutu. 2011. “Romanian Ochtoechoi and Similarities to Middle Eastern Modes and Practices: A Case Study (part I).” *Scientific Bulletin of the Transilvania University of Brasov* 4 (53/2): 89–98.

Shannon, Jonathan H. 2003. “Sultans of Spin: Syrian Sacred Music on the World Stage.” *American Anthropologist* 105 (2): 266–77. doi:10.1525/aa.2003.105.2.266.

———. 2007. “Performing Al-Andalus, Remembering Al-Andalus: Mediterranean Soundings from Mashriq to Maghrib.” *Journal of American Folklore* 120 (477): 308–34. doi:10.1353/jaf.2007.0060.

———. 2013. “Emotion, Performance, and Temporality in Arab Music: Reflections on Tarab.” *Cultural Anthropology* 18 (1): 72–98. http://www.culanth.org/articles/472-emotion-performance-and-temporality-in-arab.

Shestakov, Biacheslav. 1966. *From Ethos to Affect: History of Musical Aesthetics from Antiquity to the 18th Century [Музыкальная эстетика западноевропейского средневековья и Возрождения]*. Moscow: Muzyka [Музыка].

Shulze, Bernhard F., and Ehrenhard Skiera. 1990. “Guitarra Flamenca.” In *Flamenco: Gypsy Dance and Music from Andalusia*, edited by Claus Schreiner, translated by Mollie Peters, 121–46. Pompton Place, NJ: Amadeus Press.

Shumays, Sami Abu. 2009. “The Fuzzy Boundaries of Intonation in Maqam: Cognitive and Linguistic Approaches.” In *Conference of the Society for Ethnomusicology; 2009 Annual Meeting (54th): Mexico City, Mexico, November 19-22, 2009*, edited by Brenda M. Romero. Indianapolis: Indiana University Press. http://maqamlessons.com/analysis/media/FuzzyBoundaries_MaqamIntonation2009.pdf.

Signell, Karl L. 1977. *Makam: Modal Practice in Turkish Art Music*. Da Capo Press, Incorporated.

Simms, Rob. 2003. *The Repertoire of Iraqi Maqam*. Scarecrow Press.

Simonton, Dean Keith. 1980. “Thematic Fame and Melodic Originality in Classical Music: A Multivariate Computer-Content analysis1.” *Journal of Personality* 48 (2): 206–19. doi:10.1111/j.1467-6494.1980.tb00828.x.

Smith, Nicholas, and Mark Schmuckler. 2004. “The Perception of Tonal Structure through the Differentiation and Organization of Pitches.” *Journal of Experimental Psychology. Human Perception and Performance* 30 (2): 268–86. doi:10.1037/0096-1523.30.2.268.

Sposobin, Igor V. 1969. *Lectures on the Course of Harmony [Лекции по курсу гармонии]*. Edited by Yurii Kholopov. Moscow: Muzyka.

Stathis, Gregorius. 2014. *Introduction to Kalophony, the Byzantine Ars Nova*. Translated by Konstantinos Terzopoulos. Oxford: Peter Lang.

Subirats, Maria-Angels. 2006. “The Special Role of Music in Judeo-Sephardic Culture.” In *The Past in the Present: A Multidisciplinary Approach*, edited by Fabio Mugnaini, Pádraig Héalaí, and Tok Freeland Thompson, 107–18. editpress.

Sultan, Nancy. 1988. “New Light on the Function of ‘Borrowed Notes’ in Ancient Greek Music: A Look at Islamic Parallels.” *Journal of Musicology* 6 (3): 387–98. doi:10.1525/jm.1988.6.3.03a00050.

Talbot, Michael. 2000. “The Work-Concept and Composer-Centredness.” In *The Musical Work: Reality or Invention*, edited by Michael Talbot, 168–86. Liverpool, UK: Liverpool University Press.

Thompson, Barbara. 1985. “Flamenco: A Tradition in Evolution.” *The World of Music* 27 (3): 67–80.

Tischler, Hans. 1973. “‘Musica Ficta’ in the Thirteenth Century.” *Music & Letters* 54 (1): 38–56.

———. 1999. “On Modality in Trouvère Melodies.” *Acta Musicologica* 71 (1): 76–81.

Touma, Habib Hassan. 1996. *The Music of the Arabs*. Portland, Oregon: Amadeus Press.

Trachtenberg, Marvin. 2001. “Architecture and Music Reunited: A New Reading of Dufay’s ‘Nuper Rosarum Flores’ and the Cathedral of Florence.” *Renaissance Quarterly* 54 (3): 740. doi:10.2307/1261923.

Tsuzaki, Minoru. 1991. “Effects of the Preceding Scale on Melodic Interval Judgement in Terms of Equality and Size.” *Music Perception* 9 (1): 47–70.

Unruh, Patricia. 1983. “’Fumeur’ Poetry and Music of the Chantilly Codex : A Study of Its Meaning and Background.” Vancouver, Canada: University of British Columbia. doi:10.14288/1.0095778.

Vuvan, Dominique T., and Mark A. Schmuckler. 2011. “Tonal Hierarchy Representations in Auditory Imagery.” *Memory & Cognition* 39 (3): 477–90. doi:10.3758/s13421-010-0032-5.

Walters, Gareth. 2007. “Music.” In *A Companion to Federico García Lorca*, edited by Federico Bonaddio, 63–83. Woodbridge, Suffolk, UK: Tamesis. https://books.google.com/books?id=EQM3y6WmQoYC.

Ware, Kallistos. 2000. *The Inner Kingdom*. Crestwood, New York: St Vladimir’s Seminary Press.

West, Martin L. 1981. “The Singing of Homer and the Modes of Early Greek Music.” *The Journal of Hellenic Studies* 101: 113–29. doi:10.2307/629848.

———. 1992. *Ancient Greek Music*. New York, London: Oxford University Press.

Westrup, Jack Allan. 1954. “Medieval Song.” In *New Oxford History of Music: Early Medieval Music up to 1300*, 2:220–69. Oxford, UK: Oxford University Press.

Yöre, Seyit. 2012. “Maqam in Music as a Concept, Scale and Phenomenon.” *Journal of World of Turks* 4 (3): 267–86. EBSCO accessory # 84609213.

Zannos, I. 1990. “Intonation in Theory and Practice of Greek and Turkish Music.” *Yearbook for Traditional Music* 22 (1990): 42–59.

Zemtsovsky, Izaly. 2012. “M. Gnesin on the Modal System of Jewish Music [М. Ф. Гнесин о системе ладов еврейской музыки].” *Scholarly Bulletin of the Moscow Conservatory* 4: 6–25.

1. Following the definition by Kholopov (1988, 38) who proposed the term “hemiolic” (Greek "*hemiolia*" – the 1½:1 ratio) to refer to such modes that contain the interval of augmented 2^nd^ between two adjacent degrees – as a permanent feature of modal organization rather than temporary alteration of a degree (presence of characteristic modal intonations that explicitly feature the interval of the augmented 2^nd^ ). [↑](#footnote-ref-1)
2. Uzeir Hajibeyov, one of the founders of the music theory of mugam (Azerbaijanian music system derivative of maqam), wrote: “There is a view that if harmony is applied to naturally monophonic Azerbaijan music, all its modal peculiarities will be reduced to zero. It is quite true. Clumsy application of harmony to Azerbaijan melodies may change their character, neutralize the distinction of their modal peculiarities and even make them rough and vulgar” (Gadjibekov 1957, 32). In this light, the dominant keys might be regarded as the product of applying vertical harmony of the Western music to the modes of Maghrebian, Near Eastern and Central Asian traditional music. [↑](#footnote-ref-2)
3. If major VI receives a dedicated harmonization, then it is usually an inversion of the IV triad rather than the triad itself – as evident in the Hajibeyov’s score (http://uzeyir.musigi-dunya.az/ru/sensiz.html). [↑](#footnote-ref-3)
4. The famous association of Gypsy music with the interval of augmented 2^nd^, often qualified as “harmonic minor” or “double-harmonic minor” (if it also uses the high IV degree in minor key), should be reconsidered as the heritage of a much more ancient Indo-Arian culture – very similar modal structures have been recorded in Calcutta in 1902 (Hornbostel 1975, 1:158–168). [↑](#footnote-ref-4)
5. There is evidence that micro-intervals were still used in early Middle Ages in Europe (Meyer 1996) – but vanished with the advent of the staff notation, as Ferreira points out, probably due to the “quantization” of tuning, prompted by graphic representation on the staff lines (Ferreira 1997, 160–289). [↑](#footnote-ref-5)
6. This effect must have been explored yet by the Ancient Greeks. It has to do with the perceptual phenomenon that tones that are more frequently repeated (Aarden 2003) and assigned longer time values (Smith & Schmuckler 2004), in music not conforming to Western tonality (Cuddy 1997), are perceived as more stable (Bharucha 2002) proportional to their frequency of repetition (Krumhansl 1990, 67). Such tones also are remembered better than the unstable tones (Krumhansl 1979). According to Stefan Hagel (in personal communication), the amount of chromatic/enharmonic tones probably exceeded the amount of diatonic tones in Ancient Greek dramatic arias around the 4^th^ century BC, and especially in spondeion piping. Such music must have manifested to the contemporary audiences a noticeably different gravitational style, making the musical modes significantly less memorable, and reducing gravity in the modal anchors. Once forged, such music could have won appreciation in the neighboring folk cultures of the Near East, and survived there, impressing the visiting Carolingian authorities with their exotic sound. [↑](#footnote-ref-6)
7. Using tritone as a criterion for definition of a mode immediately brings to mind the theory of modal voicing by Yavorsky (Ewell 2012). Being criticized for lack of historic support for his theory of tritone as a driving force that directs resolution within a mode, Yavorsky's ideas might now receive confirmation from Ancient history. Babylonian texts (CBS 10996 and UET VII 74) are explicit in prescribing to leave out the "unclear interval" (triton) at the end, before reaching the octave (Gurney & West 1998) – which makes its exclusive status very clear. [↑](#footnote-ref-7)
8. This matter deserves a thorough quantitative study: modern computer technology allows to run statistic analysis of huge databases of folk melodies to estimate if the prevalent melodic direction for a given ethnic style is ascending or descending. My overall musical impression is that the Ancient Greek style can be characterized as *ataraxic*, while Arabic, Turkish and Persian styles all share *climactic* character, promoting large-scale “build-up” of melodic tension. Of course, this directionality remains only a tendency: in order to get up, the melodic line has to come down – it is impossible to keep moving in the same direction for the entirety of a music work. The climactic melodic type keeps climbing up as an *average* of all the melodic phrases, and can be terminated by few descending phrases that would therefore perceptually stand out. Such typology is often encountered in the genres of vocal music like Mawwāl and Qawwāli. [↑](#footnote-ref-8)
9. Stefan Hagel argues that West’s conclusion is not conclusive and provides references to the passages from Kleonides, Aristides, Alypios and Gaudentios, which use ascending enumeration for pitches (Hagel 2005, 299). He also points out that the alphabet does not start on the modally important note, but on C# (required only in enharmonic Dorian genus) and ends on a modally insignificant note as well. Although the descending alphabet in itself does not constitute the descending scale, nevertheless generality of the alphabet exceeds separate examples by music theorists, and testifies to the commonality and convenience of mental representation of pitch in the descending order for the purpose of studying music, which was the primary implementation for Greek notation. It could be that while some theorists could conceive ascending scales, lay music users considered descending direction more comfortable. [↑](#footnote-ref-9)
10. The maqam Kurd forms another very common genre in the Arabic music system – which is characterized by the *descending* melodic motion (Touma 1996, 36) – just like the Ancient Greek Dorian mode. It is interesting that modulation from the Kurd (E-F-G-A) to the Hijaz tetrachord (E-F-G#-A) reverses the way how the Ancient Greek Dorian tetrachord (E-F-G-A) received its chromatic shading by lowering its III degree (E-F-Gb-A). This modulation from the Dorian mode that was central for the Greek ethos could have occurred in the Sabaean culture, and passed on to the North-West Hijaz through the Sabaean colonies (Nevo 2003, 68), earning a name for the “sharpened” modification of a mode. [↑](#footnote-ref-10)
11. Although the Ajax tetrachord seems to feature the leading tone, the exact intervals in it are disputable: i.e. raising in pitch could involve just a quarter-tone. [↑](#footnote-ref-11)
12. However there remains a possibility that the entire tetrachord was engaged melodically in the part of the accompanying cithara, provided that the accompaniment to songs could have featured passages and extra tones added to the dubbing of the vocal part (Mathiesen 1999, 362). [↑](#footnote-ref-12)
13. Technically speaking, the notion of a tetrachord in Aristoxenian theory implied a melodic interval of the 4^th^ of a particular intervallic shape that was reproduced a 5^th^ or a 4^th^ above (or below) the original tetrachord to make a scale – which differs from the notion of tetrachord in the music theory of maqam (this distinction is made according to the explanation by Stefan Hagel, in personal communication). [↑](#footnote-ref-13)
14. In this light, the affinity of Mediterranean tonality with hypermodal organization, evident in maqam/dastgah as well as in oktoechos/hexaechos, obtains some deep meaning. Hypermodal opposition to chromatic alteration can be seen as an attractive feature in constructing the melodic “scapes” on the foundation of monodic textures, securing autonomy for each of the melodic modules within a corresponding “hypermodal” subset, while achieving the integration effect, required by the ethos of a specific mode. The priority of the task to illuminate this ethos at all times - without alterations and cancellations inherent to the “accidental” nature of chromaticism – could have promoted emergence of hemiolic chromaticism out of the Ancient Greek chromatic system, regulated by the hypermodal principles. [↑](#footnote-ref-14)
