# Supplementary material for: Evolution of Tonal Organization in Music Optimizes Neural Mechanisms in Symbolic Encoding of Perceptual Reality. Part-2: Ancient to Seventeenth Century
Source: Front Psychol. 2016 Mar 30;7:211. doi: 10.3389/fpsyg.2016.00211 (PMC4813086; doi:10.3389/fpsyg.2016.00211)
Supplement: Supplementary file 3 [file DataSheet1.zip › Appendices I-VIII/Appendix V. Historic Overview of Correspondence Between Tonal and Spatial Organizations.docx]

# Appendix V: Overview of Correspondence Between Tonal and Spatial Organizations in Their Historic Development

It is extremely important to identify the similarities between the tonal organization in music and spatial organization in pictorial arts throughout their historic development. Fine arts present the form for the most obvious reflection of perceptual reality. The history of pictorial art demonstrates, in the clearest possible way, the evolution of spatial representation. “Spatial” aspect of organization of pitch in “vertical” and “horizontal” parameters of virtual music space is considerably less obvious, and the course of its evolution is substantially harder to grasp. By identifying the common cultural traits in tonal organization of music and spatial organization of pictorial art, we obtain a much better view on those aspects of perceptual reality that have been abstracted and represented in tonal schemata of music.

We have already discussed the issue of much greater popularity and availability of music-making and music consumption as compared to the popularity of depicting real objects and consuming pictorial art. Understanding of how characteristic features of one’s living environment can be reflected in tonal organization of music can provide a glimpse into how the use of music according to such tonal principles could transmit and reinforce a particular thinking culture – a culture capable of promoting a particular worldview, thereby suggesting a particular criterion of selecting important features of the visible reality.

In effect, making and listening to a certain type of music might make one see the world in a certain way and, as such, depict it in drawings and paintings, accordingly. Cultural ubiquity of music, biological roots of musicality, its unprecedented capacity to entrain great number of individuals in sharing the same emotional experience – can all contribute to the “making” of one’s mind, attuning it to a mental model that is optimal for a given geographic environment and social engagement. Synesthetic equivalence of melodic and visual contours could provide the ground for “seeing” in terms of “hearing” – administered through *a set of instructions that stay embedded in structures of tonal organization* of one’s favorite music.

The first issue that needs to be addressed is the historic connection between the first forms of strict implementation of linear perspective and chromatic tonality: if this premise is correct, then formulation and cultivation of perspectival organization in Renaissance paintings should concur with the cultivation of vertical and horizontal traits of tonal organization that characterize tonality. Pronounced similarity of perspectival, horizontal harmonic, and vertical harmonic tonal organizations appears to characterize only the Western art *after* the Renaissance (see Part-2, Table-1). In Renaissance music, music theory lagged behind perspectival theory in implementation of centrality and vectorization principles in relation to *horizontal* harmonic organization. Only a single ratio principle was in place by the mid 16^th^ century: in 1567 Giacomo Gorzanis wrote a collection of 24 dance suites for lute, using each of the 12 steps of the chromatic scale (Pirina 1985), and the theoretic foundation for tuning to enable performance of such music was provided in 1584 by Vincenzo Galilei, the father of Galileo Galilei (Barbour 2004, 8).

But let’s inspect if the principles of tonicity and unity of tonal organization for a particular composition were really absent in the 16^th^ century Western music. Although the term “tonic” was introduced much later, in 1710, by Saint Lambert (Lester 1989, 100), there existed something similar in sound to tonicity during the 16^th^ century. Especially in short musical compositions, a relatively strong sense of tonicity could be evoked by means of repeating the Dorian cadence (Lowinsky 1962, 4). Short miniatures made a substantial portion of the lute repertoire, in particular the dance genres (i.e. *passamezzo*). Popularization of this kind of music paved the way for Johannes Lippius in 1612 to declare that it was the tonic triad rather than the species that formed the basis for the modes (Berger 2006).

Of course, we are looking back in history, having in mind concepts that the Renaissance musicians could not and did not have. Nevertheless, objectively speaking, marking of a single triad as the stability anchor for an entire piece was part of the experience of listening to the music of the late 16^th^ century. The succession of passamezzo antico in minor mode by passamezzo moderno in major mode on the same “tonic” tone was quite common in the dance guitar/lute practice of the mid-16^th^ century (Hudson 1970).

The only horizontal musical equivalent of the perspectival tri-unity of vantage, vanishing points, and scale that was clearly missing in the Renaissance music was the key recapitulation principle. The concept of “tonal plan,” necessary for observation of the return to the tonal scheme that was featured in the beginning of a composition, was laid out by Johann Albrechtsberger at the end of the 18^th^ century. He defined the system of relative keys and outlined their succession for principal music forms, specifying that the initial key should return at the end (Kholopov 1988, 237). However, the idea of musical recapitulation was not invented by Albrechtsberger. Its origin must have been in the classic rhetoric: *anakephalaiôsis* - recounting in brief what has been said in the narration – required as a part of composition in situations where refutations and confirmations were numerous and strong (Kennedy 2003, 28). The revival of interest in rhetoric composition for arrangement of preaches during the late Middle Ages involved the use of recapitulation (Murphy 1981, 313). The rhetoric composition of sermons had its counterpart in music that was used in the same liturgy with the purpose to support the words. From the 16^th^ century, *musica reservata* played a key role in maintaining close ties between the organization of words and music – which included compositional schemes amongst other things (McCreless 2002).

Albrechtsberger’s concept of “tonal plan” had its precursor in Baroque music – the notion of “cadential plan.” Here, the principal difference was that although Baroque tonal rules required to pass through a circle of keys throughout the composition, returning back to the original one, the underlying idea was to diversify the tonal content within a music work - and not to resume the key – quite in contrary: the diversity in cadences was called forth to *offset* the principal key (Kholopov 1988, 237). Although the classicistic concern for integrity of tonal organization throughout a composition did not exist for Baroque composers, they nevertheless did conceive the totality of tonal content for a composed work.

The Renaissance composers were coming close to grasping such concept, too. The idea of “cadential plan” was known during the Renaissance. In fact, it became one of the factors contributing to the identification of a particular mode by the time of Palestrina (Mangani & Sabaino 2008). Of course, cadential planning was motivated by purely melodic concerns: harmonic progressions were out of scope for the 16^th^ century musicians. Even the most prominent Renaissance theorists as though "wore blinders that prevented them from seeing more than two chords at a time" (Palisca 1956).

It is justified to state that “cadential plan” was a reality of modal organization in Renaissance music, albeit understood only in horizontal rather than vertical dimension of musical texture. It seems that the 16^th^ century was the turning point for genesis of the homogenous perception of musical texture, especially evident in evolution of lute music, where uniformity of timbre in a multi-part instrument allowed for cultivation of proto-homophonic textures of chordal type (Comberiati 1983). The closest to the sound of a “retained key” in the Renaissance music would be the formula-based frottolas for voice solo with the lute or lyre accompaniment, where the return of the opening harmony would come after the end of the formula, resembling recapitulation – and novel genre of aria, which most likely followed folk-song prototypes (Ashworth and O’Dette 2007) making use of refrains.

1. Audio: Tromboncino – Frottola “Ostinato vo’ seguire” (1509). The ongoing repetitions of the same harmonic formula determine most of the content of this composition – except its very conclusion - producing a clear impression of the key of G Major. This device is definitely deliberate on the part of the composer, illustrating the lyrics: “Ostinato vo’ seguire la magnanima mia impresa” (I shall stubbornly continue my generous exploit). <http://bit.ly/1LiHgzJ>

Although such compositions were not originally conceived as “in major/minor key” by their composers, nevertheless, they should be taken as a proof of awareness by the composers of increased tonal integrity in a frottola as opposed to, for instance, a motet. In fact, this increased tonal homogeneity could very well have been the reason behind a spike of frottola popularity and deliberate use of “ostinato tonality” by the Renaissance composers specializing in frottola, such as Tromboncino and Cara. The pattern of publication and distribution of frottolas in the beginning of the 16^th^ century indicates that they were intended for a new quickly developing market of the amateur performer (177 frottolas printed in 1504 alone), and simplicity of their style was supposed to enable the purchaser to build his own repertory by comfortably adjusting the musical material to his own needs (Boorman 2005, 281–290). In this capacity, frottolas clearly opposed other published music genres, i.e. masses. Not surprisingly, the tonal organization of frottolas had to provide a more uniformed and coherent tonal scheme, convenient for comprehension – unlike the intricate polymodal organization of masses which required profound scholarship from their purchaser.

All in all, the Renaissance culture provided almost all the basic features required for projecting a “heliocentric” worldview onto the realms of spatial organization in pictorial arts and tonal organization in music. The 18^th^ century developments only capitalized on the tendencies already present in the Renaissance culture. But what about the pre-Renaissance culture? The Renaissance theorists made the Ancient Greco-Roman Sources to be “born again.” Would the correspondence between perspective and early forms of tonality find its counterpart in arts and music of antiquity?

First of all, we should stress a principal difference between Renaissance and Hellenic perspectives. Ancients' concept of space was "sensuous," and therefore aggregate and finite – Ancients were interested in visible objects rather than space that separated them (Panofsky 1991, 41). Aleksey Losev (Losev 2000, 3:177) defines the ancient perspective as "harmonious combination of depicted bodily objects, which does not take into account space that separates bodies, in conceptualizing the unity of the entire picture". This “naive” realism regards only bodies touching one another, ignoring air in between. Losev characterizes the 'depth' parameter in ancient pictures in the following way: "the presence of intervals in space can be perceived, but these intervals cannot be expressed by a single and general coefficient" (ibid.). Subsequently, such “ancient” perspective was not strictly linear. Perspective looked correct only when the depicted bodies were disposed of, one by one, going into 'depth', filling up the space to the horizon. But as soon as there was a sizeable gap between the bodies, the size of these bodies was not adjusted according to the distance, which was supposed to separate them.

Renaissance artists applied perspective to infinite space, carefully constructing distances between pictorial objects. The same applies to music: Hellenic musicians did not account for tonal distance across the span of time – not at all! None of the surviving sources describes anything remotely resembling our “tonal plan”: the concern for succession of specific keys or cadences in a particular composition. Ancient Greek musicians were certainly sensitive to changes in pitch set (PS) and interval set (IS), but did not care to track one modulation to another for the entirety of the composition. They did not appreciate arching of the opening of a musical piece with its ending by the integrity of the same anchor tone. Their compositional practice seems to operate by moving from mode to mode, zooming into no more than two modes at a time (West 1992, 195).

The Hellenic non-linear perspective corresponded to *non-linear tonal plan*. Ancients took into account only the immediately sensible bodies: in relation to music, these were the "departure" tetrachord and the "arrival" tetrachord. The ability to track pitch changes over a longer span of time appears to be a product of enculturation and optimization of hierarchic processing of frequency (both, in bottom-up and top-down directions) characteristic of perception of tonality (Kumar et al. 2011).

**Table 1**. **Outline of tonal and perspectival organizations in historic and prehistoric art periods**. The spatial/tonal correspondence is structurally the strongest in the 17-18th century, somewhat pronounced in the 15-17^th^ century Western culture, is more limited in the Hellenic/Hellenistic/Islamic Mediterranean art, and shows only general similarity traits for the earlier periods of art history/prehistory.

| **Period** | **Features of Spatial Organization** | **Features of Tonal Organization** |
| --- | --- | --- |
| Age of Enlightenment (17^th^-18^th^ century) | Artists of the Age of Enlightenment conceptualized perspective into “perspectival space”: the idea of a particular place, pre-conceived by the artist who then populated that space with objects that best complimented that space (Elkins 1996, 14). The overall composition was usually compact and balanced.  Yet another development in perspectival organization, starting from Baroque, was the engagement of shading effects and tonal gradations that became more important than proportions in defining the depth aspect of a work (Bouleau 2014, 112). | The 17^th^-18^th^ century composers began to typecast texture - i.e. reserved specific textures for specific genres: chorale, minuet, march, etc., (Kholopova 1979) and filled up these standard textures with idiosyncratic melodic and harmonic material. Such textures were usually relatively compact polyphonic or homophonic, in design.  Compositions explored chromatic shading, often opposing diatonic and chromatic harmony in the manner of light/dark, promoting frequent modulations and applied functions. Harmonic functionality started taking lead in determining music form. |
| Renaissance and Early Baroque (15^th^-17^th^ century) | The rule of a single viewpoint was exempted where great scope required greater space and multiple gazes. In such cases 2-3 vanishing points and/or horizon lines were employed. The rule of a single scale was observed unless the composition required emphasis on a particular aspect of a figure or a room, in which case application of two scales was permitted as an exception.  Late 16^th^-17^th^ century compositions were frequently monumental, including enormous variety of objects, often contrasting one another, with great elaboration in detail (i.e. Veronese and Titian). “Close view” genres (portrait and still life) became popular – offsetting monumental art. | The rules of thematic composition according to the theory of rhetoric, voice-leading, and non-chordal tones were codified in treatises and observed rather strictly. The rules of alteration, modulation, and cadential plan were more flexible, allowing exceptions where strong contrast or expression of abnormal tension were needed.  Renaissance texture was usually heavily polyphonic, up to 60 parts (Tallis and Striggio) – except virtuosic instrumental pieces and new transparent stile moderno that often expressed intimate feelings. Baroque polyphony reduced the average number of parts, but complicated and elaborated the melodic and harmonic structures. |
| Hellenic, Hellenistic, and Medieval Near Eastern Art (5^th^ century BC – 15^th^ century AD) | Pre-Renaissance perspective accounted only for proportionality of objects, ordering them in the depth parameter without specifying any vantage and vanishing points (Richter 1970). Artists intuitively observed “aerial” perspective, not single-point, but flexible “multiscan,” affording oblique viewing (Arnheim 1977) – generally corresponding to what Panofsky termed "curvilinear" perspective (Panofsky 1991, 22), although strictly speaking, from an optical standpoint, it was not "curved" (Kubovy 1988, 107).  Ancient perspective, formulated by Euclid, followed the principle of angular distance, based on the “common sense” understanding of distance between the observer and objects positioned at different angles – in contrast to “level distance,” based on straight optic appearance of distant objects, discovered during Renaissance (Veltman 1980). | Music-makers considered only the modal aspect (PS) and the tonal aspect (stability) in tonal organization of a currently sounding structural unit and its immediate precursor – but not in the scope of the entire work. Mode could sustain throughout a composition, or modulate for a few times. The texture must have been heterophonic, featuring intricate non-formulaic melody and possible variants in accompanying parts, instrumental or vocal, with a probable drone tone and inclusion of instrumental variations and/or interludes.  Heterophonic voices might be thought of as equivalents to discrepant angle views, where the viewpoint shifts vertically or laterally – such shifts introduce curvature-like distortions (Veltman 1980), producing melodic variants similar to optic variants. In both cases, the perceptual whole must be assembled from summing up the variants. |
| Egyptian, Sumerian, Babylonian and Assyrian Art (30^th^-7^th^ century BC) | Near East “paratactic” or “ideoplastic” organization (Groenewegen-Frankfort 1951, 7) followed lines of what can be termed as “abstract rationalism” (Hauser 1999a, 1:23), based on axonometric principles of orthogonal projections. “All objects were depicted in direct frontal view,” while shifting the point of observation from one detail to another in order to “keep the viewing angle strictly perpendicular” (thereby producing a sum of orthogonal shots along the observation path), and “reducing all horizontal surfaces to a basic straight line” (Rauschenbach 1980, 32). The depth parameter was indicated by the overlap of a “closer” object. The object’s size was independent of the depth parameter – marking the distant view by addition of indexical signs to the images depicted from the bird’s-eye view projection (Rauschenbach 2002, 188). | Music-makers did not observe the uniformity of intervallic increment in defining the scales: Babylonian music system employed three types of different-sized semitones, whereas Classical Greeks had only one (Crickmore 2009). Absence of reliable transcriptions does not allow to discuss the details in tonal organization of music compositions. However, the texture of Mesopotamian music was most likely strict monody, because of close supervision by priests and palace administrators (Ziegler 2011), with clear standards of performance rationally defined by notation-based professional training (Michalowski 2010), and great religious importance placed on “correctness” in tonal organization – determined by its adherence to mathematical models. All music was reduced to a single melodic line, perceived by summing up familiar melodic intonations. |
| Neolithic Art (ca. 8,000-3,200 BC) | Prehistoric Egyptian art contrasted the canonic depiction of the first dynasties: the perpendicular viewing angle was not observed, leaving the continuity of the figures’ contours the main means of spatial ordering (Davis 1992, 5). This mechanistic integration reincarnated naturalistic approach of replicating visual contours by pictorial contours after a long period of “narrowly geometric stylization” (Hauser 1999a, 1:5), brought by the transition from food-gathering and hunting to cattle-breeding and planting. Increased demand for accounting promoted “diagrammatic” abstractization of the earlier Paleolithic naturalistic depiction method. The recovery of realistic principles probably had to do with the emerging function of writing in pre-literal cultures – assigned to ideographic signs, causing separation of realistic drawing from abstract symbols reserved for household accounting or calendar tracking (Huyghe 1966, 38).  “Diagrammatic” artists considered only figures adjacent in horizontal and/or vertical dimensions in spatial organization of their pictorial works. Figures remote from each other were not coordinated. | Hypothetical prehistoric music must have followed the course of development from ekmelic to emmelic organization – through the process of abstraction of pitch parameter from the entire spectral content. Isolation of pitch modulation from timbral modulation resembles separation of the ideographic sign from the graphic image. Timbral music is much more naturalistic (“graphic”) in imitating environmental sounds. Discretization of pitch presents a strongly cultural (“ideographic”) approach to sound production, based on the conventional agreement of music users. Establishment of rules for such production requires extensive cultivation of monophony, with focus on consecutive connections between different pitches – akin to figurative continuity in spatial organization.  The polyphonic implementations of emmelic music were also likely to be inherently “consecutive”: each singer had to hear his partner in order to come up with his own part (Arom 2004, 220). Such performance is tonally “short-sighted”: i.e. Buganda players only consider 2-3 neighboring steps in joined music-making (Kubik 2010, 116). |
| Paleolithic Art (ca. 40,000-10,000 BC) | Amazing realism of drawing animal outlines (including capturing animals in motion) characterizes murals of the entire Paleolithic period across a wide geographic area – until the late Magdalenian period, when stylized figurative style appeared side by side with a realistic style (Huyghe 1966, 34). However, even in stylizations, realistic connotations usually stayed strong, implemented to exaggerate a known important trait of the depicted object (Eastham 2005). Spatial disconnectivity in representation of real life objects appears to be a general marker of Paleolithic art (Okladnikov 1967, 117): no two figures comprise an ensemble – although the depicted objects might altogether represent a single scene. Depictions were often made over older depictions, as though each of them was immanent and undisturbed by overlapping (Uspensky 1995, 178). | The naturalism in representation of visual reality corresponds to what Sheikin (2002, 30) called “echolaliac instincts” that produced at first the psychophysiological, and then the organophonic intonations of (respectively) pre-modal and khasmatonal methods of tonal organization. Surviving ethnic traditions that implement these methods usually employ what can be termed “*jumbled texture*”: collective music-making without any coordination of parts – mere intuitive following of the same model by every participant with accidental discrepancies in pitch, rhythm, and timbre. No part is consciously tuned to any other part, yet all parts adhere to the same model, defined by the same goal of musical performance (and mutual lyrics). No clashing in pitch or rhythm is perceived as disrupting this model. |

If to try to draw parallels between the beginnings of pictorial art and pre-mode in prehistoric music (see Part-1), the mutual feature will definitely be the notion of contour. The ability to carve or depict a geometrically shaped line most likely corresponded to the ability to track a melodic contour. The earliest known depictive images are the Quneitra artifact from the Levant, 55,000 BP, and the Bacho Kiro bone from Bulgaria, ca. 44,000 BP (Marshack 1996).

1. A bone fragment with zigzag incision, Bacho Kiro, Bulgaria, 44,000 BP. The two rows of zigzag lines indicate the carver’s awareness of changing directions, of points marked in this way, and similarity of certain lines. <http://bit.ly/1SGlonM>

The closest musical analog to this would be vocalization of a few people following the same pitch contour with observable cross-relation between the rhythmic, timbral, and directional attributes of the contour envelop, so that the mutual resemblance could be recognized by ear.

1. Audio: Drinking chant, Campa Indians, Upper Amazon. Female choir keeps vocalizing the same “call” made by the intonation of the descending 3^rd^ – in celebration of manioc beer making (Tschopik 1954). <http://bit.ly/1NBBf2v>

There is no term in music theory to refer to this method of generating a clearly multipart texture. The most appropriate term would then be isophony – production of parts approximately equal in melodic, rhythmic, and timbral characteristics, where no particular part is salient, and the texture appears interwoven by juxtaposition of the same brief melodic contour model. The best demonstration of isophonic principle is found in such ensembles where each of the participants produces only one tone fixed in pitch throughout the session of music making.^[[1]](#footnote-1)^

1. Audio: Horn ensemble, Banda Linda, Central African Republic. The ensemble of sixteen *ongo* horns, each tuned to a particular pitch, perform the music, arranged from highest to lowest sound, in succession. <http://bit.ly/1R3qhJI>

Such music cannot be strictly qualified as “polyphonic,” since polyphony usually implies continuity and certain level of accomplishment of a part: a part is supposed to make sense on its own. Isophony, on the other hand, is characterized by fragmentary sound of music distributed to each of the participants, where taken separately, a part does not make any sense without the other parts (Jordania 2006, 103).

The isophonic texture can be elaborated by applying variations in pitch, rhythm, timbre, and length of each of the renditions of a model, assigning a particular variant to a particular member of the tribe. Then such a variant can serve as a “personal song” for this individual to be identified in the entire “choir.”

1. Audio: Akia song, Suyá Amazon Indians. A collective shout song that represents individual members of the tribe, based on their age and importance in the tribe. The lower the status, the shorter the musical phrase. The syllables “te-te-te” are used to integrate the singing between all the participants (Seeger 2004, 40). A single melodic formula integrates all the parts. <http://chirb.it/ryANbJ>

Isophonic texture can be characterized as jumbled texture, because it involves an ongoing rotation of the same set of closely related melodic elements, where they unpredictably realign with one another in time. The landmark of such tonal organization is integration of a strictly defined array of disjointed elements by means of sharing the same or very similar musical material. The visual equivalent to this would be the juxtaposition of similar images, where each image retains its autonomy by comprising a “frame” by its contours: the contour line separates such image from the “outside.”

1. Spotted horses, Grotte de Peche Merle, France, ca. 25,000 BP. The contour of one horse is painted over that of another, leaving it “see-through.” Both images remain disjointed, each existing in its own right. This is not the only “see-through” example from this cave. <http://bit.ly/1NP8Qwi>
2. Right wall of the Sanctuary at the Trois-Frères Cave, ca. 13,000 BC. Juxtaposition of many images, as found in quite a number of caves, creates an impression very much similar to “jumbled texture” in music. <http://bit.ly/1XO6mTB>

Primordial Paleolithic isophony most likely presented a repertoire of tonally disjointed melodic contours with gliding indefinite pitch – performed without any coordination by vertical intervals.

If the earliest proto-music sounded similar to wolves howling, with multiple random deviations from a “mainstream” pitch contour, the task of tuning-in must have involved individual calibration: breaking the collective vocalization into smaller components of solo and subsequently duo vocalizations. The model for this process of mastering pitch could be found in the Central African Pygmy music, where prevailing form of music-making is the collective vocalization of the entire tribe – notwithstanding the motherese and the children play music remaining monophonic (Rouget 2011). Growing children are gradually introduced to adult polyphony through 2- and 3-part singing. The course of the development of hypothetical proto-music likely followed suit.

1. Akia boy song, Amazon. This rare example, kindly provided by Anthony Seeger, compliments Ex.4 above, by demonstrating how the complex isophonic texture heard in that example was in fact constructed of the component songs at least some of which (like this one) were initially practiced solo. <http://chirb.it/HEC1th>

Primordial vocalizations were stochastic in pitch. Collective hunt-related vocalization homogenized rhythmic organization via entrainment mechanism, and enabled participants to align their “parts” vertically, in time. Rhythmically synchronized contours turned on the averaging mechanisms – both, motorically, while singing, and retroactively, while evaluating the contour produced by collective efforts. Negotiation of the average values between all the participants generated the conventional model of a contour, remembered in reference to a particular application for which vocalization was used.

Fine-tuning in pitch most likely initially occurred in private grooming vocalizations. Each individual must have developed his personal melodic manner that had to stay uniformed through all instances of grooming, serving as a personal identifier. Grooming vocalization could have evolved into motherese, which shares the function of care-taking and connotation of pleasure and love that are related to it. Subsequently, motherese vocalizations diverged into lullabies, playsongs, personal songs, and other genres, which induced at first khasmatonal, followed by ekmelic tonal organizations. Principles of vocal coordination, discovered through solo singing and mother/child responsorials, were then imported back into collective vocalizations. In this feedback process, “natural” primordial polyphony was acquiring its tuning: whatever intervals were discovered in “chamber” settings, polyphonic singing was converting horizontal intervals into vertical, respectively advancing harmonic organization (see Part-1).

Confirmation for this hypothesis is found in the absence of polyphonic samples of “timbral singing,” where vertical intervals would exhibit consistency in tuning.^[[2]](#footnote-2)^ In order for the non-periodic spectral content, generated by “dirty-sounding” false vocal folds (Lindestad et al. 2001), to clean up and produce periodic spectral content with clear pitch, it is crucial for the singer to maintain full control over the produced sound. Presence of any partner contributing “dirty” content would make it very difficult if not impossible to empirically develop self-control over the partials of one’s voice and establish vocal coordination necessary for “pure pitch” production by true vocal folds. The upgrade from what Eduard Alekseyev called “anthropophonic singing” (Latosh 2007, 17) to cleaner “bel canto” culture of sound production necessitates extensive experience of solo singing.

Acquisition of pitch coordination skills demands reduction of a number of simultaneously sounding parts in order to focus one’s attention on just two pitch contours: the source and the target – in order to match them. Only after the algorithms of matching are established is it possible to proceed to chunking information and increasing the number of simultaneously coordinated parts.

Once emmelic culture of vocal production is put in place, it encourages production of tuned multi-part music (see Part-1). Cultivation of multi-part singing, in turn, is likely to have eliminated pure khasmatonal and ekmelic modes, the traces of which survive mainly in cultures of the ethnicities of Extreme North, where polyphonic genres could not have evolved due to ecological reasons (scarcity of population).^[[3]](#footnote-3)^ The existing examples of multi-part music amongst the indigenous ethnicities of Siberia are limited either to the call and response scheme of a dance, such as Evenki sedye, or, come as a result of the interaction between a shaman and other participants in shamanic rites, as in Nganasan culture, in which case the same responsorial model is followed, although in a much more complex setting - featuring a medley of formulaic melodies, where each of the melodies is addressed to a specific spirit or deity (Dobzhanskaya 2007).

1. Audio: Evenki sedye, Indigirka region “responsorial” song-dance. <http://chirb.it/GEDEqq>
2. Audio: Osuokhai, Vilyuy River region, Yakutia. The collective song-dance utilizes variation of the same formula on every repetition by every participant, where only a single anchor tone is “tuned in,” while other tones keep “floating” in pitch (Tatarinova 2013). Such texture presents a metrically synchronized version of isophony. <http://chirb.it/hwFLH4>

A similar situation is found in deserts, like Central Australian Aboriginal music that contains ekmelic features of scalable intervals (Will 1997). In other ekmelic cultures, the tradition of singing-for-oneself can preserve the monophonic song – as in the Hopi Indian culture that contains both, ekmelic melody-making (List 1985) and tradition of individual and kinship songs (List 1997).

The responsorial setting further polished the modes, and enabled one to share one’s tuning style with the community, while negotiating the average tuning, attractive to everyone. In well-populated communities the primordial stochastic polyphony transformed into harmonically attuned polyphony, governed by a specific interval. “Jumbled texture” gave way to emmelic heterophonic and diphonic textures – distinguished by the degree of continuity and autonomy of parts: heterophonic parts each presents variants of the same tune, whereas diphony contains a second part that is different in its melodic material from the primary part, and demonstrates a certain level of accomplishment of its own melodic organization. This difference might not be well defined, in which case the divider is in whether the music is meant to present one melodic idea, or two: melodic agreement versus disagreement.

The first samples of diphony were likely to follow “step equivalence” (see Part-1) and therefore feature the 2^nd^ as a formative vertical interval.

1. Audio: Harvest Song, Shope Country, Bulgaria. 2-part polyphony based on 2^nds^ and unisons, with *otglas* at the end of each line. <http://goo.gl/rGt0sa>

A 2^nd^–based diphony must have set the model for a derivative clustered triphony (3-part texture).

1. Audio: Odkad seke nismo zapjevale, ganga, Bosnia. 3-part polyphony of “clustered” style, with common motion in parallel 2^nds^. <http://bit.ly/1epleTP>

The logical extension of this method would be a mechanical addition of equidistant steps according to the number of available participants, and if the number is too great, breaking the performers into groups, each assigned to carry out a single dedicated part in the texture. The criterion of what constitutes an excessive number of parts is, of course, cultural, however, it relies on the cognitive constraint in sharing attention between 3-4 simultaneously running parts (Stoter et al. 2013). Greater polyphony requires hierarchic organization of texture, with some parts carrying important musical material, while other parts – less important – so that the listener would use diagonal shifts of attention, skipping from one part to another, following some musical cues. In Western classical tradition this method is exemplified in imitative polyphonic techniques that characterize such genres as ricercar.

Folk music can also employ “compressing” compositional devices (i.e. imitation, ostinato, responsorial) to distribute thematically important musical material across different parts by permanently assigning an important melodic line to a specific part in the texture. In doing so, it can follow a numerical order from the top or bottom of the texture, and define the other parts in terms of their functionality to the principal part (Jordania 2006, 28).

The visual equivalent of simple diphonic texture would be an ensemble of two depicted objects, united by the subject of depiction and continuity of depicted space.

1. Two fighting rhinos, Chauvet, 30,000 BC. The ensemble of two images is created by the joint action of “fighting,” known from real life. <http://bit.ly/1RySBmw>

Another important trait of Paleolithic tonal and spatial organization is naturalism. The oldest discovered Aurignacian cave art is strikingly realistic: the unknown painter accurately converted visible contours of a real object into the contours of a pictorial image. Semantically, such representation corresponds to psychophysiological intonation in music (Sheikhin 2002, 30), which is characterized by imitation of sounds of nature (i.e. wind) and human/animal-produced sounds (i.e. flint-knapping or animal calls).

1. Audio: Geese Katajjait, Canada. Vocal imitation of geese cries. <http://bit.ly/1O63ywe>

Both, naturalistic music and depiction, must have been nourished by shamanistic ideology, based on the supposed cross-influence of the signified on the signifier, and vice versa (Hubbard 2003). Thus, in shamanic traditions of many Northern Siberian ethnicities, melodic and pictorial contours are believed to have power to affect the corresponding real objects (Novik 2004, 67–85). Great precaution characterizes use of figurative art in numerous hunter/gatherer societies of Siberia, Northern America, and South Asia: drawings that depict ritual images on tambourines are washed out upon completion of a rite, drawings on snow that accompany Eskimo story-telling are destroyed at the end of the story, faces drawn on masks are burned after their use (V.V.Ivanov 2014, 4:357). Similar precautions and taboos are found in relation to musical instruments, i.e. certain tambourines amongst different native Siberian ethnicities (Sheikhin 2002, 69-86).

Establishment of this supernatural connection made the sensory resemblance between natural objects and their human reproduction unnecessary. Naturalism is required only in the emerging phase of shamanic tradition: once the connection between an image and its real prototype is established, the depiction can become significantly simplified and stylized.

1. Cave Art of Animals and Bird on Stick with Human and Bison, Lascaux cave at Dordogne, France, c.17,000 BCE. The abstraction of the human image and of some shamanic accessory is collaged with a realistic animal picture. <http://bit.ly/1KinBTY>

Collage-like bordering of realistic and schematic images in a single picture finds a counterpart in shamanic deep throat singing tradition, widespread across Siberia and Far East. Its sound-production technique is designed to imitate “voices of nature” simultaneously with shaman’s natural “human” voice. This can be achieved in a variety of ways. The most obvious method is production of two distinct pitches by the same singer, either by generating subharmonic tones with the help of false vocal chords, or using resonance of the upper face to generate overtones. In both cases, a single singer manages to emit a heterophonic or diphonic texture, where the melodic line proceeds from one tone to another on a sustained fundamental drone.

1. Audio: Cave Spirits, Tuva. The *kargyraa* deep throat singing style, characterized by simultaneous engagement of the vocal and vestibular folds, creating a growling impression. <http://bit.ly/1R0kRiP>

Less obvious is the “collage of parts” achieved by an ongoing breaking of the voice. Thus, the Yakut *kylysakh* technique presents a peculiar bi-timbral “diphony solo” (quasi 2-part singing by a single performer) (Alekseyev 1976, 7–8), produced by quick “grace-note”-like leaps from the “overtone voice” to the normal voice – such peculiar breaking probably was initially reserved for communication with “dangerous” spirits (Novik 2004, 67–85) akin to an “auditory mask” for the shaman’s voice.

1. Audio: Song of the heaven shaman woman Aiyy Umsuur from olonkho Niurgun Bohotur, Sakha. The *kylysakh* style characterizes positive supernatural characters. Thus, in this epic chant, the shaman turns into a stark to bless Niurgun Bohotur before his battle with the underground bohotur (Alekseyev and Nikolayeva 1981, 25). <http://chirb.it/LCqqa1>

In supernatural interconnection, the extent of realism in reproduction did not matter – affording schematization of both, pictorial contour and melodic contour. Correspondingly, spectral resemblance between onomatopoeic natural prototypes (i.e. an animal call, sound of streaming water, see Part-1) and its musical imitation became less important, allowing for increase in periodic spectral content. This led to abstraction of pitches, and their crystallization in melodic intonations that maintained no resemblance to the initial prototype material, which had originally given birth to that musical genre.

1. Shamanic rite, from La Valltorta, Spain, ca. 6,000 BP. A stylized drawing of two human figures and some animal. <http://bit.ly/1SInKlU>
2. Audio: Tukuna boys and girls chant, Amazon Indians. A rare occasion of choral singing at a feast celebrating initiation of girls, where all the guests reproduce the same intonation falsetto to assure strength and protection from the evil forest spirits (Schultz and Chiara 1962). <http://bit.ly/1Q6zRvp>

Schematization of the contour towards the end of Paleolithic naturalism had nothing to do with the decline of technical skills. French caves retained schematic art side by side with realistic 3D sculptures, reliefs, and carvings of animals, such as L'Abri-du-Cap-Blanc, ca. 15,000 BP, or Roc aux Sorciers, ca. 14,000 BP. The cave located at La Marche contained many realistic depictions of human heads and figures, dated 15,000 BC. Schematization should be regarded as a *deliberate method of contour simplification in order to elaborate reliable categorization of shapes* in encoding of important information. The same definition applies to melodic schematization.

Pitch contour and intervallic typology could denote appropriate semantics by means of pure convention. Music progressed from psychophysiological echolaliac intonations to *organophonic* intonations (Sheikhin 2002, 30). In the same way that schematization simplified depiction, depriving it of realism, late Paleolithic music must have passed through simplification through thinning of its texture caused by the rationalization of pitch.

The next logical development in pictorial organization was continuity of objects designed to indicate an event by means of grouping together schematic pictorial representation of different objects.

1. Man gathering honey, from the Cuevas de la Araña en Bicorp, Valencia, Spain, ca. 8,000 BP. A schematic representation of a human figure with a vessel, tree with a hive and bees. <http://bit.ly/1N2kqk8>

The analogical arrangement of horizontal harmony would conjoin the successions of similar intonations into a continuous melodic line – especially, when the melodic line involves an ensemble of participants performing in a responsorial manner, consistently responding to one another, so that each melodic tone is harmonically influenced by the previous one.

1. Audio: Ahir Birha, Northern India and Nepal. This holler-style responsorial song represents an argument between two milkmen, and about the degree of sacredness of cow and river Gang (Junius 1972), illustrated by progressive escalation of tension. <http://bit.ly/1NABn6F>

The horizontally harmonized melodic line produces heterophony – short bifurcations and trifurcations from the principal melodic formula, designed to compliment singing preferences of each of the participants, and/or provide variety in multiple repetitions. Such texture differs from isophony by keeping all parts basically in-sync, limiting the discrepancies to the pitch domain rather than the time domain.

On the other hand, comparing to strict monophony, heterophony presents a thicker texture due to out-branching of voices. Heterophony *functionally opposes* monophony – which is often overlooked by musicologists.

- The cognitive idea of heterophony is individualizing a known melody in a collective performance.
- The cognitive idea of monophony is adherence to a single standard.

Both of these ideas have their place in life of a folk community.

1. Audio: Osuokhai, Vilyuy River region. A chain of heterophonic variants on the same short song-dance formula, performed by men’s choir during a festival. <http://chirb.it/hwFLH4>

Spatial equivalent of heterophony is such pictorial composition where one object in the ensemble masks another, thereby indicating which of them is closer to the observer. Akin to heterophony, which is the earliest and easiest form of vertical harmonization, “masking” is the earliest indicator of spatial harmonization - 3D representation onto 2D plane. It demonstrates that two depicted objects each occupy its own slot of space despite the superficial impression they give in sharing the same space. Just as heterophony was possible at earlier stage of ekmelic music, masking can be found in earliest forms of art – at first, in a form of a bas-relief, in Solutrean Fourneau du Diable, Bourdeilles, and later, in murals.

1. Font-de-Gaume bison and deer, 14,000 BC. The image of a deer is superimposed over that of a bison, queuing the depth parameter – perhaps, its earliest occurrence. <http://bit.ly/1IOhUdm>

Transition from ekmelic to emmelic organization, refreshes the isophonic texture by providing definite pitch for vertical coordination of parts. Interaction of isophony and heterophony is likely to have produced homophony – synchronized rendition of parts, comprised by dubbing the melodic line in a vertical interval that is retained most of the time throughout the music work. Such texture appears consistently thicker than heterophony, and more homogenous compared to isophony.

1. Audio: War Song, Côte d’Ivoire. Encouraging song for warriors, most of the time preserves the distance of a 3^rd^ between 2 parts. <http://bit.ly/1Jlz6aA>

Contrary to the belief of many musicologists, homophony is not exclusive to Western art music. Homophonic texture can be substantially thickened in a folk musical culture with prominent triad induction (see Part-I). Homophony can “naturally” grow into a chain of parallel triads (Kubik 1998) and even 7^th^–chords, performed in 4 parts: i.e. alternations between C-E-G-B and Db-F-Ab-C vertical harmonies recorded at Bigene village, Nola District, Central African Republic (Kubik 2005). The principal idea of homophony is the melodic agreement between multiple parts, where each part keeps its autonomy, yet it is disciplined to express a single melodic idea: in a way, plurality in unity.

The visual equivalent to folk homophonic organization^[[4]](#footnote-4)^ would be multiplication of exactly the same image, with minimal variations, organized into an ensemble within a contiguous pictorial space. Such pictorial device was commonly used to express the idea of abundance of natural resources or greatness of manpower of a tribe.

1. Antelopes and hands, Altamira, ca. 16,000-12,000 BC. Multiple images of clone-like antelopes probably signify abundance of food supply. <http://bit.ly/1Qaobrm>
2. La Valltorta cave, Spain 6,000 BP, warriors. Multiple schematic images of running humans with spears likely illustrate the power of a tribe. <http://bit.ly/1OdykO3>

The joined implementation of visual continuity and masking is known to lead to intuitive discovery of the bird’s view perspective – the idea of representing the parameter of depth by rising the vantage point so that it would be possible to look as though “over” the object in the foreground closest to the observer. The germ of such a perspective can be found in prehistoric cultures.

1. Rock art at [Balho](https://en.wikipedia.org/wiki/Balho), Djibouti, near Ethiopian border, Neolithic culture (Gutherz, Cros, and Lesur 2003). An elevated view at a herd of antelopes and two giraffes. <http://bit.ly/1m6Dm8C>
2. San bushman rock art, Ukahlamba Drakensberg Park, KwaZulu-Natal, South Africa, near Lesotho, ca. 1000 BP (Herbert 1998). An elevated view of a scene depicting the capturing of a “Rain-bull”. <http://bit.ly/1SIIMRt>

Noteworthy, both examples above belong to geographic areas where traditional polyphonic music is still in use – especially across South Africa, polyphonic textures are exceedingly common amongst almost all native ethnicities.

1. Audio: Gamo dance song music, female and male choir, Ethiopia. Functional polyphony. <http://bit.ly/1m7JT2S>
2. Audio: Dorze dance song music, female choir, Ethiopia. Functional polyphony. <http://bit.ly/1M01iPP>
3. Audio: Ntoa hae e loana ke mosebetsi, pea threshing song, Lesotho. Functional polyphony. <http://bit.ly/1NCWbuc>

Sustained repetition of short melodic patterns, meshed across different voices, and tiered at different fixed pitches within a predetermined scale characterizes polyphony of cultures of African Horn as well as of South Africa (Arom 2004, 307).

At the point of formation of functional emmelic polyphony, the development of musical texture reaches its limit within a folk culture without literacy and math-based theory (see Table-2). The next development occurs within the urban civilization with higher technology and hierarchical organization.

**Table 2**. **Evolution of the types of texture in relation to tonal and spatial organization, according to the rational coordination of pitch**. This table presents a hypothetical chronological order of development of typology of musical textures against the stages of tonal organization, highlighting the most important corresponding features between the tonal organization in those musical textures and the spatial organization in pictorial arts - as categorized in the research literature on history of arts.

| **Texture** | **Stage** | **Tonal Features** | **Spatial Features** |
| --- | --- | --- | --- |
| Ekmelic Isophony | Khasmatonal | Brief call, continuously reproduced by multiple performers with irrational deviations in timing and pitch, where each participant retains idiosyncrasy of the rhythmic, timbral, and directional attributes of the pitch contour – altogether producing a “jumbled” effect. | Disconnectedness of images, governed by reproduction of visual contours of a real prototype – with frequent cluttering and overlapping of newly made and earlier made images on the cave wall. |
| Monophony | Ekmelic | Brief formula, continuously repeated by a solo performer, with distinct timbral and pitch characteristics that keep modulating within a certain narrow range of values – representing an individual style of a singer in a manner similar to the unique sound of his voice. | Avoidance of clutter by reserving a dedicated pictorial space for each image; priority of naturalistic representation of a single object – single image per single prototype, emphasizing their “sameness”. |
| Heterophony | Oligotonal | Compact tune, collectively performed more or less in-sync, but with little deviations in pitch, so that overall the tune is recognizable as the same melodic entity by virtue of nearly perfect similarity of pitch, rhythm, and timbre between all the participants – emphasizing that all multiple personalities share a single melodic idea. | Indication of pictorial depth by means of one contour masking another, in imitation of a closer visual object blocking more distant objects – thereby representing not an object but a scene, where each object receives its own spatial spot while constituting part of a whole. |
| Emmelic Isophony | Oligotonal and Mesotonal | Same as the above, but with free distribution in time, so that overlapping of parts provides greater fluidity and continuity by masking the breaks between the end of a tune and the beginning of its repetition. | Same as the above, but with grouping of greater number of similar images together, producing greater continuity and more of the impression of a frontal view. |
| Diphony | Mesotonal | Same as the above, but with free distribution in pitch, so that the vertical intervals between the concurrent tones keep varying, while maintaining melodic continuity for each of the parts - presenting two melodic ideas. | Same as the above, but with more obvious ensemble relations formed between two different images, where each image is assigned a particular function different from that of another. |
| Triphony | Multitonal | Same as the above, but involving three similar parts, concurrently producing three different vertical intervals, and presenting three melodic ideas. | Same as the above, but with the ensemble of three different images, each executing its own function in the depicted scene. |
| Homophony | Multitonal, Pentatonic and Heptatonic | Perfectly synchronized rendition of all parts, comprised by dubbing the melodic line in a vertical interval where dubbing is retained throughout most of the musical work – representing a single melodic idea. | Multiplication of the exact same image, with minimal variations, organized into an ensemble within a contiguous pictorial space; each image executes the same function. |
| Functional Polyphony | (Pentatonic) Heptatonic | Contrast of functions of parts within the texture: i.e. one part melodically leading, another supporting, offsetting, complimenting or interfering, etc. – thereby generating multiple melodies, related to one another by some means that are sustained within a music work. | Implementation of the elevated panoramic view, as though looking from the “bird’s eye view,” to display the objects positioned behind the frontal images, so that images execute different functions in a scene. |
| Monody | MPS Diatonic | The same as monophony above, but performed by multiple vocals and/or instruments in mathematically verified tuning that is defined by reference of all the tones to a single tone and interval, octave equivalent pentachord-tetrachord division, and dominance of a single melodic line. | The same elevated panoramic view, but with mathematically verified canonic iconography: division of space in registers, alignment of images by the baseline and prevalence of frontal view in providing integrity for the picture. |
| Functional Heterophony | MPS Diatonic and MPS Chromatic | The same as heterophony above, but in mathematically verified tuning applied to continuously expanding non-formulaic melody (rather than a “tune”), where the parts would display functionality and autonomy: carrying a drone tone (possibly alternating between a few drone tones) or/and melodic dubs; marking the principal melody by more generous alterations. Listener appreciates such texture as an aesthetic object, from aside (rather than as a participant). | Based on the above, but following geometrically verifiable projections in marking the foreground and background of the picture, often engaging architecture; employing shading to indicate the depth aspect, and positioning the vantage point decidedly outside of the picture, conceiving the latter as an aesthetic object designed for appreciation from aside. |
| Modal Polyphony | Hypermode and Hemiolic Chromatic | Mathematically verified tuning optimally defines the horizontal melodic as well as the vertical harmonic aspects of the musical texture, providing all parts with equal opportunities for chromatic shading and textural functionality; listener becomes included in the texture by the task of ongoing orientation in very complex structures – when he has to keep tracking a certain thematic material amongst the parts. | Geometrically verifiable projections are used to mark the foreground and background of the picture and connect them by defining a specific vantage point that fixes the position of the viewer, thereby integrating him into the depicted image in a manner of a window; all pictorial objects are defined in relation to this point. |
| Modal Homophony | “Monality” (Wienpahl 1971) | Mathematically verified tuning that mediates chords in vertical harmony, and melodies in horizontal harmony, favoring certain vertical and horizontal combinations of tones, while excluding others; governed by the idea of harmonious distribution of the material in the virtual music space across all of its parameters. | Mathematically verified single-point linear perspective, relatively strictly observing the unity of the scale, singularity of the vantage point, and a little more flexible in the implementation of convergence points; governed by the idea of clarity of composition. |
| Homophony-Polyphony | Tonality | Mathematically verified tuning that mediates chords and melodies, allowing for unprecedented variety of functions and configurations of elements and components of texture; appreciation of originality in tonal organization; functionality of parts in texture and functionality of tones in horizontal harmony. | Mathematically verified linear perspective and shadow projection, allowing for deviations from the rules as well as spatial exaggerations; appreciation of originality in spatial organization; functionality of components in visual composition. |

Institution of diatonic MPS modes promoted rationalization of harmony, further flattening the texture by correcting the “barbaric’ (in eyes of civilized musicians) polyphony and heterophony. Codified and mathematically verified music theory together with notation produce a new type of heterophony – *orchestral* – where the discrepancies in pitch are caused not by personalization of a tune by an individual performer, but by technical discrepancies due to the difference in sound generation (i.e. blowing that sustains a tone versus plucking that requires frequent repetition of a sound in order to match the part of the wind instrument). Such disciplined melodic linear type of “art-music” production was first documented in Babylonian civilization, but must have originated earlier in urban Mesopotamian palace/temple art circa 3,000 BC. Its parallel in fine arts was the canonization of pictorial representation, most pronounced in Egyptian art.

1. Ostrich Palette, Hierakonpolis, Egypt c.3100 BC. Pre-canonic art. Pictographic representation iconically combines imitation of the real objects’ appearance and reference to what was known about them - in a rather free manner (Rauschenbach 1980, 30). <http://bit.ly/1RL6JG3>
2. Narmer Palette, Hierakonpolis, Egypt c.3000 BC. Canonic art. <http://bit.ly/1GUZto9>

It took about a century for the canonic iconography to elaborate a uniform code that governed Egyptian art for over 2,000 years until the Hellenic times:

1. division of space in registers in hierarchic scale to indicate relative importance of the images,
2. alignment by the baseline, and
3. prevalence of frontal view (Davis 1992, 1).

Egyptian pictorial canon is cognitively similar to the Babylonian MPS system that:

1. combined hierarchic organization with uniformed pentachord/tetrachord degree-based model,
2. was defined by a single tone (“tonic”) and single interval (tritone),
3. was realized through monodic melody (the same succession of tones performed by multiple vocals/instruments).

Both media apply a coherent set of rational rules to the repertory of compositional elements, in effect establishing a “mega-form” for expression of every culturally important content. Arnheim points out that Egyptian canon represented a decided method of orthogonal projection of 3D objects onto a 2D plane – was still preferred to perspectival representation, despite the latter being known to Egyptian artists at least from the 15^th^ century BC (Arnheim 1954, 75). Such preference owes itself to adherence to “abstract rationalism” of thinking (Hauser 1999a, 1:23), according to which aprioristic orthogonal projection provides greater ordering than empirical angular projections: the former imposes a single objective criterion, producing the same result for every artist, whereas the latter has to comply to multiple variable conditions, causing discrepancies between different artists.

Egyptian canonic art was the first school of pictorial representation that operated by means of geometric drafting: an artist planned his composition by securing the “correct” proportions and angles between the drawn figures (Lawlor 1982, 54). Mathematically verified pictorial composition served the same purpose as mathematically verified musical composition (known in neighboring Mesopotamia): both were seen as obtaining supernatural power through expression of divine numbers. In Egyptian iconography, gnomon (a sundial) and square were attributes of the chief deity, Osiris (72). In Sumerian iconography, the very symbol of royal authority was a measuring rod and line, often depicted in a scene of a god handing them to a king (Robson 2007).

Late prehistoric Egyptian culture split pictographic representation into hieroglyphic (dedicated to transmission of knowledge about objects) and fine art (dedicated to transmission of their appearance), where details in appearance of depicted objects acquired greater importance than the conceptual information about them. From earlier forms of depiction, this new iconography differed by its geometrically rationalized composition.

1. Middle Kingdom Egyptian wall painting of two humans with a plow. The picture is less informative and more “convincing” as to how plowing usually occurs. Nevertheless, the geometric features stand out: a 2-tier arrangement, where the upper tier’s figures are arranged in strict rhythm of equal interval and length, aligned by a straight ground-line that runs in parallel to the lower tier ground-line. <http://bit.ly/1KmTBrP>

In depiction, unimportant objects were stripped off of their informative layer more readily than were the important objects: both, Egyptian and Babylonian art, retained schematic representation of humans, while adopting naturalistic rendition of animals (Hauser 1999a, 1:23). Assyrian art became much more realistic than Egyptian art: presenting entire battle-scenes as snapshots at a climactic point, with figures captured in motion, in contrast to the Egyptian symbolic “reports” of the Pharaoh’s victory (Groenewegen-Frankfort 1951, 172). The need to preserve appearance eventually led to the emergence of illusionism - around the 18^th^ century BC, most evident in naturalistic erotic Babylonian reliefs, culminated in lead inlays from the decorative insets in the furniture of Assyrian elite, Tukulti-Ninurta I reign (Assante 2007).

1. Babylonian terracotta relief, erotic scene with a couple, 18^th^-17^th^ century BC. One of the earliest examples of naturalistic depiction of humans. <http://bit.ly/1Jj7Meo>

This is very close to the time of Hurrian hymns. The idea of evoking arousal by accurate topographic reproduction of naked human figures is not that far from the idea of evoking a state of joy by means of putting together melodic intonations known to produce joyful impression on people – this is what we learn about the musical reforms of Sumerian king Shulgi, 21^st^ c. BC: “not only that he knew how to play musical instruments, but he also knew how to cause joy with his music to those around him” (Klein 1981, 16). The new development, initiated by the Mesopotamian temple/palace culture, must have constituted taking “naturally” existing musical material from the folk tradition and faceting it so to make it serve new cultural functions.

We have already discussed the issue of opposition of folk and temple/palace musics in the Sumerian culture (Appendix-1). However, it is extremely doubtful if political rulers, like king Shulgi, could have instituted completely new artificial language of music based on only those idioms “invented” by the king himself, or by professional musicians, according to royal orders. It is much more likely that the already existing idioms were “edited” and configured according to some rationally inferred “grammar” – following the model of canonization of visual art in Ancient Egypt, which is relatively well known (Davis 1992). The task of generating a particular emotional state in the participants of a religious rite in order to “convince” a particular god in the necessity to act in a way that would be beneficial to them, must have caused some radical reshaping of the original folk material. The extent of this transformation can be seen in the development of the genre of lamentation in Sumerian culture (Löhnert 2011). Initially, closely related to pre-existing folkloric funeral laments, it quickly grew its own syntactic and semantic typology, elaborating its own “language” of expression (Cooper 2006).

In this sense, “composing” a song would constitute the “illusion” of a “naturally” existing folkloric prototype, semantically related to the subject of its musical expression. Such illusionism makes sense only for the creative output of a professional composer (West 1994, 171), hired to convey a certain emotional message. Folk musicians usually “speak for themselves” (see Appendix-3), expressing what they actually experience at the moment of performance, rather than impartially “deliver” someone else’s message – hence, they create the “real thing” and not its “illusion”. Therefore, musically evoking the state of joy (Dumbrill 1998, 142) by exposing the listener of Hurrian Hymn 6 to the intervals of the qablite mode (D-E-F-G-A-B-C-D-E), associated with the qablite ethos (Dumbrill 1998, 131) can be regarded as a form of “illusionism,” not that different from Babylonian terracotta reliefs. In both cases, emotions of the artifacts’ consumers are primed by conventional signifiers: configuration of contour lines on a 2D surface, and the intervallic makeup of the melodic line – to trigger the same psycho-physiological experience that they know from real life.

This pictorial illusionism, at the time knew no perspective in the strict sense: no unity of scale, viewpoint, or horizon. It was implemented intuitively on a sequential point-by-point basis in correspondence between the natural prototype and its graphic representation through multiple glances while sketching a drawing – with the “finish” added by the artist at a later point. Introduction of the category of “portraiture” that underlies such method of depiction – the image that highlights the most representative features of some real object, and one that is supposed to be judged in relation to its faithfulness to the original – was already in place in the early 1^st^ millennium BC, in Assyria (Winter 2009, 1:83).

The principal method of composition was the realistic reproduction of a close frontal view, encompassing all the neighboring objects in the foreground (Rauschenbach 2002, 187). This integration contrasted the earlier prehistoric methods of naturalistic depiction, which rarely united different figures in an ensemble, instead, pursuing the goal to capture a single object in motion (Okladnikov 1967, 117) – favoring animals (more important for the survival of Stone Age man) over humans (105). Otherwise, representation of depth was emblematic in, both, early Bronze and Stone Age depictions (117). It took about 5,000 years between the end of Paleolithic culture and the origin of a science-driven agricultural civilizations (Frolov 1992, 69) to arrive at the formulation of canonic illusionism. Emergence of register-based ensemble integration of still figures of humans should be regarded as the equivalent of transition from multitonal to pentatonic/heptatonic model of melodic harmony. They both involve harmonization of components: groups of figures, or trichords/tetrachords.

3D representation was discovered during the 18^th^ dynasty of Egypt and in Assyrian art:

1. animal and human figures were spread out across the available space, not standing on the same baseline, often arranged by registers (Aruz, Graff & Rakic 2014, 55);
2. distant objects were masked by the closer ones; or
3. placed higher - to form “ranks” in a bird’s-eye view (Russell 1987).
4. Sennacherib receiving the submission of Jews. Sennacherib’s Court 6 at Nineveh. Canonic bird’s eye perspective. <http://bit.ly/1Hr8SW5>

In addition to the 3 point above, even a more advanced device is occasionally found in Assyrian art:

4) the difference in scaling between close and distant figures is occasionally found in the reliefs at Ashurnasirpal Palace, anticipating later aerial perspective (Flittner 1958, 249).

1. Siege of the city. Palace of King Ashurnasirpal II. Rough scaling, suggestive of aerial perspective. <http://bit.ly/1LCjNit>

The musical analogs of this would be:

- 1. uniformed diatonic distribution of degrees and intervals in a mode, where each degree received mathematical verification rather than being defined linearly by melodic intonation (as in earlier forms of folk music);
  2. differentiation of ensemble performance, distinguishing between solo and supporting parts;
  3. in orchestral music, the parts of musical instruments were arranged to evenly fill up the available orchestral ambitus by each instrument covering a particular register;
  4. vertical intervals between the vocal melody and its instrumental accompaniment were “legitimized” by music theory and notation – rather than “sporadic” melodic inflections generated by folk heterophony.

Although we can only fantasize the sound of Babylonian/Assyrian music, it was unlikely to have featured significant heterophonic discrepancies between the parts: professionalization was relatively recent, public performance was canonic and under strict supervision – and there is no mentioning of a multi-part performance. Technical proficiency of performers must have been limited, since the Shulgi’s reform of instrumental techniques (Klein 1981) was rather recent. Hence, playing the same melody together “in tune” must have been the preferable manner of performance. The sacral status of music, closely supervised by professional administrators (Ziegler 2011), together with the existence of notated references for the core repertoire (Michalowski 2010) must have effectively reduced the amount of discrepancies, thereby flattening the melody close to perfect monody.

Ancient Greek texture probably was initially similar to the Assyrian texture – so as the spatial organization in the very first samples of Greek landscapes look about as flat as Assyrian ones.

1. Mural in the Tomb of the Diver, Paestum, Italy, 5^th^ century BC (Holloway 2006). Very flat landscape, reduced to visual contours. <http://bit.ly/1TMRcIb>

But eventually, Greek art developed hierarchic representation of depth, and Greek music became most certainly more heterophonic because of the spread of public music education and popularity of music competitions – which promoted technical advance in woodwind, string, and vocal performance, making their rendition of the same tune differ in details.

There is evidence that harmonic (vertical) intervals were familiar to Ancient Greeks. The Book of Problems of Aristotle contains eleven references to vertical intervals in relation to “problems” in harmony (Spiess 1959) – a sure indication of at least occasional use of vertical harmony. These references along with those from Plutarch’s “On Music” and Plato’s “Athenian Stranger” suggest that patterns of instrumental accompaniment to vocal singing was initially simple, but gradually became complex, possibly including the usage of harmonic intervals (2 tones of accompaniment per 1 vocal) and passages (Mathiesen 1999, 361–2). Andrew Barker sums up all the available evidence to build a case for heterophonic accompaniment to be regarded as a characteristic trait of Greek musical practice from earliest times throughout its history (Barker 1995). The heterophonic variants of the principal melody were regarded not as mere “embellishments” but as executing a particular function towards the principal melody. The intermittent dubbing of vocal melody was regarded as an auditory object in its own right. Diogenes of Babylon, 2^nd^ century BC, reports that “the hymns sung in Ephesus and by the choruses in Sparta were more impressive because accompaniment was added and so showed the power of music to move us” (Ferguson 2003, 399). Larger ensembles could have included drone (known as “ison” in later Byzantine music theory) as a point of modal reference for all participants (Daniélou 1995, 15). The tradition of Byzantine chant had Ancient Greek roots, descending from Greek-speaking monastic communities in Palestine (Jeffery 2001).

The ison in plainchant has comprised an organic part in the performance ensemble, determined by the modal structure of the chant, and therefore not notated. However, according to modern practitioners of Byzantine chant, “ison” is more than a drone tone: it often changes its position in pitch within a music work, and is regarded by performers as a discrete melodic entity, *in addition* to the principal melody of the chant (Koço 2013). Pretty much, the same applies to the heterophonic textures of folk cultures in the Balkan region (Koço 2015).

1. Audio: St. John Chrysostom - Axion Esti, Marian hymn of praise, 4^th^ c. AD. The melodic line receives support with a drone tone that is changed from time to time to accommodate singing in a new tetrachord – such practice found in today’s Greek and Syriac churches is likely to have originated much earlier. <http://bit.ly/1SQXogq>

There is no reason why heterophonic structures similar to ison could not have evolved in the instrumental music practice of ensemble performance of Ancient Greeks and medieval traditions that followed it. There is evidence of usage of instrumental ensembles in military Greek music (Franklin 2008) and at funerals (Bogdanov 2007).^[[5]](#footnote-5)^ Later practice of large (up to 600 people) festive choir performances (West 1992, 41) is likely to have produced organum-like dubs in parallel 4^ths^ and 5^ths^ that are known to naturally occur in choral performance whenever the high- and low-range vocals of musically untrained singers are used along (Tallmadge 1984). Something quite similar to this could have been implemented in instrumental music. Organum-like textures are still observed today in folk music of Pontiac Greeks who most frequently use progressions of parallel 4^ths^ and drone tones in playing lira (Ahrens 1973).

The resultant *functional heterophony* (unlike the homogenous heterophony of the earlier folk music) features complete autonomy of parts and their specialization in either grounding the melody, thickening it, or emphasizing particular portions of it. If comparatively similar number of performers would play a typical Western classical homophonic texture and functional heterophonic texture, the latter would still sound substantially thinner. However, such functional heterophony would surely produce a richer texture than the non-functional heterophony of folk music.

Correspondingly, the first survived samples of Classic Greek murals demonstrate significantly more detailed realistic “functional” (where an object is arranged in the composition according to the function of its prototype in real life) representation of a group of 3D objects onto the 2D surface, as compared to the Assyrian art.

1. Hades abducting Persephone, Vergina Tombs, a royal Macedonian burial complex, 4^th^ c. BC. Realistic organization of all 4 objects in an ensemble. <http://bit.ly/1RAeQZc>

Therefore, Greek “illusionism” surpassed Assyrian by a margin. Assyrian artists merely “reported” about certain events in their works, caring to choose such composition and attributes of its objects that would characterize the real prototypes of these objects throughout their lifetime rather than at the current moment of depiction. This caused a great deal of generalization in pictorial representation. Greek artists, from Hellenic times on, sought to represent a particular person or a particular event “as is” at the moment of happening – to capture the “momentum” in order to stir the viewer emotionally. Their illusionism obtained strong theatrical flavor, where it was necessary to convince the viewer in “reality” of the depiction to make him respond to the picture emotionally as though it was a real life event rather than its picture.

Hellenic musicians had similar task at their hands, having to make the listeners “feel” the song’s lyrics or the stories suggested by the title of the instrumental music (Power 2013). To be able to tell stories through music they had to build an arsenal of chromatic shades (see Appendix-3) – very much like their fellow-artists who discovered the expressive use of shadow.

Advances in enharmonic and chromatic music around the 5^th^ century BC coincided with the invention of “architectural painting” which employed perspectival images for home interior decorations and theatrical stage design. The painter Apollodorus was nicknamed by Plutarch “the shadow painter” for the introduction of shading technique (Little 1971, 1–10). Foreshortening of closer objects by means of placing extra shades works quite similarly to the addition of chromatic “shades” in pyknon degrees in tetrachords to give the vocal melody greater expressivity (Franklin 2005) – in a word, bringing out the principal vocal line from the other heterophonic parts (dubbing of an instrument or a drone tone) by virtue of chromatic alterations.^[[6]](#footnote-6)^

1. Seated woman playing a kithara, from Room H of the Villa of Fannius Synistor at Boscoreale, ca. 40 BC. Use of shading for 3D effect. <http://bit.ly/1FMbicp>

This “foreshortening” effect constitutes something different from perspectival organization, evident in the example above, where the legs of the chair do not match the angle suggested by the shading of the sitting woman. Shadows, cast by the sun, form orthogonal projections - in contrast to central projection required by representation of the disposition of 3D objects on a 2D plane – so, each projection has its own axis. In Greek tradition, these two aspects of organization were never integrated. Foreshortening of one object had little bearing on the foreshortening of another proximal object in pictorial composition (Ivins 1964, 32–33). In contrary, scaling of pictorial objects adjacent in their depth parameter was usually quite consistent in preserving the principle of reduction in size by increase of depicted distance.

1. Laestrygonians preparing for the battle*,* The Odyssey Landscapes, from the Esquiline Hill in Rome, 1^st^ c. BC. Not entirely consistent shadow casting and shading, while quite consistent scaling. <http://bit.ly/1RAgaLA>

Similarly, chromatic alteration in music should be thought of as a device that is different from musical texture in projecting spatial connotations. Multiple parts create a sense of “thickness” in music, whereas use of chromatic shades evokes an effect related to lighting – sort of highlighting the intervallic makeup of the melodic line.

- Differentiation of parts in heterophony reflects the *vertical* aspect of harmonization and corresponds to *scaling* in pictorial composition.
- Chromatic shading of diatonic degrees reflects the *horizontal* aspect of harmonization and corresponds to *shading* in pictorial composition.

Ancient Greek culture was the first to combine techniques of horizontal and vertical tonal harmony with 3D representation in pictorial composition. All three techniques shared a common origin from the applied discipline of *scenography* (Andersen 2008, 728–730). The sole purpose of scenography was to provide convincing emulation of known real objects in stage design of theatrical plays.

In theater, visual illusions accompanied auditory “illusions” in “aestheticized” reproductions of emotional states familiar to people through everyday life. Stage design “faked” the visual attributes of stimuli primed to trigger certain emotion, while tonal design “faked” their experiential attributes through complex mechanism of priming a particular emotional state by means of a tonal signal, such as a specific intervallic progression (Lippman 1964, 160) whose *harmonia* references a particular ethos (Mathiesen 1984).^[[7]](#footnote-7)^ Musical emotions came to provide a histrionic analog to real life emotions. Both scenic representations, visual and auditory, supported each other side by side in a single act-out event as a part of theatrical play.

Yet another important perceptional concomitant of perspectival organization in fine arts that received its analog in Ancient Greek music is the concept of the *frame*: presence of some kind of frame is necessary to delineate the virtual 3D space of a picture from the real 3D space surrounding it. This is where the semiotic approach of Ancient Greek and earlier civilizations come to differ. Greko-Roman architectural painting pioneered the same principle that later prevailed in Western civilization from Renaissance onward – the painter accepts an exterior position towards the depicted space. In contrast, the earlier artists, as a rule, favored an interior position, as though placing themselves inside the depicted virtual reality (Uspensky 1995, 173–181).

Noteworthy are those cases where painters depicted new images superimposed on the pre-existing images (quite common in cave art), as though believing that each of the layered entities enjoyed its immanent existence – disregarding whether or not they blocked each other in relation to the viewer. The reason for such disregard must have been the equal status assigned to the images and to the viewer. Inserted vantage point, evident in Ancient Egyptian and Assyrian landscapes, must have been the remnant of this older tradition. Such landscapes feature clear “*centrifugal*” orientation, where hills and trees on one side of the river are directed up, whereas on the other side – directed down, as if unleashing the energy from the center (Flittner 1958, 260).

1. Brick making, from the Rekhmire’s Tomb at Thebes, 18^th^ Dynasty. The pond is depicted from the point of view of a person in the middle of it: with trees on each side of the pond bent away from the center. <http://bit.ly/1KJZkcg>

We can infer the meaning of such orientation from its explanation in surviving medieval sources on orthodox iconography. The same centrifugal principle of orientation is described in instructional treatises “in reverse”: the right side of the picture is referred to as “left,” and vice versa – defining each side as a mirror image - from the position opposite to the actual viewer who becomes identified with the “virtual observer” inside the virtual depicted space (Uspensky 1995, 254). Reference to the “depth” dimension is treated in the same way: from the point of view of a person placed in the middle of the depicted space (299). This “interior” method of orientation by no means is limited to Russian and Byzantine canons: the inverted left/right orientation is found in analogical iconography of the Final Judgment of Catholic Church (saints at the “right” versus sinners at the “left” sides), as well as in Gothic tombs and Romanesque sculpture (Aleshkovsky 1972, 106, ff15).

The pathway of left/right inversion is traceable to the oldest cartography (Uspensky 1995, 255), and must be related to the earliest days of art. The left hand-prints constitute the overwhelming majority of hand-prints in cave murals of the Paleolithic cultures as well as in the modern Aborigines and Indian paintings – which are usually interpreted by scholars as a form of a signature of the author whose right hand is busy painting. However, this interpretation is doubtful because of the absence of individualism and the concept of authorship in syncretic cultures.

A more probable explanation is the usage of the left hand as an orientation cue unlocking the entry from the earthly world (left=evil) to the heavenly world (right=virtuous) (V.V.Ivanov 2014, 4:262–268). Linking the imprint of the painter to that of the virtual person could have had a magical connotation, activating the connection to a mythological person or the spirit of the dead (Uspensky 1995, 303). Egyptian canon also related *inversion* to the transfer between the “world of the living” and the “world of the dead,” the Dwat (inverted world), expressed in progressions of reciprocal elements (Lawlor 1982, 21).

In cases of interior vantage point, the function of framing is executed by the contrast in form between the central and the peripheral images, such as concave forms in the center and convex forms at the very margins of icons (180), or by the same building that is depicted from the interior angle at the center of the picture being shown from the exterior angle at both sides (181).

1. The Robe of Christ, from the Kremlin Cathedral of the Dormition, 17^th^ century. The combination of the interior at the center, and the exterior at the margins of the picture. <http://bit.ly/1O6CLyS>

This orientation could be interpreted as the product of the inclusive mindset of the pre-perspective artist, eager to tell us what is going on inside the building (contrary to the attitude of an external observer who does not enter the interior space of the building depicted by the painter, staying faithful to what is visible from the outside). The interior orientation, in fact, requires a *continuous mediation* between the virtual and the real worlds: the viewer has to mentally keep switching between his actual position and the viewpoint of the virtual observer implied inside the depicted space (Uspensky 1976, 231). The ongoing summation of these impressions, experienced through envisaging a pictorial composition from both, inside and outside, comprises a peculiar “self-contained” concept of space in the archaic picture. It also explains why the ancient architectural paintings were often not framed: the peripheral objects in the composition as though prevented the implied interior observer from “looking out” from his pictorial space and seeing beyond.

The perceptual equivalent for this interior orientation in music would be the ensemble performance typical for folk music, where the singer wraps his melody into the heterophonic or polyphonic texture,^[[8]](#footnote-8)^ as though *placing himself inside the composition* in the most direct sense of the word.

In contrast, exterior orientation would engage performance of one’s part in a polyphonic or homophonic composition created by someone else, where the performer has to learn his part and keep it in strict correspondence to the text designated by the composer – in essence, where the performer has to speak not for himself but from someone else’s script, while pretending as though the pronounced lines are his own. In such situation the performer is aware that his part constitutes an autonomous entity added by the composer (or tradition) to the rest of the composition. Awareness of “import” prevents intuitive appropriation and causes the performer to think of addition rather than integration of parts.

The borderline here is not always clear, since folk music might also involve doing “cover songs.” But such cases focus not on faithfulness to the original text, but on authenticity in rendition. In this case, the performer has substantial leeway in modifying the pitch, rhythm, and metric characteristics of the preexisting music, thereby assimilating its musical material to the extent that allows him to completely identify himself with the protagonist of the song and present the work as indeed “his own” remake rather than reproduction of someone else’s work.

Exterior orientation does not allow for complete enmeshment: the audience is usually aware of how the composition ought to sound, and expects more of an expressive “reading” of the familiar text, where the performer is aware that his identity is different from the identity of the author of the text. Hence, *the performer’s execution of some composer’s music does not really constitute the “music work”* – it only makes the music work perceptible for the public. It is a manifestation of the concept that exists *externally* to the performer in the form of a music score. A music expert does not need a performer to experience a particular musical composition: he can read the score and hear internally the sounds of music. Here, the performer himself occupies the position outside the composer’s creation – unlike the typical folk performer who effectively recomposes the existing composition, placing himself inside the content arranged and modified by himself.

The interior/exterior opposition also manifests itself in tonal organization of music. Understanding that the very same pitches can belong to this or that sister mode of the same MPS family necessarily involves distancing oneself from the experience of listening: one has to relate not only to what he hears now, but to what he has heard before, retrieving in his mind the sound of familiar modes and their corresponding ethos. Not only that, but also each mode has to be distanced from other modes. Such task invites the attitude of a spectator in a theatrical show – rather than that of a direct participant in the musical action – what characterizes the folk cultures.

Folk musician does not usually think in technical terms as to which mode to choose, and how one mode differs from another. He simply intuitively picks those modal formulas that correspond to his emotional state, conditioned by the genre association of a particular mode. His music-making typically occurs mainly “in the present,” and completely within the framework of a particular mode – thus, inside a designated tonal space. Music-making in the MPS system rarely fits in a single mode. More often than not, it involves a few modes, thereby, setting boundaries between modes and transition points. The *framework of one mode makes another mode appear foreign*, requiring chromatic modulation. Archaic music does not know such rigid framing in its tonal organization, affording “inclusive” treatment of sonic material by means of modal inflections (see Appendix-3). “Exclusive” treatment becomes the prerogative of musical key that accompanies the emergence of perspectival organization of pictorial images.

It can also be said that angular projection cultivated by Euclidian perspective (Veltman 1980) provides a cognitive advantage over earlier orthogonal systems of Mesopotamia and Egypt in encoding the depth parameter by making the depicted image less ambiguous. By showing an object slightly from aside, the lines that are supposed to depict the depth aspect become angled (rather than parallel) to the lines that depict the vertical aspect. This distinction significantly facilitates processing of the 3D spatial encoding in a 2D picture. Jan Deregowski rightfully calls attention to this important transition in the cognitive approach, distinguishing between the *outline* per se and *typical contour* of an object (Deregowski 2009). Ancient Greek artist preferred to arrange the projection to optimally represent the typical contour (rather than outline) as a more stable entity. What earlier artists were occasionally grasping intuitively became rationalized by Euclidian geometry and turned into a rule.

In a similar vein, the Ancient Greek musician must have started distinguishing between the melodic contour of a leading melody (as in vocals) and the melodic contour of an accompanying part (vocal or instrumental), optimizing the performance not by the principal melody alone, but by the *entirety* of the texture.

Once in place, notions of geometric perspective (formulated by Euclid) and musical key (formulated by Aristoxenus) maintained their connection throughout Western history.

The early Christian culture broke this tradition by flattening, both, texture in music, and depth in depiction, forming a new canon in iconography based on the idea of equality of all objects in the face of God and illusiveness of things mundane.

1. Audio: Tecum principium in die virtutis tue, Ambrosian chant, 5-6^th^ century AD. Strict monody with alternation of solo and choir became the principal type of texture in Christian music. <http://bit.ly/1OPRXxW>
2. The Three Magi, The Basilica di Sant'Apollinare Nuovo**,** Ravenna, 6^th^ c. AD. If pre-Constantinian Christian art imitated Euclidian perspective of non-Christian Roman art, after legalization of Christianity greater resources allowed to quickly develop a new iconographic style (Finney 1997, 225), often termed as “reverse perspective” (Deregowski 1993). <http://bit.ly/1I3jcmg>

Depiction here focused on commemorating spiritual values by means of sanctified abstract conventions – decidedly hostile to the illusionistic methods of Antiquity (Florensky 2006, 197–272). As hostile was the attitude of the Fathers of the Church to the musical “illusionism” and especially the music for aulos (McKinnon 1989, 34, 61, 72, 78), historically associated with Dyonisian style theatricality in Hellenic tradition (Franklin 2013). Plainchant canon, abstracted from the diatonic music of Platonic times, filtered out the expressive idioms of the heathen past. It forged its own pool of intonations in the chants approved by the Church authorities. It was codified and eventually fixed by notation – which in sharp contrast to the overwhelmingly hemitonic tradition of Antiquity, strongly relied on the pentatonic trichords (Hansen 1979).

The shift towards pentatony probably had to do with the intuitive discovery of the inherent property of anhemitonic intonations to reduce tonal tension (see Part-1) – a very important task for early Christian aesthetics. Fragments of the canonic melodies, collected in tonaries, have served as a source material for most of the music composed for ecclesiastic purposes in Medieval West – and extending far into Renaissance and Baroque epochs through the prevalent compositional technique of *cantus prius factus*.

Initially, very strong canonic rules restricted both, Christian iconography and monody during the early Middle Ages. Then, the rebirth of diphony and triphony out of monodic plainchant documented first in Musica enchiriadis (9^th^ century AD) accompanied the first experiments of liberating depiction from canonic rules, away from scholastic stylishness, and toward a more naturalistic and individualized manner (Schapiro 1952).

1. Journey to Bethlehem, Santa Maria foris portas. Castelseprio, 9^th^ century AD. Roman tradition refreshed by amalgamation of Byzantine and Carolingian styles (Leveto 1990). <http://bit.ly/1CGaaqD>

The growing culture of imitative and contrasting polyphony led to the emancipation of polyphonic technique in the 12^th^ century (Tischler 1956) and to the emergence of completely new polyphonic textures that reached unprecedented complexity by the 14^th^ century and that were especially complicated by the technique of hocket, which articulated phrases in such a manneristic style that even a 3-part polyphony was really hard to follow (Leech-Wilkinson 2003). Ultimately, Pope John XXII banned such polyphonic devices in 1324 (Wright 2008, 346). This ban stayed in effect until the 16^th^ century (Blackburn 2007, 90).

It took about a century for Western composers to resolve this mannersistic crisis. The switch from 4^th^-based to 3^rd^-based harmony in music of John Dunstable caught the attention of European composers (Bukofzer 1952) and allowed to greatly increase the number of parts, and to standardize handling of intervals, supporting simultaneous movement of multiple melodies. This upgrade in textural complexity concurred with the experiments of Masaccio in finding compositional schemes alternative to canonic iconography, capable to evoke a sense of lifelike movement, which prompted him to imitate 3-dimensional space in his paintings (Deimling 1995).

1. Audio: Dufay - Ecclesie Militantis, motet for 5 parts, utilizes 3 melodies, each with its own lyrics (one about militancy of Church, another about peace, and the third – omnipresence of God) – providing 2 supporting parts. <http://bit.ly/1IrIdVc>
2. Masaccio – The Tribute Money (1425). Use of linear and atmospheric perspective to emphasize Jesus’ head in unification of three episodes of the story: 1) the tax collector demanding tax from Jesus and his disciples, 2) St. Peter removing money from the fish’s mouth, 3) St. Peter paying the tax collector – all joined together by sharing a naturalistic cityscape background. <http://bit.ly/1HrKaEV>

An important consequence of the integrative effect of “chunking” musical tones into chords, and depicted objects - into representation of depth, was a marked increase in premeditation of the artistic composition. The painter had to envisage the image prior to sketching his work. The composer had to commit to a certain schemata of ambitus, certain style of rhythmic coordination of parts, and a certain principle of disposition of text through parts prior to the “compositional” phase of conception – such methodology of composition comes to light in the 15^th^ century, but is noticeable in earlier Ars Nova motets (Moll 1998).

Already in the 14^th^ century composers engaged in the pre-compositional phase in deciding the functionality of parts in the music texture based on the semantic attributes of lyrics in music. Sometimes this entailed visualization of the musical composition. The most striking demonstration of this can be found in unconventional notations: concentric circular, or in the shape of an object – such as a heart or a harp.

1. Anonymous – “En la maison Dedalus” for 3 parts (c.1377), the earliest example of circular notation (Berkeley, University of California Music Library, 744). <http://bit.ly/1U03Wxf>
2. Audio: Anonymous – Ballade “En la maison Dedalus,” voice solo with instrumental accompaniment of two polyphonic parts, where the upper part canonically imitates the lower part – circulating the same thematic material in defiance of the free-floating vocals. <http://bit.ly/20T9dvR>

Such notation was much more than a mere “cosmetic” choice for the unusual expression of conventional musical structures. Thus, the composer of “En la maison Dedalus” illustrated the lyrics that referred to the maze built by Dedalus for the Minotaur, by scoring his composition as an 11-course labyrinth, drawn with a pair of compasses (Leach 2011). The closest to the center part carries the “tune,” while the tenor and countertenor parts are notated as one part to be sung in canon where the tenor is chasing the countertenor (“Tenor faciens contratenorem alter alterum fugando”).^[[9]](#footnote-9)^ The reference to the labyrinth here serves as a metaphor for the content of a courtly poem that describes the suffering of a suicidal Lover to find his way to the “heart of his desire” (Wright 2004, 239) - expressed graphically as well as musically through the path of the melodic motion yet to be found under the pressure of the chasing voices of the accompaniment.

Matching visual and musical expressions to illuminate the text of a song maybe characterized as a stylistic trait discovered by artists of Ars subtilior: the quest for esoteric sophistication in appreciation of beauty of spatial arrangement of music texture (Smilansky 2011). Yet another famous example of expressive notation is “La harpe de melodie.”

1. Senleches - La harpe de melodie for 2 parts (1395), Chicago, Newberry Library, 54.1. Notation in the form of a harp, where notes are placed on strings, and the inscriptions on the banner wrapped around the fore pillar of the harp instructs the way to derive the second part from the strings. The accompanying text invites to see and hear the notes of the beautiful harp (Leach 2013). <http://bit.ly/20w8Z8q>
2. Audio: Senleches - La harpe de melodie, virelai. Typical for Western Medieval culture theme of courtly love is expressed here with emphasis on the need for privacy – suggesting that the pleasure from refined music and pleasure from refined company are both better off if concealed. <http://bit.ly/1PUjEIy>

This tradition of emblematic notation survived for quite a long time: John Bull’s 6-part circular canon “Sphera mundi” in an 18th-century manuscript, and canons for the Chapel Royal by Juan del Vado (1679). These should be viewed as an original way of conceiving the thematic material and the textural arrangement in a musical composition by engaging some spatial connotations. After Western music resorted to standardized textures, determined by the choice of the genre, there was no need to use weird notation, because spatial visualization of musical texture became as common as audiation of implied chords in a given melody. Composers started using conventional notation to execute unconventional “inventions” of texture, which were usually based on blurring the harmonic pulse by means of choosing a specific texture for the figuration of the accompaniment. Candace Brower gives a glimpse into such creativity: an accompaniment in Brahms’ Intermezzo in B minor, op.119, No.1, which is treated like a Möbius strip in Escher’s litographs.

1. Audio: Brahms – Intermezzo in B minor, op.119, No.1. Accumulation of 3^rds^ in the expressive legato figuration of the bass voice obscures the harmonic plan by making it unclear as to which exact chords are implied in a bar, and how exactly they are distributed between the beats. <http://bit.ly/1Tj3o6r>

Textures with such dispersed, lagging, or delayed harmonies in the figurations of one or more voices of the accompaniment became the “favorite” of the late Romantic piano composers, for instance Alexander Scriabin, who reflected his synesthetic vision of compositions as a dynamic interaction of multiple objects of different size, mass, and color, in a delimited area of space (Sabaneyev 2000).

Introduction of new methodology of pre-composition in the 14-15^th^ centuries eventually led to crystallization of a new listening style, characterized by appreciation of harmonic consonance and polyphonic complexity despite its occasional incomprehensibility (Wegman 2002).

1. Audio: Ducis – Agnus Dei, No.18 from Cantiones Triginta Selectissimae (1568). This motet for 8 parts features a quadruple retrograde canon: it makes parts 5-8 reproduce parts 1-4 in retrograde inversion (so that the first tone of part-1 is the last tone of part-5, etc.) – which is humanly impossible to perceive upon audition, no matter how well the listener knows the music. <http://bit.ly/1OSlWFk>

Respectively, “linear perspective” quickly turned from the artist’s scholarly fancy into the standard for consumption of visual fine-art (Edgerton 2009) despite extreme spatial complexity that became a trademark of some artists.

1. Tintoretto – The Origin of the Milky Way (1580). Tintoretto assembled this composition by the cosmographic allegory of the depicted figures to the complex system of curves, circles, and triangles, suggesting the mechanics of the celestial spheres (Bouleau 2014, 475) – doubtfully perceptible by the viewer. <http://bit.ly/1gsjYQW>

The textural standards of post-Renaissance music come surprisingly close to the perspectival standards of post-Renaissance art. Thus, tonal key is commonly realized through 3(4)-part polyphonic, 4-voice homophonic, and 3(4)-component polyphonic-homophonic texture which altogether cover absolute majority of the 18^th^ - mid-20^th^ century compositions (Kholopova 1979). Often, composers approached textural design creatively, conceiving an original conglomerate of voices/parts prior to laying out the music.

1. Audio: Haydn - "Uns sprießet Überfluss," No.6, from The Seasons (1799) features a 4-part vocal fugue with the orchestral accompaniment that mostly consists of 2 parts (bass and melody). This is an example of a creative textural decision where the composer has engineered a unique functional combination of parts. <http://bit.ly/1Ubemc4>

Similarly, a number of 18^th^ century artworks employ an unorthodox integration of the architectural interior on a wall with painted architectural ornaments, against a backdrop of a depicted scenery – with the addition of yet another architectural background.

1. Tiepolo – View of the east wall of Palazzo Labia, Venice (1750). The architectural interior here is incorporated into a spatial ensemble with a fresco “The Banquet of Cleopatra,” forming an original fantastic space. <http://bit.ly/1SmsLo9>

The entire Baroque culture might be characterized as a complex interaction of multiple constituent parts, where each part is rendered as an autonomous expressive entity, competing with other entities, and therefore producing contrast and conflict. Baroque polyphony differs from Renaissance polyphony by the overall smaller number of parts, yet greater individualization of the thematic material in each of the parts (Poultney 1996). Baroque fine art is recognized for its individualization of each of the component images and cluttering effect, uncharacteristic for Renaissance, usually combined with intense tonal contrast (Hauser 1999b, 2:106).

“Invention” of original spatial composition that would deliver “illusionistic” impression despite its originality is a trademark of Baroque art – pretty much in line with “invention” of original textural implementation of some thematic idea, exemplified in the musical genre of Baroque *invention*, in its evolution from Cesare Negri to J.S. Bach (Caldwell 2001).

An example of an equivalent pictorial application would be the genre of “ceiling painting,” which from Michelangelo to Le Brun has presented an arena for inventiveness of fantastic visions, in fact, often referred to as “Baroque,” in the sense of “bizarre” – a highly appreciated genre in Italy (Wittkower 1958, 481) and in France (Blunt 1957, 196). The connection to musical “invention” here was primarily tonal – through harmonic organization rather than music texture.

1. Pietro da Cortona - Triumph of the Barberini, Palazzo Barberini, Rome (1639). The stucco ornamentation creates a frame of reference for apparent realness of some figures that as though share “our” space with a stucco behind them, whereas other figures are either leaning on the stucco, or are farther away “behind” it. <http://bit.ly/1j9h1Wv>
2. Audio: Marini - Sonata d'inventione from *Sonate, symphonie con altre curiose e moderne inventioni* op.8 (1629). Demonstration of inventiveness in the development of unusual melodic and harmonic ideas – treated in a manner of *inventio* of the discipline of rhetoric. The musical development here is called to make a bizarre material appear “convincing” to the listener. <http://bit.ly/1Rrqjf6>

Paradoxically, quest for novel engineering ideas involved consolidation of the “standards” of spatial and tonal composition into a dedicated discipline that started taking progressively more and more “scientific” appearance. The “science” of perspective became an attribute of professionalism in fine arts. Already, Leonardo was writing that those who ignored perspective were like “maritime pilots that maneuvered their ships without rudder or compass – never certain of their output” (Bell 1992). Likewise, towards the end of the 17^th^ century, the theory of general bass and counterpoint acquired the status of “bar exam” for the wish-to-be-composers. The discipline of counterpoint put the vertical and horizontal aspects of harmonic organization under the same umbrella.

The switch of fashion from polyphonic to homophonic genres, which cut off the careers of J.S. Bach and Vivaldi, did not change the state of affairs in tonal organization. Neither harmonic nor textural complexities ended when contrapuntal compositional techniques went out of favor. Most typical textures of common practice period - *polyphonic-homophonic* textures - combine homophonic organization by subordinating all the parts and voices to a one-at-a-time leading melodic line (with possible dubbing), where each of the textural components receives its own discrete function in accompanying the main melody. The maximum number of components in an orchestral piece of music can reach seven:

1. principal melody;
2. its dub;
3. melodic counterpoint line (imitative or contrasting);
4. figuration of the accompaniment;
5. harmonic accompaniment (in chords or double-notes);
6. bass line;
7. pedal tones and pedal-like background layer (Kholopova 1979, 37).^[[10]](#footnote-10)^
8. Audio: Rimsky-Korsakov - Praise to Wilderness, from The Invisible City of Kitezh. The orchestral score features all 7 functional types of textural components in a single section of music form. <http://chirb.it/tfFmgE>

Very much like music theory that catered to the post-Renaissance composers’ aspiration to design original texture promoting greater and greater complexity, “classical” perspective became “tweaked” and “individualized” to afford more complex and original organization of components.

The equivalent of “texture thickness” in music for linear perspective is the number of vanishing points. Albertian perspective linked together the viewer and the depicted space by the strict tri-unity of scale, horizon, and viewpoint: vanishing point here anchored a system that “incarnated” the viewer, turning him into an organic part of the work as well as its “ruler” – gazing at the vanishing point effectively installed the viewer as the object of the composition (Bryson 1983, 106).

1. Raphael – Marriage of the Virgin (1504). “Frontal” one-point linear perspective integrates the viewer and the depicted objects in a most unobtrusive way – in a manner of “looking through the window.” <http://bit.ly/1LToEIY>

Technically speaking, it is the vantage axis between the observer’s eye and the vanishing point defined by the artist that pierces the pictorial space and reveals the number of objects placed along this axis. The “golden mean” of populating the available depth seemed to be about 4 ranks – provided that the painter did not shoot for the “crowd effect,” and supplied the interspatial objects with discrete characteristics.

The musical equivalent to such detailing would be the distinction between texture that is based on chordal accompaniment (“the crowd”) versus melodic counterpoint (“a supporting character”). An average of 3-4 textural components, occasionally simplified to just 2 or complicated by adding up to 6, is common for both, Western perspectival art and tonal music.

Mediterranean tonality (see Appendix-4) and Eastern traditions of pictorial spatial organization have never come close to the density of Western arts.

Islam, against popular conviction, neither cut with the Roman legacy nor banned drawing from nature – in fact, some religious authorities approved of picture-making.^[[11]](#footnote-11)^ In the beginning of the Islamic period, artists downgraded to earlier Hellenic-like style of representation of real life objects.

1. Hare from Fustad fragments, 10-12^th^ centuries. Hellenic influence is evident in Islamic Fatimid art, suggestive of depth and texture (Hoffman 2000). <http://bit.ly/1GMn2wd>
2. Audio: St. John of Damascus – Ya walidata-l-ilah (Canon on the Nativity), Melkite chant. The Byzantine chant was assimilated in variety of Middle Eastern communities, including translation of texts in Arabic. <http://bit.ly/1IPqFsG>

Like early Christianity, Islam also denounced *illusionism*, and gradually promoted a special style of spatial representation based on frontal and oblique parallel projection, without committing to a particular “point of view,” avoiding shades, relief, and minimizing scalar coordination (Raynaud 2009). Islamic artists were aware of the “open space,” and did not shun from its representation. But their method was decidedly non-illusionistic: devoid of horizon and vanishing points in an attempt to remove personal connection of the spectator to any anchor point in the horizon at the picture’s background – to make the picture look “impersonal” (Rauschenbach 1980, 196). A picture often merged a few discrete projections in a manner of a blueprint – presenting information unavailable from a single viewpoint (i.e. combining bird’s-eye view and worm’s-eye view), while maintaining continuity of the horizontal baseline (Orbay-Grignon 1996).

1. Entertainment in a Garden, Folio from a Khamsa of Amir Khusrau Dihlavi (16^th^ c.). Space here cannot be derived from a single glance at the picture, but requires continuous horizontal scanning. <http://bit.ly/1f0hR6c>

The frontage of the foreground took on a deciding role in representation of space, giving priority to largely 2D coordination of objects, subjecting the depth-dimension to homogeneity of colors and sizes, so that the eye would be encouraged to move sequentially through the foreground without trying to “pierce” it, often suggesting a spiral observation curve (Papadopoulo 1979, 458).

Such “superficial” motion very much corresponds to a highly “busy” and frequently “branching out” voices in the technique of implied polyphony in the maqam melodic line (Yanov-Yanovskaya 1984). Yet another source of polyphonized inflections is the inclusion of drones in solo improvisation: especially characteristic is sustaining the treble tones that are dissonant in relation to the drone, in contrast to shortening the consonant tones while building up the climax (Vinogradov 1973).

1. Audio: Shur on tar solo, Azerbaijanian mugam. 1) Use of drone tone, 2) implied polyphony, and 3) registral contrast, induce the impression of rich texture in a monodic music. <http://chirb.it/aKE1tw>

Multi-part techniques found in maqam-based music are not limited to the pedal type. According to Sarisozen, lute performers in central Anatolia often employ progressions of parallel 5^ths^ (just as double-pipe players in various parts of Turkey do), even solo flute players engage into a challenging technique of producing double-stop overtones – yet the most sophisticated organum-like 4^th^ or 5^th^ based polyphony are found in Anatolian songs and violin/bagpipe duos in North-Eastern Turkey (Picken 1953).

Some Central Asian offshoots of the maqam tradition make use of polyphonic textures based on instrumental accompaniment that repeats the ostinato figure, while the vocals execute the primary melodic line.

1. Audio: Maddoh, Pamir. The accompaniment on rubab (6-string lute) keeps repeating the melodic figure B-A#-B, with the drone tone E – in contrast to the freely unveiling vocal melody. <http://chirb.it/pOwvDI>

Such textures are definitely more sophisticated than those possible in Ancient Greek culture, but they still come nowhere close to even the simplest cases of Western Renaissance music. The same distance separates Western and Eastern spatial organization. Some of the manuscript illuminations from Persia and India feature big landscapes or many dozens of characters – but multiple objects never align along the vantage-to-vanishing points axis, and therefore do not produce hierarchic order, remaining all equally “superficial.”

In contrast, Renaissance artists and musicians were interested in “deep” content: confined to music’s middle voices or interim space of an artwork. Both of these media were vulnerable to saturation. Leonardo’s rule of 20-time reduction of the real object’s size (Kubovy 1988, 41) reflected a hard boundary beyond which the depicted detail would be too fine for the observer to see. Then, detail-rich compositions of smaller size would suffer from a strict one-point perspective - compressing the interior as the depth-distance increases, not only diminishing the “more distant” objects but also cramming them together (Arnheim 1983, 205).

The workaround was multiplication of vanishing points. Already Alberti’s pupils realized that strict tri-unity could produce a tilt in a vertical figure (Bouleau 2014, 25) or tension between the distant vanishing point and the foreground figures. Then, rising or lowering of the horizon, and placing a vanishing point on an important object to stress it would enhance the composition (Elkins 1996, 147). Multiple vanishing points allowed for a more economical distribution of objects, reserving more space for the foreground and compensating for it by flattening the background.

Renaissance painters often employed up to 3 horizons and 4 vanishing points, making one of them dominant (Rauschenbach 2002, 150).

1. Veronese – The Wedding at Cana (1563) uses 6 vanishing points to make the space appear airy despite a crowd of people populating it (Bouleau 2014, 26). <http://bit.ly/1f2MM1F>

Horizons varying in height, as well as multiple left/right vanishing points, allowed for placement of more objects between the foreground and the horizon, and were therefore synonymous with the practice of *basso continuo*: improvisation of the accompaniment on an instrument capable of playing multiple sounds at once. Technically speaking, basso continuo was a science of detecting implied harmony of the melody (melodies in polyphonic work) and filling up the distance between the melody and the bass line with chords and figurations.

By the end of the 17^th^ century, basso continuo was codified into the discipline of *figured bass* and was considered the backbone of professional composition. This was the first thing that a wish-to-be-a-composer had to study – pretty similar to how a wish-to-be-a-painter had to study perspective.

Many textbooks inaccurately describe basso continuo as a Baroque invention. But the origin of basso continuo dates back to Veronese’s times. It had evolved in practice of *stile moderno* (defined by Giulio Caccini in Le nuove musiche, 1602), which developed from the older basso seguento of *stile antico* (Schulenberg 1984). Bassi seguenti were realizations of all the vocal parts arranged for the organ as an accompaniment to choral and madrigal music. They were actually printed, starting from the 1590s, in “short” or “full” notation (Horsley 1977).^[[12]](#footnote-12)^ Sometimes the vocal bass part had long rests (i.e. in the 1580s Ferrara madrigal style), in which cases a free instrumental bass was added in the score (476), constituting a discrete extra part.

Virtuosic embellishments in vocal parts was another common source for the discrepancy with the basso continuo part. Consequently, the basso continuo turned into a “shadow” part, not always counted in the denomination of a composition (i.e. “sonata for violin solo” would in fact demand *two* extra instruments, viola da gamba and harpsichord, ignored in the title). The tones added by basso continuo would noticeably thicken the texture – however, without nominally adding an extra part. Such a trick functionally resembles multiplication of horizons and vanishing points in the fine art of the time.

Just as deviations from Alberti’s one-point perspective are already found in the works of his pupils, the roots of basso continuo are traceable to Palestrina – the staple of Renaissance polyphonic strict style – as well as in the music of other reputable polyphonists: Alessandro Striggio, Luis de Victoria, and Cristóbal de Morales (Barbieri 1994). Even earlier was the “proto-continuo” use of improvisatory repetition of the same formula in self-accompaniment for a solo song, in a manner of emulation of the Ancient Greek citharode performance, documented amongst the entertainments for the marriage of Cosimo I de’ Medici and Eleonora of Toledo in 1539 (Ashworth & O’Dette 2007). Examples of such formulas survive in tabulaturas for frottola collections and in the Bottegari *Lutebook* (1574).

The practice of thickening the texture by cramming more notes in the available tonal space was regulated in a way similar to tweaking the perspective. Application of multiple horizon lines concurred with and resembled the establishment of standardized schemes of tonal plan for the most important music forms of Baroque: fugue, sonata, and concerto. These schemes were based on 2-3 components (tonic-dominant or tonic-dominant-subdominant keys), with the prevalent tonic, and essentially tied the cadential plan to specific sections of the music form (Caplin 2001). In effect, the change of key let the composer refresh the PCS and bring in more chords that were not present in the original key.

Greater assortment of pitches to be used within the standardized chordal structures enriched the texture in a way quite similar to the way higher or lower horizon added importance to objects positioned in the lower or higher sector. Each of such adjusted objects received a “face-lift,” making it appear greater in size and therefore perceptually stand out. By the same token, multiple vanishing points worked as alternative “tonics” of keys that were reserved by the cadence plan. Vanishing points suggest vantage points. As the viewer receives more options for his viewing position, he is psychologically released from the function of impersonating the “lord” of the image, which implied certain alienation from the content of the image (Arnheim 1983, 194) – as though viewing it from aside, separated by a window, “seeing without been seen.” Consequently, introduction of *alternative oblique angles remove the window effect*, enabling the viewer to experience “being right there” - *sharing space* with the depicted objects.

Musical equivalent to this greater sense of presence was the “sensitive style” (*Empfindsamer Stil*) that originated in the 17^th^ century France, invented to counter-balance the Renaissance born “doctrine of affections” (*Affektenlehre*) by evoking “subjective” quick-passing feelings, intimate to the listener (Cowart 1984), as opposed to a single emotional state postulated for the entirety of a work by *Affektenlehre* (Lasocki 1978). Such change in musical emotions reflected greater illusionism in representation of real emotions – involving the listener in a mental “acting-out” of the situation where the listener is as though “there,” next to the stimulus responsible for triggering the emotion. Perceptually, this presence effect brought in the experience of minute fluctuations in the emotional state indicated by the details in the texture, changes of the musical material between the sections of the music form, and the tonal plan.

1. Audio: C.P.E. Bach – Trio-sonata C Minor, Wq. 161/1, H. 579 "Sangineus und Melancholicus" (1748). According to the foreword by the composer, the 1^st^ movement here presents the interaction between two persons of different temperament, where the Sanguinus is trying to snap the Melancholicus out of his melancholy. <http://bit.ly/1UY2vxx>
2. Audio: John Mundy - Fantasie No.3 from the Fitzwilliam Virginal Book (c.1600-1630). According to the remarks in the score, the music presents alternations of fair weather and storm (at 0:44), representing states of turbulence and calm. Comparing to the example above, here, each of the contrasting states is illustrated by less prominent melodic intonations, less patterned texture and tonal progressions, resulting in a more diffused and general rendition of each of the musical emotions. <http://bit.ly/1QefvQh>

Structurally, sensitive style was characterized by its resemblance to the “recitative,” with its diversity of rhythm, melody, and harmony (Helm 1972) - chromaticism being an important part of Empfindsamkeit accessories, associated with finesse in coloration in painting, seen as responsible for the projection of sensitivity and soul (Cowart 1987). Just as chromatic shades were incorporated into the 18^th^ century key to emphasize tonal hierarchy, thereby intensifying expression, much effort in French Academy of Arts went into building the unified theory of shading before shadow-casting was mathematically modeled and incorporated into perspectival theory by Girard Desargues, eventually spreading throughout Europe (Kaufmann 1975). Earlier use of shadow in art was rather controversial, and although recognized as a constituent of perspective organization already at Alberti’s times (Bauer 1987), de facto was treated by artists autonomously from a geometric perspective in compositional design, often in contradiction to perspectival organization (Casati 2004). Cognitive research confirms that cast-shadow perspective can perceptually overpower other forms of perspective (Dee 2011).

1. Caravaggio - Conversion on the Way to Damascus (1601). Highlight-shadow organization overpowers geometric perspective, creating a dramatic yet spatially distorted conflicting effect. <http://bit.ly/1HAPRR4>

Accordingly, the use of chromaticism in music before the 18^th^ century was sporadic, obscuring tonicity rather than emphasizing it (see Appendix-3). Noteworthy is chromatic explosion at the end of the 16^th^ century (i.e. Carlo Gesualdo) that coincided with the discovery of tenebrism by Caravaggio, which led to Baroque obsession with darkness (Rzepińska 1986).

1. Audio: Gesualdo – Madrigal Beltà, poi che t'assenti (1611). The chromatic shading here executes variety of expressive functions (from melodic embellishment or increase in tension to re-coloration of chords), operating on a phrase-to-phrase basis, making the overall tonal palette rather motley. <http://bit.ly/1GTO71z>
2. Audio: Kindermann – Ballet (c.1645). The chromatic shading here is tonally patterned to fit into implied chords that are diatonic to the key of E minor (I, II, III, IV, V, VII triads and 6^th^–chords) except modulations to the V degree. As a result, chromatic alteration does not dissipate the overall tonal unity, but rather enriches the tonal hues by adding tints and shades to them. <http://bit.ly/1GWiaEN>

Gradual tonalization of chromaticism throughout the 17^th^ century led to a substantial increase in uniformity of distribution of chromatic alterations across the tonal material that was used to comprise a musical composition.

1. Audio: Gesualdo - Omnes amici mei (1611), responsorium for 6 parts. The graphic visualization by Stephen Malinowski illustrates the tonal relations by harmonic coloring, where the circle of 5^ths^ is treated as a color wheel, so that closely related diatonic tones are assigned to closely related colors. <http://bit.ly/1MFU9uc>
2. Audio: Purcell - Chaconne ZT680 (1694) for harpsichord. Harmonic coloring here demonstrates a much greater tonal uniformity than in the Gesualdo, example above. <http://bit.ly/1Kxa3kS>

The moment composers learned to support tonality with chromaticism, the painters learned to conform cast-shadow perspective to linear perspective.

1. Le Seuer – Phaeton asking Apollo (1654). The perspectival organization here is done in accordance with the teaching of Abraham Bosse (Goldstein 1965), which was based on Girard Desargues’ geometric theory (Kaufmann 1975). <http://bit.ly/1L51fsb>

As we see, both, tonal organization and perspectival projection can be highly informative of the relations between the parts/voices in musical texture or inter-space objects of depiction – through gradations of crowdedness/emptiness, stability/instability, and highlight/shading in the musical as well as pictorial composition. The aesthetic ideas prevalent in a given culture manifest themselves quite strongly in, both, pictorial art and in music, disclosing pronounced historic stylistic changes from Antiquity to Modernity.

The historic development of fine art has led to the emergence of a highly effective method of capturing immensely complex visual data and encoding it in an intelligible and coherent manner that can be rationally learned and objectively discussed. The historic development of music has led to even more informatively dense method of encoding auditory data. The advantage of music over fine art in its capacity to convey information lies in its *temporal* domain. A picture has a dead limit of space that cannot encompass more discrete images than a certain minimal size – traditionally estimated as 20-time reduction of the real object’s size (Kubovy 1988, 41). Exceeding the limit by squeezing in as many objects as the brush would technically allow, renders the entire picture poorly comprehensible. A music work does not have such hard limits. Its temporal nature makes it a lot more flexible. A music work can last for a few hours and include thousands of auditory events. There are genres of music that can run for a few days, performed by shifting of the musicians – i.e. *Andalusī nūbah*. The amount of musical objects that can be shown throughout such performance could run to a million. And most of this information is normally absorbed by competent listeners.

Music is also informatively denser than verbal speech despite the latter being temporal as well. The advantage of music is that it simultaneously engages multiple voices and parts, the number of which in practice of Western music is routinely about 4-5 layers in texture. The most typical combination is:

1) the leading melody,

2) bass line,

3) melodic figuration of the accompaniment,

4) accompaniment in chords,

5) sustained pedal tones or chords.

This setting constitutes a stereotypical band in Western popular music and its local implementations in various ethnic cultures of today. Thus, this would be:

1) the singer,

2) the bass guitar part,

3) lead guitar,

4) rhythmic guitar,

5) electric keyboard playing pads.

In addition, often the choral group is engaged to imitate the soloist, present a different material or interact with the soloist in the manner of call and response.

There is nothing similar to textural organization in the way in which humans use speech for communication. Simultaneous talking of five different persons would make it impossible to understand what they are talking about – as long as each of them pronounces different words.

And music’s integrative edge does not stop here. Music conveys information simultaneously in 10 aspects of expression. Five of which are used very heavily in most forms of commonly used music:

1. Pitch idioms (such as “fanfare” or “lament” melodic intonation) are mediated by the pitch syntax (such as pitch contour and intervallic proximity);
2. Rhythm idioms (such as “punctured rhythm”) are mediated by the rhythm syntax (such as rules of grouping);
3. Metric idioms (such as binary and ternary meters) are mediated by the metric syntax (such as ostinato, syncopation, and alternation);
4. Harmonic idioms (such as a “major triad”) are mediated by the harmonic syntax (such as tonic and dominant);
5. Texture idioms (such as “melodic line,” “chords” or “figure of accompaniment”) are mediated by the textural syntax (such as polyphony, homophony, or heterophony).

Other expressive aspects also feature idioms, but lack in syntactic rules proprietary to that aspect – instead, relying on the syntax of the five primary aspects:

1. Dynamic idioms (such as “forte,” “piano,” or “sforzando”) are not connected to one another by dynamic means – instead, they depend on harmonic, metric, pitch, and to a lesser extent, rhythm syntax;
2. Tempo idioms (such as “ritenuto” or “accelerando”) do not follow any tempo-based logic – their appropriateness is decided by pitch, rhythm, and harmony;
3. Articulation idioms (such as “staccato” or “legato”) are not limited by any articulation restrictions, they can be easily combined or alternated – determined by all five primary aspects of expression, plus tempo;
4. Timbral idioms (such as “vibrato” or “con sordino”) highlight the pitch, rhythm, harmonic, and to a lesser extent, tempo and articulation;
5. Idioms of music form (such as “introduction,” “exposition,” or “development”) are integrated from all nine aspects and all syntaxes.

Although this remains to be tested, however, it seems that a minute of music is capable of transmitting substantially more information than a minute of speech.

It looks like the phonetic organization in speech, tonal organization in music, and spatial organization in pictures, all had their head start around the same time, and shared about the same capacity to compress information. But throughout the evolution of human civilization and by power of modern globalization, tonal organization has developed a lead in capacity to effectively transmit high volumes of information amongst large number of people. This advantage must be one of the main reasons for the wide spread and the great importance laid on music in the modern day culture (Rideout, Roberts, and Foehr 2010).

REFERENCES:

Ahrens, Christian. 1973. “Polyphony in Touloum Playing by the Pontic Greeks.” *Yearbook of the International Folk Music Council* 5: 122. doi:10.2307/767498.

Alekseyev, Eduard. 1976. *Problems in Genesis of Mode [Проблемы формирования лада]*. Moscow: Muzyka [Музыка].

Alekseyev, Eduard, and Nadezhda Nikolayeva. 1981. *Samples of Yakut Vocal Folklore [Образцы якутского песенного фольклора]*. Yakutsk: Academy of Sciences of USSR.

Aleshkovsky, Mark. 1972. “Russian Enkoleons of Gleb and Boris, 1072-1150 [Русские глебоборисовские энколпионы 1072–1150 годов].” In *Ancient Russian Art. Artistic Tradition of Pre-Mongolian Russia [Древнерусское искусство. Художественная культура домонгольской Руси]*, edited by V. Lazarev, G. Vagner, M. Ilyin, and O. Podobedova, 6:104–25. Moscow: Nauka.

Andersen, Kirsti. 2008. *The Geometry of an Art: The History of the Mathematical Theory of Perspective from Alberti to Monge*. Berlin: Springer.

Aristotle, and Robert Mayhew. 2011. *Problems*. Vol. 1. Cambridge, MA: Harvard University Press.

Arnheim, Rudolf. 1954. *Art and Visual Perception: A Psychology of the Creative Eye*. Berkeley, CA: University of California Press.

———. 1977. “Perception of Perspective Pictorial Space from Different Viewing Points.” *Leonardo* 10 (4): 283–88.

———. 1983. *The Power of the Center: A Study of Composition in the Visual Arts*. Berkeley, CA: University of California Press.

Arom, Simha. 2004. *African Polyphony and Polyrhythm: Musical Structure and Methodology*. Translated by Martin Thom, Barbara Tuckett, and Raymond Boyd. Cambridge: Cambridge University Press.

Aruz, Joan, Sarah B. Graff, and Yelena Rakic. 2014. *Assyria to Iberia at the Dawn of the Classical Age*. New York: Metropolitan Museum of Art.

Ashik, Anton. 1845. *Kerch Antiquities: On the Frescos of Pantekapean Catacomb [Керченские древности. О Пантикапейской катакомбе, украшенной фресками]*. Odessa: A. Brown.

Ashworth, Jack, and Paul O’Dette. 2007. “Proto-Continuo.” In *A Performer’s Guide to Renaissance Music*, edited by Jeffery Kite-Powell, 2nd ed., 225–37. Bloomington, IN: Indiana University Press.

Assante, Julia. 2007. “The Lead Inlays of Tukulti-Ninurta I: Pornography as Imperial Strategy.” In *Ancient Near Eastern Art in Context*, edited by Jack Cheng and Marian H. Feldman, 369–407. Leiden, Boston: Brill Academic Pub.

Barbieri, Patrizio. 1994. “On a Continuo Organ Part Attributed to Palestrina.” *Early Music* 22 (4): 587–605.

Barbour, James Murray. 2004. *Tuning and Temperament: A Historical Survey*. New York: Dover Publications.

Barker, Andrew. 1995. “Heterophonia and Poikilia: Accompaniments to Greek Melody.” In *Mousike. Metrica Ritmica E Musica Greca*, edited by Bruno Gentili and Franca Perusino, 41–60. Pisa, Rome: Istituti editoriali e poligrafici internazionali.

Bauer, George. 1987. “Experimental Shadow Casting and the Early History of Perspective.” *Art Bulletin* 69 (2): 211–20.

Bell, Janis C. 1992. “Filippo Gagliardi on Leonardo’s Perspective.” *Academia Leonardi Vinci* 5: 115–19.

Berger, Karol. 2006. “Concepts and Developments in Music Theory.” In *European Music, 1520-1640*, edited by James Haar, 304–28. Woodbridge, CT: Boydell Press.

Bicheool, Vladimir. 2013. “Material Culture of Taimyr Nomads (on the Example of Samodiysky peoples)[Материальная культура кочевников Таймыра (на примере самодийских народов) ].” *The Courier of the Cheliabinsk State Academy of Culture and Arts* 1 (33): 170–75.

Blackburn, Bonnie J. 2007. “How to Sin in Music.” In *Music as Social and Cultural Practice: Essays in Honour of Reinhard Strohm*, edited by Melania Bucciarelli and Berta Joncus, 86–94. Woodbridge, CT: Boydell & Brewer.

Blunt, Anthony. 1957. *Art and Architecture in France, 1500-1700*. Edited by Nikolaus Pevsner. New York: Penguin Books.

Bogdanov, V.A. 2007. “Prehistory of Ukranian Musical Art of Wind Instruments [Предыстория духового музыкального искусства Украины].” *Bulletin of the Kharkiv State Academy of Design and Arts: Art.* 12 (2): 11–20.

Boorman, Stanley. 2005. *Ottaviano Petrucci: Catalogue Raisonne*. Oxford: Oxford University Press.

Bouleau, Charles. 2014. *The Painter’s Secret Geometry: A Study of Composition in Art*. Translated by Jonathan Griffin. New York: Dover Publications.

Bryson, Norman. 1983. *Vision and Painting: The Logic of the Gaze*. Yale University Press.

Bukofzer, Manfred F. 1952. “John Dunstable: A Quincentenary Report.” *The Musical Quarterly* 40 (1): 29–49.

Caldwell, John. 2001. “Invention.” Edited by Stanley Sadie and John Tyrrell. *The New Grove Dictionary of Music and Musicians*. London: Macmillan Publishers.

Caplin, William E. 2001. “The Classical Sonata Exposition: Cadential Goals and Form-Functional Plans.” *Tijdschrift Voor Muziektheorie* 6 (3): 195–209.

Casati, Roberto. 2004. “Methodological Issues in the Study of the Depiction of Cast Shadows: A Case Study in the Relationships Between Art and Cognition.” *The Journal of Aesthetics and Art Criticism* 62 (2): 163–74.

Comberiati, Carmelo Peter. 1983. “On the Threshold of Homophony: Texture in Sixteenth‐century Lute Music.” *Journal of Musicological Research* 4 (3-4): 331–51. doi:10.1080/01411898308574535.

Cooper, Jerrold S. 2006. “Genre, Gender, and the Sumerian Lamentation.” *Journal of Cuneiform Studies* 58: 39–47.

Cowart, Georgia. 1984. “Sense and Sensibility in Eighteenth-Century Musical Thought.” *Acta Musicologica* 56 (2): 251–66.

———. 1987. “Critical Language and Musical Thought in the Seventeenth and Eighteenth Centuries.” *College Music Symposium* 27: 14–29.

Crickmore, Leon. 2009. “The Tonal Systems of Mesopotamia and Ancient Greece: Some Similarities and Differences.” In *The Archaeomusicological Review of the Ancient Near East*, edited by Richard Dumbrill and Myriam Marcetteau, 1:1–16. London.

Crocker, Richard L. 1967. “A New Source for Medieval Music Theory.” *Acta Musicologica* 39 (3/4): 161. doi:10.2307/932351.

Daniélou, Alain. 1995. *Music and the Power of Sound: The Influence of Tuning and Interval on Consciousness*. Rep Sub ed. Rochester, Vt: Inner Traditions.

Davis, Whitney. 1992. *Masking the Blow: The Scene of Representation in Late Prehistoric Egyptian Art*. Berkeley, CA: University of California Press.

Dee, Hannah M. 2011. “The Perception and Content of Cast Shadows: An Interdisciplinary Review.” *Spatial Cognition and Computation* 11 (3): 226–53.

Deimling, Barbara. 1995. “Early Renaissance Art in Florence and Central Italy.” In *The Art of the Italian Renaissance: Architecture, Sculpture, Painting, Drawing*, edited by Rolf Toman, 244–46. Koln, Germany: Könemann.

Deregowski, Jan B. 1993. *Cognition and Culture - A Cross-Cultural Approach to Cognitive Psychology*. *Advances in Psychology*. Vol. 103. Advances in Psychology. Elsevier. doi:10.1016/S0166.

———. 2009. “On the Perception and Depiction of Three Essential Characteristics of Objects.” *Cultural-Historical Psychology* 2008 (1): 83–94. http://psyjournals.ru/en/kip/2008/n1/Deregowski.shtml.

Dobzhanskaya, Oksana. 2007. “Shaman Rite as a Syncretic Phenomenon: On the Matter of Function of Ritual Music [Шаманский обряд как синкретическое явление: к вопросу о роли ритуальной музыки].” *The Courier of the Tambov University* 53 (9): 257–62. http://cyberleninka.ru/article/n/shamanskiy-obryad-kak-sinkreticheskoe-yavlenie-k-voprosu-o-roli-ritualnoy-muzyki.

Dumbrill, Richard. 1998. *The Musicology and Organology of the Ancient Near East*. London: Tadema Press.

Eastham, Michael. 2005. “The Archaeology, Anthropology and Aesthetics of Understanding Parietal Rock Images at La Grèze, Cosquer and Wangewangen.” In *Aesthetics and Rock Art*, edited by Thomas Heyd and John Clegg, 89–116. Farnham, UK: Ashgate.

Edgerton, Samuel Y. 2009. *The Mirror, the Window, and the Telescope: How Renaissance Linear Perspective Changed Our Vision of the Universe*. Ithaca, NY: Cornell University Press.

Elkins, James. 1996. *The Poetics of Perspective*. Ithaca, NY: Cornell University Press.

Ferguson, Everett. 2003. “The Art of Praise: Philo and Philodemus on Music.” In *Early Christianity and Classical Culture: Comparative Studies in Honor of Abraham J. Malherbe*, edited by Abraham J. Malherbe, John T. Fitzgerald, Thomas H. Olbricht, and L. Michael White, 391–428. Leiden, The Netherlands: Brill. https://books.google.com/books?id=UEwRAQAAIAAJ.

Finney, Paul Corby. 1997. *The Invisible God: The Earliest Christians on Art*. Oxford: Oxford University Press.

Flittner, Natalia. 1958. *Culture and Art of Mesopotamia and Neighboring Countries [Культура и искусство Двуречья и соседних стран]*. Moscow: Iskusstvo [Искусство].

Florensky, Pavel. 2006. *Beyond Vision: Essays on the Perception of Art*. London: Reaktion Books.

Franklin, John Curtis. 2005. “Hearing Greek Microtones.” *Ancient Greek Music in Performance. Vienna: Wiener Studien Beiheft* 29: 9–50.

———. 2008. “Realizations in Ancient Greek Music Beyond the Fragments.” In *Challenges and Objectives in Music Archaeology*, edited by A. A. Both, R. Eichmann, E. Hickmann, and L.-C. Koch, 323–26. Westf.: Rahden.

———. 2013. “Song-Benders of Circular Choruses’- Dithyramb and the ‘Demise of Music.” In *Song Culture and Social Change: The Contexts of Dithyramb*, edited by P. Wilson and B. Kowalzig, 213–36. Oxford: Oxford University Press.

Frolov, Boris A. 1992. *Primitive Graphics of Europe [Первобытная графика Европы]*. Moscow: Nauka.

Goldstein, Carl. 1965. “Studies in Seventeenth Century French Art Theory and Ceiling Painting.” *The Art Bulletin* 47 (2): 231–56. doi:10.2307/3048258.

Groenewegen-Frankfort, Henriette Antonia. 1951. *Arrest and Movement: An Essay on Space and Time in the Representational Art of the Ancient Near East*. Cambridge, MA: Harvard University Press.

Gutherz, Xavier, Jean-Paul Cros, and Joséphine Lesur. 2003. “The Discovery of New Rock Paintings in the Horn of Africa: The Rockshelters of Las Geel, Republic of Somalil.” In *Journal of African Archaeology*, edited by Sonja Magnavita and Peter Breunig, 1-2:227–36. Frankfurt am Main: Africa Manga Verlag. doi:10.3213/1612-1651-100011.

Hansen, Finn Egeland. 1979. *The Grammar of Gregorian Tonality: An Investigation Based on the Repertory in Codex H 159, Montpellier*. Translated by Shirley Larsen. Vol. 2. Copenhagen: Aarhus Universitet.

Hauser, Arnold. 1999a. *The Social History of Art: From Prehistoric Times to the Middle Ages*. Vol. 1. New York: Psychology Press.

———. 1999b. *Social History of Art: Renaissance, Mannerism, Baroque*. Vol. 2. New York: Psychology Press.

Helm, Eugene. 1972. “The ‘Hamlet’ Fantasy and the Literary Element in C. P. E. Bach’s Music.” *Musical Quarterly* 58 (2): 277–96. doi:10.1093/mq/LVIII.2.277.

Herbert, Karin. 1998. *Rock Art of South-Eastern South Africa and Lesotho, 1806 to 1997: An Annotated Bibliography*. Natal Muse. Pietermaritzburg: Natal Museum Council.

Hoffman, Eva R. 2000. “The Beginnings of the Illustrated Arabic Book: An Intersection between Art and Scholarship.” *Muqarnas* 17 (May): 37–52. doi:10.2307/1523289.

Holloway, R. Ross. 2006. “The Tomb of the Diver.” *American Journal of Archaeology* 110 (3): 365–88. doi:10.3764/aja.110.3.365.

Horsley, Imogene. 1977. “Full and Short Scores in the Accompaniment of Italian Church Music in the Early Baroque.” *Journal of the American Musicological Society*.

Hubbard, Timothy L. 2003. “Further Correspondences and Similarities of Shamanism and Cognitive Science: Mental Representation, Implicit Processing, and Cognitive Structures.” *Anthropology of Consciousness* 14 (1): 40–74.

Hudson, Richard. 1970. “The Concept of Mode in Italian Guitar Music during the First Half of the 17th Century.” *Acta Musicologica* 42 (3-4): 163–83.

Huyghe, René. 1966. *Larousse Encyclopedia of Prehistoric and Ancient Art: Art and Mankind*. London: Hamlyn.

Ivanov, Viatcheslav V. 2014. *Selected Works on Semiotics and History of Culture. Semiotic Systems of Culture, Arts and Science [Избранные труды по семиотике и истории культуры. Знаковые системы культуры, искусства и науки]*. Vol. 4. Smolensk, Russia: Languages of Slavic Cultures.

Ivins, William Mills. 1964. *Art & Geometry; a Study in Space Intuitions*. New York: Dover Publications.

Jeffery, Peter. 2001. “The Earliest Oktōēchoi: The Role of Jerusalem and Palestine in the Beginnings of Modal Ordering.” In *The Study of Medieval Chant: Paths and Bridges, East and West: In Honor of Kenneth Levy*, edited by Peter Jeffery, 147–210. Woodbridge, Suffolk, UK: Boydell & Brewer Ltd.

Jordania, Joseph. 2006. *Who Asked the First Question? The Origins of Human Choral Singing, Intelligence, Language and Speech*. *The Origins of Human Choral Singing, Intelligence, …*. Tbilisi, Georgia: Logos.

Junius, Manfred. 1972. *India: North Indian Folk Music*. Paris: Auvidis-UNESCO. doi:UNES08033_103.

Kaufmann, Thomas de Costa. 1975. “The Perspective of Shadows: The History of the Theory of Shadow Projection.” *Journal of the Warburg and Courtauld Institutes* 38: 258–87.

Kennedy, George Alexander. 2003. *Progymnasmata: Greek Textbooks of Prose Composition and Rhetoric*. Leiden, The Netherlands: Brill.

Kholopov, Yurii. 1988. *Harmony: A theoretic course [Гармония: теоретический курс]*. Moscow: Muzyka [Музыка].

Kholopova, Valentina. 1979. *Texture: An Essay [Фактура: Очерк]*. Moscow: Muzyka [Музыка].

Klein, Jacob. 1981. “The Royal Hymns of Shulgi King of Ur: Man ’ S Quest for Immortal Fame.” *Transactions of the American Philosophical Society* 71 (7). New Philosophical Society: 1–48.

Koço, Eno. 2013. “Byzantine Chant, the Ison, and Arbëresh Liturgical Chant.” In *Local and Global Understandings of Creativities: Byzantine Chant, the Ison, and Arbëresh Liturgical Chant*, 266–81. Cambridge, UK: Cambridge Scholars Publishing.

———. 2015. *A Journey of the Vocal Iso(n)*. Cambridge, UK: Cambridge Scholars Publishing.

Kubik, Gerhard. 1998. “Central Africa: An Introduction.” Edited by Bruno Nettl, Ruth M. Stone, James Porter, and Timothy Rice. *The Garland Encyclopedia of World Music: Africa*. New York: Garland Pub.

———. 2005. “The African Matrix in Jazz Harmonic Practices.” *Black Music Research Journal* 25 (1/2): 167–222. http://www.jstor.org/stable/30039290.

———. 2010. *Theory of African Music, Volume 2*. Chicago, IL: University of Chicago Press.

Kubovy, Michael. 1988. *The Psychology of Perspective and Renaissance Art*. New York: Cambridge University Press.

Kumar, Sukhbinder, William Sedley, Kirill V. Nourski, Hiroto Kawasaki, Hiroyuki Oya, Roy D. Patterson, Matthew A. Howard, K. Friston, and Timothy D. Griffiths. 2011. “Predictive Coding and Pitch Processing in the Auditory Cortex.” *Journal of Cognitive Neuroscience* 23 (10): 3084–94.

Lasocki, David. 1978. “Quantz and the Passions: Theory and Practice.” *Early Music* 6 (4): 556–67.

Latosh, Yekaterina. 2007. *Moscow Conservatory; Biographic Encyclopedic Dictionary: From Its Foundation to Today 1866-2006 [Московская консерватория. Биографический энциклопедический словарь. От истоков до наших дней]*. Moscow: Moscow Conservatory [Московская государственная консерватория имени П.И. Чайковского].

Lawlor, Robert. 1982. *Sacred Geometry: Philosophy and Practice*. London: Thames and Hudson.

Leach, Elizabeth E. 2011. “The Fourteenth Century.” In *The Cambridge Companion to Medieval Music*, edited by Mark Everist, 87–103. Cambridge, UK: Cambridge University Press.

———. 2013. “The Sound of Beauty.” In *Beauty*, edited by Lauren Arrington, Zoe Leinhardt, and Philip Dawid, 72–98. Cambridge, UK: Cambridge University Press.

Leech-Wilkinson, D. 2003. “Articulating Ars Subtilior Song.” *Early Music* XXXI (1): 6–19. doi:10.1093/earlyj/XXXI.1.6.

Lester, Joel. 1989. *Between Modes and Keys: German Theory, 1592-1802*. New York: Pendragon Press.

Leveto, Paula D. 1990. “The Marian Theme of the Frescoes in S. Maria at Castelseprio.” *The Art Bulletin* 72 (3): 393. doi:10.2307/3045748.

Lindestad, P. A., M. Södersten, Björn Merker, and S. Granqvist. 2001. “Voice Source Characteristics in Mongolian ‘Throat Singing’ Studied with High-Speed Imaging Technique, Acoustic Spectra, and Inverse Filtering.” *Journal of Voice* 15 (1): 78–85. doi:10.1016/S0892-1997(01)00008-X.

Lippman, Edward Arthur. 1964. *Musical Thought in Ancient Greece*. New York: Da Capo Press.

List, George. 1985. “Hopi Melodic Concepts.” *Journal of the American Musicological Society* 38 (1): 143–52.

———. 1997. “Hopi Kachina Dance Songs: Concepts and Context.” *Ethnomusicology* 41 (3): 413. doi:10.2307/852758.

Little, Alan M. 1971. *Roman Perspective Painting and the Ancient Stage*. Kennebunk, Maine: Star Press.

Löhnert, Anne. 2011. “Manipulating the Gods: Lamenting in Context.” In *The Oxford Handbook of Cuneiform Culture*, edited by Karen Radner and Eleanor Robson, 402–17. Oxford: Oxford University Press.

Losev, Aleksey. 2000. *History of Ancient Aesthetics: High Classics [История античной эстетики: Высокая классика]*. Vol. 3. Moscow: Folio.

Lowinsky, Edward E. 1962. *Tonality and Atonality in Sixteenth-Century Music*. Berkeley, CA: University of California Press.

Mangani, Marco, and Daniele Sabaino. 2008. “Tonal Types and Modal Attributions in Late Renaissance Polyphony: New Observations.” *Acta Musicologica* 80 (2): 231. http://www.jstor.org/stable/27793364.

Marshack, Alexander. 1996. “A Middle Paleolithic Symbolic Composition From the Golan Heights: The Earliest Known Depictive Image.” *Current Anthropology* 37 (2): 357. doi:10.1086/204499.

Mathiesen, Thomas J. 1984. “Harmonia and Ethos in Ancient Greek Music.” *Journal of Musicology* 3 (3): 264–79.

———. 1999. *Apollo’s Lyre: Greek Music and Music Theory in Antiquity and the Middle Ages*. Lincoln, NE: University of Nebraska Press.

McCreless, Patrick. 2002. “Music and Rhetoric.” In *The Cambridge History of Western Music Theory*, 847–79. Cambridge: Cambridge University Press.

McKinnon, James W. 1989. *Music in Early Christian Literature*. Cambridge, UK: Cambridge University Press.

Michalowski, Piotr. 2010. “Learning Music: Schooling, Apprenticeship, and Gender in Early Mesopotamia.” In *Musiker Und Tradierung Studien Zur Rolle von Musikern Bei Der Verschriftlichung Und Tradierung von Literarischen Werken*, edited by R. Pruzsinszky and D. Shehata, 8:199–239. Vienna: Lit Verlag.

Moll, Kevin N. 1998. “Voice Function, Sonority, and Contrapuntal Procedure in Late Medieval Polyphony.” *Current Musicology* 64 (64): 26.

Murphy, James Jerome. 1981. *Rhetoric in the Middle Ages: A History of Rhetorical Theory from Saint Augustine to the Renaissance*. Berkeley, CA: University of California Press.

Novik, Yelena. 2004. *Rite and Folklore in Siberian Shamanism: An Experiment in Correlation of Structures [Обряд и фольклор в сибирском шаманизме: Опыт сопоставления структур]*. Moscow: Eastern Literature, Russian Academy of Science [Восточная литература РАН].

Okladnikov, Aleksey. 1967. *Morning of Arts [Утро искусства]*. Leningrad: Art [Искусство].

Orbay-Grignon, Iffet. 1996. “Remarks on the Concept of Pictorial Space in Islamic Painting.” *METU* 16 (2): 45–58.

Palisca, Claude V. 1956. “Vincenzo Galilei’s Counterpoint Treatise: A Code for the‘ Seconda Pratica.’” *Journal of the American Musicological Society* 9 (2): 81–96.

Panofsky, Erwin. 1991. *Perspective as Symbolic Form*. Translated by Christopher S. Wood. Cambridge, MA: MIT Press.

Papadopoulo, Alexandre. 1979. *Islam and Muslim Art*. Translated by Robert Erich Wolf. H. N. Abrams.

Picken, Laurence. 1953. “Instrumental Polyphonic Folk Music in Asia Minor.” *Proceedings of the Royal Musical Association* 80 (1): 73–86. doi:10.1093/jrma/80.1.73.

Pirina, Caterina. 1985. “Michelangelo and the Music and Mathematics of His Time.” *The Art Bulletin* 67 (3): 368–82. doi:10.1080/00043079.1985.10788278.

Poultney, David. 1996. *Studying Music History: Learning, Reasoning, and Writing about Music History and Literature*. Prentice Hall.

Power, Timothy. 2013. “Kyklops Kitharoidos: Dithyramb and Nomos in Play.” In *Song Culture and Social Change: The Contexts of Dithyramb*, edited by P. Wilson and B. Kowalzig, 237–56. New York: Oxford University Press.

Rauschenbach, Boris. 1980. *Spatial Constructsin Fine Art: Overview of Main Methods [Пространственные построения в живописи: Очерк основных методов]*. Moscow: Nauka.

———. 2002. *Geometry of Picture and Optical Perception [Геометрия картины и зрительное восприятие]*. Sanct-Petersburg: Azbuka-Klassika.

Raynaud, Dominique. 2009. “Why Did Geometrical Optics Not Lead to Perspective in Medieval Islam.” In *Raymond Boudon. A Life in Sociology*, edited by M. Cherkaoui and P. Hamilton, 243–66. Oxford UK: Bardwell Press.

Richter, Gisela. 1970. *Perspective in Greek and Roman Art*. London: Phaidon Press.

Rideout, Victoria J, Donald F. Roberts, and Ulla G. Foehr. 2010. *Generation M: Media in the Lives of 8-18 Year Olds*. Menlo Park, CA: Kaiser Family Foundation. http://kff.org/other/generation-m-media-in-the-lives-of/.

Robson, Eleanor. 2007. “Literacy, Numeracy and the State in Early Mesopotamia.” In *Literacy and the State in the Ancient Mediterranean*, edited by K. Lomas, R.D. Whitehouse, and J.B. Wilkins, 37–50. London: Accordia Research Institute.

Rouget, Gilbert. 2011. “Musical Efficacy: Musicking to Survive-The Case of the Pygmies.” *Yearbook for Traditional Music*. doi:10.5921/yeartradmusi.43.0089.

Russell, John. 1987. “Bulls for the Palace and Order in the Empire: The Sculptural Program of Sennacherib’s Court VI at Nineveh.” *Art Bulletin* 69 (4): 520–39. doi:10.2307/3050997.

Rzepińska, Maria. 1986. “Tenebrism in Baroque Painting and Its Ideological Background.” *Artibus et Historiae* 7 (13): 91–112.

Sabaneyev, Leonid. 2000. *Reminiscences on Scriabin [Воспоминания о Скрябине]*. Moscow: Klassika-XXI.

Schapiro, Meyer. 1952. “The Fresco Cycle of S. Maria Di Castelsepri. Kurt Weitzmann’s Book Review.” *The Art Bulletin* 34 (2): 147–63.

Schulenberg, David. 1984. “Before Composition Rameau: Figured and Style in the Baroque.” *College Music Symposium* 24 (2): 130–48.

Schultz, Harald, and Vilma Chiara. 1962. *Anthology of Brazilian Indian Music*. New York: Smithsonian Folkways Recordings. doi:FW04311 / FE 4311.

Seeger, Anthony. 2004. *Why Suyá Sing: A Musical Anthropology of an Amazonian People*. Chicago, IL: University of Illinois Press.

Sheikin, Yurii. 2002. *History of music culture of Siberia peoples: comparative-historic investigation [История музыкальной культуры народов Сибири: сравнительно-историческое исследование]*. Moscow: Eastern Literature, Russian Academy of Science [Восточная литература РАН].

Smilansky, Uri. 2011. “A Labyrinth of Spaces: Page, Performance and Music in Late Medieval French Culture.” In *Ritual and Space in the Middle Ages: Proceedings of the 2009 Harlaxton Symposium*, edited by Frances Andrews, 130–47. Donington, UK: Shaun Tyas.

Spiess, Lincoln Bunce. 1959. “The Diatonic ‘Chromaticism’ of the ‘Enchiriadis’ Treatises.” *Journal of the American Musicological Society* 12 (1): 1–6. doi:10.2307/829512.

Stoter, Fabian-Robert, Michael Schoeffler, Bernd Edler, and Jurgen Herre. 2013. “Human Ability of Counting the Number of Instruments in Polyphonic Music.” *The Journal of the Acoustical Society of America* 133 (5): 3366–3366.

Tallmadge, William H. 1984. “Folk Organum: A Study of Origins.” *American Music* 2 (3): 47–65. doi:10.2307/3052005.

Tatarinova, Aleksandra. 2013. “Pitch Organization of the Vocalizations of Yakut Circle Dance Osuokhai [Звуковысотная организация напевов якутского кругового танца оһуохай ].” *Historical, Philosophical, Political and Law Sciences, Culturology and Study of Art. Issues of Theory and Practice* 12 (2): 179–86.

Tischler, Hans. 1956. “The Evolution of the Harmonic Style in the Notre-Dame Motet.” *Acta Musicologica* 28 (3): 87. doi:10.2307/931976.

Tschopik, Harry Jr. 1954. *Indian Music of the Upper Amazon*. New York: Folkways Records. doi:FW04458 / FE 4458.

Uspensky, Boris. 1976. “The Language of Ancient Painting.” *Dispositio* 1 (3): 219–46.

———. 1995. *Semiotics of Art [Семиотика искусства]*. Moscow: Languages of Russian Culture.

Veltman, Kim. 1980. “Panofsky’s Perspective: A Half Century Later.” In *La Prospettiva Rinascimentale: Codificazione E Trasgressioni*, edited by Marisa Emiliani, 565–84. Florence: Stampa Stiave.

Vinogradov, Viktor. 1973. “The Notes on Middle Asian Polyphony [Заметки о среднеазиатском многоголосии].” In *Music of Peoples of Asia and Africa [Музыка народов Азии и Африки]*, edited by Viktor Vinogradov, 2:98–127. Moscow: Sovetskii Kompozitor [Советский композитор].

Wegman, Rob C. 2002. “‘Musical Understanding’ in the 15th Century.” *Early Music* 30 (1): 46–67. doi:10.1093/em/30.1.46.

West, Martin L. 1992. *Ancient Greek Music*. New York, London: Oxford University Press.

Wienpahl, Robert W. 1971. “Modality, Monality and Tonality in the Sixteenth and Seventeenth Centuries: I.” *Music and Letters* 52 (4): 407–17. doi:10.1093/ml/LII.4.407.

Will, Udo. 1997. “Two Types of Octave Relationships in Central Australian Vocal Music?” *Musicology Australia* 20 (1): 6–14. doi:10.1080/08145857.1997.10415970.

Winter, Irene. 2009. *On Art in the Ancient Near East*. Vol. 1. Leiden, The Netherlands: Brill Academic Pub.

Wittkower, Rudolf. 1958. *Art and Architecture in Italy: 1600-1750*. New York: Penguin Books.

Wright, Craig. 2004. *The Maze and the Warrior: Symbols in Architecture, Theology, and Music*. Cambridge, MA: Harvard University Press. https://books.google.com/books?id=LanWAAAAMAAJ.

———. 2008. *Music and Ceremony at Notre Dame of Paris, 500-1550*. Cambridge University Press.

Yanov-Yanovskaya, Natalia. 1984. “At the Origin of Polyphony in Uzbek Music [У истоков многоголосия узбекской музыки].” In *Music of Peoples of Asia and Africa [Музыка народов Азии и Африки]*, edited by Viktor Vinogradov, 4:11–46. Moscow: Soviet Composer [Советский композитор].

Ziegler, Nele. 2011. “Music, the Work of Professionals.” In *The Oxford Handbook of Cuneiform Culture*, edited by Karen Radner and Eleanor Robson, 288–312. Oxford: Oxford University Press.

1. Such music raises the question of how short a melodic phrase of each of the performers can be in order to produce segregation into parts? Limiting a phrase to a single tone produces not melodic lines but “melodic points” in a peculiar pointillistic texture. [↑](#footnote-ref-1)
2. In fact, the very possibility of segregation of melodic streams into 2 parts remains to be tested in relation to “timbral music”: ongoing timbral modulations of the pitch-bend material might very well prevent discrimination of the vertical intervals between participating parts and make them appear irrationally “cluttered” rather than proportionally spaced. [↑](#footnote-ref-2)
3. Very few Aboriginal cultures of Northern Siberia engage in collective hunting, and such hunting is usually seasonal, such as Nganasan hunting wild reindeers during their crossing of a river (Bicheool 2013). [↑](#footnote-ref-3)
4. This should not be confused with “homophony” of the Western classical music, which usually entails functional differentiation of homophonic parts (i.e. bass, melodic voice, and different types of accompaniment, such as ostinato versus pedal) – although the exact equivalent of the folk homophony can be found in the Baroque genre of “chorale.” [↑](#footnote-ref-4)
5. The 4^th^ century BC fresco, discovered in 1842 in Kerch catacombs (Ashik 1845) and subsequently destroyed, contained the depiction of a funeral procession with the accompaniment of music jointly performed by two groups of musicians: 1) two trumpets with a pan flute and 2) four trumpets. It is highly unlikely for these seven instruments to have played in perfect unison at all times. [↑](#footnote-ref-5)
6. Although shades of chromaticism and shading in pictures share a common origin, once the pictorial shading technique received life of its own, it followed its own course of development. Thus, Roman architectural painting kept elaborating shading until eventually it integrated into a single vanishing point perspective (Little 1971) – while the chromatic and enharmonic genera in Roman music went into near oblivion by the 4^th^ century AD. [↑](#footnote-ref-6)
7. Pseudo-Aristotle lists a number of such “tonal signals”: i.e. Hypodorian mode refers to a magnificent and steadfast character, most suited for kithara song; whereas Hypophrygian mode refers to action, such as military marching, that is appropriate for an actor on stage – however, both modes are inappropriate to the chorus, which requires quiet and mournful music (Aristotle and Mayhew 2011, 1:577). [↑](#footnote-ref-7)
8. This is if he accompanies his own singing on a musical instrument – otherwise, his collaborators wrap his melody into their parts, if this is a collective performance. [↑](#footnote-ref-8)
9. The transcription of this ballade in modern notation is made by Richard Crocker (Crocker 1967). [↑](#footnote-ref-9)
10. Not all of these components have to be engaged simultaneously at any given point in time – more common is the “diagonal” method, when only 3 parts (melody, bass, and one of the accompanying voices) run throughout the composition, while 1-2 extra parts/voices appear to be temporarily replacing each other, thereby, keeping the number of simultaneously active information streams to 4-5 (i.e. Beethoven – Adagio from Sonata Pathetique). [↑](#footnote-ref-10)
11. Fatimid book of illustrations c. 11^th^ century includes even realistic depiction of a nude with lute and cup (probably with wine) in hands (Hoffman 2000). [↑](#footnote-ref-11)
12. In 1609, in Conclusioni nel suono dell'organo, Op.20, Adriano Banchieri recommended that the organist plays the bass part and add a kind of accompaniment suggested by *basso seguente*, above it. [↑](#footnote-ref-12)
