# Supplementary material for: Evolution of Tonal Organization in Music Optimizes Neural Mechanisms in Symbolic Encoding of Perceptual Reality. Part-2: Ancient to Seventeenth Century
Source: Front Psychol. 2016 Mar 30;7:211. doi: 10.3389/fpsyg.2016.00211 (PMC4813086; doi:10.3389/fpsyg.2016.00211)
Supplement: Supplementary file 3 [file DataSheet1.zip › Appendices I-VIII/Appendix VI. Comparison of the principal features in types of tonal organization.docx]

## Appendix VI. Comparison of the principal features in types of tonal organization:

|  | PS | PSC | OD | IS | ISC | GS in PS | Attraction | Mod./Alt. |
| --- | --- | --- | --- | --- | --- | --- | --- | --- |
| Pre-Mode | Indefinite pitch without any degrees | Affording any pitch value within the range of an entire vocal compass | Timbre-frequency syncretism of a snapshot-like pitch contour (no degrees) | The entire compass works as a single interval, with reference to its center | No intervallic typology | Gradual (gliding) connection in timbre and pitch unifies tones | Attraction of tones to the start or end points in the pitch contour | - |
| Khasmatonal Mode | 2-3 degrees of Indefinite pitch, distinguished by register | Affording any pitch value within the range of a particular register of the vocal compass, defined by singer’s timbral preferences | Spectral consistency of a tone in the vocal register marks it as an approximate “registral degree” | Intervals are rounded to distances between 2-3 vocal registers, each marked by a strong timbral contrast | Contrast of gradual vs. abrupt timbral/pitch changes defines 2 intervallic classes: proximal vs. distant | Spectrally similar tones bind the melodic contour, obtaining the anchoring function | Mutual attraction of tones within the same register, and their repulsion from tones from another register | - |
| Ekmelic  Mode | 2-4 degrees of Indefinite pitch, distinguished by melodic functionality | Affording any pitch value within the range of a dedicated region of a vocal register, defined by change in melodic direction and rhythmo-metric stress | Frequency consistency over a specific range (2^nd^ – 4^th^), defined by directional shifts and stops in relation to melodic contour | More definite unison & 2^nd^ contrast scalable 3^rd^ & 4^th^, all defined by relative approximate increase in intervallic distance (1<2<3<4) | Each IC represents the melodic function of a tone in relation to another tone: a unison - anchoring, a 2^nd^ - complimenting, a 3^rd^ - opposing, a 4^th^ - extreme | Grouping by stops/breaks in the sinusoid contour, with centrifugal role of peak & trough points | Attraction amongst complimenting tones vs. repulsion amongst opposing/extremizing tones. Strong melodic inertia causes scalability | Scalable transposition of IS, depending on the registral position |
| Oligotonal  Mode | 1-4 degrees: 1-2 definite, and 1-3 variable in pitch (within the range of about a 3^rd^), with a unichordal, dichordal, or trichordal kernel | Formative role of leaps that are filled up by steps: especially zigzags and skips over a tone in the melodic contour. Tuning through collective performance | Categorization of degrees by their registral order in the manner of a scale. All unstable neighbor tones become complimentary | Fixed values for unison, 2^nd^, 3^rd^, & 4^th^ in melodic & vertical versions, realized incrementally. Equivalence of neutral 2^nd^ (step equivalence) | Anchoring and complimenting turn into “class-functions,” standardizing the ICs between different degrees through heterophony | Balanced centrality of anchor(s), weakened melodic inertia. Modal bareness exposes con-/dissonance | Attraction of tones that are variable in tuning to tones permanent in tuning, which are more frequently used & rhythmically stressed | Fixed transposition of IS across the entire ambitus |
| Mesotonal  Mode | 5-6 degrees: 1-3 definite, and 2-4 variable in pitch (within a range of about a 2^nd^), with a trichordal or tetrachordal kernel | Formative role of resolution: unstable-to-stable pairs form 3 classes: gapped disjunct (C-D, F-G), step disjunct (C-D, E-F) or step conjunct (C-D, D-E) connections | Degrees are defined by fixed auxiliary (I-II) and complimentary (I-III) functions. I-II determines horizontal, I-III - vertical harmony | Added tritone, 5^th^, & 6^th^ + their vertical versions. Equivalence of neutral 3^rd^ (over a degree relation) and 2^nd^ | Anchoring, auxiliary, & complimentary functions become “class-functions” assigned to a specific degree, determining the intervals permissible on it | Centripetal gravity, based on the “resolution paths” of 2-3 auxiliary pairs, marking 2-3 level hierarchy | Permanent stability or instability of the degrees establishes the ‘tendency tones’ and fixed modal intonations. Induction of cadences | Transposition of motifs by degree within a mode. Transposition of PS by fixed interval |
| Multitonal  Mode | 7 degrees: 3 definite &  4 flexible in pitch (within a semitone), tri-, tetra- or pentachord based. Expansion of ambitus with few octave equivalent PCs | Contrast between 3^rds^ on odd & even degrees shapes a mode by forming triadic relations (3^rd^ + 3^rd^ = 5^th^) and introducing new layers in harmonic hierarchy. Gradations in stability | Categorization of degrees by odd/even triads. Distinction between major & minor 3^rds^ & 2^nds^. Addition of degrees above & below the 7-tone kernel. | Added fixed 7^th^, octave & compound vertical intervals. Equivalence of 4^th^ (over 2 degrees) & octave (limited). Emergence of “natural chromatic” modes | Induction of wide melodic & harmonic intervals, uniformity of intervals across all complimentary degrees, formulation of IC & ISC. Codification of vertical harmony and chords | Strong centripetal gravity, with  4-level hierarchy, with common alternation of 2 “tonics”: low VI-I, I-II, I-IV, or I-V (III) | Odd degrees forming octave equivalent stability axis. Even degrees are inequivalent. Induction of the leading tone. Fine gradations in attraction of degrees. | Transposition of PS ‘by interval’ and ‘by degree’: from I to low VI degree within the mode. Metric function of mutability |
| Pentatonic  Mode | 5 fixed diatonic pitch classes, defined through the circle of 5^ths^, with full octave equivalence, on a trichord base. Limited major/minor inclinations | Asymmetry in distribution of three whole tone- and two 1 ^1^/_2_ tone-apart degrees within an octave. Formative role of conjunct vs. disjunct trichords in reference to the interval of a 5^th^. Complimentarity of 4^ths^ | I degree is determined by “quadratic” (4^th^ + 4^th^) relations. Other degrees are defined by dividing a 4^th^ into a trichord & a position in relation to the 5^th^. Full octave functionality | Exclusion of minor 2^nd^, major 7^th^, & tritone. Equivalent 4^ths^, 5^ths^, & octaves allow parallel motion. Prevalent melodic consonance, although some styles employ very wide leaps | Definition of intervals by means of octave & 4^th^/5^th^. Tendency to equalize harmonic consonance & dissonance. Horizontal harmony determines vertical. Common 4^th^–chords & their inversions | Extremely diffused gravity, 2-level hierarchy for stable degrees only. Unstable degrees are rarely used in succession. Anchors are often shifted | Weak attraction, while strong melodic inertia, going over registers with segmentation in “terraces”. Plagal & authentic melodic typology determined by stressing 4^th^ or 5^th^ | Natural “enharmonic” modulation by trichord rotation. Possible auxiliary alterations. Possible composite modes |
|  | PS | PSC | OD | IS | ISC | GS in PS | Attraction | Mod./Alt. |
| Heptatonic Mode | 7 fixed diatonic pitch classes, defined through the circle of 5^ths^, with full octave equivalence, on the pentachord base. Uniform major/minor inclinations | Asymmetry in distribution of 5 whole tone- and 2 semitone-apart degrees within an octave. Formative role of conjunct vs. disjunct tetrachords in reference to the interval of a 5^th^. Complimentarity of 3^rds^ | I degree is determined by triadic (3^rd^ + 3^rd^) relations. Other degrees are defined by dividing a 4^th^ into a tetrachord & a position in relation to the tonic pentachord. Full octave functionality | Opposition of perfect & major/minor intervals. Common parallel motion in 3^rds^, 5^ths^, 6^ths^ and octaves. Frequent melodic dissonance, leaps filled by steps | Definition of the ICS by means of octave & 4^th^/5^th^. Tendency to contrast harmonic consonance vs. dissonance. Vertical harmony often determines horizontal. Common triads & their inversions | The strongest centripetal gravity:  3-level for stable & 4-level for unstable degrees, with clear tonic. Unstable degrees can be used in streaks | Uniformity of attraction/gravity values over an octave. Opposition of plagal & authentic inclinations. Minor modes are less stable than major ones. Standard cadences | Natural “enharmonic” modulation by tetrachord rotation. Auxiliary alterations. Possible composite modes |
| Diatonic Polymodal System | 2-6 fixed PC sets with shared 7 fixed diatonic pitch classes, all pentachord based, form the MPS. Major, minor, or diminished inclinations of sister PSs | Each mode’s idiosyncrasy in distribution of tone & semitone within the conjunct pentachord-tetrachord base. Formative role of tritone. Uniform tuning of PCs across MPS | The degrees are inferred from the circle of 5^th^ & octave transposition. Tetrachord marks stability as an inversion of the pentachord, I-V functionality axis | Fixed gradations in harmonicity between ISs of sister-modes. Semantic specialty of each mode (set by genre); fixedness of degrees in their tuning | Avoidance of the diatonic tritone, & its different treatment in each of the sister-modes. Rigid rules. Harmonicity of ICs determines ICS. Prevalent monodic organization | Centripetal, uniform 2-level hierarchy of stable degrees across all modes. Any unstable degree resolves to any stable degree | Functional equalization of all degrees between all sister-modes. No pronounced hierarchic relations between the unstable degrees. Chromatic alteration | “Chromatic” modulation: within a work through alteration that emulates the leading tone (emerging keys) |
| Non-Octave Hypermode | 8-12 (up to 18) fixed degrees, including 1-4 false relations, on the tri-/tetra-/pentachord base. Major, minor, or diminished inclinations of an entire hypermode | Distribution of tone & semitone within the modal subsets determines their conjunct or disjunct linking by means of “chain principle.” Minimization of melodic dissonance | The degrees are inferred from the circle of 4^th^ or 5^th^ over the tri-, tetra-, or pentachord chains, defined to avoid melodic & harmonic tritone | Natural intervals in mid subsets vs. augmented or diminished ones, formed between marginal subsets. Sister-hypermodes poorly contrast each other | Equivalence of 4^th^ in all subsets; or 4^th^ or 5^th^ equivalence of two subsets + contrasting third subset. No intervallic inversion. The richest typology of chords. Common polyphony | Centripetal or centrifugal gravity, 2- (for unstable) 3- (for stable) level hierarchy. Each subset can have its own anchor | Weak attraction, with tension set by false relations, usually increasing in the top subsets & decreasing in the bottom subsets. Strong melodic inertia | Abstaining from modulation & alteration. “Shimmering” inflections by transposition of false relations |
| Chromatic Polymodal System | 16 pitch classes with limited octave equivalence, on the tetrachord (pentachord) base, form the MPS. Multitude of subset inclinations | Contrast between chromatic clustering (pyknon) and hemiolic gapping at the opposite sides of a stable tone. Fine gradations in chromatic shading while maintaining diatonic frame | Contrast between immutable & mutable degrees inside/between modal subsets. Diatonic frame of immutable degrees. Clash between theory and practice | Wide use of micro-intervals, more so in the mid-ambitus. Ample IS, based on harmonic 4^th^, 5^th^, & octave. Moderation of melodic & harmonic dissonance | Formative role of alteration and chromaticism (including microtonal shading). Enormously rich choice of ICSs, determined by melodic resolution of unstable tones. Alternation of 3 genera | Centripetal gravity with 2-level hierarchy, diffused between each subset’s anchors, with occasional mutability I-IV | Strong attraction between the pyknon tones vs. weak attraction in gaps, increasing at the center of the ambitus. 6 types of gravitational distribution in subsets | Transposition of PSC by degree and by interval. Rich assortment of common modulations & alterations |
| Tonality  (Tonal Key System) | 17 pitch classes with full octave equivalence, based on inversion of penta-/tetrachords. Major & minor inclinations are implemented as keys with 3 minor & major modes | Codex of complex correlated rules of coordination and subordination relations between the members of a major or minor PSC, determined in reference to “tonic.” | Hierarchic division of octave into I-V & V-I subsets; then subdivision of I-III & III-V; then I-II, II-III, III-IV, IV-V, V-VI, VI-VII & VII-I; and then chromatic subdivision, with micro-chromatic fine-tuning | Octave equivalence of vertical intervals & chords is generally “implied” in melody as well. Hierarchy of natural & chromatic ICs for harmonic & melodic intervals. Formative role of inversion | Major/minor ISCs include their natural, harmonic, & melodic inflections. Augmented & diminished intervals are used to define gravity & tension. Chromatic & micro-chromatic layers are embedded into the PCSs (D#≠ Eb) | “Heliocentric” 5-tier system: balanced centripetal gravity, with “planetary” centripetal systems in all incorporated subsets (i.e. relative or dominant keys). Recapitulation rule | Strong mono-gravity: attraction of all tones to a “solar” tone, despite any distance, while keeping all “satellite” attractions. Multitude of ‘tendency tones’. Tonal plan is set to proceed through certain keys | System of key relationships, transposable by interval. Applied “dominants.” Chromatic, enharmonic, & ‘invasive’ modulations |

**PS** = Pitch Set: a set of tones typically used to make music.

**PSC** = Pitch Set Categorization: the criteria for forming of the *pitch class sets* – specific sets of tones, which are reused in multiple compositions and can be identified by ear while listening to a musical work.

**OD** = *Organization of Degrees*: the method of conceptualizing pitch classes as the degrees within the mode.

**IS** = *Interval Set*: the intervallic distances between tones of the pitch set.

**ISC** = *Interval Set* Categorization: the criteria for forming of the *interval class sets* – specific sets of intervals, which are reused in multiple compositions and can be identified by ear while listening to a musical work.

**GS in PS** = *Gravitational Scheme in a Pitch Set*: prevailing method of uniting all tones within the PS by means of tension/relaxation.

**Attraction**: the characteristic properties of the unstable tones of a mode, which contribute to generation of tension and connection of tones.

**Mod./Alt.** = *Modulation/Alteration*: the method of handling of modifications of degrees, transitions from one mode to another, and of transposition
 of modal intonations within a particular mode.

This table presents the summary of the main features of 12 principal types of tonal organization, including Western classical tonality.

It highlights the most important changes in the way how each of the schemes of tonal organization generates tonal unity within a mode.

“Pre-mode” to “Heptatonic mode” are covered in Part-1 (Nikolsky 2015).

“Diatonic Polymodal System” to “Tonality” are covered in Part-2.

This table is a revised version of the table in the Appendix IV of the Part-1. That older version should be discarded, and this version should be used instead.

REFERENCE

Nikolsky, Aleksey. 2015. “Evolution of Tonal Organization in Music Mirrors Symbolic Representation of Perceptual Reality. Part-1: Prehistoric.” *Frontiers in Psychology* 6 (1405). doi:http://dx.doi.org/10.3389/fpsyg.2015.01405.
