# Supplementary material for: Evolution of Tonal Organization in Music Optimizes Neural Mechanisms in Symbolic Encoding of Perceptual Reality. Part-2: Ancient to Seventeenth Century
Source: Front Psychol. 2016 Mar 30;7:211. doi: 10.3389/fpsyg.2016.00211 (PMC4813086; doi:10.3389/fpsyg.2016.00211)
Supplement: Supplementary file 3 [file DataSheet1.zip › Appendices I-VIII/Appendix VII. Melodic line, geometric line and environmental topography.docx]

# Appendix-VII: Melodic line, geometric line and environmental topography in traditional Nenets culture

Creators and listeners of music commonly imagine musical structures in terms of attributes of physical objects and/or their physical motion:

- center/margin (in reference to perception of changes in pitch or music form),
- height (pitch and^[[1]](#footnote-1)^ register),
- width (harmonic, melodic intervals),
- depth (the amount of parts/voices in musical texture),
- weight (register, texture, dynamics and meter/rhythm),
- shape (melodic line, texture),
- brightness (register and timbre),
- color (register, harmony and timbre),
- density (rhythm, register, texture and timbre),
- quantity (voices/parts of texture, motifs in melody, detached articulation and/or rhythmic rests),
- size (dynamics, register and texture),
- homogeneity/fracture (texture, melody, rhythmic patterns, metric pulse, music form),
- position (texture, melody, harmony, music form),
- distribution (texture, articulation, rhythm, music form),
- direction (melody),
- energy (rhythm/meter, tempo, register, articulation and dynamics),
- force (dynamics, register, texture and articulation),
- speed (tempo, meter/rhythm and articulation),
- acceleration/deceleration (tempo and texture),
- leaping/stepping/crawling incrementation of motion (prevailing melodic intervallic patterns),
- dashing/running/walking/stretching/halting pattern of gait (tempo and rhythm),
- fluidity (melody, rhythm, articulation, register and texture),
- steadiness/unsteadiness (tempo, rhythm/meter and music form),
- inertia/friction (melody/rhythm and harmony),
- attraction (unstable tones),
- gravity (stable tones),
- trajectory (texture, melody, rhythm/meter and articulation),
- restitution (rhythm, meter and music form),
- collision (rhythm, articulation and texture).

The length of this list alone suggests that music can be regarded as a grand system of spatial abstraction, where motion is perceptually specified by objective acoustical information contained in the auditioned sounds (Clarke 2001). Music competence then should be defined as a set of reliable skills for detecting this information while conceiving or auditioning music, and relating this information to a known stock of *musical idioms*. By musical idiom I mean patterns of characteristic melodic, rhythmic, metric, harmonic, and textural organization of musical material, all of which are referenced to specific emotional states recognized by the musical convention as “musical emotions” (see Zentner 2012).

Then, music appears to be a naturally formed semiotic system designed for communication of emotional states that are most important within a given social group, composed with the principal purpose to provide emotional entrainment between individual members of a group, and mediated in the best interest of each one of them (Perlovsky 2012).^[[2]](#footnote-2)^ And the above listed attributes of objects/motion, which are analogous between physical and musical virtual spaces, lay the biological foundation for effective emotional communication. The musician encodes locomotion into musical structures, and the listener decodes them, extracting the intended motion, thereby experiencing what can be called “embodied perception of music” (Leman 2008, 160). The human body here acts as a biologically designed mediator that converts physical energy into mental representations and back via the chain of performance-audition.

If that is the case, then the above-listed spatial attributes would be found across various musical cultures – hand-in-hand with cross-cultural distribution of basic emotions: music-movement relationship would secure emotional recognition (Sievers et al. 2013) in a manner of the error-correction mechanism in musical transmission - expressive denotations of typology of movement would corroborate expressive denotations of musical idioms.

Universality of *basic emotions* (Elfenbein and Ambady 2002) could find its counterpart in universality of *basic musical emotions* (Mohn, Argstatter, and Wilker 2010) to fulfill the biological need to synchronize group motivation and uniform emotional experience through acoustically mediated emotional contagion (Koelsch and Fritz 2013). The unique entraining capacity of music gives it an edge over another salient biological trait of humans – verbal communication – in satisfying this need (Perlovsky 2014). Also, hierarchic tonal organization that distinguishes music from speech is characterized by semi-automatic processing (Bidelman & Grall 2014) and exceptional informational density of effective transmission (J. H. McDermott et al. 2010), available right from birth (Bendixen et al. 2015). Verbal speech does not share such “plug-and-play” capacity: only music routinely engages cross-modal connections between sound and vision^[[3]](#footnote-3)^ (Marks 1978, 53), observed already during infancy (Dolscheid et al. 2014), as well as cross-modal connections between sound and locomotor sensations, which are uncharacteristic for experience of prosaic speech (Dalla Bella, Białuńska, and Sowiński 2013).

Synesthetic representation of musical sounds in terms of visible objects originates from motoric experience of motion that shares with musical rhythm its entraining capacity (Trainor 2007). Intersensory perception of motion relies on the vestibular system in disambiguation of musical rhythm (Trainor et al. 2009). Neither hearing the pitch contour “in terms of moving,” nor hearing it “in terms of seeing” (including multiple pitch contours, as in case of simultaneous talking of multiple speakers) are employed in typical verbal communication. That makes music the number one instrument of choice for communication not only of emotional information, but also of encoding schemes for representation of surrounding perceptual reality. Music can instruct the listener as to “how to see” – *what* to look for while viewing visual objects – and suggest to him *how* that which he sees can potentially move. The entraining properties of music make the entire audience share the same experience of a particular manner of seeing and moving, while directing their perception towards a particular denotation by means of engaging emotional communication.

It seems that the idea of tonal organization has its ultimate source in the interaction between the perceiver and the environmental topography surrounding him. Creators of the very first forms of music must have coined the first method of integration of musical tones in a perceptual whole by employing the cognitive scheme abstracted from the surrounding reality. The representational chain could have possibly started from the identification of a certain principle of spatial organization of a person’s immediate environment – what Marc Leman calls “culturally relevant descriptor” of the geographical relationship in terms of distribution of physical energy (Leman 2008, 74) - and codifying them in spectral content of music. Listening (and imagining) to such music would then cause a reverse process of transferring a mental representation of sound into a “material form” (p. xiii ibid.) by triggering locomotive experiences and spatial images. This “embodied perception” of music is likely to translate the tonal organization of music into spatial organization, in a feedback loop. In this paper I intend to present some ethnographic evidence for the presence of such loop.

The oldest forms of tonal organization may perhaps be found in societies that still resort to the same hunter/gatherer lifestyle that was conducted by Paleolithic people: similarity in social organization suggests similarity in typology of thinking, which includes music making (Both 2009). Thus, archeological evidence confirms that art forms of modern Aboriginal population of Siberia strongly resemble the artifacts recovered from the Mesolithic settlements in Siberia, suggesting an uninterrupted cultural tradition (Frolov 1992, 147). The oldest traces of human presence in Siberia date back to the end of the Paleolithic period (Khlobystin 1998, 29). The latest ethnographic estimates date the first appearance of reindeer hunters in Taimyr Peninsula (one of the utmost Northern, hard to access territories, at the coast of the Laptev Sea, by the Mesolithic period) to about 5,000 years BC (49), making its indigenous ethnicities of Nganasan, Enets, Selkup, and Nenets representative of archaic^[[4]](#footnote-4)^ music culture (Dobzhanskaya 2016).

Archeological evidence shows that the entire region of Taimyr was part of one special cultural formation of North-East Asia, with pronounced similarities found between tundra Mesolithic cultures, such as of Taimyr and Aldan – despite them being 1000-kilometer apart – as well as between the tundra and taiga cultures (49-54), demonstrating even greater homogeneity between the later Neolithic cultures of North-East Siberia (55). This homogenous culture is termed Proto-Samoyedic and dated from the beginning of the 4^th^ millennium BC to the end of the 1^st^ millennium AD, based on the data from paleolinguistics, paleobotany and lexicology of modern Samoyedic languages – breaking into constituent cultures as a result of migration of people and emergence of separate languages by the middle of the 1^st^ millennium AD (Dobzhanskaya 2011). An important factor in this ethno-genesis must have been music that bears strong contrasts in tonal organization between various indigenous ethnicities while displaying significant commonality in the patterns of its use.

An outstanding characteristics of their musical culture, as well as of their neighboring ethnicities of Extreme North, is the prominence of the so-called “personal song” as a peculiar proto-genre of folk music (Sheikin 2002, 236–334). The reason for calling it “proto-genre” is that retaining a particular configuration of musical expressive means within a specific individual’s use (and his direct posterity) precludes the formation of a genuine genre that normally operates across the family structures over wider social groups. The typology of the “personal song” probably originates in the ancestor kinship songs and totemic song imitations of calls of sacred animals. The idea of connection to an important spirit or deity by means of calling it by its unique melody (in the manner of attracting an animal by imitating its call) could have emerged in the Proto-Samoyedic culture within the Proto-Samoyedic shamanic cult, branching into shamanic traditions of separate Samoyedic cultures during the 1^st^ millennium AD (Dobzhanskaya 2011). Personal melodies of spirits and shamans could have provided prototypes for ascribing dedicated melodies to “personal songs” of common people. Most indigenous ethnicities of Siberia, Far East, and Northern North America employ personal song as means of individual spiritual representation: typically, they reuse the same melodic formula over different lyrics or vocables that are adjusted to reflect a given real life situation, such as riding, fishing, or falling asleep.

The fact of sustaining the same motif over various verbal expressions in the context of different situations and emotional states indicates that the musical component of “personal song” works as a mental equivalent of “self” – an imaginary twin-person employed to emotionally examine the interaction between the self and the environment as though from aside (Ojamaa & Ross 2004). Singers usually see “personal songs” as auditory manifestation of the “soul” of a person, or of a personal guardian “spirit” of that person (Niemi 2002). Sometimes they use *textless* personal melodies to refer to a third person: a personal motif of a relative or a friend can be used in reference to him in his absence (singing someone else’s song at his presence is usually regarded as bad manners).

Yelena Novik draws a parallel between “personal song” and “passport” – like photos in a passport, different melodies might represent the same individual in childhood, adolescence, and old age (Novik 2004, 80). Shortly after the birth of a child, his parents usually compose a melody that captures the most salient personal traits of that child, which becomes that child’s ID until reaching adulthood, at which point he creates a new melody to represent his mature “self” – some ethnicities may also engage their parent’s song as a family memorabilia (Ojamaa 2002). In this way, a personal song often carries information about the person’s family and place of origin (Sheikin 2002, 272–287). A particular melody could be inherited, given as a gift, or traded – very much like a material possession (Zemtsovsky 1983, 10).

On the other hand, the value of a purely melodic aspect of the “personal song” cannot be entirely isolated from the song itself. The melodic structures that constitute a song are perceived in an organic unity with the text, so that a singer and his indigenous listener are not capable of comprehending the pitch aspect of a song separately from its lyrics, even upon request (Ojamaa and Ross 2011). The same applies to the circumstances of performance: i.e. riding songs are performed only while riding. The “self” of a song, while being encoded in the personalized permanent progression of pitches along with the unique timbral characteristics of the singer’s voice (those that allow to recognize him by his voice), is embodied in the materialistic changeable attributes of the melody. Such are the lyrics, the activities that accompany singing, etc., which execute the function of different cloths put on the same person.

Ties to concrete attributes and circumstances testify that the “personal melody” is perceived as a “real person” rather than an abstraction of a person, such as the protagonist of a story or a film. The protagonist of a “personal song” is always the first person, and hardly ever a third person: the singer of a personal song, be it his own song or someone else’s, as a rule speaks for himself while singing.^[[5]](#footnote-5)^ Hence, the common social ban on reproducing the song of a foreign person, and especially of a shaman – since opening the gates to unfriendly spirit might let harmful influences permeate the singer’s persona (Dobzhanskaya 2016).

Therefore, a “personal song” essentially manifests the most direct form of mental representation of the imaginary “I” placed in a certain environment. This turns a musical culture where the “personal song” constitutes the most common form of music-making into the most suitable object for investigation of the correspondence between tonal and spatial organizations. If a purely musical “self,” encoded in the subjective experience of pitches, is placed in the framework of lyrics and motions, then, such placement is perceptually equivalent to defining one’s position in a certain physical place.^[[6]](#footnote-6)^

The practice of updating a personal melody from the one assigned by one’s parents to the one that is self-made, is most likely to secure the functional connection between the prevalent schemes of tonal and spatial organizations, promoting adherence to schemes that are most effective for the lifestyle of that given individual. And since each member of such society is free to adopt a melody that most suits his placement of himself in his cultural environment, then, the musical modal schemes accepted as a convention within that ethnic culture would testify to which modes of orientation work best for majority of people. Here, the orientation skills manifest themselves in two ways: as an actual way-finding strategy in a topographic environment, and as pictorial representation (or interpretation of a depicted image) of visible reality either in the form of a drawing from life or sketching a plat.

Hereby the chain of connectivity between primordial tonal and spatial schemes of organization would consist of:

- observation of environmental landscape,
- abstraction of its most prominent features into musical attributes,
- construction of melodies using these attributes,
- assigning conventional emotional meaning to the most popular melodies,
- distribution and “averaging” of such melodies through oral reproduction in a social group,
- generalization of averaged melodies into a musical mode,
- production of new melodies based upon that mode,
- abstraction of tonal principles of this mode
- translation of tonal principles into principles of spatial organization of pictorial representation.

Perhaps we could start from its final phase – spatial organization in depiction of perceptual reality. The best choice here would be a Nenets culture, one of the Samoyedic cultures of Taimyr Peninsula, perhaps the hardest to access area in Russia. Nenets traditional music of today still observes the traits of ekmelic organization – the oldest surviving form of tonal organization (Nikolsky 2015). Nenets traditional art has retained its idiosyncrasy until the XX century, and even then stayed more resilient towards Russian cultural influences than the art of the neighboring ethnicities. This has to do with stronger adherence to the traditional lifestyle: 72% of male and 38% of female Nenets maintain their traditional forms of occupation (i.e. as opposed to 29% and 5% of Evenki, respectively), 39% of Nenets population live in chums rather than houses (vs. 21% of Evenki), and 33% of Nenets still use reindeers for transportation – this is while maintaining one of the highest ethnic concentration of all Aboriginal ethnicities of Taimyr, i.e. 69,5% pure Nenets (excluding Creole Nenets)^[[7]](#footnote-7)^ and 52% of the population living in mono-ethnic settlements (Bicheool 2012).

In a unique field experimental study, where the majority of the subjects were tundra Nenets - all the reindeer herders - were found incapable of drawing a cube from real life, regardless of their level of education (Goncharov & Tiapovkin 2012).^[[8]](#footnote-8)^ Researchers put such poor projection skills on the account of influence of topological orientation in the tundra environment. According to Goncharov’s line of research, reindeer herders are known to make no use of topographic maps that would represent a physical disposition of real life objects, and find it very difficult to retrace the same route in the opposite direction. Life in a wide open space does not contribute to the development of a vertical and horizontal coordinate system. The specificity of ecological conditions of life in tundra and occupational tasks of reindeer herders promote formation of special skills and schemes of orientation throughout childhood, characterized by highly developed topological representation while deficient projection and coordinate representations (Goncharov 2007).

The 2012 experimental study confirmed that the impact of the environmental factor is more significant than the education level and cultural preferences of the subjects: both, the adult reindeer herders and their children displayed low level of projection and coordination skills in comparison with a selected control group from the city – despite the herder’s experience of visiting cities and being familiar with the cityscapes (Goncharov & Tiapovkin 2012). Mere exposure to hierarchic organization of the cityscapes apparently does not suffice to cause re-organization of non-hierarchic methods of spatial orientation, suggesting that it is only exercising orientation skills in vitally important environment that has formative power on one’s ability to represent 3D objects on 2D plane.

The peculiar combination of great topological representation and weak projection/coordination representation in spatial organization remarkably corresponds to the typology of tonal organization in music native to these herdsmen.

1. Audio: Verya Neniang Syo, personal Nenets song, Taimyr. Formula-based structure with frequent ekmelic pitch-bending that fills up the khasmatonal leaps (see Nikolsky 2015). The mode includes 4 basic degrees (if to disregard the sliding embellishments, and estimate only rhythmically stressed tones). <http://chirb.it/dgenGN>

Difficulty in topologic and geometric projection translates into difficulty in reproducing the exact same pitch contour across different performances of the same song. To be more precise, Nenets singers simply do not set a goal of reproducing a pitch contour according to some fixed reference model. In fact, they reject the very idea of pitch error: in general, they think that once a singer knows what to express, then any way of expressing it is “right.”

Nenets do not distinguish between patterns of pitch organization and patterns of lyrics (Ojamaa & Ross 2011), and therefore do not seem to track the pitch contour per se, perceiving a musical phrase in a syncretic unity of musical intonation and words. Nevertheless, pitch contours of Nenets songs do differ from those of the spoken Nenets lyrics throughout most of their song genres – unlike their rhythm and meter that generally agree between their speech and singing (Niemi 1999). This seems to demonstrate the general opposition of musical and verbal vocalizations, which characterizes tonal organization in archaic forms of music, at a stage when language and music each diverges into its own specialized domain of communication. Making melodic contours different from verbal intonations serves to assign a unique role to music: thus, the ethnographic research indicates that it is the melodic formula rather than words that is believed to directly reach a particular spirit during the shamanic rite (Dobzhanskaya 2013).

It seems plausible to conclude that during the earliest stages of tonal organization opposition of verbal and musical intonations executed the formative function, whereas at later, emmelic, stages the verbal intonations that are important for a given genre start influencing the characteristic melodic intonations of this genre.

Evidently, pitch-processing in Nenets songs is essentially ekmelic: exercised through expandable intervals and regularization of directional changes, while observing basic metric incrementality in the form of periodic rhythmical cycling - a sinusoid wave (rather than the progression of straight vector lines of emmelic music) employed as a point of reference for the construction of melodic line. Predictability of oscillation in a wave and its smoothness in covering the entirety of the available amplitude, perhaps serve as the principal facilitating features in such manner of spatial orientation. Nenets melodies are characterized by a pronounced one-dimensionality of tonal organization: a single anchor tone attracts coordinated non-hierarchical degrees, typically marking the beginning and the end of song (Niemi 2009, 102).

In most cases of tundra Nenets songs the singer seems to follow the strategy of filling up the ambitus of his song with numerous reproductions of a single formula (sometimes with considerable variation and addition/insertion of new intonations) - all wrapped around a single point of reference, with melodic intervals slightly varying in size. Such melody moves in a way much like a tundra inhabitant who follows his course through a vast flat surface with scarce landmarks by reference to the sun and the wind that may disappear or wind can change directions.

Sled tracks present the easiest way of navigation (akin to the verse of a song), yet they are vulnerable to blizzards – so, the natives have to develop a keen sense of the wind that shapes ridges in the snow (Lewis and George 1991). Orientation by sun or wind (Fortescue 2011, 74) works similarly to weak (and possibly fluctuating) tonicity of one or two anchor points indicated by degrees more permanent in tuning, as opposed to less permanent ones. Ekmelic reliance on the *gradations in permanence* resembles tundra inhabitants reliance on weather gradations in visibility, and changeable wind.

Estimations here cannot be made in a clear-cut numerical style: Arctic inhabitants demonstrate amazing discrepancy in estimating distance, where not only different individuals widely disagree between one another, but the very same person changes his estimate from day to day – and prefers measuring the distance by rather flexible means of counting the days of his travel or by the amount of the dog-food the dogs consume (Aporta 2004). Indeterminacy is also manifested in Nenets orientation of the entrance in *chum* – their nomadic tent: they make entrance from the side opposite to the direction of a wind that is prevalent at a given location, whereas other ethnicities of Taimyr fix the entrance by the compass point, such as West or South-West (Bicheool 2013a).

In such conditions, ekmelic method of modal organization can prove to be very helpful in exercising the mind to process a set of flexible-in-pitch degrees as a modal unity, integrated by the functional relation of the degrees to a single stable anchor and alternative less stable anchor tone.

Something very similar to the method of organizing melodic motion through “extraneous,” ambitus-driven, outlook at the ekmelic degrees is described by Andrei Golovnev in regards to the Nenets method of wayfaring: envisaging the view of a particular place as though watching oneself from the sky “as a moving dot on the map”^[[9]](#footnote-9)^ – in their custom of employing such integrated projection Nenets differ from the surrounding ethnicities, such as the taiga (deep forest) Khanty people, who memorize “every hummock of his hunting ground” and walk by proceeding sequentially and linearly from “point A” to “point B,” etc. (Istomin and Dwyer 2009, 43).

The cosmological beliefs of Nenets bear similar “vertical” orientation in distinction from Khanty beliefs. Khanty differ from the surrounding ethnicities by their “horizontal” outlook on the Universe as the World River, where the Heaven is located at the river head, and the Underworld – at the river mouth (Martynova 1998, 123). The most important deity is the top Underworld god *Khyn’ Iki*, whose anthropomorphic clothed doll was retained in every household – to be redressed in case of someone’s illness. After him, the most important to consider for pleasing is the god of water, *Yink Yeurt*, omnipresent in any source of water (124) and the spirits of local landmarks, family spirits and totem animals, which comprised the rest of the Khanty pantheon (126-8). Unlike Nenets, Khanty worship Ural mountains by going on pilgrimage with their sledge idols to Urals (125). This testifies to the fixedness and directionality of Khanty spatial thinking.

Nenets beliefs, on the other hand, reserve an important place for the cults of Sun (*Heyha*) as a force of life, and Moon – as a force of death – cults that initially must have occupied the top rank at the pantheon, but throughout the course of the 20^th^ century they were subordinated to the god of hunting, *Vaesoko* (Lar 2008). The more recent Nenets mythology still features a sophisticated cosmogony. It divides universe into three worlds, where the Upper World is considered the “Earth” (with its own rivers, mountains, stars and sun) for its top deity, Num, who subdivided it in 7 heavens, appointing each of his sons to govern 6 lower ones, placed himself and his wife, Ya’Minia, the Goddess of destiny and children, at the highest 9^th^ heaven, and reserved the 7^th^ Heaven for Moon and stars, and the 8^th^ Heaven for Sun (Lar 1998, Chapter 2). Equally powerful, Num’s brother, Nga, rules the Underworld, and every human can choose whether to comply to Num’s or Nga’s rule. Shamans can reach only the 5^th^ Heaven (and Underworld) with the help of the spirits that normally live in clouds and lower Heavens. If for some reason, a shaman is not available, humans could appeal to a spirit or lower deity by themselves through their home idol, *siadei* and *khekhe*, which therefore had to be fed, put to sleep and entertained by conversation.

Yet another means of reaching spirits for Nenets, like Evenks and Nganasans who are ethnically close to them, was sacred reindeers that symbolized the connection of wood and stone with Earth, light - with Sun, blizzard – with Water, etc. (Gratchyova 1983, 33), embodying the elemental cosmological aspects. Overall, Nenets respected many more deities than Khanty, paying tribute in sacrifices to the spirits of fire, water, patrons of various animals and places, and family spirits – the entire life of a Nenets consisted of responding to great many “signs” in his surroundings by appealing to dozens of deities and spirits in order to maintain harmony with nature (Lar 2008). The sheer amount and importance of superstitions in the everyday life, tell Nenets culture apart from the neighboring ethnicities. In the 20^th^ century, the celestial objects still exercised the most socializing influence in Nenets culture. The annual festival of the Sunrise made the central event in Nenets calendar for all clans to meet and join in a rite and a feast during which the most important events of the coming year were to be planned, i.e. deciding on the routes for the herds (Lehtisalo 1998, 14).

It seems that, overall, the Nenets culture followed a substantially more “vertical” worldview, with developed hierarchy of skies, earth and underworld, rather than “horizontal” river paradigm by the Khanty. Although Khanty also distinguished between Heavens, Earth and Underworld, their beliefs from the times of migration of their Finno-Ugric ancestors to Western Siberia were based on a unique principle of cultural dichotomy, reflected in the division in two phratries (of Por and Mos’), each specializing in sacrifices with raw versus cooked meat, gender-based division of house/tent, left/right association with evil/good, and placement of good spirits at South and East, whereas evil spirits – at North and West (Veres 2014). As it follows, Nenets’ greater dependence on the sun and the moon in navigation in tundra put in place “celestial” style mapping, and gave greater importance to metaphysical cosmological powers, invisible to the eye, as opposed to more “materialistic” religious views of the Khanty, distinguished by their binary opposition of more tangible entities. And here ekmelic organization of Nenets traditional music opposes emmelic organization of Khanty music that employs “materialistic” style of way-finding from “point A-to-point B”.

Similar counter-opposition is trackable in the domain of visual arts. Remarkably, Nenets traditional visual art until very recently did not feature realistic depictions on a plane surface (Ivanov 1954, 58).^[[10]](#footnote-10)^ Nenets’ plats demonstrate failure to grasp the notion of scaling (766). Initiated by ethnographers attempts of Nenets subjects to draw from nature during the first half of the XX century revealed a style similar to Paleolithic petroglyphs: realistic zoomorphic and schematic human features without any grouping of objects or reference to a mutual ground line (784). Even paintings of the first Nenets artist, Tyko Vylko, who was trained by Russian painters in 1907-12, strike one as flat, devoid of spatial modeling and shading (Rafaenko 1972). The only form of depiction found in majority of Nenets tribes is strictly ornamental, reserved to the design on what they wear or carry: where few types of ornaments schematically represent real-life objects (Mitlianskaya 1983, 206).

1. Female Nenets kapor (hat). The ornament is known amongst Nenets as the “reindeer’s horns.” It can be found on garments and bags, but not footwear – out of respect for the animal that is principal for Nenets’ survival. <http://bit.ly/1gYNhdG>

Just as pitch contour of Nenets song contrasts Nenets speech contour, shapes of their art bear little resemblance to real objects. Flatness of tonal subordination to a single anchor-tone corresponds to deadpan flatness in projective drawings. Inability to sustain a single increment in map scaling corresponds to “changeable in size” ekmelic intervals, where pitch-gliding works as in a blind person trying to represent the disposition of objects around him by touching them consecutively, one after another.^[[11]](#footnote-11)^ Permanence of rhythm and meter in tundra Nenets song resembles the ornamentation principle that is observed amongst other tundra ethnicities: an ornament consists of the element/pattern that is reproduced over equal distances in vertical and horizontal dimensions – usually in a single direction, forming a band – often vertically symmetric (A. G. Petrova 2014).

1. 11 samples of Nenets ornaments, known as “man’s head,” “woman’s head,” “calf’s horns,” “hare’s ears,” “reindeer’s path,” etc. <http://bit.ly/1U0rF2n>

Ability to conserve intervallic distances appears to be limited primarily to metric organization of music rather than pitch, indicating greater capacity for the incrementation of time than space. Just as music of indigenous population of Siberia features many common traits in rhythmic organization, the ornaments found in traditional art of different ethnicities display great commonality of design. It appears that different ethnic musical styles contrast each other more so in tonal than rhythmic organization.

On the other hand, realistic images occupy a significant place in the visual art of Khanty people, boasting an impressive lineage that dates back to 2,000 years BC, with 54 sites of rock art located in Ural mountains – many images of which are still found in use of folk art by the modern Khanty (Shirokov and Tchairkin 2011). Some compositions feature quite sophisticated spatial organization with noticeable canonic rules: i.e. most petroglyphs face South, animals are depicted in profile, but people are depicted in “full face”, and the landscape elements are included in the composition, forming the background layer – following pronounced centripetal organization in relation to the principal subject of the composition: the objects surrounding it might be depicted upside down (Tchernetsov 1971, 24–27).

1. Fragment of petroglyphs from the Vishera stone, Kama region, Ural, Bronze Age. The entire rock contains 213 images representing reindeers, bears, anthropomorphic creatures, masks, and geometric symbols, all facing South – next to the sacrificial place (Shirokov and Tchairkin 2011, 28). <http://bit.ly/1KozXHG>

Depiction of real life objects is encountered in almost every Khanty household: animal figures are carved on boats, cradles, needle-holders, mortars, and tattooed pictures are common although they were more common in the past (Lukina 1985). Images play an important role in religious beliefs, such as healing rituals that require making a “voodoo” doll, figurines and pictures of anthropomorphic and zoomorphic idols are recovered at the ruins of sacrificial sites, and images and figures of personal patrons are routinely manufactured from wood, bark, textile scraps, or stone, by male and female Khanty (Martynova 1998). Furthermore, the aesthetic appreciation occupies greater role in Khanty art as compared to the Nenets. Decoration of an artifact is no less important for the Khanty than its manufacturing, often exceeding the latter in expense and production time: such items as a bag or a coat, are considered ready for use only after their decoration is completed (Siazi 1995). Attention to decoration transpires into the complexity and innovation of form, prominent in the composition of ornamental designs in Khanty applied arts.

Khanty music also demonstrates greater concision of details, attention to music form, permanence in tuning, and concern for diversity of tonal schemes. Most of the compositions are based on octave-equivalent pentatonic modes, but heptatonic and hexatonic music is also found. In contrast to the overwhelmingly vocal monophonic Nenets music, the Khanty musical texture is more complex: featuring either monodic or heterophonic design due to the use of instrumental accompaniment (Bogdanov 1982). Khanty manufacture and play on the rich assortment of percussive, wind, and string instruments, including 2-string violin, 12-string harp, and 5-string zither. The popularity of instrumental music is evident from the wide spread of instrumental personal pieces used along with personal songs to musically represent an individual. The melodies are characterized by clear patterning, contrast, featuring salient thematic material, and reliance on “square” metric structures (featuring even and regular distribution of metric accents) with little importance given to variation (Soldatova 2012). Music form is usually of a composite type, created by repeated alternation of two or three motifs (A-B, A-B-C) (Lazar 1997).

1. Audio: Sortyng pusl pohyry, Khanty song. Octave equivalent pentatonic mode, alternation of two motifs. <http://chirb.it/G213p9>

Clearly, Khanty visual art and music contain more definite, proportional, and complex structures that involve subordination and coordination of permanently shaped elements by means of fixed intervallic values. This organization closely corresponds to Golovnev’s description of Khanty wayfaring strategy. Much of Khanty preference for a “composite” approach to musical and pictorial compositions, as well as to topographic mapping, must have to do with the mixed terrain of mountains, valleys, rivers, and forest in which they live. Their more sedentary life style, promoted by fishing and hunting, where they constantly return to the same place of residence, could also have contributed to the emergence of fixed intervals, permanent emmelic pitch set, and music forms based on refrain and recapitulation, with little place left for variation and improvisation.

A very different situation with tundra Nenets art also has explanation in their geographic environment and tasks typical for a nomadic lifestyle.^[[12]](#footnote-12)^ It could be that ekmelic organization is representative of nomadic life style living on plane surface, and Khanty ancestors had ekmelic music by the time when they exercised nomadic lifestyle in the South Uralic steppes. According to most recent estimations, Nenets culture appears to be substantially younger than Khanty culture: the Nenets ethnicity was probably formed around the 2^nd^ half of the 1^st^ millennium AD in the taiga zone between the two rivers of Ob’ and Taz, and obtained its cultural identity through migration to the tundra area, and commitment to reindeer herding (Labanauskas 1992, 2:1–3). The Nenets assimilation with local Aborigines tundra people, *sikhirtia*, dedicated hunters and fishermen, most likely took place during the 15-17^th^ centuries, generating two principal Nenets phratries, *Khariuchi* and *Vanuito* (V. P. Petrova and Khariuchi 1999, 9). Khanty phratries of Por and Mos’ were most likely formed some time between the 12^th^ and 10^th^ centuries BC (Veres 2014), and Khanty ethnicity took its modern shape at around the 9^th^-13^th^ centuries AD, according to bone measurements at archeological sites in Tiumen region (Bagashev and Poshekhonova 2008). If Khanty ancestors were ever employing the ekmelic organization in their music, they must have accomplished the transition to emmelic music some time during Middle Ages.

The orientation principles cultivated by the Khanty tonal organization are of no use for life in a snowy flat surface with scarce landmarks. In contrary, the principles of integrating multiple variables in an ongoing reproduction of the same cycle of migrating with a herd from one area to another, never following the same route, constantly depending on track-breaking skills in finding virgin land rich of vegetation, are of the highest value in Nenets life. And it is exactly these skills that are promoted by the ekmelic organization of their music. Tim Ingold points out that way-finding shares with music an essentially *temporal* character: “the path, like the musical melody, unfolds over time rather than across space” (Ingold 2000, 238). In remarkably similar way, the Nenets and Nganasan shamanic rites ensure uninterrupted flow of a melodic line as means of reaching a necessary spirit of deity – a group of shaman associates have to help the shaman out to avoid any silence in carrying out the melody that is made of variations on the personal melodic formula of a spirit – resembling the continuity of moving by the road to a magic target, contingent upon not ever stopping (Gratchyova 1983, 56).^[[13]](#footnote-13)^ Amidst the snowy tundra, stopping would imply risk of freezing to death.

It is very likely that music serves to encode and reinforce a mentality that is so crucial for survival in harsh climate conditions. That could be yet another explanation for such prominenceof personal songs amongst the Arctic ethnicities - they might have originated from the tundra inhabitants, later spreading to the forest and mountain areas. Krushanov describes an example of a Chukchi hunter, caught by the storm for few days, who was trying to keep himself warm so that he would not freeze to death by singing a new melody, and how was found by his sons, who heard his melody and made it their “personal song” after his death (Krushanov 1987, 234). Much time spent all by oneself in such critical climate conditions would promote a culture of singing-to-oneself.

Noteworthy, there is a difference in style between songs of tundra and forest Nenets (Jones-Bamman 2009), which is attributable to the differences in geographic landscape (Brodsky 1976). Yuri Sheikin (Sheikin 2008, 44) holds that there are more differences than similarities between the musical features of taiga and tundra Nenets songs that are determined by the great contrast in their living environment. Thus, the tundra songs tend to feature undulating-horizontal melodic contour, whereas the forest songs follow the apex-nadir model (Niemi 1999). Overall the forest songs feature greater finesse of the melodic detail, greater freedom of variation, often employing what Niemi calls “additive pitch organization” (Niemi 2009, 98) – insertion of small melodic motifs based on intonations that are different from those of the melodic formula - and even contain open-end improvisatory development in metrically flexible elaboration. Such improvisatory personal songs strongly contrast the strict formulaic style of tundra songs.

Geographic differences seem to contribute to different methods of orientation and construction of mental maps adopted by people that otherwise share the same lifestyle. Thus, Komi reindeer herders follow well established migration routes that have remained unchanged over centuries, travelling in two parallel brigades 5-10 km apart in linear fashion for 400-500 km north over the terrain that includes rivers, streams, lakes, ponds, bogs, thermokarsts, hills, hill ranges, forest and areas with specific vegetation, such as dwarf trees – which are used for navigation (Dwyer and Istomin 2009). Taz (tundra) Nenets reindeer herders move within the plot of about 60 square km in a circular fashion, without any established routes, changing the plots every 5 years, shifting by up to few hundred km away, using such landmarks as rivers, bogs, forested patches, lakes, hills, ponds and areas with specific soil type (Istomin & Dwyer 2009).

The interviews of the herders reveal the presence of drastically different cultures of orientation employed by Komi and Nenets people. Komi herders could describe the vicinities in detail up to 15 km aside their linear route, in reference to various objects, whether these objects were present in the immediate environment or far away – as long as the distance was along their route. Nenets herders could describe the area up to 30 km in radius, but only in relation to the objects observable in the surroundings – as soon as an object would go out of sight, a Nenets could not show direction to that object any more.^[[14]](#footnote-14)^ Nenets navigation strategy consisted in breaking the terrain in patches delineated by the closest watersheds, and then examining and remembering landmarks in the territory enclosed within that patch.^[[15]](#footnote-15)^ Thinking in terms of plots of land occupies the principle place in Nenets ability to find their way – without which they appear to loose most of this ability. The strategy of defining the hydro-system first probably has to do with what Golovnev referred to as “celestial” perspective that distinguishes Nenets culture from those of the surrounding people: a skill to organize space from the birds’ point of view, from very high in the sky (Istomin and Dwyer, 2009, 40). The origin of this cultural trait must be the interplay of 4 factors:

- Vast openness of horizon line in all directions in Nenets’ native tundra area;
- Shortage of landmarks in their native environment;
- A special importance of rivers and streams in their nomadic lifestyle;
- Religious beliefs that give special importance to the gods of sun, moon and water.

The findings of Istomin and Dwyer demonstrate that people who belong to two different ethnic cultures follow completely different methods of mapping the surrounding space despite each sharing the same occupation and operating on more or less the same terrain.

- Komi pay attention to vegetation, whereas Nenets – to the soil;
- Komi map in linear fashion, whereas Nenets – in circular;
- Komi can rotate landmarks in an abstract way (without seeing them), whereas Nenets cannot;
- Komi map sequentially, while Nenets – by first defining an area, and then examining its content.

It is enticing to explain these differences by the kind of music that each of these ethnicities uses. Nenets music follows ekmelic organization, characterized by circular melodic motion formed by repetition of the same formula, with one fixed tone and other 2 or 3 tones floating in pitch, defined in relation to the anchor and to the position of the beginning and end points of a phrase on the melodic curve. This practically means that a music user defines the entire ambitus by the amplitude of a sinusoid curve, breaks the curve into segments by marking the start/end points of a formula in reference to the peak and trough, and then examines the content of each of the segments – essentially, the same strategy as Nenets herders employ in spatial orientation. Their inability to abstract projections corresponds to their music that makes no use of permanently fixed pentatonic and heptatonic interval set classes. Their degrees need a uniform curve and a permanent anchor. Without actually singing a formula, ekmelic singer cannot define the pitches of a “mode” – unlike a singer of the emmelic mode, who possesses pre-compositional knowledge of the sound of a particular mode. Orientation by the register plays a greater role in ekmelic singing than orientation by the intervallic distance between the tones of a melody (Nikolsky 2015). A performer maps a tone in relation to the particular registral range, as to its top, middle or bottom – where the exact intervallic distances are defined by the rhythmo-metric and syllabic properties of the tones in a wavelike musical formula. Such orientation is remarkably similar to breaking the terrain into plots: a singer breaks the entire ambitus of his voice into registers and selects a register for a specific song, after which the tones of that song are defined in relation to the margins of a register.

Komi musician, on the other hand, is accustomed to the heptatonic organization. Few of the oldest Komi songs utilize oligotonal and mesotonal hemitonic modes, whereas most of their vocal and instrumental music is based on Aeolian minor or Ionian major modes, with common mutability between the I-VI degrees (Chistalev 1977). That means that Komi singers utilize their knowledge of how a particular mode “goes” prior to making a song. The melodic style of Komi music is marked with pronounced Russian influence, featuring advanced heterophonic and polyphonic textures, with common 3-part singing (Chistalev 1976). Komi instrumental music is very advanced, and includes 36 instruments, such as different types of zither (up to 6 strings), lutes (up to 4 strings), balalaikas, harmonica, variety of flutes, double-pipes, pan-flutes, ocarina, clarinet, oboe, horn and different types of accordion – many of which are engaged in the polyphonic ensemble performance (Chistalev 1984).

1. Audio: Komi mu, song about the motherland, Komi. This song displays prominent influence of Russian folk music, and features Aeolian C minor key. <http://chirb.it/K9gzC0>

Komi music is created following pre-compositional knowledge of the IS of a specific key. Music making here consists of on-going inference of intervallic values for the distances between parts in a polyphonic texture and between adjacent tones in a melodic contour. Intense melodic variation in polyphonic textures cultivates skills of attuning one’s part to the neighboring part while elaborating the melodic and rhythmic detail in rendering the repetitions of a verse and, to a less extent, chorus. The span of a verse or chorus is considerably longer than typical Nenets melodic formula. Subsequently, it is not difficult for the Komi people to mentally visualize the route all the way from the point of departure to the point of arrival in a manner of linear succession of specific landmarks. Neither it is difficult for them to maintain parallel direction between the routes of two brigades of a herd. They can navigate in a familiar melody both, between the tones in vertical (multiple parts) and in horizontal (melodic phrases) dimensions – pretty much like they can define the position of any known landmark in relation to where they stand. In both cases, they engage memory of the intervallic distances between the adjacent points.

It could be that the Nenets people forged their ekmelic method of tonal organization while living in the tundra conditions, therefore their music absorbed the cognitive schemes needed for orientation in terrains with scarce landmarks. Once such music was adopted as representing Nenets cultural identity, it started exercising formative influence on orientation strategies of the entire Nenets population, whether they lived in tundra or taiga conditions – as long as they grew listening to the ekmelic music. Then, herding in terrains rich in landmarks would have little impact on Nenets herders’ strategy of way finding – they would mentally represent the space with diverse landmarks in the same way how they would represent the space devoid of such landmarks. Ekmelic habituation could be responsible for the inability to map sequentially in a linear fashion and to define position of a landmark that is not immediately visible, disclosed by Istomin & Dwyer (2009).

Different styles of mental mapping, in turn, are shown to affect the representation of 3D space in pictorial images of tundra Taz Nenets as opposed to Chukchi and Yup’ik Eskimos of mountainous Chukotka (Istomin, Panáková & Heady 2014). Thus, Nenets children were found to include fewer contextual information in their drawings to make pictorial objects larger in size and to commit to the strategy of at first drawing the background, and then the foreground objects – in contrast to the Chukchi and Eskimos children. The authors of the study put these differences on the account of the difference in the lifestyle and the appearance of the native landscapes. Nenets population is nomadic, spending most of the year migrating through the tundra, living in temporary camps without any differentiation of social roles (apart from those based on gender), and without a formal authority figure. On the other hand, Chukchi and Eskimos of Chukotka mostly have a sedentary lifestyle, are organized in kins that governed by the elders, and observe division of roles, particularly prominent amongst hunters. In regards to landscapes, Providensky and Chukostky districts contain a mosaic of seashore, coastal marshes, basins, and mountain ridges of various sizes, areas densely overgrown with mid-size bushes and areas of mountain tundra covered by grasses and sedges where mountains are most salient, often closing the line of the horizon. In contrary, Tazovsky district (the home of Taz Nenets) contains nothing but flatland tundra, without any elevations higher than 10 meters and very few trees, opening vast vistas in all directions, and making the horizon line always visible. More diverse scenery of Chukotka could be responsible for greater attention to detail in Chukchi and Eskimos drawings, as well as their foreground strategy – since mountains frequently block the horizon line – in contrast to the Taz tundra landscape. Presence of social hierarchy is also likely to promote greater differentiation between pictorial objects and focusing on the foreground figures.

However, the tonal organization of traditional music could be yet another powerful factor in the choice of pictorial strategies. Chukchi music is overall much closer to Nenets music than to Komi or Khanty musics: like Nenets, Chukchi lack instrumental music,^[[16]](#footnote-16)^ widely cultivate personal song and shamanic music, and employ throat singing in a number of genres. Nevertheless Chukchi music engages more complex form and content as compared to the Nenets music. The *pil’g’ein’en* songs often sonically illustrate running of a reindeer herd scared by a wolf, riding a reindeer sleigh, migration of birds, spring blooming, different types of human labor, various moods, etc. – that are performed in regional song contests (Krushanov 1987, 234), which is likely to promote originality and aesthetic aspect in the art of musical composition. Chukchi personal songs also often incorporate “program music,” such as imitation of the contour of the hills through the pitch contour of a melody (ibid).

The oldest Chukchi songs are based on a 4-degree anhemitonic mode that most likely came as the development of the earlier ekmelic organization. Many songs feature pentatonic organization of up to 6 degrees.

1. Audio: Keyukei, Chukotka. This Chukchi song about the reindeer calf features pentatonic organization in a 6-degree mode, based on two 4^ths^ (G#-C# and D#-G#), with frequent melodic leaps and a rather complex music form (based on multiple formulas). <http://chirb.it/LEd56G>

Greater complexity of the musical form and illustrative tendency in the musical content could account for greater attention to detail in Chukchi’s drawings as compared to Nenets drawings, reported by Istomin, Panáková & Heady. And the very presence of anhemitonic intervallic type testifies to the existence of the emmelic PCSs in Chukchi music. Singing a melody in a known emmelic mode necessarily involves reference to the tonal anchor that is intervallicly related to the other PCs of a mode – remarkably similar to the hierarchic representation of pictorial objects and compositional preference for the foreground objects in Chukchi drawings. When the singer performs a tone of a melody that utilizes an anhemitonic mode, that singer brings this tone to the foreground of a known melodic contour, and maps it in relation to the surrounding tones under the condition of avoiding an interval of a semitone. Such point-by-point processing of a melody is an equivalent of point-by-point processing of a vista while depicting it, which explains the Krushanov’s example of a Chukchi song that sonically depicted the hills of the singer’s neighborhood. Nenets strategy of drawing the background first, on the other hand, corresponds to the register-based rather than interval-based processing of melody: Nenets at first define a pitch range, and only then can infer the position of a specific tone in that range – very similar to Istomin and Dwyer’s description of Nenets way-finding strategy of dividing the terrain into plots by the watersheds.

Yet another cognitive by-product of ekmelic register-based orientation could be the prevalence of the bird's-eye view in the drawings of Nenets children as opposed to the more horizontal projections in the drawings of Chukchi and Eskimo: Nenets representation of the same scenery typically presented a more distant view from greater height (Istomin, Panáková, and Heady 2014, 90). Such tendency must reflect the same cognitive component that determined the “celestial” mapping strategy, described by Golovnev, which is likely to have originated in the strategy of defining the register for a song prior to reproducing its familiar melodic contour. Operation of delimiting the register is quite similar to envisaging an enclosed space for a picture, which involves positioning the spectator “outside” of that space in order to effectively encompass it, and to elevate the view point in order to cover more area in all the depicted dimensions, including the dimension of depth.

Emmelic tonal organization does not introduce such a bias, because it proceeds by intervallic relations between the adjacent tones, fixed through the matrix of a known musical mode. Therefore, an “emmelic” drawer is likely to resort to the strategy of transferring the visual contour into the pictorial contour, point-by-point, starting from the most proximal figure. The musical equivalent to this would be conceptualizing the tune as an extension of the opening melodic interval of that tune – a very common strategy of remembering melodies (Smith 1997). Such task does not require mental distancing from a tone that is currently processed. The entire melody can be easily represented as a succession of pairs of tones by means of melodic intervals. Hence, there is no need to “step further away” in order to grasp the register, which is relatively unimportant in emmelic tonal organization, especially in Western classical music.

“Point-by-point thinking” enables drawing of a pictorial contour, which becomes the preferential strategy for drawing from observation by untrained Westerners who draw. The transition from the alternative strategy of drawing what is generally known about an object of depiction, to the strategy of matching the visual contour of a real visible object to the pictorial contour, is most evident in the order of acquisition of drawing skills by children: after they learn to draw contours at about the age of 5, they discover “intellectual realism” around the age of 7, and build their skills of contour transfer by 12 (Winner 2007). In drawing from nature they start from the most salient object, closest by its spatial relation to 0 in projection of the dimension of depth (Rosser et al. 1985). Then, they resort to the order of constructing a picture, which is learnt for a particular type of object – they have to know “how” to draw a person or a car, etc. (1978). If they do not know a particular graphic-motor schemata for the object of depiction, they often copy another drawing rather than the object itself (Wilson and Litgvoet 1994). This methodology is essentially the same as an emmelic singer singing. The singer also needs to know the mode in which the target tune goes: in which case he renders the known model in a point-by-point manner by fitting the melodic contour in the pitch “slots” of a particular PS, or, if he does not know the mode, he is likely to start copying the singing of a singer who does know the right mode – again, point-by-point.

All in all, drawing from nature is likely to be present in those cultures that utilize emmelic singing, since both are based on point-by-point rendition of contours. And the opposite of this is also true: a culture where ekmelic singing serves as the principal form of music making is likely to cultivate strictly ornamental form of pictorial art – exactly what we observe in case of the Nenets traditional art. Ekmelic organization requires monophonic texture and is too technically difficult for instrumental production, thereby excluding the possibility for a musician to visualize the intervallic distances on a fretted string instrument or between the holes of a pipe. Subsequently, ekmelic artist would find it difficult to represent the depth dimension in his drawings and to observe “intervallic” proportionality between the dimensions of the pictorial images.^[[17]](#footnote-17)^

Traditional Chukchi art manifests compositional features indicative of the emmelic organization from very early on. Even the ornamental design in traditional Chukchi applied art exhibits “intervallic” rendition, which often symbolizes numerous objects of the perceptual reality, joined in an ensemble: Chukchi embroidery often includes ornamental representation of the sun, sunrays, stars, birds, or the moon (Bogoraz 1991, 162). The roots of Chukchi graphic art go back to 1,000 BC – the Pegtymel petroglyphs discovered by Samorukov in 1967, and first described by Nikolai Dikov (Dikov 1971). They demonstrate sophisticated technology and spatial organization, including ensemble scenery, where the figure of a frontal animal masks the animals “behind” it – all images being carved by quartz and metal tools (Devlet and Girya 2011). Such masking indicates the ability to conceive vertical harmonic intervals, since presence of two simultaneously sounding parts, separated by an interval of a fixed size, is perceptually equivalent of one image being blocked by another image.

1. The reindeers, Pegtymel, Chukotka. In this rock carving we see the presence of spatial composition, where two reindeers in the foreground are bigger in size than the smaller reindeers in the distance. <http://megalithica.ru/assets/gallery/191/rezcrop/r800_1986.jpg>
2. A boat with a crew, Pegtymel. Here we have a clear case of an ensemble of figures integrated in a single pictorial image. Such visual composition corresponds to emmelic music composition. <http://photos.wikimapia.org/p/00/04/44/91/48_big.jpg>

The Pegtymel petroglyphs reveal similarity in style with the modern Chukchi bone carving art (Ivanova-Unarova 2005, 139). Chukchi metal, wood, and bone artifacts of the 18-19^th^ centuries found their place in numerous Russian museums, especially realistic animal figurines cut from bones, decorated with ornaments which carried magical meaning (Krushanov 1987, 241). Etchings on sea-lion tusks often represent animal and human figures, and even scenes of hunting or recreational activities (141). Reproduction of a hunting scene from the beams of a Chukchi dugout was captured on paper by the Russian traveller, Voronin, in the 18^th^ century (Vdovin 1965, 41). Drawing of animals on wooden plates and oars is still a common rite amongst modern day Chukchi hunters.

The manner of spatial representation in a drawing is likely to be determined by the manner of connecting musical tones in a native music, which in turn is likely to originate in the manner of spatial orientation in the native environment. And all of them can be charged by a certain emotional denotation by means of public convention. People are known to form mental images of those places which demonstrate high communality (Gould and White 1986, 27–32). Importantly, the most common attributes of the earliest form of aesthetics must have been what Stephen Davies tags as “landscape aesthetics”: appreciation of the typical scenic design consisting of water, sky, vegetation, animals, and people – all set to exemplify the ideal habitat – apparently, so powerful in its positive impression that it still maintains its grip over majority of world’s population even in industrial societies (Davies 2012, 87). Clear aesthetic character of overwhelming “liking” of this landscape content might testify to the predisposition to learn and remember as a reference-frame those landscape environments that are native to us and are associated with abundance of life resources. Hence, geographic environment receives an edge in determining our aesthetic preferences, providing a channel for the environmental topology to affect our preferable mode of orientation – which can be abstracted into the scheme of orientation in tonal organization of music.

It seems plausible to accept as a general tendency that the more diverse are the visual forms of the natural habitat, the more detailed the primordial tonal organization in cultures native to that region is likely to be (provided no significant cultural influences are imposed from foreign cultures). Thus, the archipelago of Tonga in Polynesia remained relatively free from external cultural influences until late 19^th^ century. Traditional Tongan chant is famous for its unique sophistication, routinely employing up to 6-part textures of functional polyphony (Kaeppler 1990, 195). Tongan traditional art seems to provide a close match with its complexity of pictorial composition. Tongan barkcloth is assembled from multiple materials contrasting in texture and ornamentation (Lythberg 2013). The projection and coordination representational skills of Tongans are also relatively high: Tongans can draw accurate hierarchically organized maps – representing diverse topology of their island’s landscape (Bennardo 2002).

1. Ngatu, Tongan barkcloth, British Museum, article 2013,2015.1. One of the most popular in Tonga forms of art is ngatu that involves imprinting, painting, and gluing together multiple natural materials applied onto the bark of the mulberry. It presents a complex combination of multiple schematic and pictorial images designed to express a conventional metaphor (Kaeppler 2008, 102–4). <http://bit.ly/1ldDsek>
2. Audio: Lakalaka, Navutoka village celebrating coronation, Tonga. Traditional Tongan chant has survived in Royal ceremonies. It uses polyphonic and homophonic multipart a capella singing that requires high coordination and subordination skills from the entire village population. <https://www.youtube.com/watch?v=GLtiLbi8J_8>

It is important to underline that representational models cultivated by spatial and tonal organizations are not mere *conventions* randomly adopted within a community of users – they are characteristic forms of *adaptation* of human perceptual system to such reflection of reality that is accurate enough for success in social goals, typical for a given culture (Kubovy 1988, 109). The connection between frequency of sound and visual dimension of height appears to be inborn (Walker 2004) – there is experimental evidence that congenitally blind people engage in aural-to-tactile transfer exactly like sighted people do in aural-to-visual (Walker 1985). Segregation of auditory information into distinct auditory streams is shown to operate by the same principles as the segregation of spatial information (Schadwinkel & Gutschalk 2010). Throughout childhood one constructs specific schemes of categorization of pitch as well as methods of its spatial representation – prompted by his cultural environment.^[[18]](#footnote-18)^

The reason why Nenets neither fix intervals in their music nor fix intervals in their drawings is not because they happened to like to express themselves in this way, but because their natural capacities to hear spectral organization of sounds and interpret visual depth cues were developed to satisfy their daily needs in the tundra environment.^[[19]](#footnote-19)^ As long as they stayed in this environment, their mind stuck to the "ekmelic" manner of representation, overriding personal differences in style of seeing and hearing. This manner is only *partially* a convention – it cannot be arbitrarily reformed by a composer or a performer. It has to be effective in promoting that which people need on an everyday basis. It can change only when people's needs change. Until then, Nenets children who are raised in their natural environment are going to keep learning this standard from the adults in their community. But if these children move from the tundra to the city, their needs will radically change – they abandon Nenets music and learn Western tonality (Abramovich-Gomon 1999), because tonality is the method of thinking that corresponds to the urban way of life in a technologically driven consumer society.

Thus, children raised in a Western urban culture are shown to perceive pitch and rhythm in mathematical terms (“how much,” “how many,” and “what ratio”) at the age of 11 years (Bamberger & DiSessa 2003). This tendency could very well be the result of learning the hierarchical organization in tonality: the rankings in stability and instability provide easy demonstration of the conversion of quality into quantity, and the development of harmonic skills which usually becomes established around the age of 12, based on the acquisition of the ability to hear the diatonic frame of a tonal key at the age of 6-7 (Hargreaves 1986, 92). The way children are taught and learn music is shown to determine the development and a particular structure of their mental representations (Gruhn 2004).

Tonality requires skills of subordination and coordination, equal incrementing, scaling, and tracking of an object in relation to multiple axes in space and time. This set of features distinguishes tonality from modality (see Nikolsky 2015), as much as from atonality: attribute of spatial organization is evident only in music composed during the so-called common practice period, “spatial articulation” notoriously recedes in avant-garde music composed after 1945 (V. McDermott 1972).

Different musical cultures feature different schemes of spatial representation. English musicians represent pitch/time as vertical/horizontal axes; Japanese musicians raised on Western music share the same representation, while Japanese musicians raised on Japanese traditional music encode pitch/time as horizontal/vertical axes; and Papua musicians are not receptive to any of these axes at all, instead, they categorize melody in terms of hue and loudness (Athanasopoulos & Moran 2013). Another study found similar discrepancy in visualization of pitches between educated Cairo citizens and inhabitants of Bedouin village in Egypt (Sadek 1987). Yet another confirmation comes from comparative analysis of pitch perception in urban Canadian, Indian, and Intuit children: Western culture promotes estimation of tones in pitch (as opposed to other auditory properties) and the representation of pitch in terms of vertical height (Walker 1987) - non-trained children seem to hear the changes in frequency, but are not capable of mapping them incrementally to a vertical axis, although they favor it as a visual analog to frequency. Similar changes in pictorial strategies and schemata are reported in Australian Aborigines school children after their exposure to Western cultural influences (Cox 1998).

Ethnographers report that the “mediascape” – virtual space constructed by means of computerized modern technology and distributed in the form of films, images and videogames – to be the prime offender of the traditional culture of spatial representation, when the configuration of “virtually real” objects throws off the indigenous knowledge about spatial organization of visual reality (Bicheool 2012). The practice of colonization of minor ethnicities by big nations has created a real threat of complete absorption and thereby destruction of traditional indigenous cultures (Khakimulina 2009). The same tendency is evident in Samoyedic cultures of Taimyr: as a result of on-going Westernization throughout the XX century, many ethnicities lost their nomadic lifestyle, were handed Russian-based scripting system and became subjected to teaching in Russian, acquired social stratification due to introduction of new professions (i.e. technician, worker), which reduced the share of traditional crafts, especially pronounced amongst females (i.e. common occupations of doctor, teacher that involve working in an institution with hierarchic organization) – altogether resulting in abandonment of traditional reindeer herding that used to be the prime factor determining traditional lifestyle (Bicheool 2013b). Introduction of hierarchic organization in social life and living at a permanent residence are likely to reduce the need in ekmelic musical organization and promote Western tonality. Not surprisingly, younger generations of Samoyedic people become confused about their traditional forms of art and music, mistaking traits of spatial and tonal organization of other ethnicities for their own – explaining their lack of competence by their disinterest in their cultural heritage (Bicheool 2009).

Tonal Western music, along with perspective organization, cultivates completely different set of skills, analytical and analogical in their essence, which are preferable in the modern urban environment, where an individual has to compete with other individuals in execution of rather complex tasks^[[20]](#footnote-20)^. Everyday life a cityscape creates perceptual environment of the “carpentered world” – space characterized by prevalence and salience of straight parallel lines and right angles which do not exist in traditional habitat (Segall, Campbell, and Herskovits 1966). Living in such an environment predisposes one to develop a “perspectival” worldview by establishing a tendency to perceive converging lines as parallel. The experience of the carpentered world makes the discrimination of right angles and non-right angles more precise (Deregowski 1989). Rasterizing the surrounding space in terms of right angles and parallel lines generates unified hierarchic incremental spatial representation. Its social equivalent is life in a highly structured complex society, where every member’s activity is mediated by a number of neighboring in rank members with discrete functionality of subordination and coordination. The musical equivalent of such organization is Western tonality.

This must be the principal reason behind the steady trend of Westernization of world's music – it merely reflects the ongoing process of adoption of Western lifestyle by societies that previously adhered to their own lifestyles. The steady trend of adoption of perspectival organization as a standard of pictorial representation by non-Western cultures manifests the other side of the same coin. It is plausible to conclude that the starting and ending points in evolution of music are shared between many known world’s cultures: at their dawn, archaic traditional music cultures start by abstracting and encoding the orientation schemes that are standard for their native environmental topography, and end up by importing the Western tonality, or hybridizing it with the traditional local forms of tonal organization.

REFERENCES:

Abramovich-Gomon, Alla. 1999. *The Nenets’ Song: A Microcosm of a Vanishing Culture*. Farnham, UK: Ashgate.

Aporta, Claudio. 2004. “Routes, Trails and Tracks: Trail Breaking Among the Inuit of Igloolik.” *Études Inuit Studies* 28 (2): 9. doi:10.7202/013194ar.

Athanasopoulos, George, and Nikki Moran. 2013. “Cross-Cultural Representations of Musical Shape.” *Empirical Musicology Review* 8 (3): 185–99.

Bagashev, Anatolii N., and Olga Ye. Poshekhonova. 2008. “Anthropological Makeup and Problems of the Origin of the Medieval Taiga People of Middle Ob’ Region [Антропологический состав и проблемы происхождения средневекового таежного населения Среднего Приобья].” *The Courier of Archeology, Anthropology and Ethnography* 8: 87–97.

Bamberger, Jeanne Shapiro, and Andrea DiSessa. 2003. “Music as Embodied Mathematics: A Study of a Mutually Informing Affinity.” *International Journal of Computers for Mathematical Learning* 8 (2): 123–60. doi:10.1023/B:IJCO.0000003872.84260.96.

Bendixen, Alexandra, Gábor P. Háden, Renáta Németh, Dávid Farkas, Miklós Török, and István Winkler. 2015. “Newborn Infants Detect Cues of Concurrent Sound Segregation.” *Developmental Neuroscience* 37 (2): 172–81. doi:10.1159/000370237.

Bennardo, G. 2002. “Map Drawing in Tonga, Polynesia: Accessing Mental Representations of Space.” *Field Methods* 14 (4): 390–417. doi:10.1177/152582202237727.

Bicheool, Vladimir. 2009. “Modern State of Nganasan Culture [Современное состояние культуры нганасан].” *The Courier of the Cheliabinsk State Academy of Culture and Arts* 4 (20): 49–54.

———. 2012. “Consequences of Cultural Expansion of the Russian-Speaking Population to Taimyr [Последствия культурной экспансии русскоязычного населения на Таймыре].” *The World of Science, Culture and Education* 5 (36): 17–19.

———. 2013a. “Material Culture of Taimyr Nomads (on the Example of Samodiysky peoples)[Материальная культура кочевников Таймыра (на примере самодийских народов) ].” *The Courier of the Cheliabinsk State Academy of Culture and Arts* 1 (33): 170–75.

———. 2013b. “Colonization in Taimyr: The Socio-Cultural Impacts [Колонизация на Таймыре: социально-культурные последствия].” *Knowledge, Understanding, Skill (ZPU)* 1: 104–8.

Bidelman, Gavin M., and Jeremy Grall. 2014. “Functional Organization for Musical Consonance and Tonal Pitch Hierarchy in Human Auditory Cortex.” *NeuroImage* 101 (November). Elsevier Inc.: 204–14. doi:10.1016/j.neuroimage.2014.07.005.

Bogdanov, I.A. 1982. “Khanty and Mansi Music.” *Encyclopedia of Music [Музыкальная энциклопедия]*. Soviet Encyclopedia [Советская энциклопедия].

Bogoraz, Waldemar. 1991. *Материальная культура чукчей*. Translated by Е. А. Михайлова and И. С. Вдовин. Moscow: Nauka.

Both, Arnd Adje. 2009. “Music Archaeology: Some Methodological and Theoretical Considerations.” Edited by Don Niles and Arnd Adje Both. *Yearbook for Traditional Music* 41. Ljubljana, Slovenia: International Council for Traditional Music: 1–11. www.jstor.org/stable/25735475.

Brodsky, I. A. 1976. “On Study of Music of People of North of Russia [Бродский].” In *Traditional and Contemporary Folk Music Art. Collection of Essays. [Традиционное и современное народное музыкальное искусство. Сборник трудов]*, edited by B.B. Yefimenkova, 29:244–59. Moscow: Gnessin Russian Academy of Music [Российская академия музыки имени Гнесиных].

Chistalev, Prometei. 1976. “Russian-Komi Folk Musical Ties [Русско-Коми фольклорные музыкальные связи].” In *Ethnography and Folklore of the Komi [Этнография и фольклор Коми]*, 16–31. Syktyvkar, Russia: Academy of Science of USSR [Изд-во Академии наук СССР].

———. 1977. “Komi Folk Music [Коми народная музыка].” In *Musical Heritage of the Finnish-Ugoric Peoples [Музыкальное наследие финно-уторских народов]*, edited by I. Ruutel, 451–73. Tallin: Eesti Raamat.

———. 1984. *Komi Folk Musical Instruments [Коми народные музыкальные инструменты]*. Syktyvkar, Russia: Komi Publishing House [Коми книжное изд-во].

Clarke, Eric F. 2001. “Meaning and the Specification of Motion in Music.” *Musicae Scientiae* 5 (2): 213–34. doi:10.1177/102986490100500205.

———. 2005. *Ways of Listening: An Ecological Approach to the Perception of Musical Meaning*. Oxford; New York: Oxford University Press.

Cox, Maureen V. 1998. “Drawings of People by Australian Aboriginal Children: The Inter-Mixing of Cultural Styles.” *Journal of Art & Design Education* 17 (1): 71–79. doi:10.1111/1468-5949.00107.

Dalla Bella, Simone, Anita Białuńska, and Jakub Sowiński. 2013. “Why Movement Is Captured by Music, but Less by Speech: Role of Temporal Regularity.” *PLoS ONE* 8 (8): e71945. doi:10.1371/journal.pone.0071945.

Davies, Stephen. 2012. *The Artful Species: Aesthetics, Art, and Evolution*. Oxford, UK: Oxford University Press.

Deregowski, Jan B. 1989. “Real Space and Represented Space: Cross-Cultural Perspectives.” *Behavioral and Brain Sciences* 12 (01): 51. doi:10.1017/S0140525X00024286.

Devlet, Yekaterina G., and Yevgenii Yu. Girya. 2011. “An ‘Art Layer’ in the Rock Art and Research of the Technique of Execution of the North Eurasian Petroglyphs [«Изобразительный пласт» в наскальном искусстве и исследование техники выполнения петроглифов Северной Евразии].” In *Ancient Art in the Mirror of Archeology [Древнее искусство в зеркале археологии]*, edited by V. Bobrov, O. Sovetova, and Miklashevich E., 7:186–20. Kemerovo, Russia: Kuzbassvuzizdat.

Dikov, Nikolai N. 1971. *Rock enigmas of the Ancient Chukotka: Petroglyphs of Pegtymelia [Наскальные загадки Древней Чукотки: Петроглифы. Пегтымелия]*. Moscow: Nauka.

Dobzhanskaya, Oksana. 2011. “Ritual Musical Folklore of Samoyedic Peoples as an Object of Study: Main Results and Research Perspectives [Обрядовый музыкальный фольклор самодийских народов как объект изучения: основные результаты и перспективы исследования].” In *From Congress to Congress: Materials of the Second All-Russian Congress of Folklore Study [От конгресса к конгрессу. Материалы Второго Всероссийского конгресса фольклористов]*, edited by A.S. Kargin, 3:300–311. Moscow: State Republican Center of Russian Folklore.

———. 2013. “Nganasan Ritual Songs Performed by the Shaman’s Heirs (on Imitation of Shamanic Ritual) [Нганасанские обрядовые песни в исполнении наследников шамана (к проблеме имитации шаманского ритуала)].” In *Epic Heritage and Spiritual Practice in Past and Present [Эпическое наследие и духовные практики в прошлом и настоящем]*, edited by V.I. Kharitonova, 53–64. Moscow: IEA RAN.

———. 2016. “The Live Makes Sounds, the Dead Is Silent [Живое - звучит, мертвое - молчит].” *Anthropology and Archeology of Eurasia*. in print.

Dolscheid, S., S. Hunnius, D. Casasanto, and A. Majid. 2014. “Prelinguistic Infants Are Sensitive to Space-Pitch Associations Found Across Cultures.” *Psychological Science* 25 (6): 1256–61. doi:10.1177/0956797614528521.

Dwyer, Mark J., and Kirill V. Istomin. 2009. “Komi Reindeer Herding: The Effects of Socialist and Post-Socialist Change on Mobility and Land Use.” *Polar Research* 28 (2): 282–97. doi:10.1111/j.1751-8369.2009.00108.x.

Elfenbein, Hillary, and Nalini Ambady. 2002. “On the Universality and Cultural Specificity of Emotion Recognition: A Meta-Analysis.” *Psychological Bulletin* 128 (2): 205–35.

Fortescue, Michael. 2011. *Orientation Systems of the North Pacific Rim*. Kopenhagen: Museum Tusculanum Press.

Frolov, Boris A. 1992. *Primitive Graphics of Europe [Первобытная графика Европы]*. Moscow: Nauka.

Goncharov, Oleg. 2007. *Perception and Representation of the Third Dimension [Восприятие и изображение третьего измерения]*. Syktyvkar, Russia: Syktyvkar University.

Goncharov, Oleg, and Yuri Tiapovkin. 2012. “Cultural and Environmental Factors in the Perception of Perspective Among Indigenous Tundra Inhabitants.” *Journal of Russian and East European Psychology* 50 (5): 65–86. doi:10.2753/RPO1061-0405500504.

Gould, Peter, and Rodney White. 1986. *Mental Maps*. London: Routledge.

Granot, Roni Y., and Zohar Eitan. 2011. “Musical Tension and the Interaction of Dynamic Auditory Parameters.” *Music Perception* 28 (3): 219–45. doi:10.1525/mp.2011.28.3.219.

Gratchyova, Galina N. 1983. *Traditional Worldview of the Taimyr Hunters (on the Materials of Nganasan of the XIX-XX Centuries [Традиционное мировоззрение охотников Таймыра (на материалах нганасан XIX –XX века)]*. Leningrad: Nauka.

Gruhn, Wilfried. 2004. “Are Different Types of Mental Representation Reflected by Brain Activation Patterns?” In *Music Perception and Cognition; ICMPC8*, edited by Scott Lipscomb, Richard Ashley, Robert Gjerdingen, and Peter Webster, 124–27. Adelaide, Australia: Causal Productions.

Hargreaves, David J. 1986. *The Developmental Psychology of Music*. Cambridge, UK: Cambridge University Press.

Ingold, Tim. 2000. *The Perception of the Environment: Essays on Livelihood, Dwelling and Skill*. Psychology Press. https://books.google.com/books?id=S3GakE5OT-kC.

Istomin, Kirill V., and Mark J. Dwyer. 2009. “Finding the Way.” *Current Anthropology* 50 (1): 29–49. doi:10.1086/595624.

Istomin, Kirill V., Jaroslava; Panáková, and Patrick Heady. 2014. “Culture, Perception, and Artistic Visualization: A Comparative Study of Children’s Drawings in Three Siberian Cultural Groups.” *Cognitive Science* 38 (1): 76–100. doi:10.1111/cogs.12051.

Ivanov, Sergei V. 1954. *Materials on Fine Art of Ethnicities of Siberia of 19-Early 20th Centuries: Genre Drawing and Other Types of Depiction on Plane Surface [Материалы по изобразительному искусству народов Сибири XIX-начала XX в: сюжетный рисунок и другие виды изображений на*. Edited by L.P. Potapov. Leningrad: Academy of Science of USSR [Изд-во Академии наук СССР].

Ivanova-Unarova, Zinaida. 2005. *Traditional Art of Ethnicities of North-East Siberia (Evenki, Even, Yukagir, Dolgan, Chukcha, Koryak) [Традиционное искусство народов Северо-Востока Сибири (эвенки, эвены, юкагиры, долганы, чукчи, коряки)]*. Yakutsk: Yakutsk University.

Jones-Bamman, Richard. 2009. “Khynum—The Prayer: Songs of the Nenets.” *Yearbook for Traditional Music*.

Kaeppler, Adrienne. 1990. “The Production and Reproduction of Social and Cultural Values in the Compositions of Queen Salote of Tonga.” In *Music, Gender, and Culture*, edited by Marcia Herndon and Susanne Ziegler, 191–219. Wilhelmshaven: Florian Noetzel Verlag.

———. 2008. *The Pacific Arts of Polynesia and Micronesia*. Oxford ; New York: Oxford University Press.

Khakimulina, Olga. 2009. *History and Culture of Indigenuous People of Taimyr [История и культура коренных народов Таймыра]*. Sankt-Petersburg: Prosvesheniye.

Khlobystin, Leonid. 1998. *Ancient History of Taimyr and the Formation of North Eurasian Cultures [Древняя история Таймырского Заполярья и вопросы формирования культур севера Евразии]*. Sankt-Petersburg: Dmitri Bulanin.

Koelsch, Stefan, and Thomas Fritz. 2013. “Acoustically Mediated Emotional Contagion as an across-Species Homology Underlying Music Processing.” In *Evolution of Emotional Communication: From Sounds in Nonhuman Mammals to Speech and Music in Man*, edited by Eckart Altenmüller, Sabine Schmidt, and Elke Zimmermann, 300–312. Oxford: Oxford University Press.

Krushanov, Andrei I. 1987. *History and culture of Chukchi: historic-ethnographic sketches [История и культура чукчей: историко-этнографические очерки]*. Leningrad: Nauka.

Kubovy, Michael. 1988. *The Psychology of Perspective and Renaissance Art*. New York: Cambridge University Press.

Labanauskas, Kazimir. 1992. *Folklore of the Taimyr Peoples [Фольклор народов Таймыра]*. Vol. 2. Dudinka: Taimyr Regional Center of Folk Art.

Lar, Leonid A. 1998. *Shamans and Gods [Шаманы и боги]*. Tiumen: Institute of the Problems of Exploration of North.

———. 2008. “Traditional Religious Rites in Life of the Nenets [Традиционная религиозно-обрядовая жизнь ненцев].” *Bulletin of the Russian State Pedagogical University of A.Herzen* 62: 98–108.

Lazar, Katalin. 1997. “Folk Songs of the Eastern Ostyaks.” In *Studies on Surgut Ostyak Culture*, edited by Márta Csepregi and Ágnes Kerezsi, 42:109–46. Budapest: Néprajzi Múzeum.

Lehtisalo, Toivo. 1998. *The Mythology of Yurako-Samoyed (Nenets) [Мифология юрако-самоедов]*. Translated by Nadezhda V. Lukina. Tomsk: The Tomsk University Press.

Leman, Marc. 2008. *Embodied Music Cognition and Mediation Technology*. Cambridge, MA: MIT Press.

Lewis, David H., and Mimi George. 1991. “Hunters and Herders: Chukchi and Siberian Eskimo Navigation Across Snow and Frozen Sea.” *Journal of Navigation* 44 (1): 1. doi:10.1017/S037346330000967X.

Lukina, Nadezhda F. 1985. *Formation of the Material Culture of Khanty [Формирование материальной культуры хантов]*. Tomsk: University of Tomsk.

Lythberg, B. J. 2013. “Polyvocal Tongan Barkcloths: Contemporary Ngatu and Nomenclature at the Museum of New Zealand Te Papa Tongarewa.” *Tuhinga: Te Papa’s Research Journal* 24: 85–104. http://hdl.handle.net/2292/27612.

Marks, Lawrence E. 1978. *The Unity of the Senses: Interrelations among the Modalities*. New York: Academic Press.

Martynova, Yelena P. 1998. *Sketches on history and culture of Khanty people [Очерки истории и культуры Хантов]*. Moscow: Russian Academy of Science [Ин-т этнологии и антропологии РАН].

McDermott, Josh H., Michael V. Keebler, Christophe Micheyl, and Andrew J. Oxenham. 2010. “Musical Intervals and Relative Pitch: Frequency Resolution, Not Interval Resolution, Is Special.” *Journal of the Acoustical Society of America* 128 (4/1): 1943–51. doi:10.1121/1.3478785.

McDermott, Vincent. 1972. “A Conceptual Musical Space.” *Journal of Aesthetics and Art Criticism* 30: 489–94.

Mitlianskaya, T.B. 1983. *On Folk Art Crafts of Siberia and Far East for Village Teacher [Сельскому учителю о народных художественных ремеслах Сибири и Дальнего Востока]*. Moscow: Prosvesheniye.

Mohn, C., H. Argstatter, and F.-W. Wilker. 2010. “Perception of Six Basic Emotions in Music.” *Psychology of Music* 39 (4): 503–17. doi:10.1177/0305735610378183.

Niemi, Jarkko. 1999. “The Genres of the Nenets Songs.” *Asian Music* 30 (1): 77–132. doi:10.2307/834981.

———. 2002. “‘You Are of the Kind of Kin, Are You?’ Reflections of the Animistic World View in the Oral Folklore of the Modern Day Kanin Nenetses.” In *Mental Spaces and Ritual Traditions. An International Festschrift to Commemorate the 60th Birthday of Dr. Mihály Hoppál*, edited by Elek Bartha and Veikko Anttonen, 401–16. Debrecen: University of Debrecen.

———. 2009. “Evaluating Parameters of Structural Analysis in Indigenous Siberian Singing.” In *Perspectives on the Song of the Indigenous Peoples of Northern Eurasia: Performance, Genres, Musical Syntax, Sound*, edited by Jarkko Niemi, 88–121. Tampere, Finland: Tampere University Press.

Nikolsky, Aleksey. 2015. “Evolution of Tonal Organization in Music Mirrors Symbolic Representation of Perceptual Reality. Part-1: Prehistoric.” *Frontiers in Psychology* 6 (1405). doi:http://dx.doi.org/10.3389/fpsyg.2015.01405.

Novik, Yelena. 2004. *Rite and Folklore in Siberian Shamanism: An Experiment in Correlation of Structures [Обряд и фольклор в сибирском шаманизме: Опыт сопоставления структур]*. Moscow: Eastern Literature, Russian Academy of Science [Восточная литература РАН].

Ojamaa, Triinu. 2002. “The Story of Life in Music: Autobiographical Songs of the Nganasans.” *Folklore: Electronic Journal of Folklore*. doi:10.7592/FEJF2002.21.songs.

Ojamaa, Triinu, and Jaan Ross. 2004. “Relationship between Texts and Tunes in the Siberian Folksongs.” In *CIM04: Conference on Interdisciplinary Musicology*, edited by R. Parncutt, A. Kessler, and F. Zimmer, 134–35. Graz, Austria: Graz University.

———. 2011. “The Perceived Structure of Forest Nenets Songs: A Cross-Cultural Case Study.” *Psychomusicology: Music, Mind & Brain* 21 (1/2): 159–75. doi:100.1037/h0094010.

Perlovsky, Leonid. 2012. “Cognitive Function, Origin, and Evolution of Musical Emotions.” *Musicae Scientiae* 16 (2): 185–99. doi:10.1177/1029864912448327.

———. 2014. “The Cognitive Function of Music. Part II.” *Interdisciplinary Science Reviews* 39 (2): 162–86. doi:10.1179/0308018813Z.00000000041.

Petrova, Anna G. 2014. “Universal Composition Forms in Arctic Peoples’ Ornament [Универсальные композиционные формы в орнаменте народов Арктики].” *Gramota* 2 (40): 150–53.

Petrova, Valentina P., and Galina P. Khariuchi. 1999. *The Nenets in History of Yamalo-Nenets Autonomous Region [Ненцы в истории Ямало-Ненецкого автономного округа]*. Tomsk: The Tomsk University.

Pushkaryova, Yelena T. 2001. “The Specification of Genres of Nenets Folklore and Their Performance Tradition [Специфика жанров фольклора ненцев и их исполнительской традиции].” In *The Nenets Folkore [Фольклор ненцев: в записях 1911, 1937, 1946, 1953, 1965-1987 годов]*, edited by Yelena T. Pushkaryova and Liudmila Khomich. Novosibirsk: Nauka.

Rafaenko, V. Ya. 1972. “By the Reindeer’s Path: Folk Applied and Fine Art of Nenets [Тропою оленей: народное прикладное и изобразительное искусство ненцев].” In *Rainbow on the Snow: Culture, Traditional and Contemporary Art of the Ethnicities of Soviet Extreme North [Радуга на снегу: Культура, традиционное и современное искусство народов советского Крайнего Севера]*, 97–109. Moscow: Molodaya Gvardiya.

Rosser, R A, S S Ensing, J Mazzeo, and P F Horan. 1985. “Visual Perspective Taking in Children: Further Ramifications of an Information-Processing Model.” *The Journal of Genetic Psychology* 146 (3): 379–87. doi:10.1080/00221325.1985.9914466.

Sadek, Amal A. M. 1987. “Visualization of Musical Concepts.” *Bulletin of the Council for Research in Music Education*, no. 91 (April): 149–54.

Schadwinkel, Stefan, and Alexander Gutschalk. 2010. “Activity Associated with Stream Segregation in Human Auditory Cortex Is Similar for Spatial and Pitch Cues.” *Cerebral Cortex* 20 (12): 2863–73. doi:10.1093/cercor/bhq037.

Segall, Marshall H., Donald Thomas Campbell, and Melville Jean Herskovits. 1966. *The Influence of Culture on Visual Perception*. Bobbs-Merrill Co. https://books.google.com/books?id=2V1AAAAAIAAJ.

Sheikin, Yurii. 2002. *History of music culture of Siberia peoples: comparative-historic investigation [История музыкальной культуры народов Сибири: сравнительно-историческое исследование]*. Moscow: Eastern Literature, Russian Academy of Science [Восточная литература РАН].

———. 2008. “Ethnomusicology as the Discipline at Arctic State Institute of Arts and Culture [Этномузыкология как дисциплина в Арктическом государственном институте искусств и культуры].” *Vestnik AGIIK [Вестник Арктического государственного института культуры и искусств]* 1 (1): 37–48.

Shirokov, Vladimir N., and Sergei Ye. Tchairkin. 2011. *Rock Images of Northern and Middle Ural [Наскальные изображения Северного и Среднего Урала]*. Yekaterinburg: Ashur.

Siazi, Antonina M. 1995. *Decorative Applied Art of Low Ob Khanty [Декоративно - прикладное искусство хантов Нижней Оби]*. Tiumen’: Institute of the Problems of Exploration of Northern Siberia.

Sievers, Beau, Larry Polansky, Michael Casey, and Thalia Wheatley. 2013. “Music and Movement Share a Dynamic Structure That Supports Universal Expressions of Emotion.” *Proceedings of the National Academy of Sciences of the United States of America* 110 (1): 70–75. doi:10.1073/pnas.1209023110.

Smith, J. David. 1997. “The Place of Musical Novices in Music Science.” *Music Perception* 14 (3): 227–62.

Soldatova, Galina Ye. 2012. “Musical Folklore of Ob Ugrians [Музыкальный фольклор Обских угров].” In *People Bathing in Sounds [В звуках купающиеся люди]*, edited by A.V. Lebedeva and M.A. Lapina, 4–15. Khanty-Mansiisk, Russia: Print-Class.

Szabolcsi, Benedict. 1935. “The Eastern Relations of Early Hungarian Folk-Music: (The Persistence of an Archaic Middle-Asian Music-Style in Middle-Europe).” *The Journal of the Royal Asiatic Society of Great Britain and Ireland*, no. 3: 483–98. http://www.jstor.org/stable/25201171.

Tchernetsov, Valerii N. 1971. *Rock Images of Ural [Наскальные изображения Урала]*. Moscow: Nauka.

Trainor, Laurel J. 2007. “Do Preferred Beat Rate and Entrainment to the Beat Have a Common Origin in Movement?” *Empirical Musicology Review* 2: 17–20. http://hdl.handle.net/1811/24480.

Trainor, Laurel J., Xiaoqing Gao, Jing-jiang Lei, Karen Lehtovaara, and L.R. Harris. 2009. “The Primal Role of the Vestibular System in Determining Musical Rhythm.” *Cortex* 45 (1): 35–43. doi:10.1016/j.cortex.2007.10.014.

Uspensky, Boris. 1976. “The Language of Ancient Painting.” *Dispositio* 1 (3): 219–46.

Vdovin, Innokentii. 1965. *The sketches of history and ethnography of the Chukchi [Очерки истории и этнографии чукчей]*. Leningrad: Nauka.

Veres, Peter. 2014. “The Etiological Myth of Obugrians on the Origins of Phratrial Organization, and Their World model.[Этиологический миф обских угров: происхождение фратриальной организации и модель мира].” *Transactions of the Karelian Research Centre of Russian Academy of Science* 3: 43–52.

Walker, Robert. 1985. “Mental Imagery and Musical Concepts: Some Evidence from the Congenitally Blind.” *Bulletin of the Council for Research in Music Education*, no. 85 (October): 229–37.

———. 1987. “Some Differences between Pitch Perception and Basic Auditory Discrimination in Children of Different Cultural and Musical Backgrounds.” *Bulletin of the Council for Research in Music Education*, no. 91 (April): 166–68.

———. 2004. “Cultural Memes, Innate Proclivities and Musical Behaviour: A Case Study of the Western Traditions.” *Psychology of Music* 32 (2): 153–90. doi:10.1177/0305735604041493.

Wilson, Brent, and Johan Litgvoet. 1994. “Across Time and Cultures: Stylistic Changes in the Drawings of Dutch Children.” In *Drawing Research and Development*, edited by D. Thistlewood, 75–88. London: Longman.

Winner, Ellen. 2007. “Development in the Arts: Drawing and Music.” In *Handbook of Child Psychology*, edited by William Damon and Richard M. Lerner, 859–904. Hoboken, NJ, USA: John Wiley & Sons, Inc. doi:10.1002/9780470147658.chpsy0220.

Zemtsovsky, Izaly. 1983. “Song as a Historic Phenomenon [Песня как исторический феномен].” In *Popular Song: Problems of Study [Народная песня. Проблемы изучения]*, edited by V. Gusev, 6:22–35. Leningrad: Saint Petersburg State Theatre Arts Academy [ЛГИТМИК].

Zentner, Marcel. 2012. “A Language for Musical Qualia.” *Empirical Musicology Review* 7 (1/2): 80–83.

1. The word “and” here refers to the expressive aspects of music that interact with one another in order to produce an impression of a particular physical attribute – otherwise, instead of the word “and” comma is used. [↑](#footnote-ref-1)
2. I have put forward the outline of such emotional semiosis in music in the following research paper: https://www.researchgate.net/publication/291974302_How_Emotion_Can_Be_the_Meaning_of_a_Music_Work [↑](#footnote-ref-2)
3. Although imagery perception of speech involves mental visualization, however, the connection between a specific image and the configuration of the sound of speech, is governed entirely by the learned *indexical* reference of word to its meaning – and not by the expressive “*iconic*” attributes of sound “as such,” as we hear it in music (Ingold 2000, 248). There does exist the phenomenon of onomatopoeia in language, but it is of very limited use in pragmatics of speech, and definitely does not constitute a system of transmission of information anywhere close to that of tonal organization in music. [↑](#footnote-ref-3)
4. The concept of “archaic” in relation to folk culture was introduced by Bartok and Kodaly in reference to the type of music that characterized Hungarian folk tradition at its earliest date of forming its own ethnic identity – in contradistinction to the later stylistic additions through cultural borrowing and elaboration (Szabolcsi 1935). Modern ethnomusicologists usually employ the term “archaic” to refer to a primordial musical culture that predated formation of “national” cultural features, and reflected a more biological, instinctive, use of music by the individual as an expression of his mediation with nature (Sheikin 2002, 4–7). [↑](#footnote-ref-4)
5. Shaman’s songs might constitute a peculiar case where the shaman sings on the behalf of a client in an attempt to mediate with a particular spirit, thereafter acquiring the voice and the personal song of that spirit in order to answer the request previously sung by shaman’s natural voice. [↑](#footnote-ref-5)
6. Unfortunately, matters of orientation, spatial and tonal organization that are habitual to different Aboriginal ethnicities of Siberia are only in embryonic state of investigation. There is much that remains to be discovered, and this paper just barely touches upon this important area of research. [↑](#footnote-ref-6)
7. This point is one of the most crucial, since most of the Creole Aboriginal population lose command of their native tongue, abandon their traditional beliefs, and tend to follow the metropolitan Russian cultural stereotypes (Bicheool 2012). [↑](#footnote-ref-7)
8. All the subjects followed the same strategy: they drew the front side of the cube as a square, then added the left side, and then the invisible bottom side. Upon comparing their drawing to the original cube before them, they became dissatisfied with what they had drawn, and made several more attempts to complete the drawing, but every time, to their surprise, they produced the same result. Only 2 out of 20 adult subjects managed to draw the cube in the form of combined rectangles. None of the 22 children could draw the cube. On those drawings that contained multiple sides, the near cube side was depicted narrower and shorter than the one on the far side. Another source of difficulty was the task of selecting projections, where subjects had to choose the best match between a few 3-D objects of various geometric shapes, and their photos taken from 8 different angles. Majority of children could correctly identify only one to two of the ten projections. The execution of all tasks by adults and children exhibited more commonalities than individual differences. [↑](#footnote-ref-8)
9. Golovnev insists on this what he terms ‘celestial’ aspect of Nenets navigation as a characteristic trait of their culture, claiming that, unique to their region, they move according to rotation of the sky-picture, shifting sight to the adjacent star in the direction of their travel every 7-10 kilometers, thereby, making simultaneous use of the land and sky maps. [↑](#footnote-ref-9)
10. The only genre of realistic representation was siadei – an idol with human features carved on a totem-like wooden block, erected in a sacrificial place (Rafaenko 1972). But even this genre became indisputably anthropomorphic only in the late 19th century, under the Russian cultural influence. Prior to that representation was symbolic – connecting a *siadei* to the spirit that it was supposed to call upon. [↑](#footnote-ref-10)
11. This strategy of connecting the distant pitches by insertion of quick passages is characteristic for improvisations of blind keyboard and string players. [↑](#footnote-ref-11)
12. Michael Fortescue reports similar contrast between nomadic and sedentary methods of navigation in relation to the inhabitants of Chukotka and Kamchatka (Fortescue 2011, 67) – suggesting that commonality of tonal organization might correspond to commonality of lifestyle, to a great degree determined by commonality of geographic conditions. [↑](#footnote-ref-12)
13. This uninterrupted method of delivery of melodic line has become a performance school in Nenets music: *teltangova* (from “te’eltango” – “repeat”) – a system of singing by the leading singer (*meta*) and his assistant (*teltanzyoda*), used to perform all the traditional Nenets genres except only four genres of personal songs, riddles, *bylichka* and *byval’shina* (Pushkaryova 2001). [↑](#footnote-ref-13)
14. Thus, the informant Nenets was saying that he knew every tussock near his native village, but he could not point to the location of that village from his current position of the pasture. [↑](#footnote-ref-14)
15. The informants insisted that once inside the region they could point to “every tussock” from any given point, but once outside of the region they were unable to tell most of the places inside that region. Their mapping seemed to proceed in two independent stages: using the environmental hydro-system to break the area into plots, and then learning the disposition of various landmarks within each plot. The latter was viewed as much harder, requiring special qualifications. Thus, Nenets were telling that they could navigate by watersheds to get to a very good pasture about which they had heard, but gave the following reason for not going there: “to live in a place one has to know the land, and not just the rivers… to know the land we need an old man (*vesako*) to go there” (Istomin and Dwyer, 2009, 40). [↑](#footnote-ref-15)
16. Although there are accounts of 3-string plucking instrument, 2-string bowing instrument, 1-string percussive instrument and whistles being used by Chukchi in the past, no public performances on such instruments have been known since 1954 (Krushanov 1987, 236). [↑](#footnote-ref-16)
17. Of course, this tendency would hold true only for the traditional art of a decidedly ekmelic culture – and not for the drawings of the children who were actually taught to draw at school, as in the study by Istomin, Panáková, and Heady. [↑](#footnote-ref-17)
18. Granot and Eitan (Granot & Eitan 2011) explain the cross-modal correspondence between low register, falling pitch contour, tempo acceleration, and tension, which they discovered in their experiment, by proposing an ecological model, formulated by Clarke (Clarke 2005) According to it, the tension effects are experienced by the listeners as a result of their association of auditory variables with psychophysiological tension and arousal triggered by “natural” causes in their cultural habitat (i.e. association of low pitch with growling and aggression). [↑](#footnote-ref-18)
19. Boris Uspensky (Uspensky 1976) explains this difference by the contrasting strategies in encoding of space employed by the Renaissance linear perspective and historically earlier depicting methods. He points out that the viewer of pre-perspectival art was supposed to draw conclusion from the depicted image based on summation of multiple gazes on the picture, gliding along it in a particular order, and gathering information. Viewing a perspectival image requires instantaneous decoding of the optimal vantage point that works like a key that “unlocks” the hierarchical organization in the image – remarkably similar to the key in tonality that unlocks the map of tonal tension. [↑](#footnote-ref-19)
20. Even a pizza delivery boy has to know how to drive a car, operate a cellular phone, look for addresses, know basic math, have reading and writing skills. [↑](#footnote-ref-20)
