# Supplementary material for: Evolution of Tonal Organization in Music Optimizes Neural Mechanisms in Symbolic Encoding of Perceptual Reality. Part-2: Ancient to Seventeenth Century
Source: Front Psychol. 2016 Mar 30;7:211. doi: 10.3389/fpsyg.2016.00211 (PMC4813086; doi:10.3389/fpsyg.2016.00211)
Supplement: Supplementary file 3 [file DataSheet1.zip › Appendices I-VIII/Appendix ViII. Summary of Stages of Tonal Development.docx]

# Appendix VIII: Summary of Schemes of Tonal Organizations

Overall, there are 14 schemes of tonal organization that probably occurred through 10 stages of evolutionary development. Stages 1-6 went cumulatively, until the divergence of pentatony and heptatony. Then, heptatony kept evolving into a chromatic system, which in turn diverged from the diatonic system and the diatonically organized hypermode. It seems that the third divergence took place during the last three centuries: Ancient Greek chromatic system produced Western tonality and hemiolic modality. Their opposition is hard to define in structural terms of music theory, because of their historic modernity as well as lack of documentation on the origin of modes with augmented 2^nd^ – however, there is enough material to conclude that both tonal organizations present competing approaches to musical composition and consumption.

Below, is an overview of each of the schemes with a brief explanation of the reasons for how they are dated.

1) **Pre-Mode (ca. 700,000 BC).**^[[1]](#footnote-1)^ The ground zero of tonal organization seems to be the vocalized pure pitch contour without any regard for exact intervallic distance and permanence of frequency values of the tones that constitute a pitch contour. Its vocalization is executed through conscious control of the changes in the pitch direction, only approximately coordinated in time. The prototype of such organization would be a pack of wolves howling together: adherence to a single signal model, with multiple random deviations in pitch, very roughly synchronized in time. The modeled parameters would have included timbre, frequency, dynamics, and rhythm. Their regulation was probably instinctive “echolaliac” – holistic in essence: processed as a “single-bit” snapshot of a timbral/pitch/dynamic contour.

Processing of melody by pre-2 year old infants presents a glimpse of such “single-bit” composition based on holistic treatment of indefinite pitch intonations (McKernon 1979). Gliding pitch plays the key role in tonal organization of such vocalization, making directional cues more prominent than intervallic cues (Fancourt, Dick & Stewart 2013).

The modern newborn is essentially in the same position as the Neanderthal newborn – except that the Neanderthal baby did not have models of tonal music performed in his environment, and was therefore likely to “get stuck” in his initial tonal scheme. Progression to some other scheme would have required many generations of music users adhering to the same new melodic model. The smallest upgrade of the tonal scheme must have taken a lifetime of an entire generation in order for the new convention to be formed and adopted by the following generation – provided that the tribe would not go extinct and would export its repertory of proto-musical signals to other tribes. The sonic environment of Paleolithic man must have included animal-like grunts, expressing positive emotions, and aggressive shrieks, used for hunting or confrontation, with later addition of narrative intonations, as linguistic communication was taking shape.

Both, music and language, feature “vocal production learning” (Merker 2012) – a capacity to match an auditory model by shaping one’s vocal output. Collective vocalization, uncoordinated in intervals, provided the ground for learning “melodic skills.” Collective syllabification of gliding contours generated accents, thereby enabling the formulation of stereotypical melodic phrasal units. They were probably categorized by their melodic contour in relation to the entire ambitus of one’s vocal range: the starting, finishing, and climactic points served to draw organizational maps reflecting the distribution of pitch across the time-line, and to memorize a particular contour in terms of roughest estimations of “high” and “low.”

2) **Khasmatonal Mode (ca. 250,000 BC)**.^[[2]](#footnote-2)^ The most favorite melodic contours were used over and over again within the same community, forming the melodic repertory. Individual users developed “personal songs” that contained their unique signature of timbral transformations, pitch-bends, and special effects like vibrato or “dirty” phonation. Motif-formulae were vocalized in an unnatural manner, to differentiate them from verbal vocalization.

The prototype of khasmatonal organization can be found in babbling of the 2-3 year old children, when they explore leaps by at first making them per chance and later learning to fill them up with more gradual intervallic distances (Davidson 1994). Such composition is driven by the cognitive opposition of leap to step, where leap earns its association with tonal tension, and step – tonal relaxation. An entire melody can be mapped in this way.

Reference to a register becomes pivotal for khasmatonal organization - “a phonation frequency range in which all tones are perceived as being produced in a similar way and that possess a similar voice timbre” (Sundberg 1987, 49). Infants learn meaning of particular voice registers before they learn meaning of particular words (Sicoli 2015). The singing register is less of what the listener hears, and more of what the singer experiences while trying to sustain a desirable tonal quality (Miller 2000). Therefore, register works as a peculiar delimiter that helps the singer navigate through the available compass of tones by permanently mapping certain tones to certain sensations in the dedicated spots of the vocal folds and the vocal tract, creating what has been known to vocal coaches as “vocal coordination.”

Abrupt contrast in register was the first strictly musical means of formatting a melodic line. Cultivation of leaps in dedicated register of a song's ambitus brought to life the khasmatonal mode, defined by timbral transformation over a register and sequential order of tones. Changes in pitch only supported timbral changes, as evident in existing examples of khasmatonal organization in ethnicities of Extreme North (Mazepus 2009), which require timbral analysis in order to reflect their musical composition (Eerola 2009). Importance of timbral changes in tonal organization of such music prompted ethnomusicologists to distinguish “timbre-centered music” from “pitch-centered music” (Levin and Süzükei 2006, 51).

Usage of timbral markers in an individual voice is likely to emphasize the contrast of registers, which then acquires a reference role, formative for mental representation of pitch. Vocal ambitus is usually divided in 2-4 registers, and 3 registers for pre-pubertal boys and girls (McAllister, Sederholm, and Sundberg 1993). Placement of a pitch envelop across the registers would mark specific tones of the envelop as belonging to this or that register, thereby locking them within the range of that register, which could be further specified as to which part of the register a tone belongs. Khasmatonal vocal coordination was likely based on the sensation of pitch continuity within a register versus leaps between registers.

Texture-wise, khasmatonal music probably remained primordially polyphonic, without any vertical intervallic coordination. The example of such organization is described by Anthony Seeger in relation to the Amazon Indians (Seeger 2004). It is better called “isophony,” to distinguish it from polyphony by its uncoordinated vertical harmony and its going in and out of phase in time when every participant reproduces the same pitch contour – making parts sound fragmentary, featuring brief motifs of similar size (see Appendix V).

3) **Ekmelic Mode (ca. 50,000 BC)**.^[[3]](#footnote-3)^ Singing in resonant caves with the accompaniment of pitched instruments emphasized certain frequencies that were used for reference. The contrast between ascending and descending intonations, as well as lyrics, stressed certain pitches, causing their coordination in pitch. Some tones became more permanent in tuning than the rest of the tones.

Singers associated a specific intonation with a specific register: tones were still unfixed in pitch, but defined within a more narrow range of pitch values. Melody acquired somewhat flexible "degrees." The 2-4 coordinated anchor points were used for melodic navigation. The nucleus of ekmelic mode had tendency to expand in a centrifugal manner, as the performer was getting more excited. Pitch outweighed timbre in its navigational importance, and frequency transformation over register became the prime melodic vehicle.

The developmental equivalent of the “ekmelic stage” is the acquisition of vocal skills by the 3-5 year olds. This is when children start forming skills of chest-like phonation by learning to engage the growing thyroarytenoid muscle (Grachiova 1971) in interaction with abdominal muscles (Stulova 1992, 44–45), with the contribution of resonance in the cavities, palates, nose, mask, diaphragm, and even feet - which also regulate singing together with the kinesthetic and baro-receptive components of the feedback corrective mechanisms for adjusting the voice (Morozov 1977, 148–158). Preschool children use chest voice (Bagadurov 1953, 52) together with head voice (Yakovlev 1958) and voluntarily alternate between chest and falsetto registers (Dmitriyev 1968, 427). Chest-voice coordination becomes usually established during the period of 5-9 years of age (Bogomilsky & Chistiakova 2008, 111) – following what can be called ekmelic method of orientation within a designated register.

At first, there are only two of these registers that are pretty narrow, about a 3^rd^ each, and as the child grows, they increase in number and size. Quite similar is the development of ekmelic mode in Sakha folk music, as established by Eduard Alekseyev (Alekseyev 1976): two degrees in the oldest samples of epic music, olonkho, and up to 4 degrees in more modern genres. Two reference points become defined by the trough and apex points in a wavelike pitch contour, with two additional degrees added later by functional relation to the principal two.

Degrees can compliment, oppose, or extremize (polarize) each other, projecting melodic attraction or repulsion. Thereby, the notions of 4 intervals relative in size are formed, defined by tonal functionality. Unison always anchors, 2^nd^ compliments – one of its tones attracts another, whereas 3^rd^ opposes tones (either by creating competition between two anchors or two complimenting tones). The interval of 4^th^ extremizes: generates “khasmatonal” relationship of maximal discontinuity between two tones.

Functionality of these indefinite in pitch ekmelic intervals sets in place their horizontal consonance/dissonance valence. Unison and 2^nd^ constitute perfect consonance with their smooth relation of tones, 3^rd^ becomes imperfect consonance, presenting a small change in tonal leaning, especially obvious in transition from one anchored tone to another. And 4^th^ represents dissonance by its distinct leap. Ekmelic organization generates the first discrete intervallic typology, albeit relative in pitch values, yet defined by functional relationship of the tones in a melodic formula – therefore, comprising the first clear case of “musical mode” in a strict sense of the word, where every tone forms different intervals in relation to every other tone, thus, establishing the harmonious relations within a set of tones.

Discrimination between horizontal consonance and dissonance institutes the first gravitational scheme of instability/stability that acquires the association with tension and relaxation. Consonant unison and 2^nd^ obtain the functionality of, respectively, stability and attraction. Less consonant 3^rd^ and dissonant 4^th^ obtain the functionality of melodic repulsion. If attraction tends to keep the frequencies close to each other, repulsion moves them apart. This sets in place the first form of melodic dynamism: melodic line starts “moving.”

Melodic motion shapes the categorization in pitch: stable and complimentary tones display less variability in tuning, whereas opposed or extremized tones vary in tuning substantially more. Strong melodic inertia makes wider intervals very elastic, only approximately maintaining their relative size. Inertia is also responsible for the overall prevalence of centrifugal gravity: the ambitus of an ekmelic song tends to keep spreading wider, fueled by repulsion of the extremized tones of the interval of a 4^th^.

Ekmelic music promotes monodic and responsorial types of texture. The imperative condition for this process of pitch fixation remains to be the flattening of texture: if in primordial isophony there are as many parts as there are singers, ekmelic music requires monophonic melodic line and culture of personal singing.

4) **Oligotonal Mode (34,000 BC)**.^[[4]](#footnote-4)^ Ekmelic culture instituted melodic thinking in numerical terms within a registral range: by conceptualizing pitches as a bunch of possible “brush”-values, some thicker, others thinner, all vertically coordinated, where one would be higher or lower than another. Resolving pitches into discrete modal degrees formed the basis for fixation and categorization of different modes. If ekmelic and khasmatonal music demonstrated high uniformity across remote cultures, oligotonal music germinates idiosyncrasy in modal organization. One oligotonal mode can considerably differ from another (i.e. Sakha from Russian, or a personal song of one individual from another).

This stage corresponds to the 4-6 year-olds’ acquisition of musical skills, when children start learning culture-specific melodic intervals (Louhivuori 2006). Davidson (1994) points out the importance of “step” and “leap” dialectics in development of children’s improvisatory and imitative songs. Evidently, the primordial distinction here lies between “step” and “leap” realized through the trichordal model of a 3^rd^ that is made up by adding two similar 2^nds^. Such tonal system is intervallicly binary: based on the contrast between leap (3^rd^) and step (2^nd^).

Once the idea of constructing a leap by means of isomorphic steps becomes assimilated into compositional strategies, it can be tested on a wider leap of a 4^th^. Filling it up by steps equidistant with the steps utilized in the earlier trichordal model, would produce a tetrachordal model that affords three types of intervals: a step (2^nd^), a conglomerate of steps (3^rd^), and a leap (4^th^) – providing the ground for grasping the idea of intervallic categorization.

Any singing of contours that included leaps over degrees engages the step/leap dialectics, introduces the need for conservation of interval size, and promotes observation of some increment in distancing the degrees. Oligotonal degrees are conceptualized in numerical order, in the manner of a scale, and all unstable neighbors receive a complimentary function. Complimenting and anchoring become “class-functions.”

Ekmelic intervals already put in place the idea of incrementality embedded in numerology of the intervals: although intervals were stretchable, generally, 2^nd^ was larger than unison, 3^rd^ larger than 2^nd^, and 4^th^ larger than 3^rd^. Oligotonal intervals make incrementality more well-defined. Tones involved in leaping become narrow-tuned, establishing typology of intervals of absolute size, especially through collective performance. Musicians learn to distinguish between melodic consonance (displacing interval) and dissonance (tracing interval), with further distinction between vertical consonant/dissonant traces of 3^rd^ and 4^th^. Oligotonal texture affords heterophony, allowing for vertical as well as horizontal implementation of intervals.

5) **Mesotonal Mode (17,000 BC)**.^[[5]](#footnote-5)^ Consolidation of the fixed in pitch 4-degree oligotony tends to assign a particular formative importance to the interval of 4^th^ and 2^nd^. Mesotonal mode capitalizes on this tendency, usually employing step equivalence along with trichordal or tetrachordal organization. Unison, 2^nd^, 3^rd^, and 4^th^ become standardized and distinguished in melodic and harmonic aspects of composition as the discrete building blocks.

Dissonance/consonance valence and gradations of degrees in permanence/variability of pitch establish the phenomenon of “tonal resolution” by grouping the resolving and the resolved tones together. This mesotonal innovation bears far-reaching consequences: once the combination of adjacent tones is realized as a “resolution cell,” the other degrees in the mode become rasterized in terms of such cells, leading to further refinement of pitch. Oligotonal “brush-like” pitch culture is supplanted by mesotonal “point-like” pitch. Finer tuned degrees form riverbeds for specific intonations that acquire the importance of characteristic modal intonations (what Huron called “tendency tones” (2006, 160)) – retained from song to song, making songs sound similar. This leads to formation of the repertory of modes, each associated with a particular expression, usually fixed by the framework of a particular musical genre.

Each mode, in effect, receives a specific gravitational map. Following this map secures reproduction of the “same” kind of melody, molded by the fixed riverbeds of the “tendency tones.” Composing a new song becomes the matter of taking an existing mode and filling it up with some new melodic material. The resulting song will necessarily be of the same kind as the other songs created in this mode. Mesotonal modes disclose certain capriciousness: they often feature directional versions (like melodic minor), if the “tendency tones” are only one-way.

Mesotonal stage corresponds to the age group of 6-7 year-olds in acquisition of musical skills, when children develop harmonic and tonal ear in detecting vertical intervals and progressions of vertical harmonies (Trainor & Trehub, 1994). This stage is characterized by increase in the ambitus of comfortable singing compass from about a 5^th^ at 4 years of age, to a 6^th^ at 5, and further expansion at age 7 (Radynova, Katinene, and Palavandishvili 1994, 101–104) – very much like the oligotonal mode which organically grows into mesotonal, which in turn progresses into multitonal mode. Neighboring degrees start contrasting in stability/instability, as they acquire either leaning or passing functions in the melodic line. Unstable degrees form permanent auxiliary functionality in relation to neighboring stable degrees, and complimentary functionality becomes the property of the degrees that share the same valence: odd (I-III) or even (II-IV). Anchoring, auxiliary and complimentary functions become “class-functions” assigned to a specific degree, determining the intervals afforded on it.

The difference between older “complimentary” functionality, rooted in ekmelic organization, and new “complimentary” functionality, related to valence of pitches, is the result of emergence of vertical harmonization. The old complimentarity of neighboring degrees was a product of the *horizontal* harmonization. The new complimentarity of degrees that share the same valence (odd or even) is the consequence of collective music-making and experiments with heterophony and multipart textures, thereby related to *vertical* harmonization. Separation of melodic-based auxiliary function from harmonic-based complimentary function characterizes the evolution towards heptatonic tonal organization. Pentatonic organization does not follow their distinction.

Class-functionality of hemitonic modes gradually locks all the degrees to more or less fixed pitch values. Consequently, melodies incorporate rough interval-based transposition and textural dubbing, paving the road toward homophonic thinking. Cultures with well-developed poetry advance to the next stage of tonal development, where the hierarchy of accents in words generates hierarchy of pitches within the mode. Thus, emerges the equivalence of 3^rd^: two odd degrees receive permanent fixation in pitch, and acquire function of stability conceptualized into the “tonic” vertical 3^rd^, while two even degrees become loose in pitch, sharpening or flattening, depending on the direction of the melodic line.

At this point the mesotonal modes start breaking into hemitonic and anhemitonic varieties. Succession of a loose unstable degree by a permanently tuned stable degree marks the general “resolution skeleton” for the entire mode, providing the backbone for integration of all tones in a single tonal entity. In anhemitonic mode, the pairs of resolution are distanced from one another by a gapped disjunct connection (i.e. C-D, F-G, Bb-C), producing the equivalence of a 4^th^. In hemitonic mode, they follow one another with a step disjunct connection (i.e. C-D, E-F, G-A), generating the equivalence of a 3^rd^. They can also form a more rare “natural microtonal” mode by engaging a conjunct connection between the pairs of resolution (i.e. C-D, D-E, E-F). Such modes resort to the equivalence of a 2^nd^, and eventually develop “natural chromatic” degrees (Db between C-D, Eb between D-E etc.). A number of Balkan cultures use such diaphonic modes in multipart settings.

The hemitonic IV degree turns into what is most tonally challenging to the I degree – offering an alternative anchor. The interval of 5^th^ and tritone become common and introduce extra gradations in consonance and dissonance in the IS. Variety in intervals enables diphonic textures, as well as the return to isophony – but this time coordinated in definite pitch. As a result mesotonal music does not sound as bare as oligotonal. It supports induction of motif and cadence.

6) **Mutitonal Mode (12,000 BC).**^[[6]](#footnote-6)^ Addition of the VII degree produces a number of very important changes. It enables much greater variety of modal organization: as 6 equivalent steps, 3 equivalent 3^rds^ (trichord base), 2 equivalent 4^ths^ (tetrachord base) or a 5^th^ with a 3^rd^ (pentachord and trichord) - as well as their combinations. Of them, the tetrachord organization is the most common. The VII degree strengthens and enriches functionality of the V degree, by providing an equivalent 3^rd^ to the “dominant” tone, and the auxiliary tone to the VI degree. The VII degree can also promote complex homophony up to the 7^th^-chords and the formation of two alternating anchors for two 7^th^-chords on the odd and even degrees (Kubik 2005).

More common, nearly a standard, is the formation of the centripetal triadic functionality, comprised of two 3^rds^ sharing the same function. The axis of stability here falls on the odd I-III-V degrees, leaving the even II-IV-VI degrees to form the axis of instability, with an alternative triad of low VII-II-IV. All in all, multitonal mode promotes the emergence of chords and homophonic thinking. It also carries important melodic ramifications. It enables wide melodic leaps and compound intervals. Overall, increase of the ambitus, combined with the comb-like valence of degrees, compresses the increments between the degrees in a mode, directing the tonal organization towards heptatonic rather than pentatonic organization.

Stable/unstable dichotomy of odd/even degrees tends to increase distinctions between major and minor intervals and secure uniformity of intervals across complimentary degrees. This allows for expansion of ambitus over an octave, featuring limited octave-equivalence: I, II, III, and VII staying identical in tuning over an octave, whereas VI and V frequently engage octave inequivalence. Non-equivalent degrees are bound to form alternating gravitational centers. Gravitational mutability is the signature for multitonal mode: it can occur between the I and II degrees, the I and low VI degrees (most common), the I and IV degrees, and the I and V or I and III degrees. Such diversity seems to compensate for increase in centripetal tendency due to a more pronounced hierarchic organization and contrast between stable and unstable degrees.

A side-effect of mutability is the emergence of harmonic pulse: periodicity in switching between the central "tonic" and alternative "tonic." Such switches involve a slight shift in the tuning values of the unstable tones – to serve to a new "master" tone. Periodic re-tuning promotes memorization of a tuning table as a scheme, which lays the foundation for the genesis of functionality in vertical harmony. Not only musicians conceive harmonic intervals, they also conceive their functions of stability, instability, and neutrality.

7) **Pentatonic Mode (7,000 BC)**.^[[7]](#footnote-7)^ If multitonal mode tends to evolve into heptatonic, then mesotonal mode can develop in either multitonal or pentatonic mode. A culture often tries out both, and favors only one. Chinese culture tried out heptatonic organization, but preferred pentatony (Daniélou 1995, 33). Hebraic tradition seems to have discovered the pentatonic organization, but eventually abandoned it in favor of heptatony (Szabolcsi 1943). Sometimes a peculiar intermediate hexatonic mode is formed – which, however, did not evolve into a discrete intercultural type of tonal organization. Such mode usually leans either towards an expanded pentatonic or a shrunk multitonal mode. Of the two, pentatonic mode proves to be much more conservative – perhaps, the most conservative of all. Many existing cultures still adhere to pure pentatony. The landmark of such adherence is the dominance of horizontal aspect over vertical in tonal organization of the composition, where melodic processing occurs in trichords made up of the division of 4^th^ into two contrasting intervals: traceless “step” and tracing “gap.”

The reason why multitonal mode competes with pentatonic organization is that “gaps” require space, and that the more degrees are fit within the tonal space of a mode, the more likely it is for the increments between the degrees to compress. Once they are compressed, the “gaps” become impossible – instead, they turn into “composite steps” – which promotes thinking in terms of a single incremental standard, a “step” (minor, major, or neutral 2^nd^), rather than binary standard of 2^nd^ and 3^rd^. The peculiar pentatonic harmony is based on the systemic contrast between steps of different size, which distributes the tonal tension in such a way that it can never be consolidated, making pentatony into the most harmonious tonal scheme while retaining hierarchic organization and contrasts in ICs and PCs. In this way, pentatonic harmony has cognitive advantage over less hierarchic mesotonal systems that feature high harmonicity, such as equidistant pentachord and hexachord systems.

What separates pentatony from mesotonal modes is the strict octave equivalence and diatonic organization through the circle of 5^ths^. The IS is defined by the contrast of three 1-tone degrees and two 1^1^/_2_-tone degrees within an octave, and the strict exclusion of minor 2^nd^, major 7^th^, and tritone. As a result, all possible vertical intervals become relatively good-sounding – without the need for resolution rules typical for vertical harmony in multitonal and heptatonic modes. Well-distributed consonant and dissonant intervals across even and odd degrees of a pentatonic mode provide superb tonal blending that equalizes degrees in their stability/instability values, whereby effectively diffusing gravity. Pentatonic degrees display little attraction, easily excited by melodic impetus, allowing the melody to glide with strong inertia, usually building “terraces” across the entire melodic range. The unevenness of steps in the scale prevented the genesis of tension and elaboration of functionality, locking the music system, making it conservative to historic changes.

Some pentatonic cultures – i.e. Chinese – developed the concept of tonicity. However, in the absence of triadic organization in melody and harmony, the tonic function in pentatony remains weak and empirically questionable (until confirmed by experimental research). Instead of triadic harmony, pentatony relies on what can be called “quadratic” harmony, with 4^th^ being the most common interval for parallel vertical melodic lines in homophonic or polyphonic textures (Kubik 2005). Such intervallic harmony can easily produce 4^th^–chords. Pentatonal hierarchy is limited to only stable degrees (even then, only affording a simple 2-level hierarchy). Modality here presides over tonality: each mode is recognized by its specific ISC rather than a dedicated tonic, and affords transposition of PSC by interval.

8) **Heptatonic Mode (6,400 BC).**^[[8]](#footnote-8)^ Cultures that subscribed to the even/odd principle of tonal organization at earlier stages (i.e. oligotonal) usually end up bypassing the pentatonic stage and proceeding to heptatonic organization. The VII degree leads to discovery of octave equivalence as the formative modal principle in hemitonic mode: it exposes the VIII degree as the modal equivalent of the I degree. Then, the tetrachordal organization transforms into pentachordal, generating centripetal gravity toward a single “tonic,” often assigning the role of “dominant” to the V degree – replacing the mutability of I-IV degrees typical for tetrachordal organization.

The “over-degree” stable/unstable functionality of the pentachord I-V receives conjunct connection with the “enclosed” stable/unstable functionality of the tetrachord V-VIII. This tetrachord is perceived as the “inversion” of the I-V pentachord: the VIII degree is regarded as exact equivalent of the I degree, and the VI and VII degrees are regarded as equivalent in their instability status to the II and IV degrees. However, each of the degrees is categorized by its unique gradation of stability or instability in relation to the tonic. Tonic is used as the reference to the most stable tone, and the VII degree – to the most unstable tone. Heptatonic scheme presents the most pronounced tonicity with the clearest gravitational mapping.

Like pentatonic, heptatonic mode introduces strict octave equivalence and diatonicity of the circle of 5^ths^. However, its landmark is ternary division of the tetrachord into three perceptually similar intervals of a 2^nd^, where two of them are equal in size, while the third is slightly smaller. This difference in size makes the steps within the mode asymmetric, with the semitone acting as a marker in the modal structures – however, without creating the psychoacoustic contrast between the whole-tone degrees and semitone degrees. Both kinds of 2^nd^ retain their property of melodic consonance and harmonic dissonance. This qualitative uniformity sets heptatony apart from pentatony by enabling progressions of unstable degrees in melody, causing accumulation of tension. Prolongation of tension, and postponing its resolution, boosts radical development of harmony, elaborating devices of composition that feature fine gradations of tension. Heptatonic composition is likely to promote originality and innovation, leading to progressive increase of complexity in music.

Not only a rich IS becomes defined, but various chord-sets emerge, building typology of chords and fertilizing melodic harmony by establishing new pathways between chordal tones. Intervals and chords produce inversions and promote formation of basic function-classes: tonic, plagal, and authentic. Mode institutes a pentachord base and obtains major or minor inclination. Tonic-dominant relation serves as an axis for cadential organization within a music work. Class-like functionality, reproduced in different works, straightens out the octave discrepancies inherited from multitonal mode, greatly reducing modal mutability and increasing centripetal gravity.

Compared to the multitonal mode, heptatony introduces one extra level of centripetal hierarchy. Octave equivalence affords great expansion of the ambitus and rotation of tetrachords, promoting formation of new modes. The derivative modes can be combined in a composite mode, producing what may appear as chromaticism.

9) **Heptatonic MPS:** **Diatonic Polymodal System (2,200 BC).**^[[9]](#footnote-9)^ Practice of ensemble performance, accompanied by the rise of musical notation and math-driven prescriptive music theory marks a new phase in tonal development, during which music becomes professionalized. Instrumental technique, especially on string instruments, has the strongest formative influence on emergence of diatonic MPS. Names of strings in Mesopotamian and Greek cultures provided the names for tones in their mega-pitch-sets. Transposition of the same mode by degree, while retaining the names of the MPS tones instituted a new type of diatonicity – absolute diatonic relations within the MPS, where the “sameness” of the tone is as important as the position of the tonic for expressive tuning.

The resulting equalization in tuning of shared degrees between the sister-modes flattens hierarchic relations between the unstable degrees (as compared to the stand-alone heptatonic mode), thereby reducing centripetal gravity in every mode. Ease of modulation between the sister-modes further weakens each mode’s tonic. It is possible for modulations to engage alteration of a leading degree to emphasize the transition of the tonic, especially that the centripetal gravity in the mode is not very pronounced. In such case, a mode turns into a diatonic key – the evidence for which can be found in Babylonian tuning practice (Hagel 2005, 297).

On the other hand, the differences between the modes are more strongly marked. Each sister-mode inherits the very same stereotypical hierarchy from the parent mode (usually Mixolydian). The modal typology abides by the numerical degrees of the mode (i.e. VII degree executes functionality of the leading tone regardless of the actual interval between the VII and VIII degrees). The functional sameness of all the degrees across all sister-modes highlights different IC in every mode (i.e. in one mode the III degree is minor, whereas in another it is major), facilitating the comparison of modes. The I-V degrees functionality carries the most formative role in each of the sister-modes.

The position of a tritone becomes decisive in defining semantics of a mode: different inclinations (major, minor, or diminished) and different harmonicity of the ICS determine the assignment of a specific ethos to each of the modes. The MPS obtains a fixed set of semantic values, held in place by aesthetic emotions reinforced by religious/philosophical conventions. Temple/palace culture plays a central role in instituting such semantic system.

Formulation of the diatonic MPS music theory usually involves another flattening of the texture (similar to that during the transition from ekmelic to emmelic organization). Orchestral and/ or choral performance involve synchronization of multiple instruments/vocals, each utilizing its own manner of sound production, yet being held “in tune” according to a rationally defined music theory. This need is addressed by resorting to a monodic texture, where all the participants follow the same melodic model.

10) **Pentatonic MPS: Diatonic Polymodal System (c. 9 - 6^th^ century BC)**.^[[10]](#footnote-10)^ I haven’t had the opportunity to research this system in order to characterize how exactly it differs from simple octave equivalent pentatony that is found in many traditional folk cultures (i.e. Mongolian). However, the research on history of Chinese traditional music theory leaves no doubts that Chinese musical thought passed through the same stage of Cosmogonic theoretization as did its contemporary Mesopotamian and Greek music theories, bringing remarkably similar mathematic and astronomic theories (Goodman & Lien 2009), which were implemented in a decidedly pentatonic principles, applied to the entire chromatic gamut of the ambitus, made available by all known instruments (Daniélou 1995).

The circle of 5^ths^ was run 60 times according to the astrological constellations (McClain and Hung 1979) - this method of tuning was documented in Pere Amiot's treatise (1780) on Chinese music, where he translated excerpts from Prince Chu Tsai-yi's two works: Lu Hsueh Hsin Shuo (New Report about the Theory of the Twelve Semitones, 1584) and Lu Lu Ching I (Refined Interpretation of the Twelve Semitones, 1595 or 1596). Both works contained an overview of the historic development of tuning in Chinese music, and described the first attempt at the Pythagorean-style tuning system established by the 2^nd^ century BC (Kuttner 1975). All 60 pitches were standardized by reference to the common pitch standard, established by a set of specially manufactured bells (Falkenhausen 1992). The result of this well-tempered tuning was reproduction of the same set of legitimized pentatonic modes from all possible pitches (Bagley 2005) – a pentatonic diatonic^[[11]](#footnote-11)^ MPS.

Its realization seems to have come later than that of the heptatonic MPS – especially if to take into consideration that the indication for the use of mathematical theory to derive musical tuning in Mesopotamia comes about 800 years (Hilprecht 1906, 20:21) after the first archeological evidence of the use of a pitched instrument, harp (Lawergren 1997). The evidence of the first pitched instrument found on the territory of China is dated by the 7,000 BC – about 6,300 years before the mathematical theory was applied to tuning.

This slower development can be explained by the conservative nature of anhemitonic pentatony (Nikolsky 2015) that tends to stress stability and consonance in tonal organization, thereby discouraging accumulation of tension by compositional means and depriving musical tradition of a stimulus to achieve greater expressivity. There is therefore less need to establish a norm in tuning practices, since even imprecise pentatonic tuning still demonstrates reasonably high harmonicity. It appears that much of the effort to infer an optimal tuning was inspired by the idea to establish superiority of pentatonic over heptatonic tonal organization – with gradual waning of the heptatonic genres over the course of Chinese music history.

The Chinese MPS set the model for similar MPS systems developed in Korea, Japan, and Vietnam. The principal idea of their tonal organization seems to be pan-harmonization of all the available pitch space according to the metaphysical correspondence of cosmological, political, and personal physical states of equilibrium (Thrasher 1981). This music is characterized by strong contribution of ethos, engagement of aesthetic emotion, professionalized composition according to the codified principles and concept of authorship – quite similar to the diatonic MPS implementation in Babylon and Ancient Greece.

11) **Non-Octave Hypermode (500 BC)**.^[[12]](#footnote-12)^ In some cultures the diatonic equalization of the multitonal mode occurs without breaking into discrete sister-modes. The primary cause seems to be the prominence of the “chain principle” (Sachs 1960) in the melopoeia practice, where the modal kernel becomes reproduced above the kernel’s top note, whenever the singer needs an extra tone to be added to the ambitus. The expanded PS is treated compositionally as a single entity rather than modulation from one tetrachord to another. The idea of preserving the modal integrity must be underlying the genesis of the hypermode in place of the diatonic MPS. The association of modal integrity with choral religious chant characterizes the implementation of hypermode in Byzantine, Russian, Georgian, and Armenian cultures. Prevailing choral application is likely to have promoted fusion of the modal subsets (trichords or tetrachords) into a single hypermode – especially in polyphonic settings.

The fusing tendency is most evident in the overall avoidance of melodic dissonance: whether the hypermode is used in monody or polyphony, it commits to stepwise melodic motion, hardly using any leaps. Melodic inertia is the strongest in hypermode amongst all other forms of tonal organization. The idea of preserving “diatonic” relations between the neighboring degrees forms the hypermodal composition, typically creating false relation in the marginal registers, so that they would not meet to sound simultaneously.

Monodic application succeeds in projecting a strict diatonic impression, without any alterations or modulations. Polyphonic applications are more revealing of the false relations that usually involve 1-2 tones. Harmonically, this produces contrast between the diatonic natural and the augmented/diminished intervals. Augmented intervals mark the overall centrifugal gravity, whereas diminished intervals – centripetal gravity. However, this gravity can be substantially diffused when each subset uses its own anchor tone. In particular, polyphonic applications can distribute tonicity very smoothly between the parts, forming amazing variety of chords, including 4^th^–chords and clusters.

False relations produce an “elastic effect”: whenever the parts gradually ascend, they smoothly increase tension, whereas whenever they descend, they increase relaxation. Music users often describe this in terms of lightening and darkening. It is through this effect that hypermode becomes the first tonal system to decidedly turn around from tonal characteristics of verbal speech where the traditional association of high register with friendly disposition opposes the association of low register with aggressive disposition (Ohala 2006).

Subsets determine organization of vertical harmony, resulting in replacement of octave equivalence with equivalence of 4^th^, or mixture of subsets, some of which are 4^th^ or 5^th^ equivalent, and some are contrasting. Therefore, hypermode does not afford an octave-bound interval inversion. To add to the confusion, false relation can be transposed from a higher register to a lower register, should the singer have difficulty singing too high. In such situation the hypermode can exhibit “shimmering” degrees (Rudneva 1994, 150) – where both tones that form a false relation are found next to each other in the same register. This “shimmering” is not meant to be a chromatic modification of a degree, but constitutes a very special case of modal inflection.

Hypermodes can bear major, minor, and diminished inclinations, realized through conjunct or disjunct joining of the modal subsets. The differences between sister-hypermodes are much less obvious than those between the MPS sister-modes - because of greater modal complexity (up to 18 degrees) and flatter hierarchy of instability (2 levels only).

12) **Chromatic Polymodal System (500 BC)**. The principal idea behind the chromatic tonal organization is finding extra means of marking instability in order to enable expression of greater intensity of tension and finer gradations of it. Dissatisfied with “plainness” of diatonic multimodal system, musicians sought out ways to intensify tonal development. Since “stability” could not be increased in a centripetal hierarchic mode, their inventiveness went in the direction of increasing instability in an incremental manner. The solution was found in altering the gap between the degrees in one place in a tetrachord, and clustering the degrees in another place. Such alteration was achieved by nudging an unstable degree, while keeping the stable degree in place. An altered unstable degree becomes more unstable than a regular unstable degree.

From this, emerges the idea of chromatic and enharmonic types of alteration. They were not very well distinguished from each other throughout Antiquity, forming an assortment of tonal “shades” of various microtuning. Darkening or lightening of shade came to diversify the tonal gamut rather than adding discrete new tonal colors by introduction of new degrees or new modes.

To measure microtonal modifications, music theory had to standardize a smaller incremental unit. Polymodal chromatic system became an open-end mixture of tetrachords of different intervallic types, achieved by conjunct stitching of modal subset units – in a manner similar to non-octave hypermode. However, the chromatic system pursues completely opposite goals – maximizing tension in order to achieve dramatic effects. That is why the development of the chromatic system concurs with the flourish of aesthetic emotion, expressed to the fullest in the rise of theatric arts (tragedy or dithyramb).

Chromatic music becomes professional, and completely splits apart from amateur music, producing tradition of passive music listening and aesthetic appreciation. Here, the pivotal role is played by the notion of chromatic alteration – an accidental modification of the normative value of the PC, done with the purpose of inducing greater tension in order to direct the melodic motion towards a new tonal anchor.

Awareness of the conflict between the normative value and its chromatic alteration closely corresponds to awareness of the fictional character of the emotional state represented by music as opposed to the “real” emotional state. The listener and the performer of chromatic music develop a habit of estranging from the expression of music, taking it as an aesthetic object rather than a direct act of communication on the part of the performer, who is “reporting” about his actual emotional state.

Growing complexity of chromatic music induces greater subordination within chromatic subsets, while maintaining coordinative connections between its PS members, producing different hierarchic organization in every subset and every mode of the polymodal system – with amazing versatility of options (including modal mutability). The overall gravity presents a compromise between diatonic MPS and non-octave hypermode: gravity is distributed amongst 2-4 anchors, each of which can be strongly stressed by chromatic alterations. This system affords, both, transposition by degree and by interval of a PS.

Chromatic music produced a new type of texture – functional heterophony – that differed from earlier forms of heterophony, by sustaining each of the parts in a certain textural function (i.e. drone or dub). Therefore, functional heterophony presented a more complex texture than the folk heterophony. Chromatic shading played an important role in shaping such a texture: being applied to the vocal part, it made it tonally different from a more diatonic instrumental accompaniment. Once formed, functional heterophony was used in the diatonic MPS music of the time.

Chromatic music started to oppose diatonic music by forming its own ethos, and associating it with specific genres. Market consumption, music contests, public festivities, personal use, and private social gatherings, constituted the main domains for chromatic music use in an Ancient society. After about the 1^st^ century AD the chromatic music ceded its development in the West, but found a new soil in the East – eventually reincarnating in the plainchant of Eastern Orthodox churches – when exactly, it is hard to tell.

However, the Chrysanthos reform of the Greek Orthodox church indisputably restored the chromatic and enharmonic genera of the Ancient Greeks in the music theory of plainchant (Barsky 2014, 61), albeit with some modifications (Chrysanthos and Rōmanou 1973, 105–108), under the aegis of purification of modal principles and their protection from the influence of Western tonality (Lind 2012, 68). According to this policy, the chromatic plainchant that was probably already widely used in Christian liturgy on the territory of Greece, Turkey, Romania, Bulgaria, Macedonia, and Serbia became reserved for the II and VI echoi of the oktoechos, while its enharmonic variety was reserved for the III and VII echoi (Scurtu and Tutu 2011).

13) **Hemiolic mode (c.11 – 18^th^ century)**.^[[13]](#footnote-13)^ Ancient Greek Chromatic music spread throughout the Mediterranean, Near East, Central Asian, and Indian regions, where it survived during the Middle Ages, when Christianity took a negative position towards chromatic music. Sometime, in parallel to Western classical tradition that was elaborating its own chromatic music (ficta), the Ancient chromatic modality transformed into a more tense and modally flexible framework of hemiolic mode. It is difficult to establish whether it evolved directly from the Ancient Greek sources, or it was transformed through passing on the Greek heritage to the local cultures of the Near East and Central Asia, or that perhaps it originated from the older Hebraic tradition, or that it was absorbed from Balkan folk cultures. Hemiolic typology was definitely nourished by the Arabic, Persian, and Turkish traditional art music, functional in maqam - at least from the 13^th^ century. Hemiolic gap is chiefly responsible for providing a “musical face” to the idea of “oriental music” that served to provide an identity for numerous cultures of the Mediterranean region: Spain, countries of Maghreb, Levant, Greece, Romania, Croatia, Serbia, Turkey, as well as Azerbaijan - and the Central Asia region: Iran, Afghanistan, Uzbekistan, Tadjikistan, and Pakistan. The Jewish and Gypsy traditions have distributed hemiolic music internationally.

Hemiolic mode seems to easily pair with one of the parallel diatonic “dominant modes” (see Appendix IV) in a way similar to how relative major and minor keys pair in Western tonality. The division of dominant modes into hemiolic and diatonic also resembles the Ancient Greek distinction between chromatic/enharmonic (which in practice, were poorly distinguished from each other) and diatonic genera. The “dominant keys” of the flamenco music provide the most obvious functionality of the key because of the strong involvement of instrumental accompaniment and usage of chords. It is quite possible to regard the *dominant key* as the heir of the combined chromatic and enharmonic genera of the Ancient Greeks.

Hemiolic modes initially featured a strong microtonal component (Marcus 1993), but from the 19^th^ century on they became influenced by Western tonality through the practice of large instrumental ensemble performance. Extensive use of chords, emulating Western orchestral textures in the arrangement of traditional tunes, eventually stripped hemiolic modes off of much of microtonal inflections. Today only some samples of folk hemiolic music feature explicit microtonal modal organization (Katsanevaki 2011). Otherwise, microtonal adjustments retain their place mostly in the non-chordal implementations of hemiolic modes, such as maqam.

At a given moment, it is hardly possible to conclude with certainty whether hemiolic organization is progressing towards its own unique form of handling the chromatic organization – alternative to the path taken by Western tonality, which embeds the chromatic alphabet in the concept of a tonal key – or, whether hemiolic scheme is in the process of merging with Western tonality. The music theory of the hemiolic organization has to be outlined and verified in relation to numerous music cultures that use the gapped modes. The relationship between the hemiolic and diatonic organization has to be defined for all respective cultures. Only then will it be possible to judge where exactly the hemiolic music stands in relation to Western tonality.

The conglomerate of the modes used by the flamenco, maqam, dastgah, and local folk traditions of the Mediterranean area, seems to bind into a rather uniformed method of harmonic organization – sometime by the late 18^th^ century amalgamated into what can be called the “Mediterranean tonality” (Manuel 1989). The diatonic and hemiolic modes of the “dominant key” that characterizes this tonality, at present, do oppose the tonal major/minor keys of Western classical music in their tonal hierarchic organization, their typology of textures, and prevalent compositional techniques. The aesthetics of music, created in “Mediterranean tonality,” provides perhaps the greatest testimony towards holding it as the music system that is alternative and even opposing the aesthetics of Western classical music (see Appendix IV).

If that is the case, then we might witness a new, third divergence, in evolution of tonal organization. The *first divergence* produced the opposition of heptatonic and anhemitonic pentatonic cultures, most pronounced in the antagonism of music philosophies of the West and of the Far East: progressivism and constructiveness versus conservatism and contemplation.^[[14]](#footnote-14)^

The *second divergence* originated in the 5^th^ century BC Athens in confrontation of chromatic and diatonic aesthetics of music – resurfaced again during Early Middle Ages along the lines of incompatibility of the Christian values with the pagan values. This divergence generated hypermodal organization as a strictly regulated quasi-diatonic alternative to chromatic music (see Appendix II), which determined the development of the Christian plainchant, and influenced polyphonic forms of music in the Eastern European cultures.

It could be that the distinction between Mediterranean and Western tonalities marks the growing *third divergence* between the Islamic and Western cultural values. Christian ideology definitely played a substantial role in promoting the integrative power of the Western tonality and polyphonic/homophonic texture in reincarnating the theatric illusionistic philosophy of the Ancient Greeks and Romans, and elevating it to new spiritual heights. On the other hand, Islamic ideology, as well as iconoclastic tendencies that have remained alive from the Byzantine times in the Eastern Orthodox aesthetics, have proved to be as formative in nurturing the ornamental modular style of compositional arrangement, devoid of theatricality and seeking more direct, “indexical” connection (rather than iconic imitation of perceptual reality). In relation to music, anti-theatric attitude means setting the goal of the music composition to attain a single musical emotion and to stay at the height of its artistic rendition to provide the audience with the ecstatic experience – as opposed to more dramatic expression that characterizes Western music, where music composition usually presents an “emotional story.”

Both ideologies appear to follow the lines of Pythagorean/Aristoxenian dichotomy of diatonic and chromatic musics: the Islamic and Eastern Orthodox traditions still observe the philosophy of the “music of the spheres,” where human passions are supposed to be regulated by musical alignment with celestial “rhythms.” In contrary, Western classical music tradition inherited the theatrical manner or making some kind of point through the composition of music in order to impress the listener. Both ideologies could have spontaneously engaged their own form of “tonality” to rally their supporters by emotive means of music.

REFERENCES:

Ackermann, Hermann, Steffen R. Hage, and Wolfram Ziegler. 2014. “Brain Mechanisms of Acoustic Communication in Humans and Nonhuman Primates: An Evolutionary Perspective.” *The Behavioral and Brain Sciences* 37 (6). Cambridge University Press: 529–46. doi:10.1017/S0140525X13003099.

Alekseyev, Eduard. 1976. *Problems in Genesis of Mode [Проблемы формирования лада]*. Moscow: Muzyka [Музыка].

Atema, Jelle. 2014. “Musical Origins and the Stone Age Evolution of Flutes.” *Acoustics Today* 10 (3): 25–34.

Bagadurov, Vsevolod A. 1953. *Vocal Training of Children [Вокальное воспитание детей]*. Moscow: Academy of Pedagogical Sciences of Russia.

Bagley, Robert. 2005. “The Prehistory of Chinese Music History.” In *Proceedings of the British Academy*, edited by P.J. Marshall, 131:41–90. Oxford, UK: OUP/British Academy. doi:10.5871/bacad/9780197263242.001.0001.

Barsky, Vladimir. 2014. *Chromaticism*. London: Routledge.

Bednarik, Robert. 2014. “Pleistocene Paleoart of Europe.” *Arts* 3 (Bednarik 2013): 245–78. doi:10.3390/arts3020245.

Bogdanov, V.A. 2007. “Prehistory of Ukranian Musical Art of Wind Instruments [Предыстория духового музыкального искусства Украины].” *Bulletin of the Kharkiv State Academy of Design and Arts: Art.* 12 (2): 11–20.

Bogomilsky, M.R., and V.R. Chistiakova. 2008. *Diseases of Ear, Throat and Nose in Children Age: The National Guide [Болезни Уха, Горла, Носа В Детском Возрасте: Национальное Руководство]*. Edited by Yu. Radzig. Moscow: GEOTAR-Media [ГЭОТАР-Медиа].

Brown, Steven. 2007. “Contagious Heterophony: A New Theory about the Origins of Music.” *Musicae Scientiae* 11 (1): 3–26. doi:10.1177/102986490701100101.

Chou, Wen-Chung. 1976. “Chinese Historiography and Music: Some Observations.” *Musical Quarterly* 62 (2): 218–40. doi:10.1093/mq/LXII.2.218.

Chrysanthos, and Kaitē Rōmanou. 1973. *Great Theory of Music by Chrysanthos of Madytos*. Translated by Kaitē Rōmanou. Bloomington, IN: Indiana University.

Conard, Nicholas J, Maria Malina, and Susanne Münzel. 2009. “New Flutes Document the Earliest Musical Tradition in Southwestern Germany.” *Nature* 460 (7256): 737–40. doi:10.1038/nature08169.

D’Errico, Francesco, Christopher Henshilwood, Graeme Lawson, Marian Vanhaeren, Anne-Marie Tillier, Marie Soressi, Frédérique Bresson, et al. 2003. “Archaeological Evidence for the Emergence of Language, Symbolism, and Music — An Alternative Multidisciplinary Perspective.” *Journal of World Prehistory* 17 (1): 1–70.

Daniélou, Alain. 1995. *Music and the Power of Sound: The Influence of Tuning and Interval on Consciousness*. Rep Sub ed. Rochester, Vt: Inner Traditions.

Dissanayake, Ellen. 2013. “Born to Artify: The Universal Origin of Picturing.” In *Origins of Pictures: Anthropological Discourses in Image Science*, edited by Klaus Sachs-Hombach and Jorg Schirra, 230–49. Koln, Germany: Herbert von Halem Verlag.

Dmitriyev, Leonid B. 1968. *The Foundations of Vocal Methodology [Основы вокальной методики]*. Moscow: Muzyka [Музыка].

Dumbrill, Richard. 2007. “The Earliest Evidence of Heptatonism in a Late Old Babylonian Text: CBS 1766.” *The Archaeomusicological Review of the Ancient Near East* 30 (2): 43–45.

Eerola, Jan. 2009. “Examination of Stylistic Traits in Sound Production of the Veps Lühüd Pajo Songs Using Computer-Aided Music Analysis.” In *Perspectives on the Song of the Indigenous Peoples of Northern Eurasia: Performance, Genres, Musical Syntax, Sound*, edited by Jarkko Niemi, 160–97. Tampere, Finland: Tampere University Press.

Falkenhausen, Lothar von. 1992. “On the Early Development of Chinese Musical Theory: The Rise of Pitch-Standards.” *Journal of the American Oriental Society* 112 (3): 433–39. doi:10.2307/603079.

Fancourt, Amy, Frederic Dick, and Lauren Stewart. 2013. “Pitch-Change Detection and Pitch-Direction Discrimination in Children.” *Psychomusicology: Music, Mind, and Brain* 23 (2): 73–81. doi:10.1037/a0033301.

Friberg, Joran. 2011. “Seven-Sided Star Figures and Tuning Algorithms in Mesopotamian, Greek, and Islamic Texts.” *Archiv Für Orientforschung* 52: 121–55.

Goodman, Howard, and Edmund Lien. 2009. “A Third Century AD Chinese System of Di-Flute Temperament: Matching Ancient Pitch-Standards and Confronting Modal Practice.” *The Galpin Society Journal* 62: 3–24.

Grachiova, Magdalina S. 1971. “Human Vocal Fold [Голосовая складка человека].” In *Development of Child’s Voice [Развитие детского голоса]*, edited by Valentina Shatzkaya, 1:20–28. Moscow: Academy of Pedagogical Sciences of Russia.

Hagel, Stefan. 2005. “Is Nîd Qabli Dorian? Tuning and Modality in Greek and Hurrian Music.” *Baghdader Mitteilungen* 36: 287–348. http://cat.inist.fr/?aModele=afficheN&cpsidt=17961761.

———. 2009. *Ancient Greek Music: A New Technical History*. New York: Cambridge University Press.

Hilprecht, Hermann. 1906. *The Babylonian Expedition of the University of Philadelphia*. Vol. 20, part 1. Philadelphia: University of Pennsylvania, the Department of Archaeology.

Huron, David. 2006. *Sweet Anticipation: Music and the Psychology of Expectation*. Cambridge, MA: MIT Press.

Huyge, Dirk. 1990. “Mousterian Skiffle? Note on a Middle Palaeolithic Engraved Bone from Schulen, Belgium.” *Rock Art Research* 7: 125–32.

Katsanevaki, Athena N. 2011. “Chromaticism: A Theoretical Construction or a Practical Transformation?” *Muzikologija: Casopis Muzikoloskog Instituta Srpske Akademije Nauka I Umetnosti* 11: 159–80. doi:10.2298/MUZ1111159K.

Kubik, Gerhard. 2005. “The African Matrix in Jazz Harmonic Practices.” *Black Music Research Journal* 25 (1/2): 167–222. http://www.jstor.org/stable/30039290.

Kuttner, Fritz A. 1965. “A Musicological Interpretation of the Twelve Lüs in China’s Traditional Tone System.” *Ethnomusicology* 9 (1): 22–38.

———. 1975. “The 749-Temperament of Huai Nan Tzu (+ 123 B. C.).” *Asian Music* 6 (1): 88–112.

Lawergren, Bo. 1997. “Mesopotamia, Musical Instruments.” Edited by Ludwig Finscher. *Die Musik in Geschichte Und Gegenwart: Allgemeine Enzyklopädie Der Musik*. Kassel, Germany: Bärenreiter and Metzler.

Leblon, Bernard. 2003. *Gypsies and Flamenco: The Emergence of the Art of Flamenco in Andalusia*. Hatfield, UK: University of Hertfordshire Press.

Legge, James. 1872. *The Chinese Classics: With a Translation, Critical and Exegetical Notes, Prolegomena, and Copious Indexes. Dukes Seang, Ch’aon, Ting, and Gae, with Tso's Appendix and the Indexes*. Hong Kong: Lane, Crawford and Co. https://books.google.com/books?id=srBUAAAAcAAJ.

Levin, Theodore Craig, and Valentina Süzükei. 2006. *Where Rivers and Mountains Sing: Sound, Music, and Nomadism in Tuva and Beyond, Volume 1*. Bloomington, IN: Indiana University Press. https://books.google.com/books?id=a8B02Dl4_6cC&pgis=1.

Lind, Tore Tvarnø. 2012. *The Past Is Always Present: The Revival of the Byzantine Musical Tradition at Mount Athos*. Lanham, MD: Scarecrow Press. https://books.google.com/books?id=VPWjSeLibr4C.

Louhivuori, Aino. 2006. “Tonal Development of a Child’s Song Improvisations: A Case Study.” In *The Proceedings of The First European Conference on Developmental Psychology of Music, 17-19 November 2005, University of Jyväskylä, Finland*, edited by Pirkko Paananen and Maija Fredrikson, 287–90. Jyväskylä, Finland: University of Jyväskylä.

Makris, Eustathios. 2005. “The Chromatic Scales of the Deuteros Modes in Theory and Practice.” *Plainsong and Medieval Music* 14 (01). doi:10.1017/S0961137104000075.

Manuel, Peter. 1989. “Modal Harmony in Andalusian, Eastern European, and Turkish Syncretic Musics.” *Yearbook for Traditional Music* 21: 70–94. doi:10.2307/767769.

Marcus, Scott. 1993. “The Interface between Theory and Practice: Intonation in Arab Music.” *Asian Music* 24 (2): 39–58.

Marshack, Alexander. 1996. “A Middle Paleolithic Symbolic Composition From the Golan Heights: The Earliest Known Depictive Image.” *Current Anthropology* 37 (2): 357. doi:10.1086/204499.

Mazepus, Vladimir. 2009. “Analysis of Timbres in Ethnomusicology: The Articulatory Tension and Its Acoustical Correlates.” In *Perspectives on the Song of the Indigenous Peoples of Northern Eurasia: Performance, Genres, Musical Syntax, Sound*, edited by Jarkko Niemi, 198–209. Tampere, Finland: Tampere University Press.

McAllister, Anita, Elisabeth Sederholm, and Johan Sundberg. 1993. “Acoustic and Perceptual Analysis of Vocal Registers in Children.” *Music and Hearing Quarterly Progress and Status Report* 34 (4): 29–34. http://www.speech.kth.se/prod/publications/files/qpsr/1993/1993_34_4_029-034.pdf.

McClain, Ernest G., and Ming Shui Hung. 1979. “Chinese Cyclic Tunings in Late Antiquity.” *Ethnomusicology* 23 (2): 205. doi:10.2307/851462.

McKernon, Patricia E. 1979. “The Development of First Songs in Young Children.” *New Directions for Child and Adolescent Development*, no. 3: 43–58. doi:10.1002/cd.23219790306.

Merker, Björn. 2012. “Vocal Learning Constellation.” In *Music, Language, and Human Evolution*, edited by Nicholas Bannan, 215–62. Oxford, UK: Oxford University Press. doi:10.1093/acprof:osobl/9780199227341.003.0009.

Miller, Donald Gray. 2000. “Registers in Singing: Empirical and Systematic Studies in the Theory of the Singing Voice.” Groningen, The Netherlands: Rijksuniversiteit Groningen. http://irs.ub.rug.nl/ppn/19458396.

Morley, Iain. 2013. *The Prehistory of Music: Human Evolution, Archaeology, and the Origins of Musicality*. Oxford: Oxford University Press.

Morozov, Vladimir P. 1977. *Biophysical Foundations of Vocal Expression [Биофизические основы вокальной речи]*. Leningrad: Nauka.

Münzel, Susanne, Friedrich Seeberger, and Wulf Hein. 2002. “The Geißenklösterle Flute: Discovery, Experiments, Reconstruction.” In *Archäologie Früher Klangerzeugung Und Tonordnung; Musikarchäologie in Der Ägäis Und Anatolien*, 10:107–18. Rahden, Germany: M. Leidorf.

Neubaer, E. 1992. “Music in the Islamic Environment.” In *History of Civilizations of Central Asia*, edited by Clifford Edmund Bosworth and Asimov, 4:2:712. Motilal Banarsidass Publ.

Neugebauer-Maresch, Christine, Margit Bachner, and Johannes Tuzar. 2008. “Kammern-Grubgraben.” *Wissenschaftliche Mitteilungen Aus Dem Niederösterreichischen Landesmuseum* 19: 119–28.

Nikolsky, Aleksey. 2015. “Evolution of Tonal Organization in Music Mirrors Symbolic Representation of Perceptual Reality. Part-1: Prehistoric.” *Frontiers in Psychology* 6 (1405). doi:http://dx.doi.org/10.3389/fpsyg.2015.01405.

Ohala, John J. 2006. “The Frequency Code Underlies the Sound-Symbolic Use of Voice Pitch.” In *Sound Symbolism*, edited by Leanne Hinton, Johanna Nichols, and John J. Ohala, 325–47. Cambridge, UK: Cambridge University Press.

Oniani, Ekaterine. 2010. “On the Polyphony of Georgian Chant.” In *Proceedings: The Fifth International Symposium on Traditional Polyphony: 4–8 October, 2010, Tbilisi, Georgia*, edited by Rusudan; Tsurtsumia and Joseph Jordania, p. 381–86. Tbilisi, Georgia: Tbilisi State Conservatoire.

Pohlmann, Egert, and Martin L. West. 2001. *Documents of Ancient Greek Music: The Extant Melodies and Fragments*. Oxford: Oxford University Press.

Radynova, Olga, Albina Katinene, and Marine Palavandishvili. 1994. *Musical Upbringing of Preschoolers [Mузыкальное воспитание дошкольников]*. Edited by Olga Radynova. Moscow: Prosvesheniye.

Robson, Eleanor. 2003. “Tables and Tabular Formatting in Sumer, Babylonia, and Assyria 2500 BCE- 50 CE.” In *The History of Mathematical Tables: From Sumer to Spreadsheets*, edited by Martin Campbell-Kelly, Mary Croarken, Raymond Flood, and Eleanor Robson, 18–47. Oxford, UK: Oxford University Press.

Rowan, Diana. 2013. “The Universal Lyre: Three Perspectives.” *American Harp Journal* 24 (1): 55–63. EBSCO accession # 89761478.

Rudneva, Anna. 1994. *Russian Traditional Musical Works: Essays on the Theory of Folklore [Русское народное музыкальное творчество: очерки по теории фольклора]*. Moscow: Kompozitor [Композитор].

Sachs, Curt. 1960. “Primitive and Medieval Music: A Parallel.” *Journal of the American Musicological Society* 13 (1/3): 43–49.

Scurtu, Bogdan, and C. Tutu. 2011. “Romanian Ochtoechoi and Similarities to Middle Eastern Modes and Practices: A Case Study (part I).” *Scientific Bulletin of the Transilvania University of Brasov* 4 (53/2): 89–98.

Seeger, Anthony. 2004. *Why Suyá Sing: A Musical Anthropology of an Amazonian People*. Chicago, IL: University of Illinois Press.

Sicoli, Mark A. 2015. “Voice Registers.” In *The Handbook of Discourse Analysis*, edited by Deborah Tannen, Heidi E. Hamilton, and Deborah Schiffrin, 105–26. Chichester, UK: John Wiley & Sons.

Sillitoe, Paul, and Karen Hardy. 2003. “Living Lithics: Ethnoarchaeology in Highland Papua New Guinea.” *Antiquity*.

Stulova, Galina. 1992. *The Development of Child’s Voice in the Process of Teaching Singing [Развитие детского голоса в процессе обучения пению]*. Moscow: Prometei.

Sundberg, Johan. 1987. *The Science of the Singing Voice*. DeKalb, IL: Northern Illinois University Press.

Szabolcsi, Bence. 1943. “Five-Tone Scales and Civilization.” *Acta Musicologica* 15 (1/4): 24–34.

Thrasher, Alan R. 1981. “The Sociology of Chinese Music: An Introduction.” *Asian Music* 12 (2): 17–53. doi:10.2307/834055.

Wurz, Sarah. 2010. “Interpreting the Fossil Evidence for the Evolutionary Origins of Music.” *Southern African Humanities* 21 (2005): 395–417.

Yakovlev, A.V. 1958. “On Physiological Foundation of the Genesis of a Singing Voice [О физиологических основах формирования певческого голоса].” In *The Matters of Vocal Education of School Children: In Assistance to the School Teacher of Singing [Вопросы певческого воспитания школьников: В помощь школьному учителю пения]*, 7–13. Leningrad: Uchpedgiz.

Zhang, Juzhong, Xinghua Xiao, and Yun Kuen Lee. 2004. “The Early Development of Music. Analysis of the Jiahu Bone Flutes.” *Antiquity* 302 (78, April): 769–78.

Zubrow, Ezra B.W., and Elizabeth C. Blake. 2006. “The Origin of Music and Rhythm.” In *Archaeoacoustics*, edited by Christopher Scarre and Graeme Lawson, 142. Cambridge, UK: McDonald Institute for Archaeological Research.

1. The dates for periodization are grand approximations, and should be taken as a mere reference for rough correspondence between the ethnomusicological characteristics found in existing folk cultures and the known prehistoric cultures. I date the first known (or hypothetically indicated) occurrence of a given scheme to substantially vary in time, whereby, in reality, different cultures can “discover” this or that scheme much later. Pre-mode is dated by the origin of Homo heidelbergensis, whose larynx was capable of sustaining pitch for a long time without dropping it (Wurz 2010). The area in the brain responsible for muscular control of the larynx is involved in the tasks of discrimination in pitch between different melodies, indicating that vocal capacity to precisely control the pitch, and hearing capacity to match timing in vocal contours, together, determine the genesis of music (Brown 2007). And it is this combination that constitutes what Ackermann et al. (2014) called “communicative musicality,” which seems to be the biological component of musicality. [↑](#footnote-ref-1)
2. Khasmatonal mode is dated by the beginning of the production of the first artifacts, such as marked or colored stones, that suggest the development of the ability to manufacture “extra-ordinary” things – to which class, sonic effects of melodic shaping and timbration belong (Dissanayake 2013). The outcome of khasmatonal culture must have been the production of discrete contour lines - the earliest example of which is the Quneitra artifact from the Levant, 55,000 BP (Marshack 1996). [↑](#footnote-ref-2)
3. Ekmelic mode is dated by the very end of Mousterian period, when scrapers with incised lines, zigzag engravings, or highly polished areas started being produced (Bednarik 2014). Some of such scrapers could be used as music instruments: rasping (Huyge 1990) or flint-knapping (Zubrow & Blake 2006) – in a way similar to forms of music-making observed today (Sillitoe & Hardy 2003). Production of marks on tools testifies that their makers were considering certain objects as special, and intended a specific function in mind. In a similar vein, manipulating one’s voice in order to reach a specific register requires understanding of singing as a special form of vocalization, and intending a particular genre application for it. Furthermore, rasping and knapping expose metric properties of regularity and proportionality. The idea of breaking the vocal compass into pitch zones correlated by size and order, and creation of melodies characterized by ongoing oscillation, by a sinusoid curve, is very similar to the idea of rhythmisized rasping/knapping. Both activities involve regular repetition: rhythm in the medium of time, melody in the medium of pitch. Such repetition serves to explore and order its medium, developing a peculiar type of coordination. [↑](#footnote-ref-3)
4. Oligotonal mode is dated by the first bone “flutes” whose design featured contrast between intervals of 2^nd^ and 3^rd^ in the positioning of holes on the pipe: the Geißenklösterle-1 flute (Münzel et al. 2002). Gravettian 4-hole 83888(a) and 3-hole 86757(a) feature equidistant design (D'Errico et al. 2003). Categorization of tuning of a pipe by an interval of certain size testifies towards assigning a fixed pitch value at least to one tone (from which the estimation of an intervallic increment is made). The very fact of manufacturing of over 120 flutes dated 36,000 to 30,000 years ago (Conard et al. 2009) – that feature 2-5 holes (Morley 2013, 32–98) and came to replace earlier whistles – indicate oligotonal culture. [↑](#footnote-ref-4)
5. Mesotonal mode is dated by the appearance of the first flutes where the holes were positioned so that the tones they produced formed a triad – the Grubgraben flute (Neugebauer-Maresch, Bachner, & Tuzar 2008). Tuning of an instrument to the triad testifies to the modal importance of the triad, indicative of the triad induction that shapes the pentachord functionality. [↑](#footnote-ref-5)
6. Multitonal mode is dated by the appearance of the 6- and 9-hole horns found in Molodova, Ukraine (Bogdanov 2007) – their complexity should be sufficient to produce a multitonal scale. There is no point of making that many holes on a single instrument unless all the tones produced by these holes are engaged at least in some important music works. The first proof of octave equivalence which distinguishes the multitonal organization from mesotonal, comes from the M282:20 and M282:21 Jiahu flutes, 6,400 BC (Zhang, Xiao & Lee 2004). [↑](#footnote-ref-6)
7. The term “pentatonic” here is used exclusively in relation to the anhemitonic pentatonic – regarding it as a special method of tonal organization, alternative to heptatonic organization (hemitonic pentatony occupies a position closer to heptatony). Emergence of pentatonic organization is dated by the time of the first generation Jiahu flute, M341:2, Wuyang, China – that features clear anhemitonic pentatonic design with perfect octave equivalence. [↑](#footnote-ref-7)
8. Heptatonic mode is dated by the time of the second generation Jiahu flute M282:20. Although this flute proves only the hemitonic design, nevertheless, it strongly suggests heptatonic tuning (see Appendix II for Part-1). Either by combined fingering, or by varying the pressure of the lips, or by production of harmonics, a Jiahu flutist was likely to generate the G tone that lacked a dedicated hole – especially, since its contemporary, the M282:21 flute, features a chromatic hexachord. Next in time-line stands the Veyreau flute. Its tuning implies the presence of a degree on G#, although it does not reserve a dedicated hole for it (Atema 2014). This flute was most likely tonicizing E, and its 7^th^ tone, G#, would have served as the “tonic” 3^rd^ – a degree that can hardly be missed in a mode. [↑](#footnote-ref-8)
9. The first documented heptatonic MPS system comes from the Old Babylonian tablet CBS 1766 that postulated tuning of the heptatonic mode within the family of modes (Dumbrill 2007). Although Dumbrill dates it by the 15^th^ century BC, other researchers consider this tablet conforming to the older standards of mathematical reference tablets, characteristic to Larsa, 18^th^ century BC (Friberg 2011), as established by Robson (Robson 2003). Hilprecht (Hilprecht 1906, 20:21) offers evidence of such tables as early as 2,200 BC. It is very possible that the origin of heptatonic tuning goes back even earlier, preserved through some oral tradition, at least to the 26^th^ century, when the Lyre of Ur was made (Rowan 2013). [↑](#footnote-ref-9)
10. Rise of the pentatonic MPS system is indisputably dated by the time of manufacturing the Zeng Hou Yi bells, post 433 BC (Falkenhausen 1992), most likely came into being much earlier – unfortunately, the Chinese references to the origin of the learned theory of pentatonic system (attributed to the reign of Huang Ti, 26^th^ century BC) are compromised by multiple attempts to rewrite history by various emperors during the Late Antiquity and Middle Ages. More substantial is the fact of assigning unique names to each of the 5 pitches in the 9^th^ century BC (Kuttner 1965), indicative of complete octave equivalence, but falling short to prove the existence of scalar transpositions. The anecdotal references to Te Shao’s musical compositions and their high esteem by Confucius, who put in place philosophy quite similar to Pythagorean, suggest that by the 6^th^ century BC the “cosmological” music theory already existed (Chou 1976). The description of the consultation of the Marquis of Jin by the physician He in 541 BC leads to confirm this conclusion, as He speaks of rules of music composition and “five regular intervals” as the model of cosmic order, civic order, and physical health (Legge 1872, 580). The first formulation of the pentatonic scale based on intervallic subtraction and addition is stated in Kuan Tzu, attributed to Kuan Chung, 7^th^ century BC (Chou 1976). [↑](#footnote-ref-10)
11. The term “diatonic” can be applied to the pentatonic system, because its tones are arithmetically “distributed” (this is what the term “diatonos” implies) by means of tuning each tone according to the circle of 5^ths^. [↑](#footnote-ref-11)
12. Non-octave and chromatic systems correspond to Ancient Greek Smaller and Greater systems, formulated by the time of Aristoxenus, but most likely existed earlier (Hagel 2009, 6). Non-octave hypermode is organically related to the partially non-octave implementation of expressive tuning in multitonal modes of folk prehistoric origin. Non-octave hypermode capitalizes on the registral determination of functionality of the multitonal degrees (i.e. low VI degree is more stable than high VI degree, whereas low VII is more unstable than high VII), evolving it into a principal in tonal organization. However, it is difficult to draw a definite line as to which is the first music composed in hypermodal organization – monodic music makes it hard to tell whether a particular melody was conceived as a single complex modal entity or as a chain of discrete modulations between different modes. The definite integrative conception is obvious only in polyphonic composition. The earliest polyphonic implementation of non-octave hypermode is likely to have originated from Byzantine chant during the early Middle Ages in Georgia (Oniani 2010). [↑](#footnote-ref-12)
13. It is difficult to pinpoint the origin of the hemiolic mode, if to take as its birthmark the crystallization of the hemiolic tetrachord C-Db-E-F. Generally, its modal organization seems to be present in both, the folk music of the Mediterranean region and the local Orthodox chants, perhaps descending from the Ancient Greek chromatic genus by means of the Byzantine chant (Katsanevaki 2011) or simply preserving the hemiolic modification of the chromatic genus that occurred circa 1^st^ century BC (Pohlmann and West 2001, 58–9) via its dissolution in the local music traditions. There is some evidence that augmented 2^nd^ was reflected in the pre-Ottoman Byzantine music theory (Makris 2005). The hemiolic maqam Hijaz is first mentioned in a theory treatise in the 11^th^ century (Neubaer 1992, 597). The first music scores that document the modal use of gapped tetrachord come from the 18^th^ century popular Romanian piano music (Manuel 1989), and the arrangements of Spanish flamenco, which surfaced by about the same time, the late 18^th^ century (Leblon 2003, 105). [↑](#footnote-ref-13)
14. Of course, as with every other dichotomy in culture, only the poles are clear cut opposite – there are music cultures that demonstrate some extent of hybridization between pentatony and heptatony, expressed in hexatonic modes, or special cases of “segregated pentatony,” where its application is limited to melody, while keeping harmony heptatonic. [↑](#footnote-ref-14)
